# Clusters of Conserved Beta Cell Marker Genes for Assessment of Beta Cell Phenotype

**DOI:** 10.1371/journal.pone.0024134

**Published:** 2011-09-02

**Authors:** Geert A. Martens, Lei Jiang, Karine H. Hellemans, Geert Stangé, Harry Heimberg, Finn C. Nielsen, Olivier Sand, Jacques Van Helden, Frans K. Gorus, Daniel G. Pipeleers

**Affiliations:** 1 Diabetes Research Center, Vrije Universiteit Brussel (VUB), Brussels, Belgium; 2 Department of Clinical Chemistry and Radioimmunology, Universitair Ziekenhuis Brussel, Brussels, Belgium; 3 Microarray Facility, Rigshospitalet, Copenhagen, Denmark; 4 Bioinformatique des Génomes & des Réseaux (BiGRe), Université Libre de Bruxelles (ULB), Brussels, Belgium; University of Frankfurt - University Hospital Frankfurt, Germany

## Abstract

**Background and Methodology:**

The aim of this study was to establish a gene expression blueprint of pancreatic beta cells conserved from rodents to humans and to evaluate its applicability to assess shifts in the beta cell differentiated state. Genome-wide mRNA expression profiles of isolated beta cells were compared to those of a large panel of other tissue and cell types, and transcripts with beta cell-abundant and -selective expression were identified. Iteration of this analysis in mouse, rat and human tissues generated a panel of conserved beta cell biomarkers. This panel was then used to compare isolated versus laser capture microdissected beta cells, monitor adaptations of the beta cell phenotype to fasting, and retrieve possible conserved transcriptional regulators.

**Principal Findings:**

A panel of 332 conserved beta cell biomarker genes was found to discriminate both isolated and laser capture microdissected beta cells from all other examined cell types. Of all conserved beta cell-markers, 15% were strongly beta cell-selective and functionally associated to hormone processing, 15% were shared with neuronal cells and associated to regulated synaptic vesicle transport and 30% with immune plus gut mucosal tissues reflecting active protein synthesis. Fasting specifically down-regulated the latter cluster, but preserved the neuronal and strongly beta cell-selective traits, indicating preserved differentiated state. Analysis of consensus binding site enrichment indicated major roles of CREB/ATF and various nutrient- or redox-regulated transcription factors in maintenance of differentiated beta cell phenotype.

**Conclusions:**

Conserved beta cell marker genes contain major gene clusters defined by their beta cell selectivity or by their additional abundance in either neural cells or in immune plus gut mucosal cells. This panel can be used as a template to identify changes in the differentiated state of beta cells.

## Introduction

In this study we tried to establish a blueprint of the pancreatic beta cell transcriptome conserved from rodents to humans. Identification of novel beta cell-selective biomarkers can have several (pre)clinical applications. Beta cell-selectively expressed genes are likely important for beta cell survival and specialized functions and could yield novel targets for drugs that regulate glucose-sensing and insulin synthetic capacity of the beta cell. They could also serve as candidate biomarkers for diagnostic purposes, e.g. beta cell surface proteins for in vivo imaging of beta cell mass, or to find targets for assays that detect beta cell biomarkers that are specifically discharged from dying beta cells in analogy to GAD65 [1]. A comprehensive view on beta cell-selective biomarkers can also guide the preclinical development of novel types of transplantable beta cell grafts: to solve the current shortage in donor beta cells, studies are undertaken to generate functional beta cells through differentiation of stem cells or reprogramming of developmentally related cell types [2]. These lab-generated beta cell preparations need to be extensively evaluated in the preclinical phase, to ensure that their gene and protein expression profiles and functional properties closely resemble primary beta cells.

In the present study we used Affymetrix oligonucleotide arrays to record the transcriptome of freshly isolated highly FACS-purified rat beta cells (>90% insulin+), freshly isolated mouse islets and cultured human beta cells obtained from 10 donor organs and FACS-enriched to ±55% insulin-positivity. In each species we compared these to a large panel of mRNA profiles of other primary tissues or cell types, and selected transcripts with relatively abundant expression in the beta cell preparations. To filter out inevitable experimental noise associated with each of these comparisons, we focused only on marker genes that had a beta cell-selective and – abundant expression in the three model species. Notwithstanding the fact that there are proven differences in beta cell function and gene expression between rodents and man, our focus on evolutionary conserved features serves as additional argument for likely biological significance of identified beta cell biomarkers.

In a second part of the study, the experimental usefulness of the resulting conserved beta cell marker genes was evaluated. First, the panel was used to compare beta cell preparations obtained by cell isolation or in situ laser capture microdissection. Second, the panel was used to examine how beta cell differentiated states is dynamically regulated by fasting. Third, two different bioinformatics algorithms were used to identify likely conserved transcriptional regulators in the conserved beta cell biomarkers. Finally, we confirmed beta cell-selective expression of the corresponding proteins using quantitative LC-MS/MS analysis of unfractionated beta cell proteomes and immunohistochemistry on pancreatic sections. Protein phosphatase 1, regulatory (inhibitor) subunit 1A (PPP1RIA) came out as a novel beta cell marker with high molar protein abundance and beta cell-restricted expression in the pancreas.

## Results

### Selection of conserved beta cell marker genes

**Figure 1:**
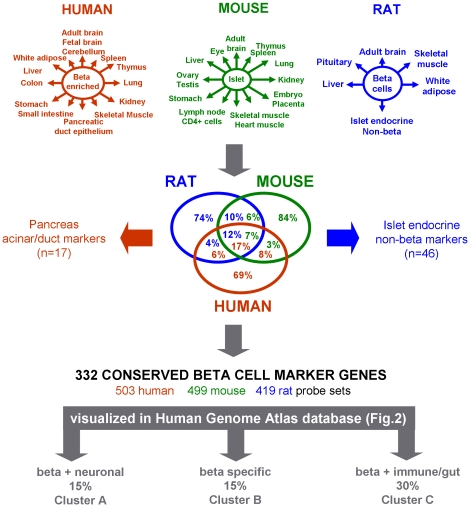
Selection algorithm of conserved beta cell marker genes. Beta cell preparations were compared to the indicated tissues in mouse, rat and human and genes with conserved beta cell-abundant expression selected (Venn diagram intersect). The rat data set was used to discard 46 islet endocrine non-beta cell (mainly alpha cell) markers; the human data set enabled curation of 17 pancreatic exocrine (acinar and duct) markers. Final set of conserved beta cell marker genes consists of 332 genes, represented by 419 (rat), 499 (mouse) or 503 (human) oligonucleotide probe sets.

In each of the three examined species, we selected a set of 2500 transcripts with higher expression in beta cells than in other tissues (from 20% to 100-fold higher). Intersects were sought with common annotated genes (Fig. 1) and only transcripts with conserved relative beta cell abundance were further analyzed. This restriction lowers comprehensiveness (sensitivity), but increases robustness (specificity) of the resulting 395 candidate beta cell marker transcripts, which represent 16% (mouse) to 31% (human) of initial input genes. We then removed transcripts that can be attributed to the pancreatic non-beta cell types that contaminate beta cell preparations. We used mRNA profiles of FACS-purified rat islet endocrine non-beta cells to remove 46 transcripts that are at least 1.5-fold higher in alpha than in beta cells (p<0.05, n = 3, e.g. hormone-coding genes *GLUC*, *PPY* and *SST*). The human data sets enabled removal of transcripts typical for exocrine duct (n = 5) or acinar (n = 12) cells. As shown in Fig. S1, the contamination by exocrine-associated genes is much lower in FACS-purified human beta cells than in the whole islet-preparations. The resulting panel of 332 genes with conserved beta cell-abundant expression (Table S1, further referred to as beta cell marker genes) is represented by 419, 499 or 503 probe sets on the rat, mouse and human arrays respectively.

### Distribution of conserved beta cell marker genes over three clusters with different tissue selectivity

**Figure 2:**
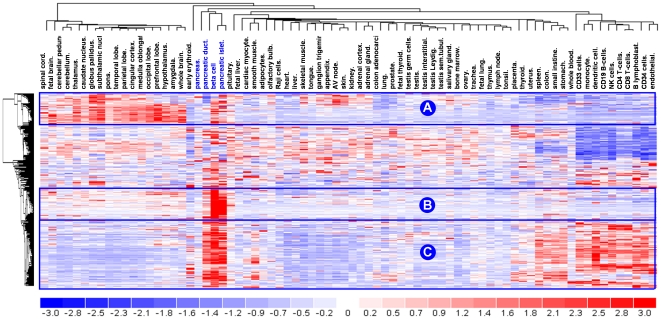
Shared gene clusters between beta cells, neuronal cells and immune cells. The 503 human probe sets corresponding to the 332 conserved beta cell marker genes are visualized by hierarchical clustering in the Human Genome Atlas data set [3]. At least 15% of beta cell marker genes show near absolute beta cell selectivity (beta cell selective **cluster B**). Beta cells also share an equally extensive cluster (15%) with various brain regions (neuroendocrine **cluster A**), and an even larger cluster (30%) with immune and gut mucosal cells (gut/immunological **cluster C**). Statistical enrichment analysis indicates that these clusters accentuate different functions proper to the specialized beta cell phenotype. Blue to red correspond low to high relative expression, as shown in legend (bottom). Relevant genes of clusters A, B and C are shown in Fig. 3.

The 332 beta cell marker genes were visualized for their natural tissue tropism in the context of the Human Gene Atlas [3], a public repository of mRNA expression profiles of human organs or cell types. Beta cell-selectivity was independently confirmed for most markers (Figure 2). The hierarchical cluster graph highlights that most beta cell marker genes are also actively transcribed in other cell or tissue types and few genes can be called beta cell-specific. Combined however, our beta cell marker gene list clearly discriminates beta cells from all other examined cell types: this is graphically illustrated by the cluster tree in Figure 2 and its associated principle component analysis (Fig. S2). In terms of their overall tissue-selectivity, at least 60% of beta cell marker genes fall into three main clusters; i.e. a neuroendocrine cluster (cluster A), a beta cell cluster (cluster B) and a immune/gut enriched cluster (cluster C) (Fig. 2).

### Beta cell marker genes with high degree of beta cell-selectivity

**Figure 3:**
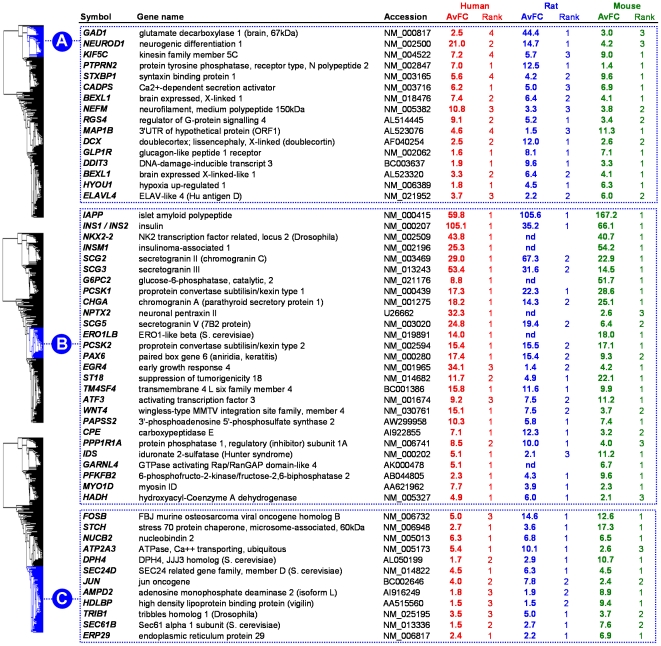
Examples of beta cell marker genes belonging to neuroendocrine cluster A, beta cell selective cluster B and beta-immune cluster C. Average fold change (AvFC) indicates the relative abundance of a given mRNA in the beta cell as compared to the other tested tissues in that species, while lower rank value reflects higher beta cell selectivity (see methods). From each tissue-tropic cluster (A, B, C from Fig. 2), beta cell marker genes with, calculated for the 3 species, an averaged avFC score ≥4, are shown. Relative beta cell abundance (and selectivity) decreases from cluster B over cluster A to cluster C, and consequently this is reflected in number of retained exemplary genes.

At least 15% of conserved beta marker genes (n = 48) show near absolute beta cell-selectivity (beta cell selective cluster B in Fig. 2, exemplary genes shown in Fig. 3 and Fig. S4). This cluster contains established beta cell markers such as hormones (*INS2 IAPP*) and hormone processing enzymes (*PCSK1, CPE*), transcription factors (*NKX2-2, INSM1*), metabolic enzymes (*HADH, G6PC2, PFKFB2*) and secretogranin proteins. It also reveals novel beta cell markers such as putative tumor suppressor *ST18*, phosphatase regulator *PPP1R1A*, ketone body-metabolizing enzyme *AACS* and several RAB/RAS GTPase-associated proteins (*RRAGD, RAB2A, GARNL4*)., mRNA expression of these markers is 10 to 15-fold higher in beta cells than in other tissues. The cluster is statistically enriched (p<0.001) in ontologies of *hormone processing*, *protein folding* and *secretory granules* (Fig. S4).

### Beta cell marker genes sharing selectivity with neuronal cells

At least 15% of beta cell marker genes (n = 47) are also abundant in various brain regions (cluster A in Fig. 2, exemplary genes shown in upper panel Fig. 3). Some have been previously reported as beta cell markers (e.g. *PTPRN2* alias IA-2β, *DDIT3* and *GLP1R*); others were so far considered as brain-selective. This cluster is enriched (p<0.001) in ontologies such as *synaptic vesicle*, *neurotransmitter transport* and *nervous system development*. Examples of genes involved in neuronal development or migration are *NEUROD, ROBO1*, *DCX* and *PAFAH1B1*. Genes belonging to this beta-cell cluster were found to be particularly transcribed in the dopaminergic neurons of the globus pallidus (GP) and the glutaminergic/GABAergic neurons of the subthalamic nucleus (STN) (Fig. S3). An exemplary marker is dopa decarboxylase (*DCC*), rate-limiting enzyme for biogenic amine synthesis, of which the mRNA is 20-fold more abundant in beta cells than in whole brain (Table 1).

**Table 1:** qPCR analysis of beta cell marker genes in rat tissues.

Table 1	Beta		Alpha		Pancreas		Brain		Duod.		Liver		WAT		Muscle		WBC	
Gene	GEO	SD	GEO	SD	GEO	SD	GEO	SD	GEO	SD	GEO	SD	GEO	SD	GEO	SD	GEO	SD
***INS2***	**20576**	914	**413**	63	**636**	26	**0.0**	0.0	**0.2**	0.0	**nd**		**nd**		**nd**		**nd**	
***IAPP***	**999**	56	**134**	25	**56.8**	4	**nd**		**nd**		**nd**		**nd**		**nd**		**nd**	
***SLC2A2***	**143.7**	8.5	**11.1**	1.7	**nd**		**0.0**	0.0	**5.0**	0.1	**1.8**	0.1	**nd**		**nd**		**nd**	
***PTPRN***	**137.1**	8.7	**98.0**	18.2	**4.5**	0.2	**18.2**	3.0	**0.0**	0.0	**0.0**	0.0	**0.1**	0.1	**0.4**	0.1	**0.0**	0.0
***CPE***	**133.1**	5.3	**46.4**	6.4	**12.6**	1.1	**27.2**	1.1	**0.9**	0.0	**nd**		**nd**		**5.9**	1.3	**0.0**	0.0
***PCSK1***	**26.1**	1.2	**8.1**	5.1	**nd**		**0.3**	0.0	**0.1**	0.0	**nd**		**nd**		**nd**		**0.1**	0.1
***SCG5***	**22.8**	0.9	**25.4**	5.9	**2.5**	0.2	**5.6**	0.1	**0.0**	0.0	**nd**		**nd**		**nd**		**nd**	
***PPP1R1A***	**17.9**	0.6	**1.3**	0.5	**2.0**	0.1	**1.9**	0.2	**0.0**	0.0	**nd**		**nd**		**12.0**	4.5	**nd**	
***DCX***	**14.6**	0.4	**2.4**	0.3	**0.4**	0.1	**0.7**	0.1	**0.0**	0.0	**0.0**	0.0	**nd**		**nd**		**nd**	
***HYOU1***	**9.2**	0.2	**1.3**	0.6	**6.3**	0.1	**0.7**	0.0	**0.3**	0.0	**5.1**	0.2	**8.5**	13.7	**2.6**	0.3	**2.1**	1.7
***FOSB***	**8.4**	1.4	**3.3**	1.4	**nd**		**0.3**	0.1	**0.0**	0.0	**nd**		**nd**		**nd**		**nd**	
***DDC***	**7.1**	0.6	**2.5**	0.6	**1.5**	0.1	**0.3**	0.0	**2.4**	0.2	**1.5**	0.1	**nd**		**0.1**	0.0	**0.0**	0.0
***PDX1***	**5.8**	0.5	**0.5**	0.2	**2.0**	0.2	**nd**		**0.0**	0.0	**0.0**	0.0	**nd**		**nd**		**nd**	
***HADHSC***	**4.8**	0.2	**0.6**	0.1	**1.1**	0.1	**0.1**	0.0	**0.7**	0.0	**0.8**	0.0	**0.2**	0.2	**1.0**	0.1	**0.1**	0.1
***WNT4***	**3.7**	0.1	**1.0**	0.8	**0.4**	0.0	**0.1**	0.0	**0.0**	0.0	**0.1**	0.0	**1.2**	1.2	**5.1**	0.8	**0.0**	0.0
***SCG2***	**3.6**	0.2	**1.7**	1.1	**0.2**	0.0	**0.2**	0.0	**0.0**	0.0	**nd**		**nd**		**nd**		**nd**	
***ST18***	**3.5**	0.2	**nd**		**nd**		**0.0**	0.0	**nd**		**nd**		**nd**		**nd**		**nd**	
***SYTL4***	**3.3**	0.1	**0.9**	0.6	**0.3**	0.0	**0.0**	0.0	**0.0**	0.0	**0.0**	0.0	**0.3**	0.3	**0.1**	0.0	**0.0**	0.0
***ARMET***	**3.0**	0.1	**0.4**	0.3	**3.0**	0.4	**0.2**	0.0	**0.3**	0.0	**0.5**	0.0	**nd**		**0.3**	0.0	**0.2**	0.1
***GPSM1***	**2.5**	0.1	**0.5**	0.1	**0.1**	0.0	**0.4**	0.0	**0.0**	0.0	**0.0**	0.0	**0.2**	0.2	**0.1**	0.0	**0.1**	0.1
***GAD2***	**2.1**	0.2	**0.0**	0.0	**0.1**	0.0	**5.3**	0.1	**0.0**	0.0	**nd**		**nd**		**nd**		**nd**	
***NNAT***	**1.0**	0.0	**0.5**	0.2	**0.1**	0.0	**5.5**	0.4	**0.0**	0.0	**0.0**	0.0	**0.0**	0.0	**0.4**	0.1	**nd**	
***NEUROD1***	**0.7**	0.0	**0.0**	0.0	**0.1**	0.0	**0.0**	0.0	**0.0**	0.0	**nd**		**nd**		**nd**		**nd**	
***GCK***	**0.6**	0.0	**0.8**	0.1	**0.1**	0.0	**0.0**	0.0	**0.0**	0.0	**0.2**	0.0	**nd**		**nd**		**nd**	
***NPY***	**0.6**	0.0	**0.0**	0.0	**nd**		**0.9**	0.1	**0.0**	0.0	**0.0**	0.0	**nd**		**nd**		**nd**	
***DDIT3***	**0.5**	0.0	**0.4**	0.1	**0.1**	0.0	**0.1**	0.0	**0.1**	0.0	**0.0**	0.0	**nd**		**0.2**	0.1	**0.2**	0.1
***NKX2-2***	**0.2**	0.0	**nd**		**0.1**	0.0	**0.0**	0.0	**0.0**	0.0	**nd**		**nd**		**nd**		**nd**	
***GCG***	**46**	1.5	**2711**	601	**170**	6.6	**0.0**	0.0	**0.4**	0.0	**nd**		**nd**		**0.1**	0.0	**nd**	
***CTRB1***	**341**	17.0	**304**	48	**30043**	1145	**0.0**	0.0	**2.3**	0.0	**0.0**	0.0	**nd**		**nd**		**0.1**	0.1

mRNA expression levels are expressed as geometric mean (GEO) ± SD (n = 3) of indicated markers normalized to 4 reference genes (*BACT, UBC, PPIA, PSMC5*). Marker genes are sorted by decreasing order of their mRNA abundance in beta cells. Duod., duodenum; WAT, white adipose tissue (abdominal); WBC, white blood cells.

### Beta cell marker genes sharing selectivity with immune and gut mucosa cells

Roughly 30% (n = 99) of beta cell marker genes appear also actively transcribed in immune cells, and to a lesser extent in cells of the gut mucosa (cluster C in Fig. 2, examples in Fig. 3). Functionally these genes are enriched (p<0.001) in pathways such as *response to stress*, *small GTPase*, *protein folding* and *endoplasmic reticulum*, reflecting a role of many of these marker genes in the synthesis and proper folding of secretory proteins in the endoplasmic reticulum and Golgi. Compared to the other two clusters, it shows a more moderate beta cell-selectivity with only a 2 to 3-fold higher expression in beta cells than in other tissues.

### Quantitative real-time PCR confirmation of novel beta cell markers

Beta cell selectivity of 27 marker genes – positive controls as well as novel candidate markers from the three clusters – was verified by qPCR in the rat model (Table 1). Use of geometric normalization of marker mRNAs to 4 validated reference genes [4] allows reliable quantification of mRNA abundances both within and between different cell types. The selected beta cell marker genes show large variations in their mRNA levels, with the most abundant mRNAs *INS2* and *IAPP* respectively 10^5^ and 5×10^3^ more abundant than the least abundant one, that of transcription factor *NKX2.2*. Of note, none of the genes with the exception of *IAPP* shows a truly beta cell-selective expression, since low hybridization signals were detected in other tissues, notably brain and gut. The strongest novel markers are *ST18* – with undetectable expression in all other cell types except brain - and *DCX*, *PPP1R1A* and *FOSB* – which are detected in other tissues but at much lower levels than in beta cells. Other markers show abundant expression in islet endocrine cells with weak or no selectivity towards either beta or alpha cell lineage, such as *GCK*, *PTPRN* (IA2) and *SCG5*.

### Conserved beta cell markers obtained by analysis of isolated beta cells also mark beta cells obtained by laser capture microdissection

**Figure 4:**
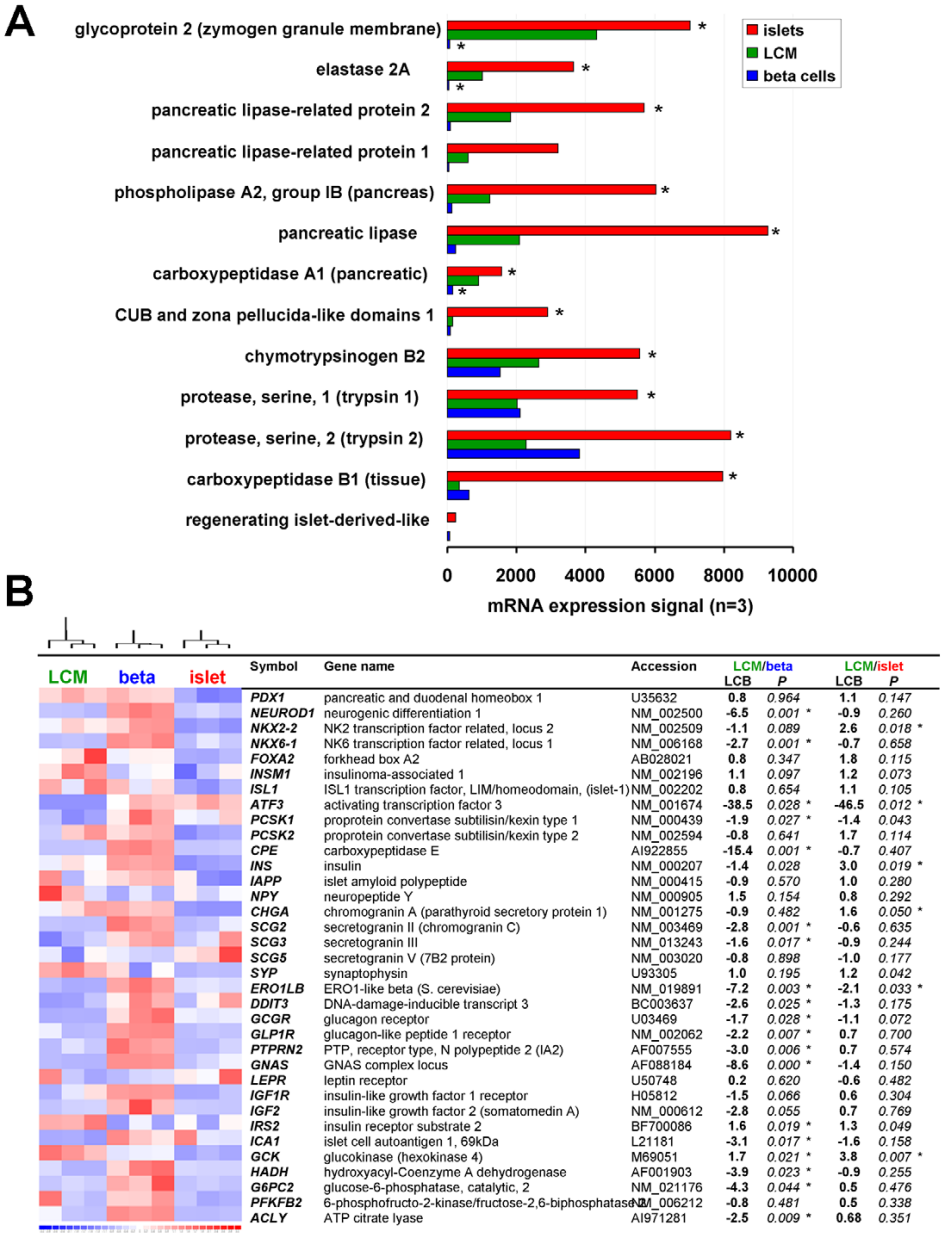
Conserved beta cell marker genes in isolated versus laser capture microdissected human beta cells. Panel A shows mRNA expression signal on HG133A chip of 13 established pancreatic exocrine (acinar) genes, in freshly isolated human islets (islets, red bars, n = 3), laser capture microdissected beta cells (LCM, green bars, n = 3) and 2–3 w cultured beta cells that were subsequently FACS-enriched (beta cells, blue bars, n = 3). * p<0.05 versus LCM. Panel B shows relative mRNA levels established beta cell-specific genes in cultured beta (blue), fresh islets (red) and LCM-beta cells (green). Table shows fold change (LCB, lower confidence bound) and P values of versus LCM-beta.

The panel of conserved beta cell markers was obtained by study of isolated beta cell preparations. Cell isolation entails hypoxia, mechanical stress, disruption of intercellular and cell-to-matrix interactions and possibly contamination by exocrine pancreatic cells. Since associated biases on gene expression are reportedly less pronounced in beta cells obtained by laser capture microdissection (LCM) [5], we compared mRNA profiles of cultured human beta cells to published [5] profiles of freshly isolated islets and laser capture microdissected (LCM)-beta cells. First we quantified exocrine contamination. LCM-beta cells indeed showed 7-fold lower mRNA expression of 13 acinar markers (p<0.05 for 9/13, n = 3) than freshly isolated islets but they expressed on the average 13-fold higher levels (p<0.05 for 3/13, range −2.8 to 73-fold) than cultured beta cells (Fig. 4a).

Next we looked at signs of isolation-associated cell stress and found no statistical enrichment in cultured beta cells of clusters involved in HIF alpha signaling, TNF signaling or MAPK/ERK signaling as reported for freshly isolated islets [5]. Cultured beta cells did show however a significant (p<0.001) activation of genes involved in *unfolded protein binding*, but this was accompanied by significant (p<0.001) up-regulation of clusters with role in *mRNA splicing in the spliceosome*, *ribosomal subunits*, *translation initiation*, *protein transport*, *ER to Golgi transport* and *vesicle-mediated secretion* (Table S2), reflecting concerted activation of the protein synthetic machinery rather than unfolded protein response due to cell stress. This associated with higher expression of genes coding for mitochondrial metabolic enzymes (Table S2).

Third, we compared isolated and LCM-beta cells for mRNA level of classical beta cell marker genes and observed that most established beta cell markers were more abundantly expressed in cultured beta cells, with mRNAs of *NEUROD1*, *NKX6.1*, *PCSK1*, *HADH*, *G6PC2* and *GLP1R* 1.5 to 4-fold up (*p<0.05*, Fig. 4B). For *ATF3* differences were more dramatic, with ±40-fold (p<0.03) higher levels both in isolated islets and beta cells.

Finally, when compared to other human tissues, our panel of conserved beta cell marker genes proved also abundantly expressed in LCM beta cells (not shown), and consequently this gene panel could equally well discern LCM and cultured beta cells from all other tissue types, as shown in PCA graph in Fig. S2.

### Use of conserved beta cell marker genes to identify transcription factors important for maintenance of beta cell differentiated state

**Figure 5:**
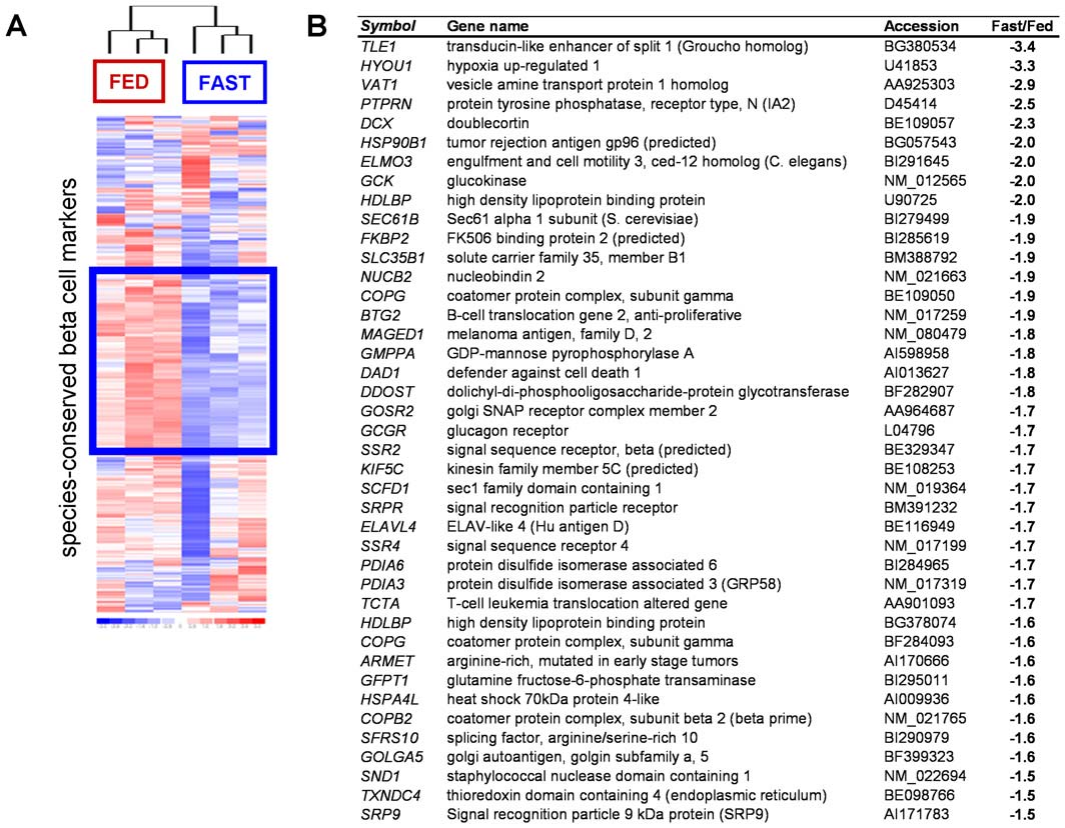
In rat beta cells fasting down-regulates cluster C genes with role in ER function and protein processing. Heat map in Fig. 5A shows the complete set of species-conserved beta cell markers, visualized in FACS-purified beta cells freshly isolated from control (FED) and 24-h fasted (FAST) rats; one third is reproducibly ≥1.25-times down-regulated (blue window) most of which statistically significantly (21% of all beta markers, p<0.05, n = 3). Table in panel B shows the 42 beta markers that are at least 1.5-fold down-regulated (p<0.05) by fasting: 50% of the fasting-suppressed beta cell marker genes belong to cluster C, also functionally enriched in pathways of protein folding and processing through ER/Golgi.

Next we used the conserved biomarkers to identify corresponding conserved transcriptional regulators. Two complementary algorithms were used for identification of statistically overrepresented transcription factor binding sites. First, the DiRE algorithm [6] was used to scan for cis-regulatory motifs in conserved promoter regions. This analysis was run on the full panel of beta cell marker genes as well as on the three main clusters in terms of tissue-tropism (Table S3). In a fully complementary strategy, candidate promoter regions were searched for conserved enrichment using a matrix-scan approach [7] (Table S4, Fig. 5). Motifs identified by both strategies (Fig. 5, Venn diagram intersect) include members of the Creb/Atf (Creb, Atf6, Atf3), Pax (Pax4 or Pax6) and Fox families, established beta cell transcription factors Nkx2-2 and Pdx1 and several transcriptional regulators with likely role in nutrient-redox regulated gene expression: upstream factors (Usf), Arnt/HIF, yingyang 1 (YY1), Xbp1, Oct1, Nfat and Stra13. Other overrepresented binding sites are those for transcription factors mostly studied in relation to blood (Tal1, Meis, Hoxa9, Gfi1b) or muscle (Myogenin, Tbx5, Rfx1, Mrf2) homeostasis. Of these, Atf3, Pdx1 and Nkx2.2 are also beta cell-abundant at the mRNA level. This is also the case for Foxa2, Nkx6.1, MyoD1, c-Jun and FosB, which are only identified by the DiRE algorithm.

### Use of conserved beta cell marker genes to evaluate effect of fasting on beta cell differentiated state and functional phenotype

**Figure 6:**
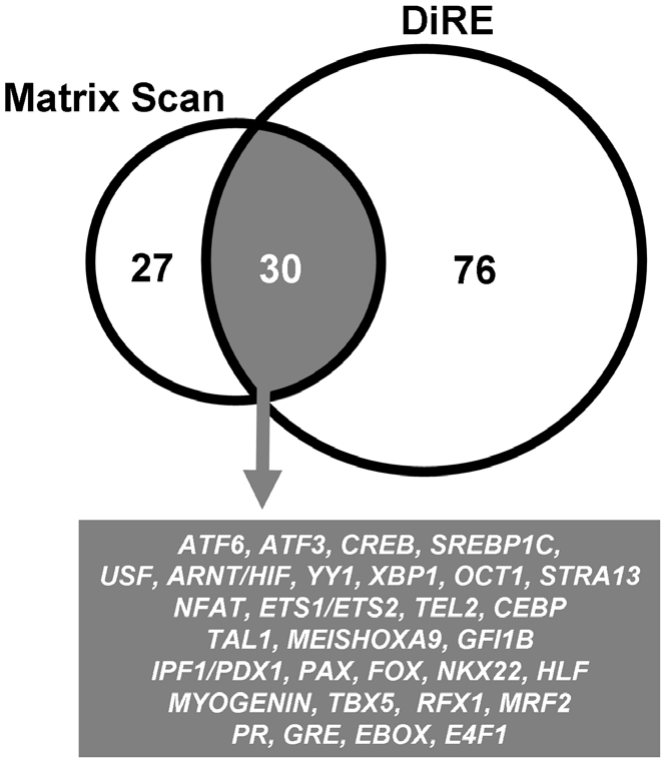
Transcription factor binding sites overrepresented in the conserved beta cell marker gene set. Using two complementary search algorithms (Matrix Scan and DiRE), upstream promoter regions were scanned for statistical enrichment of TRANSFAC-annotated transcription factor binding consensus sites. Venn diagrams indicate number of identified matrices by each program, and with 30 commonly identified matrices indicated in gray field. See Tables S3 and S4.

Short periods of fasting are thought to mediate the beta cells' competence for insulin synthesis and secretion [8]. We examined whether it influenced their typical mRNA expression. After 24 h of fasting, a 1.5 fold change was detected in 1.5% of all assayed transcripts (p<0.05), with 95% of altered genes (430 of 452) being down-regulated (Fig. 6A). Suppression was more marked for the beta cell marker genes of which 12% were 1.5-fold suppressed (42 transcripts, Fig. 6B). Of these 42 transcripts, 21 expressed genes of *endoplasmic reticulum (ER)*, *ER to Golgi transport*, *protein folding* and *secretory vesicle-mediated transport*, i.e. the pathways of protein synthesis and transport. These genes mostly belonged to cluster C which shared genes with immune and gut mucosa cells (Fig. 2). In contrast few changes occurred in the two other clusters: 4 genes down-regulated in the beta cell selective cluster, and only 1 in the cluster sharing selectivity with neural cells.

### Confirmation of conserved beta cell marker genes at the protein level

We previously reported a quantitative LC-MS proteome analysis of FACS-purified rat beta and alpha cells, in comparison to liver and brain tissues [9]. Analysis of unfractionated cell proteomes is biased towards the proteins with higher molar abundance: only 38 proteins coded by the set of 332 beta cell marker genes (11%) were detected (Table 2). Of these, 12 proteins (32%) were uniquely detected in the beta cells, at relatively high molar levels (13 to 105 times above the MS quantification limit). An additional 9 markers (24%) appeared islet-restricted. 17 (45%) proteins were also detected in liver and/or brain tissues but typically at lower abundances than in beta cells. Overall 74% of detected proteins indeed showed highest abundance in beta cells. Those that did not were typically shared by alpha and beta cells with a molar predilection for the alpha cell (e.g. the secretogranins 2, 3 and 5).

**Table 2:** Conserved beta cell marker genes identified in rat beta cells, alpha cells, liver and brain tissue using quantitative proteomics.

Accession	Symbol	Alpha	Beta	Brain	Liver	Protein full name	mRNA	protein	mRNA	protein
		relative molar abundance (fmolr)					AvFC	fold of LLQ	Rank	Rank
O35165	**Gosr2**	-	0.00527	-	-	Golgi SNAP receptor complex member 2	1.8	105	1	1
Q7M0E3	**Dstn**	-	0.00329	-	-	Destrin	2.8	66	1	1
O35142	**Copb2**	-	0.00223	-	-	Coatomer subunit beta	1.9	45	1	1
P19103	**Ppp1r1a**	-	0.00219	-	-	Protein phosphatase 1, regulatory (inhibitor) subunit 1A	10.0	44	1	1
P28840	**Pcsk1**	-	0.00195	-	-	proprotein convertase subtilisin/kexin type 1	22.3	39	1	1
P0C5H9	**Armet**	-	0.00168	-	-	Protein ARMET	3.5	34	1	1
Q6AYG5	**Echdc1**	-	0.00166	-	-	Enoyl CoA hydratase domain containing protein 1	1.7	33	2	1
P35571	**Gpd2**	-	0.00149	-	-	Glycerol 3 phosphate dehydrogenase mitochondrial	1.4	30	3	1
Q920J4	**Txnl1**	-	0.00120	-	-	Thioredoxin like protein 1	1.5	24	2	1
P12336	**Slc2a2**	-	0.00091	-	-	Solute carrier family 2 facilitated glucose transporter member 2	28.6	18	1	1
Q9R064	**Gorasp2**	-	0.00079	-	-	Golgi reassembly stacking protein 2	1.5	16	1	1
Q9ESI7	**Dcx**	-	0.00067	-	-	Neuronal migration protein doublecortin	12.0	13	1	1
Q9WVK7	**Hadh**	0.00162	0.01151	-	-	Hydroxyacyl coenzyme A dehydrogenase mitochondrial	6.0	230	1	1
P12969	**Iapp**	0.00252	0.00809	-	-	Islet amyloid polypeptide	105.6	162	1	1
P15087	**Cpe**	0.00809	0.00641	-	-	Carboxypeptidase E	12.3	128	1	2
P10362	**Scg2**	0.01099	0.00295	-	-	Secretogranin 2	67.3	59	2	2
P10354	**Chga**	0.00401	0.00254	-	-	Chromogranin A	14.3	51	2	2
P27682	**Scg5**	0.01081	0.00242	-	-	Neuroendocrine protein 7B2	19.4	48	2	2
Q4AEF8	**Copg**	0.00099	0.00228	-	-	Coatomer subunit gamma	2.1	46	1	1
P28841	**Pcsk2**	0.00901	0.00222	-	-	Neuroendocrine convertase 2	15.5	44	2	2
P47868	**Scg3**	0.00583	0.00104	-	-	Secretogranin 3	31.6	21	2	2
P61765	**Stxbp1**	-	0.00136	0.00420	-	Syntaxin binding protein 1	4.2	27	2	1
O35303	**Dnm1l**	-	0.00075	0.00089	-	Dynamin 1 like protein	1.4	15	3	2
O88600	**Hspa4**	0.00111	0.00169	0.00072	-	Heat shock 70 kDa protein 4	1.6	34	1	1
P61751	**Arf4**	0.00125	0.00106	0.00104	-	ADP ribosylation factor 4	2.0	21	1	2
Q62902	**Lman1**	-	0.00354	-	0.00227	Protein ERGIC 53	3.9	71	1	1
Q66HD0	**Hsp90b1**	0.00547	0.01348	-	0.00500	Endoplasmin	2.7	270	1	1
Q63081	**Pdia6**	0.00410	0.01173	-	0.00382	Protein disulfide isomerase A6	2.8	235	1	1
Q63617	**Hyou1**	0.00251	0.00836	-	0.00098	Hypoxia up regulated protein 1	4.5	167	1	1
Q9JI85	**Nucb2**	0.00155	0.00272	-	0.00057	Nucleobindin 2	6.8	54	1	1
Q66X93	**Snd1**	0.00090	0.00146	-	0.00148	Staphylococcal nuclease domain containing protein 1	2.1	29	1	2
P06761	**Hspa5**	0.00656	0.02272	0.00109	0.00913	78 kDa glucose regulated protein	2.9	454	1	1
P11598	**Pdia3**	0.00643	0.01311	0.00059	0.00631	Protein disulfide isomerase A3	2.7	262	1	1
Q6NYB7	**Rab1A**	0.00345	0.00347	0.00123	0.00129	Ras related protein Rab 1A	1.6	69	1	1
P18596	**Atp2a3**	0.00085	0.00307	0.00031	0.00051	Sarcoplasmic endoplasmic reticulum calcium ATPase 3	10.1	61	1	1
P05712	**Rab2a**	0.00221	0.00236	0.00127	0.00026	Ras related protein Rab 2A	1.4	47	1	1
P11507	**Atp2a2**	0.00109	0.00201	0.00131	0.00083	Sarcoplasmic endoplasmic reticulum calcium ATPase 2	2.4	40	2	1
Q63941	**Rab3b**	0.00186	0.00092	0.00050	<LLQ	Ras related protein Rab 3B	1.9	18	2	2
Lower Limit of Quantification (LLQ) = 0.00005										

Data represent relative molar amounts of proteins coded by beta cell marker genes that could be detected in label-free alternate-scanning LC-MS proteomics of unfractionated cells and tissues (mean of triplicate injections of 3 biological replicates, total CV%<20% [9]). For comparison avFC and rank of mRNA transcript (array) are compared to protein abundance (fold above lower limit of quantification, LLQ) and rank (see Methods and reference [9] for details).

**Figure 7:**
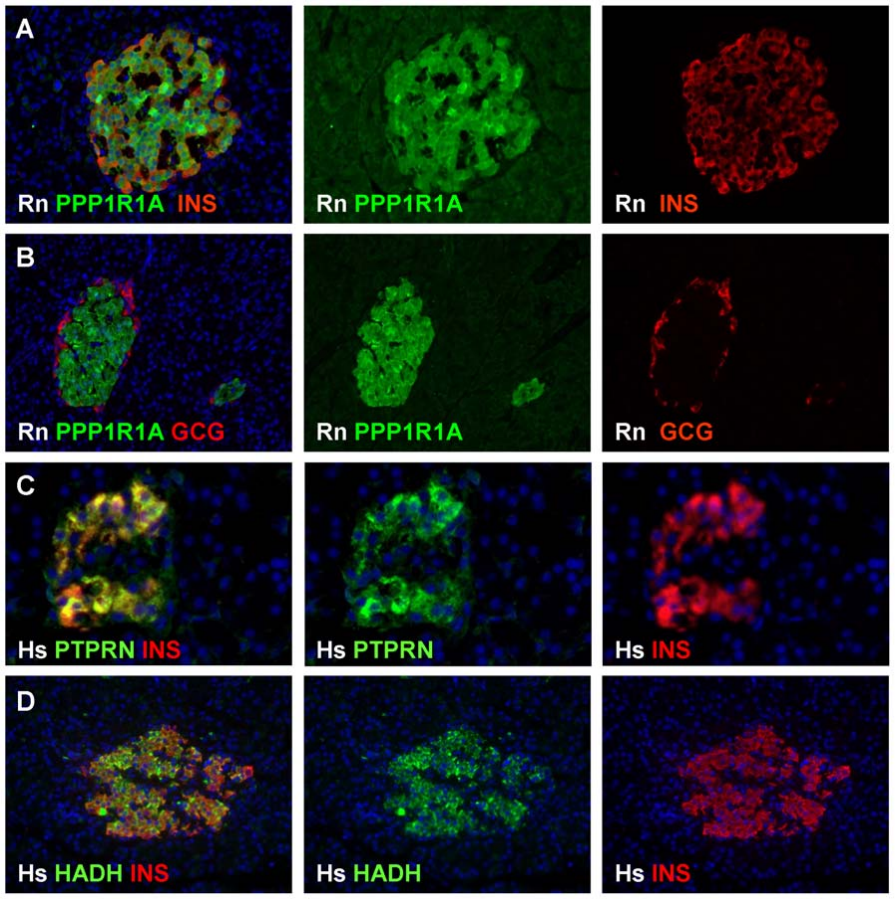
*In situ* analysis of beta cell-selective expression of PPP1R1A, PTPRN and HADH proteins. Panels A and B respectively show abundant expression of PPP1R1A protein in insulin+ cells and absence in glucagon+ cells in islets from 10 w old rats. Beta cell-selective expression of PTPRN and HADH proteins in human beta cells is illustrated in panels C and D respectively. All images are representative of at least 3 different organs with at least 3 sections analyzed per pancreas.

Protein phosphatase 1, regulatory (inhibitor) subunit 1A (PPP1RIA) came out as a novel marker with high molar protein abundance specifically in beta cells. Molar Ppp1r1a abundance was similar to that of the established beta cell marker proprotein convertase subtilisin/kexin type 1 (*PCSK1*) and 30% of islet amyloid polypeptide (*IAPP*) abundance (Table 2). In situ analysis in rat pancreas confirmed a restricted expression in insulin+ cells with no protein detected in glucagon+ cells or surrounding exocrine tissue (Fig. 7 A–B). In situ analysis in human pancreas also confirmed beta cell-selective expression of protein tyrosine phosphatase, receptor type, N (*PTPRN*) alias IA2 (Fig. 7C). Both markers display an equal beta cell-selectivity as the beta-oxidation enzyme L-3-hydroxyacyl-CoA dehydrogenase (HADH), shown in human pancreatic sections in Fig. 7D.

## Discussion

### Novelty of our beta cell biomarker selection approach

Previous studies have generated gene lists characteristic for mouse [10], rat [11]–[13] or human [5], [12]–[16] pancreatic islets, which represent a resource for novel beta cell biomarkers. Our study advances these efforts for three reasons.

*First*, rather than using a rigid score of mRNA expression as absent/present, a more permissive algorithm was used that highlights mRNAs with a higher averaged abundance in beta cells as compared to other tissues and cell types. It zooms in on genes that are apparently actively transcribed in beta cells without excluding genes that also have a high expression in other specialized cells. Indeed, virtually all beta cell markers showed a varying degree of cell type ‘tropism’ or ‘selectivity’ and can rarely be considered absolutely beta cell-specific.

*Second*, our combined data sets made it possible to control for ‘contaminating’ gene markers of other cell types present in pancreas. Though it is theoretically not excluded that the gene clusters shared with neuronal or immune/gut tissues arise partly from contamination with exocrine cells, neurons, endothelial or blood cells that are co-isolated along with the beta cells, this is unlikely for several reasons: *(i)* beta cell marker genes were selected for their higher expression in purified beta cell preparations – particularly in the rat with FACS-purifications >90% insulin-positivity – to whole, freshly frozen tissues. The latter are more likely to contain blood vessels and blood cells, and also nerve fibers and ganglia dispersed in the tissue parenchyma, since beta cell FACS-sorting according to cellular flavin adenine dinucleotide content not only discriminates islet beta cells from islet endocrine non-beta-cells but also from other cell types such as fibroblasts, leukocytes, and exocrine cells [17]. *(ii)* The human beta cells were FACS-isolated from endocrine aggregates after sustained (2–3 weeks) culture, a condition that does not well support survival of differentiated endothelial, blood and neuronal cells unless specific growth factors are supplemented. *(iii)* the gene clusters shared with neuroendocrine and immune/gut tissues essentially reflect the secretory and protein biosynthetic specializations, respectively, and these make sense in the context of the beta cell. *(iv)* The pancreatic acinar cell is considered as the cell type with the most specialized gene expression in the human body [18], so even moderate contaminations can have strong impact. Indeed, in one reported list of human islet-specific genes [12] more than half of the top-10 genes belonged to exocrine cells. The FACS-enrichment of human beta cells to ±55% insulin positivity needs improvement but is currently state-of-the-art. But even with <1% exocrine contamination as in the rat model, some impact by exocrine contamination on resulting beta cell marker gene list cannot be fully excluded. Therefore we used the comparison of whole pancreatic tissue to isolated beta cells in the human data to eliminate pancreatic exocrine marker genes, mainly of the acinar cell. Moreover, cultured human beta cells contained much less exocrine contamination than freshly isolated islets, but also clearly less than laser microdissected human beta cells.

*Third*, our focus only on genes that are beta cell abundant in rat, mouse and human filters out experimental noise due to variability coming from differences in beta cell isolation techniques and age differences (standardized adolescent 10-week old rodents versus 47±10 adult human beta cells). An added advantage is that the evolutionary conservation from rodents to man can be seen as an indirect index for biological significance.

Our approach also has downsides. Not all relevant regulators in beta cell physiology are conserved: examples are NADP-dependent malic enzyme (*ME1*) that is important for glucose-stimulated insulin secretion in rat and human but not mouse [19], or cyclin D2, beta cell-abundant in rodents but not humans [20]. Also, expected conserved markers were not identified because of technical limitations of the array platforms: e.g. *SLC30A8* [16] or *FXYD2* [13] were not retrieved since probes for these genes were not present on the oligonucleotide arrays.

### Limits to the concept of beta cell specificity: many shared traits with other cell types

As first validation, beta cell marker genes were examined for their natural tissue tropism, by visualization in the independent Human Gene Atlas data set [3]; this confirmed the power of our *combined* marker gene list to discriminate the beta cell from any other assayed tissue type. Yet at the level of *individual* marker genes, both microarray and qPCR findings underline the notion that few if any genes can be confidently called absolutely beta cell-specific. Hydrolysis probes-based qPCR detects insulin 2 mRNA also in brain and intestine albeit at respectively 3 and 5 orders of magnitude lower than in pancreas. This is not novel since insulin expression was previously observed in brain, thymus, liver and bone marrow [21]. *ST18* mRNA proved one of the most beta cell-selective transcripts but was also qPCR-detected in brain, and was previously observed in the human breast ductal cells [22]. These low expression-signals could reflect *illegitimate* (*ectopic*) expression of cell type-selective genes [23]. It could alternatively indicate abundant expression only in one specific cell type within a tissue, e.g. in small populations of glucose-sensing neurons or endocrine cells in central nervous system [24], [25] and gut [26]–[28]. Latter beta cell-like cells express glucokinase and K^+^_ATP_-dependent membrane channels; gut K-cells can even be engineered to achieve regulated insulin secretion [29]. Direct comparison of such cell populations after their purification could disclose even higher similarity to beta cells, and further limit the notion of beta cell-specificity. We therefore prefer to use the term of cell type *selectivity* rather than *specificity*, and degree of selectivity likely varies depending of the number of corresponding mRNA or protein copies/cell.

Among the conserved beta cell marker genes only 15% show a near absolute beta cell-selectivity. This gene cluster contains known beta cell markers (e.g. *IAPP*, *INS1/2*, *PCSK1*, *INSM1*, *HADH*), as well as novel ones, such as *(i) ST18*, a nuclear protein with tumor suppressor activity [22]; (*ii*) Protein phosphatase 1, regulatory (inhibitor) subunit 1A (*PPP1R1A*), known as regulator of muscle activity [30] found to be more strongly expressed in beta cells where its target protein, protein phosphatase 1, regulates insulin synthesis. PPP1R1A was shown here to associate a very high molar abundance in beta cells – as judged by quantitative LC-MS proteome analysis - to a clear beta cell-selectivity in rat pancreas which calls for further exploration of its functional role in beta cells; (*iii*) *FOSB* gene products regulate neuronal plasticity [31] but are 20-times more abundant in beta cells than in brain and *(iv) WNT4*, which was recently linked to the repressed state of canonical Wnt signaling in beta cells [32].

At least another 15% of the conserved beta cell markers are clearly shared with neurons. Beta cells are known to express neuronal traits. They make synaptic-like vesicles containing glutamate decarboxylase and produce GABA [27], [33]–[36]. Beta cell development is guided by transcription factors that also control formation of dopaminergic neurons [35], [37]–[39] e.g. NeuroD1, Nkx6-1, Foxa2, neurogenins [40]. Also, beta cell progenitors can be epigenetically reprogrammed to a neuronal phenotype by polycomb target derepression via loss of trimethylated histone H3K27 [41], possibly by shutting down activity of neuron-restrictive silencer REST [33].The presently reported beta-neuron cluster represents, to our knowledge, the most comprehensive list of neuronal genes in beta cells. A third cluster of beta cell marker genes was functionally enriched in activities of protein folding and processing in the endoplasmic reticulum. These genes were also abundant in immune and gut mucosal cells. The similarity in gene expression between gut and immune cells may be related to the presence of gastrointestinal-associated lymphoid cells, which account for 10% of all gut cells [42]. This cluster does not contain white blood cell-selective clusters of differentiation nor other immune-selective transcripts, arguing against contamination as obvious cause for expression similarity.

### The conserved beta cell biomarker panel also marks beta cells obtained in situ by laser capture microdissection

Our conserved beta cell marker genes were selected on beta cell preparations that underwent isolation and in the human beta cells also a 2–3 week in vitro culture followed by FACS-enrichment. Since cell isolation can entail a combined mechanical and hypoxic cell stress, we verified expression level of conserved beta cell marker genes in human beta cells obtained by laser capture microdissection, which presumably closely approximates in vivo expression. We cannot rule out some bias of cell isolation since we found higher mRNA levels of stress response transcription factor ATF3 [43] in isolated as compared to laser capture-beta cells; this likely reflects active ATF3 since we also found statistical enrichment of its binding sites in the conserved beta cell marker genes. Yet, overall we found that the conserved beta cell marker gene panel equally discerns laser capture and isolated beta cells from all other tissues (Fig. S2) and that the expression levels of established beta cell marker genes were equal or higher in the isolated, cultured human beta cells. While cultured beta cells indeed show statistical enrichment of genes involved in heat shock and protein folding, they also showed concerted activation of gene clusters with role in mRNA processing, translation factor activity and protein processing for secretion, reflecting their higher activity of regulated protein synthesis, which represents the beta cell's core function.

### The beta cell biomarker panel is a practical tool to monitor phenotypic adaptations of the beta cell

The beta cell acutely and chronically adapt to nutrient stimulations [8], [44]. We used the conserved beta cell marker gene panel as practical tool to see how 24 h fasting impacts on beta cell differentiated state. While genome-wide comparison showed that fasting suppresses <2% of coding genes, it affected more than 10% of the beta cell marker genes. Interestingly, no changes occurred in the cluster of beta cell selective genes nor in that of beta-neuron genes indicating that short-term fasting preserved the neuroendocrine differentiation. On the other hand, suppression was clear in the cluster of genes with a role in protein processing and synthesis, and which thus appear induced by feeding.

### From conserved mRNA expression to conserved transcriptional regulators

Finally we applied the conserved beta cell marker genes to partly map the transcriptional network that maintains the specialized state of nutrient-sensing beta cells. Two entirely different but complementary searches for conserved transcription factor consensus motifs showed a substantial overlap, and clearly highlighted members of the activating transcription factor Atf/Creb family. Atf2, Atf3 and Atf4 are known regulators of beta cell function [43], [45] and their mRNA appears also abundantly expressed in these cells. Overrepresentation of binding sites for several other nutrient-regulated transcription factors (Arnt/Hif, upstream factors, Oct1, Nfat and Stra13) also fit with their previously described roles in beta cellular glucose sensing. The enrichment of Tal1 and Meis1 binding sites fits with the reported similarities between beta and immune cells since these transcription factors were mainly studied as regulator of white blood cell function. Equally surprising is the enrichment of binding sites for factors involved in muscle/heart formation (Myogenin, Myod1, Rfx1, Mrf2). Perhaps the latter is explained by the abundance of contractile intermediary filaments in beta cells as part of their secretory vesicle trafficking machinery.

In conclusion, the proposed set of conserved beta cell marker genes is a practical tool to interpret holistic changes in gene expression of rodent and human beta cells. It reveals novel beta cell selective transcripts with potential as physiological regulator. The gene set also underlines the notion that few if any genes are absolutely beta cells specific, and highlights functional similarities of the beta cells with neuronal and immune/gut cells.

## Materials and Methods

### Cell and tissue preparations

Human pancreas donor organs were procured by European hospitals affiliated with the Eurotransplant Foundation (Leiden, the Netherlands) and processed by the Beta-Cell Bank of the Center for Beta-Cell Therapy in Diabetes. Use of human cells derived from donor organs was approved by the Medical ethical committee of the Universitair Ziekenhuis Brussel (UZB), according to Belgian laws.

Rodents were housed according to the Belgian animal welfare regulations. Animal killing was kept to the strict minimum, after proper CO_2_-anesthesia. Use of animal cells and tissues was approved by the Commissie Proefdiergebruik (CPG) of the Vrije Universiteit Brussel (VUB), for a project entitled “in vitro and in vivo markers for beta cell death and function” (CPG approval ID 07-274-3).

Rat beta (92±3% insulin+) and alpha (68±9% glucagon+ with <10% insulin+) cells were FACS-purified from 10-weeks old Wistar rats as described [46]. Other rat tissues (total brain, pituitary, *M. Soleus* skeletal muscle, white adipose tissue and liver for microarray; for qPCR extended panel as shown in Table 1) were snap frozen and stored in liquid nitrogen. Mouse islets were in house isolated from C57/BL6 mice (CERJ, France); mouse tissue CEL files were obtained from Gene Expression Omnibus GSE1986. Human beta cells (55±13% insulin+ with 13±8% glucagon+ cells and 21±7% non-granulated cells) and pancreatic duct cells (85±7% CK+ with 4±1% insulin+ and 6±4% glucagon+) were FACS-sorted from 2–3 w cultured fractions [47]. We hybridized 3 different pools of 6 µg RNA obtained from 10 human organs in total: pool 1 (45% insulin+, from n = 3 donor organs), pool 2 (65% ins+, from n = 3 donor organs) and pool 3 (55% insulin+, from n = 4 donor organs). Average donor age and BMI were 47±10 yrs and 26±4, respectively. RNA pools from 13 other human tissues were provided by Finn C. Nielsen (Rigshospitalet, Copenhagen, Denmark).

### Gene chip hybridization

Total RNA was isolated using TRIzol Reagent (Invitrogen, Carlsbad, CA, USA.) and quality-controlled by 2100 Bioanalyzer (Agilent, Santa Clara, CA, USA), taking RIN>8. One µg RNA was used to synthesize double-stranded cDNA with the Superscript Choice system (Invitrogen Corporation, Carlsbad, CA, USA) using an oligo(dT) primer containing a T7 RNA polymerase promoter (GenSet, Paris, France) and cDNA was in vitro transcribed to synthesize biotin-labeled antisense cRNA (BioArray high yield RNA transcript labelling kit; Enzo Diagnostics, Farmingdale, NY, USA). After fragmentation at 94°C for 35 min in 40 mM Tris, 30 mM magnesium acetate, 10 mM potassium acetate, labeled cRNA was hybridized for 16 h on Affymetrix (Santa Clara, CA, USA) expression arrays. Arrays were washed and stained with phycoerythrin-streptavidin (SAPE) in Affymetrix Fluidics Station 400 and scanned in the Affymetrix GeneArray 2500 scanner. For human beta and duct cells, 3 respectively 2 independent isolates, each obtained from 3–4 non-selected donor pancreases, were hybridized on Affymetrix HG133A. Three independent isolates of rat tissues or cells were hybridized on Affymetrix RG230A. Three independent isolates of mouse islets were compared to samples from Gene Expression Omnibus GSE1986 series. CEL files of freshly isolated human islets (n = 3) and laser capture beta cells (n = 3) were obtained from reference [5], and normalized in one group with our human beta cell CEL files to array with median intensity. Raw data are publicly available through Gene Expression Omnibus (human data GSE30803).

*Gene chip data analysis* Scanned arrays were analyzed with dChip model-based expression analysis [48] after normalization to array with median intensity (default) and using background correction for mismatch hybridization. Excel software was used to calculate the relative transcript expression in beta cell preparations versus the other tissues, a ratio further referred to as ‘fold change’. Within each species, the average of all these ratios calculated for all assayed tissues is called ‘*average fold change*’ (avFC). We also calculated a ‘*beta cell rank*’ to reflect beta cell selectivity: rank 1, 2, 3,…denotes respectively that the transcript showed the highest, 2^nd^ highest, 3^rd^ highest expression in beta cells as compared to all other tissues. Selection of conserved beta cell marker genes is described in Results section. The final set of beta cell marker genes was independently validated in the Human Gene Atlas [3], a freely available data set (Gene Expression Omnibus series GSE1133) comparing duplicate hybridizations of RNA pools from 79 human primary tissues and cell types, and further supplemented by our beta and duct cell samples and previously reported CEL files of human islets [49]. dChip was also used for hierarchical clustering (correlation/centroid mode by cluster tightness) and gene ontology enrichment. Gene markers are referred to by their official gene symbol, in capitalized italic font.

### Bioinformatics enrichment analysis of cis-regulatory motifs in the promoters of beta cell marker genes

We used the whole collection of TRANSFAC matrices (442 matrices for human, 392 for mouse and 243 for rat). Using retrieve-ensembl-seq [50], promoter sequences were retrieved over 2,000 bp upstream the transcription start site, masking the coding sequences of upstream genes; in a separate file the first intron of each gene (median length = 3,845 bp) was collected. Repeats were masked in both promoter and intron sequences. *Site enrichment analysis*. Enrichment analysis was led with the program matrix-scan [7], which automatically estimates the enrichment of sites for a given motif in a given input sequence for all possible matrix weight score thresholds. The weight score indicates the similarity between a sequence and the motif, as defined as by Hertz and Stormo [51]. For each motif, matrix-scan computes the number of hits in the input sequences above each possible weight score threshold, and compares it with the number of hits expected by chance, according to the background model. The level of the over-representation is estimated with the binomial P-value [52], [53], which is converted to a significance score defined as sig = −log10 (P val). The program returns the optimal matrix score, i.e. the weight score associated to the minimal P-value. The background model was estimated from the input sequences, as a Markov model of order 1. The first-order model enables to account for dependencies between adjacent residues, which is especially important to model the general rarity of the CpG dinucleotide in vertebrate promoters. *Negative control* As negative control, we permuted randomly the columns of the TRANSFAC matrices, and applied the same procedure as with the original matrices. The main interest of that type of control is that it preserves the residue composition and the information content while removing the actual motif. As complementary approach, DiRE (http://dire.dcode.org/) was used using default settings [6].

### Validation of relative beta cell abundant mRNAs by quantitative real-time PCR using hydrolysis probes

Hydrolysis probes were obtained from Applied Biosystemes (TaqMan MGB, assays' IDs available on request) using 4 reference genes (beta-actin, *BACT;* cyclophilin A/peptidyl prolyl isomerase A, *PPIA*, ubiquitin C, *UBC* and proteasome 26S subunit, ATPase 5, *PSMC5*) for normalization along the GEOnorm principle [4].

### Validation of beta cell-selective protein expression by quantitative label-free LC-MS and immunohistochemistry

The proteomes of unfractionated rat liver, brain and FACS-purified islet alpha and beta cells were previously quantified using label-free alternate-scanning LC-MS [9]. Selected markers were in situ confirmed 10w-old male Wistar rat and/or human pancreas. Primary antibodies were rabbit anti-human PPP1R1A (1∶1000), chicken anti-human PTPRN (1∶500) and chicken anti-human HADH (1∶1000) all from Genway (San Diego, CA, USA. PPP1R1A staining worked best on paraffin sections, IA2 and HADH staining best on tissue fragments fixed for 30 min in 4% formaldehyde followed by overnight incubation in 30% sucrose prior to snap freezing in liquid nitrogen and cryosectioning. Non-specific staining was blocked with 10% donkey serum in PBS, followed by overnight incubation at 4°C with primary antibodies, 60 min with secondary antibodies and addition of bisbenzimide (Hoechst 33342, Sigma). Secondary antibodies (dylight 488 for markers, dylight 649 for insulin and glucagon) were from Jackson Immunoresearch Laboratories (West Grove, PA, USA).

### Statistical analysis

Data are presented as arithmetic or geometric means ± SD or SE of *n* independent experiments; statistics used by dChip, DiRE) and Matrix Scan outlined above or in original publications.

## Supporting Information

Figure S1 **View on exocrine contamination and its removal from our beta cell marker gene list.** Fig. S1A shows a hierarchical clustering of 526 probe sets corresponding to human genes with species-conserved relative beta cell abundant expression, including 17 exocrine contaminants (cluster marked in blue) that uniquely expressed in pancreatic samples with strongest expression in total pancreas>islets>FACS-sorted beta cells. Bar graphs in Fig. S1B highlight the individual genes in this cluster; most correspond to digestive enzymes, and are markers for exocrine acinar cells. Note that cultured, FACS-purified human beta cell preparations used in our study show minimal contamination with exocrine markers as compared to freshly isolated whole human islets. Expression signal in total pancreas and whole islets are comparable; note that this probably represents an artifact of the oligonucleotide array technique: due to probe saturation by highly abundant mRNAs, expression signal is no longer linearly related to actual mRNA concentration.
(TIF)

Figure S2 **Beta cell marker genes discriminate beta cells from any other cell type in the body.** Fig. S2 shows a 2-dimensional principle component analysis (PCA) of the 332 species-conserved mRNAs (n = 503 probe sets) with relative beta cell abundant expression in the tissue mRNA profiles of the human GSE1133 data set, containing duplicate hybridizations of RNA pools obtained from human tissues or cells [3]. This data set was supplemented by Affymetrix hybridizations of FACS-enriched cultured human beta cells (obtained from Beta Cell Bank, Brussels, Belgium, data point 1, n = 3 hybridizations on cells from 10 donors) and freshly isolated human islets (data point 2) and laser capture microdissected human beta cells (data point 3) – the latter two preps representing n = 3 hybridizations each from one donor, and previously published by Marselli L. et al. [5] from Joslin Diabetes Center, Harvard, USA. The PCA illustrates that the conserved beta marker genes can discriminate beta cell from all other tissues in a data set independent from the one used to compile it; it also shows that the biomarkers can equally discriminate isolated and laser capture beta cells. X- and Y-axes of the PCA represent respectively the major and minor components of variation.
(TIF)

Figure S3 **View on brain region-tropism of shared neuron-beta cell marker genes.** Bar graphs show relative mRNA expression signal (human HG133A array), in the human beta cells as compared to the indicated brain region, of 15 beta cell marker genes that are also abundantly expressed in the human brain (mean of 2–3 hybridizations). Left panel shows these data for 15 shared beta cell-neuron marker genes, while right panel shows the median ratio for a given brain region. Selected beta cell marker genes reach highest levels in globus pallidus and nucleus subthalamicus.
(TIF)

Figure S4 **Focus on gene cluster with shared relative abundant expression in gut, hematological and beta cells as evidenced by hierarchical cluster graph from** **Figure 1** **.** Associated table shows gene symbols and names, accession number and for each species the associated avFC and rank scores. Table on left panel shows functional pathways that are statistically overrepresented in this cluster, using dChip gene ontology enrichment (p<0.05).
(TIF)

Table S1 **332 genes with species-conserved relative abundant expression in beta cells.** Table S1 shows 419, 499 and 503 probe sets on respectively rat (RG230A, blue), mouse (MG430.20, green) and human (HG133A, red) Affymetrix microarrays. These correspond to 332 genes, since Affymetrix chips associate multiple probe sets to the same genes. In 2^nd^ column the genes belonging to cluster A (neuroendocrine), B (beta cell-selective) or C (gut/immune) of Fig. 2 are indicated. Table also shows for each species probe set IDs, Accession/Locuslink ID, average fold change and beta score ranging from minus to plus 6 in rat, 14 in human and 17 in mouse. Note: for user-friendly display and to allow direct comparison of between homologous probe sets of different species, some probe sets are shown more than once when number of probe sets per gene differs between species, so that all rows contain values and can easily be sorted.
(PDF)

Table S2 **Statistically overrepresented functional gene ontologies in cultured versus laser capture microdissected human beta cells.** 2736 probe sets were ≥1.5 fold (LCB) up-regulated (p<0.05, n = 3) in cultured FACS-enriched versus laser capture microdissected (LCM) human beta cells. Table S2 shows the functional gene ontologies that are statistically (p<0.001) overrepresented in the cultured (blue) versus LCM (green) beta cells. For comparison corresponding mRNA expression signals in freshly isolated islets (red) and statistics versus LCM beta cells are also shown. Cultured FACS-enriched beta cells show a concerted up-regulation of the transcriptional-protein synthetic pathway, starting from mRNA splicing and processing, translational initiation and ribosomal RNA to protein folding in endoplasmic reticulum and subsequent processing through Golgi apparatus to the secretory vesicle.
(PDF)

Table S3 **DiRE analysis of transcriptional regulators of the beta cell phenotype.** DiRE was used to retrieve transcription factor binding sites (TFBs) that are statistically overrepresented in beta cell marker genes versus random reference gene sets (±5000 genes) of the human (hg18), mouse (mm9) and rat (rn4) genomes. Score indicate statistical importance (ranging from 0 to 1). For each species, the analysis was run on the whole set of beta cell markers (all), and also separately on the 3 outstanding clusters of neuro-endocrine genes (cluster A, see Fig. 3), beta cell-selective genes (cluster B, see Fig. 2) and markers shared with immune and gut cells (cluster C, see suppl. Fig. 3) genes. Data (human analysis) are graphically presented in Fig. 6.
(PDF)

Table S4 **Transcription factor consensus sites identified by 2 independent algorithms.** DNA consensus sites (TRANSFAC) with potential for transcription factor binding were identified by DiRE and Matrix Scan; 27 consensus sites were identified by both algorithms (ochre), 27 only by the more stringent Matrix Scan (blue), and 77 only by DiRE (yellow). Marked in bold are transcription factors with established role in beta cell development and physiology Data are graphically presented in Fig. 6.
(PDF)
