# Correction: Clusters of Conserved Beta Cell Marker Genes for Assessment of Beta Cell Phenotype

**DOI:** 10.1371/annotation/a91571a6-acbb-456f-bcc5-f4a431e28516

**Published:** 2012-01-24

**Authors:** Geert A. Martens, Lei Jiang, Karine H. Hellemans, Geert Stangé, Harry Heimberg, Finn C. Nielsen, Olivier Sand, Jacques Van Helden, Frans K. Gorus, Daniel G. Pipeleers

A reference was inadvertently excluded. The reference should be numbered 31 and is: Vander Mierde D, Scheuner D, Quintens R, Patel R, Song B, Tsukamoto K, Beullens M, Kaufman RJ, Bollen M, Schuit FC (2007) Glucose activates a protein phosphatase-1-mediated signaling pathway to enhance overall translation in pancreatic beta-cells. Endocrinology 148: 609-617.

The references that follow in the list, 31-53, should be increased by one, to 32-54.

The citation for this reference belongs in the following sentence in the "Limits to the concept of beta cell specificity: many shared traits with other cell types" section in the Discussion: "Ppp1r1a, also known as Inhibitor1 (I-1) protein was previously found to be abundantly expressed in mouse MIN6 beta cell line and mouse primary islets, where it forms a complex with translation factor eIF-alpha and protein phosphatase-1 [31]."

