# Correction: Clusters of Conserved Beta Cell Marker Genes for Assessment of Beta Cell Phenotype

**DOI:** 10.1371/annotation/4aae21a9-e176-4feb-9f15-103b265d3335

**Published:** 2012-01-24

**Authors:** Geert A. Martens, Lei Jiang, Karine H. Hellemans, Geert Stangé, Harry Heimberg, Finn C. Nielsen, Olivier Sand, Jacques Van Helden, Frans K. Gorus, Daniel G. Pipeleers

There is an error in the second paragraph in the "Limits to the concept of beta cell specificity: many shared traits with other cell types" section in the Discussion. The correct text is: "Among the conserved beta cell marker genes only 15% show a near absolute beta cell-selectivity. This gene cluster contains known beta cell markers (e.g. IAPP, INS1/2, PCSK1, INSM1, HADH), as well as novel ones, such as (i) ST18, a nuclear protein with tumor suppressor activity [22]; (ii) Protein phosphatase 1, regulatory (inhibitor) subunit 1A (PPP1R1A), was previously studied as regulator of muscle activity [30]. Ppp1r1a, also known as Inhibitor1 (I-1) protein was previously found to be abundantly expressed in mouse MIN6 beta cell line and mouse primary islets, where it forms a complex with translation factor eIF-alpha and protein phosphatase-1 [Reference added post-publication, Vander Mierde et al]. Our LC-MS analysis of purified beta and alpha cells, and in situ immunohistochemical analysis now further substantiate its specific and abundant expression in the beta cell lineage, and calls fur further exploration of how its association to protein phosphatase 1 mediates the latter proven action in glucose-regulation of insulin synthesis [Reference added post-publication, Vander Mierde et al]; (iii) FOSB gene products regulate neuronal plasticity [31] but are 20-times more abundant in beta cells than in brain and (iv) WNT4 , which was recently linked to the repressed state of canonical Wnt signaling in beta cells [32]."

