# Correction: Clusters of Conserved Beta Cell Marker Genes for Assessment of Beta Cell Phenotype

**DOI:** 10.1371/annotation/7aa0ff33-5660-4b56-889a-4b86a273d522

**Published:** 2012-01-24

**Authors:** Geert A. Martens, Lei Jiang, Karine H. Hellemans, Geert Stangé, Harry Heimberg, Finn C. Nielsen, Olivier Sand, Jacques Van Helden, Frans K. Gorus, Daniel G. Pipeleers

There are errors in the "Gene chip hybridization" paragraph in Materials and Methods. The correct text is: "Total RNA was isolated using TRIzol Reagent (Invitrogen, Carlsbad, CA, USA.) followed by a cleanup step with qiagen RNeasy mini columns (Qiagen, Cologne, Germany) and quality-controlled by 2100 Bioanalyzer (Agilent, Santa Clara, CA, USA). The expression profile of rat tissues and cells was analysed using rat 230A-arrays from Affymetrix (Santa Clara, CA, USA) according to the Genechip expression analysis technical manual 701025 Rev.2. Briefly, 5 μg RNA was used to synthesize double-stranded cDNA with the Superscript Choice system (Invitrogen Corporation, Carlsbad, CA, USA ) using an oligo(dT) primer containing a T7 RNA polymerase promoter (Affymetrix,GenSet, Paris, France) and cDNA was in vitro transcribed to synthesize biotin-labeled antisense cRNA (BioArray high yield RNA transcript labelling kit; Enzo Diagnostics, Farmingdale, NY, USA). After fragmentation at 94°C for 35 min in 40 mM Tris, 30 mM magnesium acetate, 10 mM potassium acetate, labeled cRNA was hybridized for 16 h on Affymetrix (Santa Clara, CA, USA) expression arrays. Arrays were washed and stained with phycoerythrin-streptavidin (SAPE) in Affymetrix Fluidics Station 400 and scanned by Affymetrix 3000 GeneScanner."

