# Correction: Clusters of Conserved Beta Cell Marker Genes for Assessment of Beta Cell Phenotype

**DOI:** 10.1371/annotation/13c6d084-a8fd-4019-a3cf-12a0d8abe309

**Published:** 2012-01-24

**Authors:** Geert A. Martens, Lei Jiang, Karine H. Hellemans, Geert Stangé, Harry Heimberg, Finn C. Nielsen, Olivier Sand, Jacques Van Helden, Leentje Van Lommel, Frans Schuit, Frans K. Gorus, Daniel G. Pipeleers

There is an error in the author by-line. Two authors, Leentje Van Lommel and Frans Schuit, were inadvertently omitted. The correct author by-line is: Geert A. Martens, Lei Jiang, Karine H. Hellemans, Geert Stangé, Harry Heimberg, Finn C. Nielsen, Olivier Sand, Jacques Van Helden, Leentje Van Lommel, Frans Schuit, Frans K. Gorus, and Daniel G. Pipeleers

Both Leentje Van Lommel and Frans Schuit are affiliated with Gene Expression Unit, Katholieke Universiteit Leuven (KUL).

Frans Schuit's contributions were: Conceived and designed the experiments, Performed the experiments, Analyzed the data, Contributed reagents/materials/analysis tools.

Leentje Van Lommel's contributions were: Performed the experiments.

