# Supplementary figures and images for: Clusters of Conserved Beta Cell Marker Genes for Assessment of Beta Cell Phenotype
Source: PLoS One. 2011 Sep 2;6(9):e24134. doi: 10.1371/journal.pone.0024134 (PMC3166300; doi:10.1371/journal.pone.0024134)

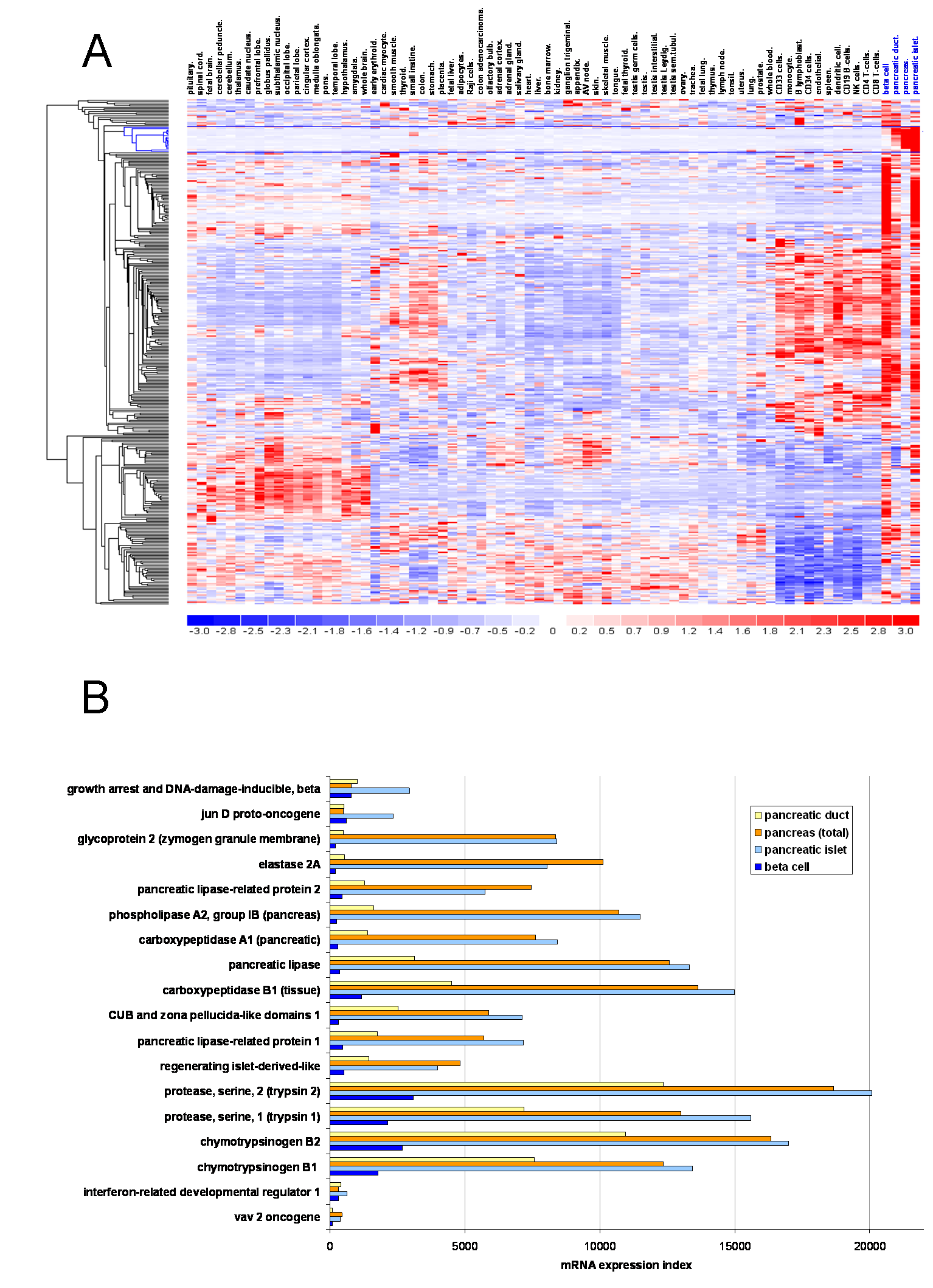

Supplement: Figure S1 — View on exocrine contamination and its removal from our beta cell marker gene list. Fig. S1A shows a hierarchical clustering of 526 probe sets corresponding to human genes with species-conserved relative beta cell abundant expression, including 17 exocrine contaminants (cluster marked in blue) that uniquely expressed in pancreatic samples with strongest expression in total pancreas>islets>FACS-sorted beta cells. Bar graphs in Fig. S1B highlight the individual genes in this cluster; most correspond to digestive enzymes, and are markers for exocrine acinar cells. Note that cultured, FACS-purified human beta cell preparations used in our study show minimal contamination with exocrine markers as compared to freshly isolated whole human islets. Expression signal in total pancreas and whole islets are comparable; note that this probably represents an artifact of the oligonucleotide array technique: due to probe saturation by highly abundant mRNAs, expression signal is no longer linearly related to actual mRNA concentration. (TIF) [file pone.0024134.s001.tif]

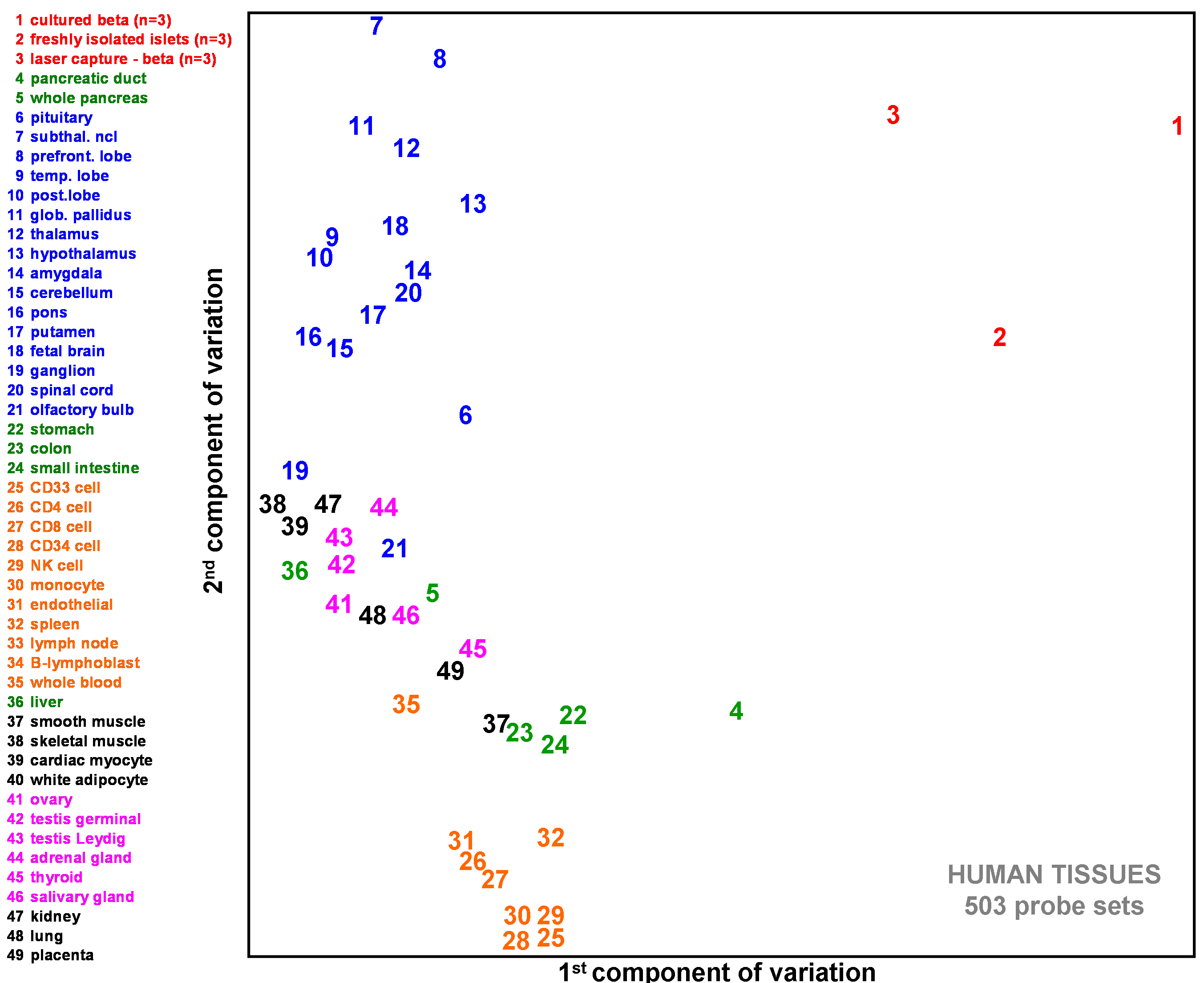

Supplement: Figure S2 — Beta cell marker genes discriminate beta cells from any other cell type in the body. Fig. S2 shows a 2-dimensional principle component analysis (PCA) of the 332 species-conserved mRNAs (n = 503 probe sets) with relative beta cell abundant expression in the tissue mRNA profiles of the human GSE1133 data set, containing duplicate hybridizations of RNA pools obtained from human tissues or cells [3]. This data set was supplemented by Affymetrix hybridizations of FACS-enriched cultured human beta cells (obtained from Beta Cell Bank, Brussels, Belgium, data point 1, n = 3 hybridizations on cells from 10 donors) and freshly isolated human islets (data point 2) and laser capture microdissected human beta cells (data point 3) – the latter two preps representing n = 3 hybridizations each from one donor, and previously published by Marselli L. et al. [5] from Joslin Diabetes Center, Harvard, USA. The PCA illustrates that the conserved beta marker genes can discriminate beta cell from all other tissues in a data set independent from the one used to compile it; it also shows that the biomarkers can equally discriminate isolated and laser capture beta cells. X- and Y-axes of the PCA represent respectively the major and minor components of variation. (TIF) [file pone.0024134.s002.tif]

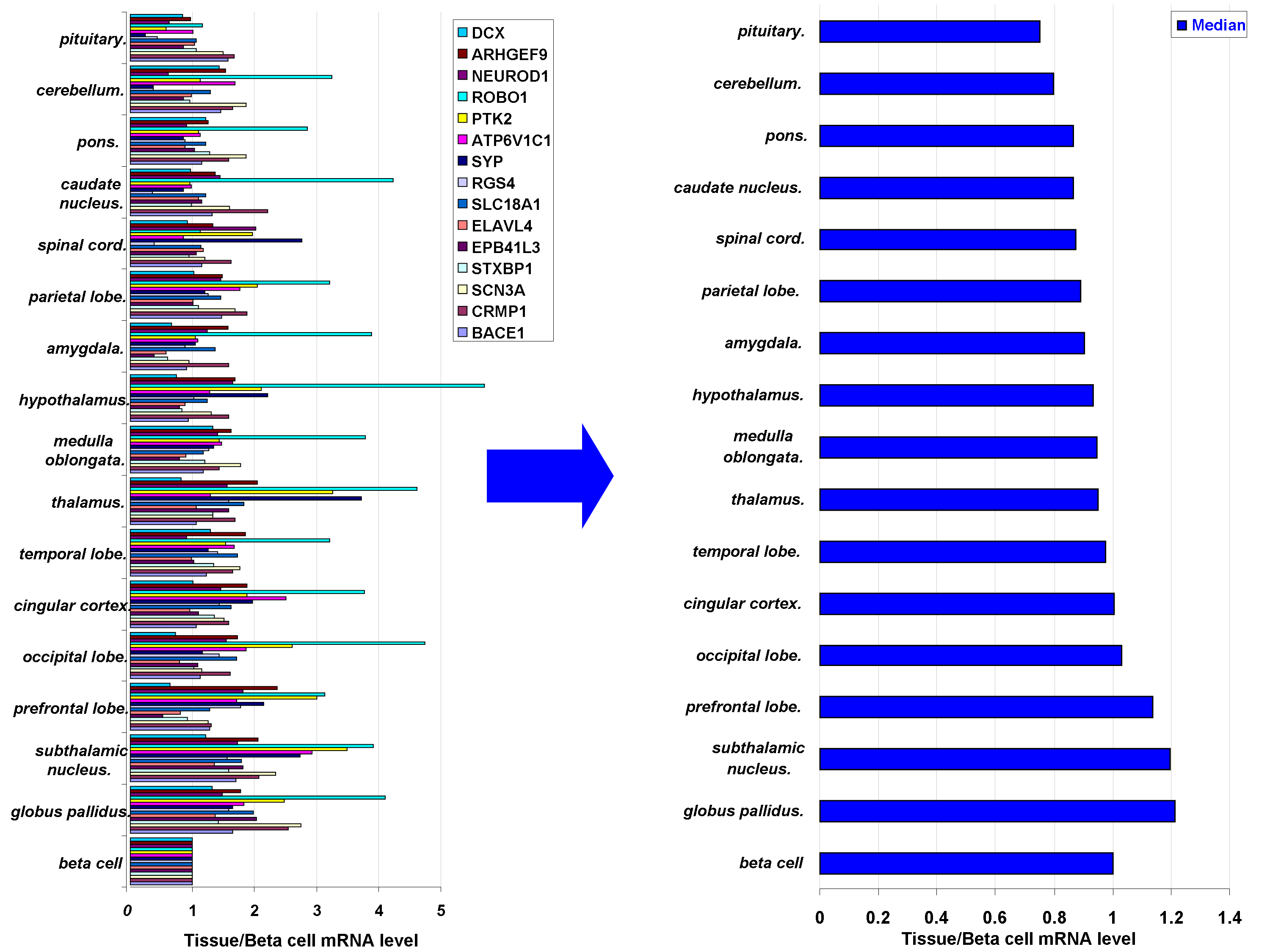

Supplement: Figure S3 — View on brain region-tropism of shared neuron-beta cell marker genes. Bar graphs show relative mRNA expression signal (human HG133A array), in the human beta cells as compared to the indicated brain region, of 15 beta cell marker genes that are also abundantly expressed in the human brain (mean of 2–3 hybridizations). Left panel shows these data for 15 shared beta cell-neuron marker genes, while right panel shows the median ratio for a given brain region. Selected beta cell marker genes reach highest levels in globus pallidus and nucleus subthalamicus. (TIF) [file pone.0024134.s003.tif]

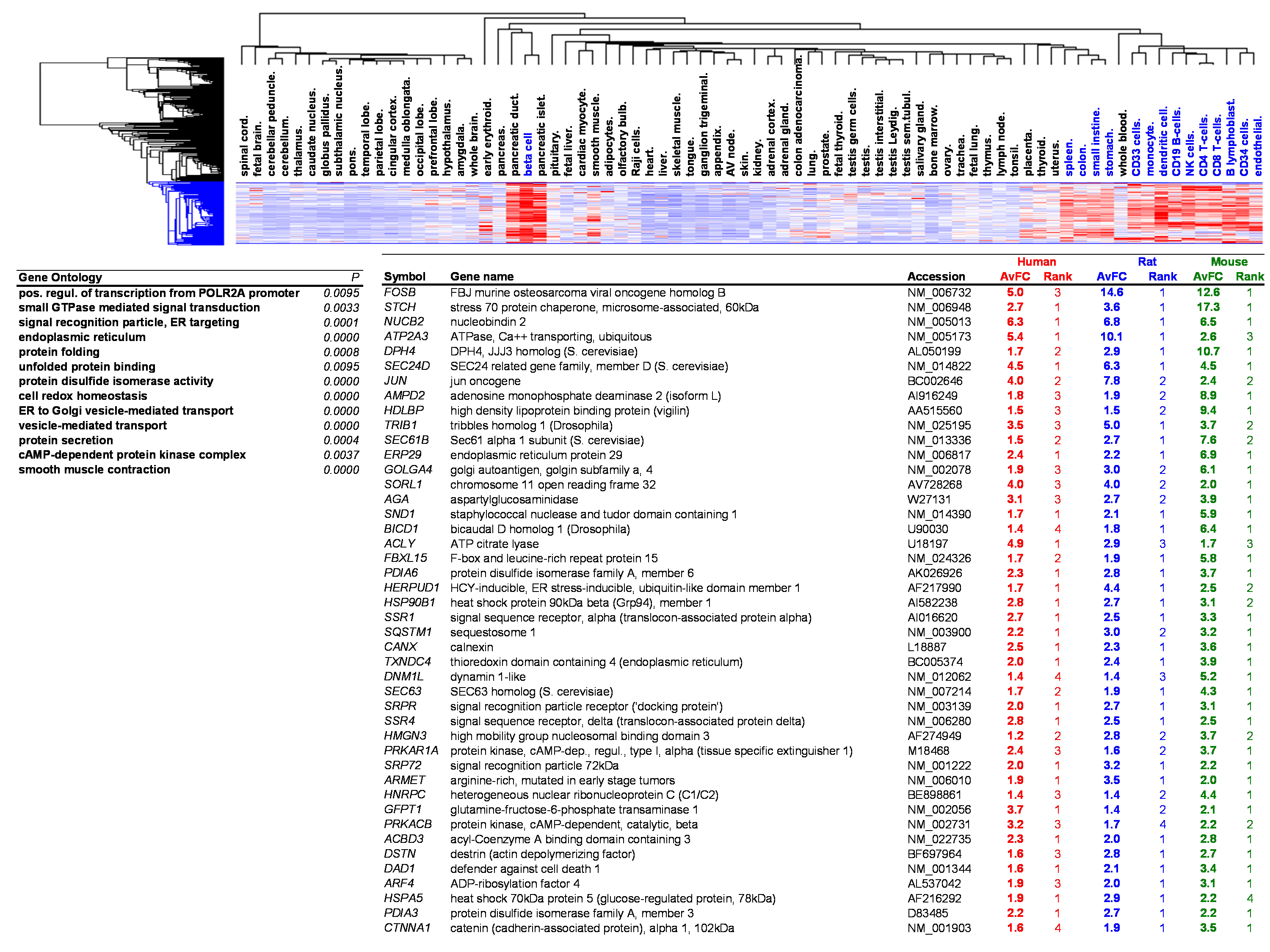

Supplement: Figure S4 — Focus on gene cluster with shared relative abundant expression in gut, hematological and beta cells as evidenced by hierarchical cluster graph from Figure 1 . Associated table shows gene symbols and names, accession number and for each species the associated avFC and rank scores. Table on left panel shows functional pathways that are statistically overrepresented in this cluster, using dChip gene ontology enrichment (p<0.05). (TIF) [file pone.0024134.s004.tif]
