# Supplementary material for: Clusters of Conserved Beta Cell Marker Genes for Assessment of Beta Cell Phenotype
Source: PLoS One. 2011 Sep 2;6(9):e24134. doi: 10.1371/journal.pone.0024134 (PMC3166300; doi:10.1371/journal.pone.0024134)
Supplement: Table S1 — 332 genes with species-conserved relative abundant expression in beta cells. Table S1 shows 419, 499 and 503 probe sets on respectively rat (RG230A, blue), mouse (MG430.20, green) and human (HG133A, red) Affymetrix microarrays. These correspond to 332 genes, since Affymetrix chips associate multiple probe sets to the same genes. In 2nd column the genes belonging to cluster A (neuroendocrine), B (beta cell-selective) or C (gut/immune) of Fig. 2 are indicated. Table also shows for each species probe set IDs, Accession/Locuslink ID, average fold change and beta score ranging from minus to plus 6 in rat, 14 in human and 17 in mouse. Note: for user-friendly display and to allow direct comparison of between homologous probe sets of different species, some probe sets are shown more than once when number of probe sets per gene differs between species, so that all rows contain values and can easily be sorted. (PDF) [file pone.0024134.s005.pdf]

Tissue-conserved, beta cell-abundant transcripts

symbol	cluster Fig.2	probe set	gene (Rat)	Accession	av FC	Beta score	probe set	Accession	av FC	Beta score	probe set	Accession	av FC	Beta score
**AACS**	B	1368126_at	acetoacetyl-CoA synthetase	NM_023104	2.1	4	218434_s_at	NM_023928	1.2	10	1451116_at	BC026817	4.837	17
**ACBD3**	C	1372552_at	acyl-Coenzyme A binding domain containing 3	AI102273	2.05	6	202324_s_at	NM_022735	2.28	14	1456316_a_at	BI965035	2.798	17
**ACBD3**	C	1372552_at	acyl-Coenzyme A binding domain containing 3	AI102273	2.05	6	202323_s_at	AI636775	1.7	14	1452137_at	BB704602	2.283	13
**ACBD3**	C	1372552_at	acyl-Coenzyme A binding domain containing 3	AI102273	2.05	6	202323_s_at	AI636775	1.7	14	1426635_at	BB704602	1.803	13
**ACBD3**	C	1372552_at	acyl-Coenzyme A binding domain containing 3	AI102273	2.05	6	202323_s_at	AI636775	1.7	14	1428668_at	BI790311	1.591	15
**ACLY**	C	1367854_at	ATP citrate lyase	NM_016987	2.91	2	201127_s_at	AI971281	2.43	14	1446315_at	BB261367	6.284	17
**ACLY**	C	1367854_at	ATP citrate lyase	NM_016987	2.91	2	201128_s_at	NM_001096	3.79	14	1438389_x_at	BB424434	2.938	17
**ACLY**	C	1367854_at	ATP citrate lyase	NM_016987	2.91	2	210337_s_at	U18197	4.94	14	1439445_x_at	AV347837	1.746	13
**ACLY**	C	1367854_at	ATP citrate lyase	NM_016987	2.91	2	201128_s_at	NM_001096	3.79	14	1451666_at	BI456232	2.52	17
**ACLY**	C	1367854_at	ATP citrate lyase	NM_016987	2.91	2	201128_s_at	NM_001096	3.79	14	1439459_x_at	BB276877	1.902	17
**ADRA2A**		1387708_at	adrenergic receptor, alpha 2a	U79031	2.64	6	209869_at	AF284095	1.22	8	1423022_at	NM_007417	2.889	17
**AGA**	C	1372421_at	aspartylglucosaminidase (predicted)	AA943766	2.72	6	204332_s_at	M64073	1.53	12	1434665_at	AV052058	3.899	17
**AGA**	C	1372421_at	aspartylglucosaminidase (predicted)	AA943766	2.72	6	204333_s_at	NM_000027	1.67	14	1434665_at	AV052058	3.899	17
**AGA**	C	1372421_at	aspartylglucosaminidase (predicted)	AA943766	2.72	6	216064_s_at	W27131	3.12	14	1434665_at	AV052058	3.899	17
**AMPD2**	C	1372782_a_a	similar to adenosine monophosphate deaminase 2 (isoform L)	BE111787	1.88	4	212360_at	AI916249	1.76	10	1438128_at	BB427907	8.87	17
**AMPD2**	C	1372782_a_a	similar to adenosine monophosphate deaminase 2 (isoform L)	BE111787	1.88	4	212360_at	AI916249	1.76	10	1438941_x_at	AV330806	2.884	15
**AMPD2**	C	1372782_a_a	similar to adenosine monophosphate deaminase 2 (isoform L)	BE111787	1.88	4	212360_at	AI916249	1.76	10	1426757_at	AK004759	2.263	17
**AP3B2**	A	1376844_at	adaptor-related protein complex 3, beta 2 subunit (predicted)	BI298352	1.44	2	205678_at	NM_004644	1.2	8	1451837_at	BC024722	2.044	17
**AP3B2**	A	1376844_at	adaptor-related protein complex 3, beta 2 subunit (predicted)	BI298352	1.44	2	205678_at	NM_004644	1.2	8	1426927_at	AB030202	1.991	15
**AP3B2**	A	1376844_at	adaptor-related protein complex 3, beta 2 subunit (predicted)	BI298352	1.44	2	205678_at	NM_004644	1.2	8	1445224_at	BI134892	1.858	13
**AP3D1**		1375444_at	adaptor-related protein complex 3, delta subunit (predicted)	BF409829	1.6	6	210974_s_at	AF130042	1.21	10	1417964_at	NM_007460	1.579	15
**AP3D1**		1367549_a_a	adaptor-related protein complex 3, delta subunit (predicted)	BI282757	1.37	0	208710_s_at	AI424923	1.2	8	1417964_at	NM_007460	1.579	15
**APLP1**	A	1371572_at	amyloid beta (A4) precursor protein	BM986220	1.35	-2	209462_at	U48437	6.71	8	1435857_s_at	AI848048	3.641	15
**APLP1**	A	1371571_at	amyloid beta (A4) precursor protein	BM986220	1.4	0	209462_at	U48437	6.71	8	1416134_at	NM_007467	1.909	15
**ARCN1**	C	1398896_at	archain	AA892567	1.61	6	201176_s_at	NM_001655	1.75	14	1423743_at	BC017124	2.415	15
**ARCN1**	C	1398896_at	archain	AA892567	1.61	6	201176_s_at	NM_001655	1.75	14	1451089_a_at	BC017124	1.806	17
**ARCN1**	C	1398896_at	archain	AA892567	1.61	6	201176_s_at	NM_001655	1.75	14	1436062_at	BB757123	1.763	15
**ARF4**	C	1398758_at	ADP-ribosylation factor 4	NM_024151	2.02	6	201096_s_at	AL537042	1.94	10	1434074_x_at	BB754834	3.116	17
**ARF4**	C	1398758_at	ADP-ribosylation factor 4	NM_024151	2.02	6	201097_s_at	NM_001660	1.69	12	1434074_x_at	BB754834	3.116	17
**ARFIP1**	C	1369026_at	ADP-ribosylation factor interacting protein 1	NM_021763	2.2	6	218230_at	AL044651	1.86	14	1454916_s_at	AV087417	1.733	15
**ARFIP1**	C	1369026_at	ADP-ribosylation factor interacting protein 1	NM_021763	2.2	6	214483_s_at	AF124489	1.5	12	1454916_s_at	AV087417	1.733	15
**ARHGAP1**	C	1393113_at	Rho GTPase activating protein 1 (predicted)	AA957578	1.88	4	202117_at	BG468434	1.51	10	1458118_at	AV233159	2.512	17
**CD164**	C	1386886_at	CD164 antigen	NM_031812	2.35	6	208654_s_at	BF669455	2.26	14	1416440_at	NM_016898	1.794	17
**ARHGEF9**		1377216_at	Cdc42 guanine nucleotide exchange factor (GEF) 9	BI295997	1.77	0	203263_s_at	AI625739	1.49	10	1436577_at	BB380620	3.924	17
**ARHGEF9**		1377216_at	Cdc42 guanine nucleotide exchange factor (GEF) 9	BI295997	1.77	0	203263_s_at	AI625739	1.49	10	1436258_at	BE651574	3.665	15
**ARMCX2**		1373156_at	armadillo repeat containing, X-linked 2	AI385330	4.91	4	203404_at	NM_014782	2.05	14	1456739_x_at	BB392869	3.011	15
**ARMCX2**		1373156_at	armadillo repeat containing, X-linked 2	AI385330	4.91	4	203404_at	NM_014782	2.05	14	1437849_x_at	BB443695	2.746	15
**ARMCX2**		1373156_at	armadillo repeat containing, X-linked 2	AI385330	4.91	4	203404_at	NM_014782	2.05	14	1415780_a_at	NM_026139	1.953	13
**ARMCX3**	A	1373958_at	armadillo repeat containing, X-linked 3 (similar to ALEX3 protein)	AA943149	1.82	6	217858_s_at	NM_016607	1.29	10	1424373_at	AK004598	3.482	17
**ARMET**	C	1372352_at	arginine-rich, mutated in early stage tumors (predicted)	AI170666	3.46	6	202655_at	NM_006010	1.89	14	1428112_at	AK014338	1.999	17
**ASAH1**	C	1369077_at	N-acylsphingosine amidohydrolase 1	NM_053407	2.61	6	210980_s_at	U47674	1.61	8	1416735_at	NM_019734	2.675	17
**ASAH1**	C	1374770_at	N-acylsphingosine amidohydrolase 1	AA800062	2.22	6	213702_x_at	AI934569	1.56	10	1416735_at	NM_019734	2.675	17
**ATF2**	C	1369472_a_a	activating transcription factor 2	NM_031018	1.98	6	212984_at	BE786164	2.13	12	1452116_s_at	BM119623	1.691	15
**ATF3**	B	1369268_at	activating transcription factor 3	NM_012912	7.46	4	202672_s_at	NM_001674	9.16	10	1449363_at	BC019946	11.24	17

ATF4	C	1367624_at	activating transcription factor 4	NM_024403	2.54	6	200779_at	NM_001675	1.27	8	1448135_at	M94087	1.798	15
ATP2A2	C	1369065_a_a	ATPase, Ca++ transporting, cardiac muscle, slow twitch 2	NM_017290	2.4	4	209186_at	M23114	1.71	8	1416551_at	NM_009722	2.461	13
ATP2A2	C	1370426_a_a	ATPase, Ca++ transporting, cardiac muscle, slow twitch 2	J04024	2.56	6	209186_at	M23114	1.71	8	1427250_at	AA245637	2.08	17
ATP2A3	C	1368477_at	ATPase, Ca++ transporting, ubiquitous	NM_012914	10.1	6	207522_s_at	NM_005173	5.44	14	1450124_a_at	NM_016745	2.63	13
ATP2A3	C	1368477_at	ATPase, Ca++ transporting, ubiquitous	NM_012914	10.1	6	213036_x_at	Y15724	3.91	14	1450124_a_at	NM_016745	2.63	13
ATP2A3	C	1368477_at	ATPase, Ca++ transporting, ubiquitous	NM_012914	10.1	6	207521_s_at	AF068220	2.88	14	1450124_a_at	NM_016745	2.63	13
ATP2A3	C	1371165_a_a	ATPase, Ca++ transporting, ubiquitous	AF458230	6.39	6	213042_s_at	AA877910	3.35	14	1450124_a_at	NM_016745	2.63	13
ATP6V1B2	A	1387664_at	ATPase, H+ transporting, V1 subunit B, isoform 2	NM_057213	2.46	2	201089_at	NM_001693	2.47	10	1446465_at	BB500880	1.77	17
ATP6V1B2	A	1371402_at	ATPase, H+ transporting, V1 subunit B, isoform 2	BM390600	2.16	2	201089_at	NM_001693	2.47	10	1446465_at	BB500880	1.77	17
ATP6V1C1	A	1374396_at	ATPase, H+ transporting, V1 subunit C, isoform 1 (predicted)	BI293306	2.02	4	202872_at	AW024925	1.77	10	1419546_at	AA987147	1.631	15
BACE1	A	1369256_at	beta-site APP cleaving enzyme	NM_019204	1.46	6	217904_s_at	NM_012104	2.21	10	1421825_at	AF200346	4.681	17
BACE1	A	1369256_at	beta-site APP cleaving enzyme	NM_019204	1.46	6	217904_s_at	NM_012104	2.21	10	1455826_a_at	BB114336	3.043	17
BEXL1	A	1373458_at	Brain expressed X-linked 4	BI289546	6.38	4	218332_at	NM_018476	7.43	12	1428209_at	AK010400	4.119	17
BEXL1	A	1373458_at	Brain expressed X-linked 4	BI289546	6.38	4	215440_s_at	AL523320	3.27	12	1428209_at	AK010400	4.119	17
BICD1	C	1373902_at	Bicaudal D homolog 1 (Drosophila) (predicted)	AI230054	1.83	6	214806_at	U90030	1.4	8	1437548_at	AV367839	6.374	17
BICD1	C	1373902_at	Bicaudal D homolog 1 (Drosophila) (predicted)	AI230054	1.83	6	214806_at	U90030	1.4	8	1438701_at	BB130665	2.79	15
BICD1	C	1373902_at	Bicaudal D homolog 1 (Drosophila) (predicted)	AI230054	1.83	6	214806_at	U90030	1.4	8	1442298_at	AI426427	2.117	17
BICD1	C	1373902_at	Bicaudal D homolog 1 (Drosophila) (predicted)	AI230054	1.83	6	214806_at	U90030	1.4	8	1451684_a_at	BC016192	1.803	15
BTG2	B	1386995_at	B-cell translocation gene 2, anti-proliferative	BI288701	4.46	4	201235_s_at	BG339064	4.38	14	1448272_at	NM_007570	2.977	13
BTG2	B	1386994_at	B-cell translocation gene 2, anti-proliferative	BI288701	2.22	4	201236_s_at	NM_006763	2.96	14	1416250_at	NM_007570	5.667	17
CACNA1A	A	1367821_a_a	calcium channel, voltage-dependent, P/Q type, alpha 1A subunit	BF400631	1.44	6	214933_at	AA769818	2.29	8	1459996_at	AI848293	3.153	17
CACNA1A	A	1367821_a_a	calcium channel, voltage-dependent, P/Q type, alpha 1A subunit	BF400631	1.44	6	214933_at	AA769818	2.29	8	1450510_a_at	AB066608	1.911	15
CACNA1D	A	1370640_a_a	calcium channel, voltage-dependent, L type, alpha 1D subunit	AF370009	1.58	6	210108_at	BE550599	5.53	14	1427974_s_at	BB048682	3.773	17
CACNA1D	A	1370640_a_a	calcium channel, voltage-dependent, L type, alpha 1D subunit	AF370009	1.58	6	207998_s_at	NM_000720	1.56	14	1427974_s_at	BB048682	3.773	17
CACNB2	A	1388023_a_a	calcium channel, voltage-dependent, beta 2 subunit	AF394941	1.45	4	213714_at	AI040163	1.7	8	1444693_at	BG069383	4.709	17
CACNB2	A	1370178_at	calcium channel, voltage-dependent, beta 2 subunit	BG373973	3.03	6	213714_at	AI040163	1.7	8	1456401_at	BB078175	2.511	13
CADM1		1370594_at	immunoglobulin superfamily, member 1	AF322217	1.35	4	207695_s_at	NM_001555	1.22	10	1447702_x_at	AV152162	5.647	15
CADM1		1370410_at	immunoglobulin superfamily, member 1	AF322216	1.74	2	207695_s_at	NM_001555	1.22	10	1438589_at	BB023061	1.902	17
CADPS	A	1368523_at	Ca<2+-dependent activator protein for secretion	NM_013219	4.97	2	204814_at	NM_003716	6.22	14	1445586_at	BB283411	6.856	17
CADPS	A	1368523_at	Ca<2+-dependent activator protein for secretion	NM_013219	4.97	2	204814_at	NM_003716	6.22	14	1448955_s_at	NM_012061	3.46	13
CADPS	A	1368523_at	Ca<2+-dependent activator protein for secretion	NM_013219	4.97	2	204814_at	NM_003716	6.22	14	1458298_at	AI462674	3.21	13
CAMKK2		1368753_at	calcium/calmodulin-dependent protein kinase kinase 2, beta	NM_031338	1.43	6	207359_at	NM_006549	1.65	14	1424476_at	BI157430	3.018	15
CANX	C	1371687_at	calnexin	AA848326	2.29	6	208853_s_at	L18887	2.51	14	1428935_at	AI988026	3.552	17
CANX	C	1371686_at	calnexin	AA848326	1.9	6	208852_s_at	AI761759	2.18	14	1428935_at	AI988026	3.552	17
CANX	C	1388442_at	calnexin	AA848326	2.19	6	200068_s_at	M94859	1.32	10	1428935_at	AI988026	3.552	17
CCDC6		1372134_at	coiled-coil-helix-coiled-coil-helix domain containing 6 (predicted)	AW918650	1.55	6	204716_at	NM_005436	1.62	12	1423779_at	BC011331	1.989	15
CCPG1	B	1375027_at	Cell cycle progression 1 (predicted)	AI407365	1.9	6	222156_x_at	AK022459	3.38	14	1424420_at	BC006717	2.791	17
CCPG1	B	1375027_at	Cell cycle progression 1 (predicted)	AI407365	1.9	6	221511_x_at	AB033080	2.15	14	1420127_s_at	C85100	3.918	17
CCPG1	B	1375027_at	Cell cycle progression 1 (predicted)	AI407365	1.9	6	221156_x_at	NM_004748	1.81	14	1420127_s_at	C85100	3.918	17
CD164		1386886_at	CD164 antigen	NM_031812	2.35	6	208405_s_at	NM_006016	1.62	10	1416440_at	NM_016898	1.794	17
CDK2AP1		1375326_at	CDK2 (cyclin-dependent kinase 2)-associated protein 1 (predicted)	AI602458	2.37	6	201938_at	NM_004642	1.51	10	1458451_at	BE985383	3.259	17
CHGA	B	1387235_at	chromogranin A	NM_021655	14.3	4	204697_s_at	NM_001275	18.2	14	1418149_at	NM_007693	25.11	17
CHM		1369227_at	choroidermia	NM_017067	2.4	6	207099_s_at	NM_000390	1.5	14	1448712_at	NM_018818	2.281	17
CHM		1373802_at	Choroidermia	BF392902	2.15	6	207099_s_at	NM_000390	1.5	14	1448712_at	NM_018818	2.281	17
CHST3		1369512_at	carbohydrate (chondroitin 6/keratan) sulfotransferase 3	NM_053408	1.51	6	208252_s_at	NM_004273	1.2	10	1460322_at	NM_016803	2.43	17
CKAP4		1375654_at	cytoskeleton-associated protein 4 (predicted)	BI278813	1.41	6	200999_s_at	NM_006825	1.85	8	1426755_at	BB312117	2.106	13
CLCN3	B	1392453_at	chloride channel 3	AI763523	1.74	0	201735_s_at	NM_001829	2.89	14	1416610_a_at	NM_007711	1.967	13
CLCN3	B	1380547_at	chloride channel 3	BI288519	1.82	0	201734_at	AI760629	2.43	14	1416610_a_at	NM_007711	1.967	13
CLCN3	B	1380547_at	chloride channel 3	BI288519	1.82	0	201732_s_at	AF029346	1.98	14	1416610_a_at	NM_007711	1.967	13
CLCN3	B	1380547_at	chloride channel 3	BI288519	1.82	0	201733_at	AA902971	1.77	10	1416610_a_at	NM_007711	1.967	13

**CNOT2**		1376067_at	CCR4-NOT transcription complex, subunit 2	AA800637	1.5	4	222181_at	BG105204	1.3	14	1447212_at	BB165759	2.197	17
**COPA**		1388377_at	coatomer protein complex subunit alpha (predicted)	AW142717	1.91	6	214336_s_at	AI621079	1.33	8	1437274_at	BM204808	3.998	17
**COPA**		1388377_at	coatomer protein complex subunit alpha (predicted)	AW142717	1.91	6	214336_s_at	AI621079	1.33	8	1415706_at	BQ175820	1.619	17
**COPB2**	C	1367454_at	coatomer protein complex, subunit beta 2 (beta prime)	NM_021765	1.88	6	201098_at	NM_004766	1.85	14	1456175_a_at	AV109541	1.69	17
**COPG**		1372464_at	coatomer protein complex, subunit gamma	BE109050	2.1	6	217749_at	NM_016128	1.4	12	1416017_at	BC024686	2.018	15
**COPG**		1371810_at	Coatomer protein complex, subunit gamma	BF284093	1.39	4	217749_at	NM_016128	1.4	12	1415670_at	BC024686	2.364	15
**COX15**	C	1377444_at	COX15 homolog, cytochrome c oxidase assembly protein (yeast)	BE120680	1.77	6	221550_at	BC002382	1.15	14	1426693_x_at	BC021498	2.671	17
**COX15**	C	1377444_at	COX15 homolog, cytochrome c oxidase assembly protein (yeast)	BE120680	1.77	6	221550_at	BC002382	1.15	14	1452146_a_at	BC021498	1.564	15
**CPE**	B	1386921_at	carboxypeptidase E	NM_013128	12.3	6	201116_s_at	AI922855	7.13	14	1415949_at	BC010197	3.248	15
**CPE**	B	1386921_at	carboxypeptidase E	NM_013128	12.3	6	201117_s_at	NM_001873	6.85	14	1415949_at	BC010197	3.248	15
**CPEB1**		1392996_at	cytoplasmic polyadenylation element binding protein 1 (predicted)	BG668435	2.18	6	219578_s_at	NM_030594	1.27	10	1417960_at	NM_007755	8.554	17
**CPLX2**	A	1368584_a_a	complexin 2	NM_053878	1.63	2	206368_at	NM_006650	2.59	14	1421477_at	NM_009946	3.797	15
**CPLX2**	A	1368584_a_a	complexin 2	NM_053878	1.63	2	206368_at	NM_006650	2.59	14	1455672_s_at	BE946238	2.065	15
**CRMP1**	A	1387899_at	collapsin response mediator protein 1	U52102	3.51	0	202517_at	NM_001313	1.81	8	1448289_at	AB006714	2.205	13
**CRYZ**		1374628_at	crystallin, zeta (predicted)	AI232098	1.38	4	202950_at	NM_001889	3.03	10	1430546_at	AK010433	1.905	15
**CTNNA1**	C	1371921_at	catenin (cadherin-associated protein), alpha 1, 102kDa	BM986272	1.88	6	200765_x_at	NM_001903	1.6	8	1443662_at	BE989574	3.473	17
**CTNNA1**	C	1371921_at	catenin (cadherin-associated protein), alpha 1, 102kDa	BM986272	1.88	6	200764_s_at	AI826881	1.54	8	1443662_at	BE989574	3.473	17
**CYB561**		1398383_at	cytochrome b-561 (predicted)	BI282164	1.5	4	209163_at	AL514271	2.34	14	1417507_at	BC006732	2.049	11
**CYB561**		1398383_at	cytochrome b-561 (predicted)	BI282164	1.5	4	209164_s_at	BC002976	1.23	12	1417507_at	BC006732	2.049	11
**DAAM1**		1389318_at	Dishevelled associated activator of morphogenesis 1 (predicted)	BF282986	1.36	4	216060_s_at	AK021890	1.97	12	1431035_at	AW988556	2.658	17
**DACH1**	B	1384907_at	similar to dachshund homolog 1 (Drosophila)	not on RG230A array			205471_s_at	AW772082	3.46	14	1433743_at	BG075820	3.216	17
**DAD1**	C	1367465_at	defender against cell death 1	AI013627	2.1	6	200046_at	NM_001344	1.61	14	1454860_x_at	BI966630	3.403	17
**DAD1**	C	1367465_at	defender against cell death 1	AI013627	2.1	6	200046_at	NM_001344	1.61	14	1418528_a_at	NM_010015	1.975	17
**DCX**	A	1374966_at	doublecortin	BE109057	12	6	204850_s_at	NM_000555	1.69	12	1418139_at	BB418548	29.54	17
**DCX**	A	1374966_at	doublecortin	BE109057	12	6	204850_s_at	NM_000555	1.69	12	1418141_at	BB418548	9.299	17
**DCX**	A	1374966_at	doublecortin	BE109057	12	6	204851_s_at	AF040254	2.46	12	1448974_at	BB418548	2.602	15
**DDIT3**	A	1369590_a_a	DNA-damage inducible transcript 3	NM_024134	9.57	6	209383_at	BC003637	1.95	14	1417516_at	NM_007837	3.35	17
**DDIT3**	A	1376178_at	DNA-damage inducible transcript 3	BF403703	7.01	4	209383_at	BC003637	1.95	14	1443897_at	BB200603	2.104	15
**DDOST**	C	1372247_at	dolichyl-di-phosphooligosaccharide-protein glycotransferase (predictec	BF282907	2.63	6	208674_x_at	BC002594	1.91	12	1416493_at	NM_007838	2.059	17
**DDOST**	C	1372247_at	dolichyl-di-phosphooligosaccharide-protein glycotransferase (predictec	BF282907	2.63	6	208675_s_at	D29643	1.84	14	1416493_at	NM_007838	2.059	17
**DDR1**	B	1370216_at	discoidin domain receptor family, member 1	L26525	1.76	2	210749_x_at	L11315	4.37	14	1459990_at	BF462661	7.157	17
**DDR1**	B	1370216_at	discoidin domain receptor family, member 1	L26525	1.76	2	207169_x_at	NM_001954	5.07	14	1439382_x_at	BB378700	2.187	15
**DDR1**	B	1370216_at	discoidin domain receptor family, member 1	L26525	1.76	2	208779_x_at	L20817	4.73	14	1439382_x_at	BB378700	2.187	15
**DDX50**		1390688_at	DEAD (Asp-Glu-Ala-Asp) box polypeptide 50 (predicted)	AA799576	1.55	2	221699_s_at	AF334103	1.71	12	1439082_at	AA518255	3.026	17
**DDX50**		1390688_at	DEAD (Asp-Glu-Ala-Asp) box polypeptide 50 (predicted)	AA799576	1.55	2	221699_s_at	AF334103	1.71	12	1417875_at	NM_053183	2.205	17
**DDX6**		1398945_at	DEAD (Asp-Glu-Ala-Asp/His) box polypeptide 6 (mapped)	AA851439	1.48	4	204909_at	NM_004397	1.27	14	1424598_at	BC021452	6.716	17
**DDX6**		1398945_at	DEAD (Asp-Glu-Ala-Asp/His) box polypeptide 6 (mapped)	AA851439	1.48	4	204909_at	NM_004397	1.27	14	1447789_x_at	BB150520	3.517	17
**DLG5**		1377121_at	Discs, large homolog 5 (Drosophila) (predicted)	AW520410	2.01	6	201681_s_at	AB011155	1.58	12	1431187_s_at	BF140264	2.872	13
**DNAJB9**	B	1387116_at	DnaJ (Hsp40) homolog, subfamily B, member 9	NM_012699	3.87	6	202843_at	NM_012328	3.68	14	1417191_at	NM_013760	1.903	13
**DNAJB9**	B	1387116_at	DnaJ (Hsp40) homolog, subfamily B, member 9	NM_012699	3.87	6	202842_s_at	AL080081	2.83	14	1417191_at	NM_013760	1.903	13
**DNAJC1**		1376079_at	DnaJ (Hsp40) homolog, subfamily C, member 1 (predicted)	BI295806	1.83	4	218409_s_at	NM_022365	1.59	12	1459791_at	AV129500	2.56	17
**DNM1L**	C	1369220_at	dynamin 1-like	NM_053655	1.36	2	203105_s_at	NM_012062	1.4	8	1428008_at	BC027538	5.16	17
**DNM1L**	C	1388022_a_a	dynamin 1-like	AF132727	1.43	2	203105_s_at	NM_012062	1.4	8	1428087_at	BM249101	1.591	15
**DPH4**	C	1390168_a_a	DPH4, JJJ3 homolog (S. cerevisiae) (zinc finger, CSL-type containing	BI289564	2.92	6	213853_at	AL050199	1.68	12	1451389_at	AY028460	10.74	17
**DPH4**	C	1390168_a_a	DPH4, JJJ3 homolog (S. cerevisiae) (zinc finger, CSL-type containing	BI289564	2.92	6	213853_at	AL050199	1.68	12	1430718_s_at	AV313469	7.305	17
**DST**		1375357_at	dystonia 1	BE112237	1.35	4	220154_at	NM_020388	1.36	10	1450119_at	NM_010081	2.144	13
**DSTN**	C	1371375_at	destrin	BI285599	2.79	6	201021_s_at	BF697964	1.58	10	1439535_at	BB204161	2.742	17
**DSTN**	C	1375881_at	destrin	AI170442	2.41	6	201021_s_at	BF697964	1.58	10	1439535_at	BB204161	2.742	17
**DYNC2LI1**	C	1387782_at	dynein light chain-2	NM_080697	1.65	6	203762_s_at	NM_016008	1.33	10	1428446_at	AK008822	2.038	17
**DYNLT1**		1388903_at	T-complex associated-testis-expressed 1-like (Protein 91/23)	AI179335	1.51	4	201999_s_at	NM_006519	1.66	8	1420614_at	NM_025975	1.846	15

**DYNLT1**		1388903_at	T-complex associated-testis-expressed 1-like (Protein 91/23)	AI179335	1.51	4	201999_s_at	NM_006519	1.66	8	1449928_at	NM_025975	1.767	15
**EBAG9**	C	1391510_at	estrogen receptor-binding fragment-associated gene 9 (predicted)	BF398331	1.7	6	204274_at	AA812215	1.86	14	1422669_at	AY009091	1.607	13
**EBAG9**	C	1391510_at	estrogen receptor-binding fragment-associated gene 9 (predicted)	BF398331	1.7	6	204278_s_at	NM_004215	1.27	8	1422669_at	AY009091	1.607	13
**ECHDC1**	C	1374585_at	enoyl Coenzyme A hydratase domain containing 1	AW915152	1.7	4	219974_x_at	NM_018479	1.2	8	1425001_at	BC023104	1.523	17
**EFCBP2**	A	1368443_at	EF hand calcium binding protein 2	NM_133415	1.73	6	215005_at	AV723666	1.29	10	1418881_at	NM_054095	5.643	17
**EGR4**	B	1387442_at	early growth response 4	NM_019137	7.92	2	207767_s_at	NM_001965	2.18	14	1449977_at	NM_020596	4.169	17
**EGR4**	B	1375784_at	Early growth response 4	BF412806	1.37	6	207768_at	NM_001965	34.1	14	1449977_at	NM_020596	4.169	17
**EIF3S10**	C	1389968_at	eukaryotic translation initiation factor 3, subunit 10 (theta) (predicted)	BG376953	1.38	4	200596_s_at	BE614908	1.27	14	1416661_at	AW701127	8.263	17
**EIF3S10**	C	1389968_at	eukaryotic translation initiation factor 3, subunit 10 (theta) (predicted)	BG376953	1.38	4	200595_s_at	NM_003750	1.65	12	1416660_at	AW701127	1.907	13
**ELAVL4**	A	1389975_at	ELAV (embryonic lethal, abnormal vision, Drosophila)-like 4 (Hu antige	BE116949	2.23	4	206051_at	NM_021952	3.69	10	1428741_at	AK013588	6.031	15
**ELAVL4**	A	1389975_at	ELAV (embryonic lethal, abnormal vision, Drosophila)-like 4 (Hu antige	BE116949	2.23	4	206051_at	NM_021952	3.69	10	1450258_a_at	NM_010488	3.83	15
**ELAVL4**	A	1389975_at	ELAV (embryonic lethal, abnormal vision, Drosophila)-like 4 (Hu antige	BE116949	2.23	4	206051_at	NM_021952	3.69	10	1452894_at	AK013588	3.563	15
**ELAVL4**	A	1389975_at	ELAV (embryonic lethal, abnormal vision, Drosophila)-like 4 (Hu antige	BE116949	2.23	4	206051_at	NM_021952	3.69	10	1457399_at	BB211758	2.708	17
**ELMO3**		1374995_at	engulfment and cell motility 3, ced-12 homolog (C. elegans) (predicted	BI291645	3.9	4	219411_at	NM_024712	1.43	10	1434489_at	AI481208	2.041	17
**EPB41L3**	A	1370503_s_a	erythrocyte protein band 4.1-like 3	BM383451	3.08	4	206710_s_at	NM_012307	2.14	10	1440595_at	BB036542	7.606	17
**EPB41L3**	A	1368515_at	erythrocyte protein band 4.1-like 3	NM_053927	2.52	4	212681_at	AI770004	3.1	14	1426010_a_at	AF177146	4.595	15
**ERO1L**	B	1389297_at	ERO1-like (S. cerevisiae)	AI146215	2.61	6	218498_s_at	NM_014584	1.41	12	1449324_at	BM234652	2.092	13
**ERO1LB**	B		ERO1-like beta (S. cerevisiae)	not on RG230.20 array			220012_at	NM_019891	14	14	1449948_at	NM_026184	18.01	17
**ERO1LB**	B		ERO1-like beta (S. cerevisiae)	not on RG230.20 array			220012_at	NM_019891	14	14	1434714_at	BB234316	18.2	17
**ERO1LB**	B		ERO1-like beta (S. cerevisiae)	not on RG230.20 array			220012_at	NM_019891	14	14	1425705_a_at	AF489856	12.61	17
**ERP29**	C	1387031_at	endoplasmic retuclum protein 29	U36482	2.23	6	201216_at	NM_006817	2.41	14	1453634_a_at	AK013303	6.92	17
**FBXL15**	C	1374114_at	F-box and leucine-rich repeat protein 15 (predicted)	BI282971	1.86	6	218938_at	NM_024326	1.71	12	1417855_at	NM_133694	5.81	17
**FIP1L1**	C	1389621_at	FIP1 like 1 (S. cerevisiae) (predicted)	BE113454	1.45	4	221007_s_at	NM_030917	1.43	8	1428280_at	BM199874	3.019	17
**FIP1L1**	C	1389621_at	FIP1 like 1 (S. cerevisiae) (predicted)	BE113454	1.45	4	221007_s_at	NM_030917	1.43	8	1452720_a_at	BM199874	1.883	15
**FKBP2**	C	1375669_at	FK506 binding protein 2 (predicted)	BI285619	2.1	6	203391_at	NM_004470	2.17	14	1450694_at	NM_008020	1.951	17
**FOSB**	C	1375043_at	FBJ murine osteosarcoma viral oncogene homolog	BF415939	14.6	6	202768_at	NM_006732	5.04	10	1422134_at	NM_008036	12.58	17
**FOSB**	C	1375043_at	FBJ murine osteosarcoma viral oncogene homolog	BF415939	14.6	6	202768_at	NM_006732	5.04	10	1423100_at	AV026617	7.402	17
**FOXA2**		1368711_at	forkhead box A2	NM_012743	10.6	4	210103_s_at	AB028021	4.43	14	1422833_at	NM_010446	10.66	17
**FOXA2**		1368711_at	forkhead box A2	NM_012743	10.6	4	40284_at	AB028021	2.88	14	1422833_at	NM_010446	10.66	17
**G6PC2**	B		glucose-6-phosphatase, catalytic, 2	not on RG230.20 array			221453_at	NM_021176	8.76	14	1423529_at	Z47787	51.7	17
**GAD1**	A	1368344_at	glutamate decarboxylase 1	NM_017007	44.4	6	205278_at	NM_000817	2.48	8	1416561_at	AF326547	2.981	13
**GAD1**	A	1370760_a_a	glutamate decarboxylase 1	M38350	7.6	6	205278_at	NM_000817	2.48	8	1416561_at	AF326547	2.981	13
**GADD45G**		1388792_at	growth arrest and DNA-damage-inducible 45 gamma (predicted)	AI599423	3.34	6	204121_at	NM_006705	1.65	12	1453851_a_at	AK007410	8.5	17
**GARNL1**	C	1368994_a_a	GTPase activating RANGAP domain-like 1	AF041107	1.43	6	214855_s_at	AL050050	1.27	8	1458117_at	BB468955	2.343	17
**GARNL4**	B		GTPase activating Rap/RanGAP domain-like 4	not on RG230.20 array			213280_at	AK000478	5.06	14	1434754_at	AU067654	6.699	17
**GARS**		1388715_at	glycyl-tRNA synthetase (predicted)	AA818089	1.72	6	208693_s_at	D30658	1.58	12	1423784_at	BC021747	1.525	17
**GARS**	C	1388715_at	glycyl-tRNA synthetase (predicted)	AA818089	1.72	6	208693_s_at	D30658	1.58	12	1423784_at	BC021747	1.525	17
**GCGR**		1370522_at	glucagon receptor	L04796	3.45	4	210565_at	U03469	1.36	12	1450127_a_at	NM_008101	3.092	15
**GCK**		1387312_a_a	glucokinase	NM_012565	1.86	4	211167_s_at	M69051	2.05	14	1419146_a_at	BC011139	14.77	17
**GCK**		1387312_a_a	glucokinase	NM_012565	1.86	4	211167_s_at	M69051	2.05	14	1425303_at	L38990	8.96	17
**GFPT1**	C	1377314_at	glutamine fructose-6-phosphate transaminase 1	BE104391	1.36	4	202722_s_at	NM_002056	3.65	14	1444329_at	BB533749	2.147	17
**GFPT1**	C	1373373_at	Glutamine fructose-6-phosphate transaminase 1	BI295011	2.54	6	202721_s_at	BE645771	1.64	12	1444329_at	BB533749	2.147	17
**GGA2**	C	1372408_at	golgi associated, gamma adaptin ear containing, ARF binding protein 2	BI278620	1.81	6	210658_s_at	BC000284	1.7	10	1428141_at	AK004632	2.12	13
**GGA2**	C	1372408_at	golgi associated, gamma adaptin ear containing, ARF binding protein 2	BI278620	1.81	6	208913_at	AA868560	1.52	10	1428141_at	AK004632	2.12	13
**GGA2**	C	1372408_at	golgi associated, gamma adaptin ear containing, ARF binding protein 2	BI278620	1.81	6	208915_s_at	AF190863	1.16	10	1428141_at	AK004632	2.12	13
**GGA2**	C	1372408_at	golgi associated, gamma adaptin ear containing, ARF binding protein 2	BI278620	1.81	6	214190_x_at	AI799984	1.14	12	1428141_at	AK004632	2.12	13
**GLA**		1373199_at	Galactosidase, alpha (mapped)	AA943573	1.57	4	214430_at	NM_000169	1.14	10	1449006_at	NM_013463	2.331	17
**GLG1**	C	1387367_at	golgi apparatus protein 1	NM_017211	2.31	6	214730_s_at	AK025457	1.51	12	1448580_at	BG074127	2.054	17
**GLG1**	C	1387367_at	golgi apparatus protein 1	NM_017211	2.31	6	212045_at	N32761	1.15	12	1448580_at	BG074127	2.054	17
**GLP1R**	A	1369699_at	glucagon-like peptide 1 receptor	NM_012728	8.09	6	208391_s_at	NM_002062	1.6	14	1422330_at	NM_021332	7.109	17

**GLP1R**	A	1369699_at	glucagon-like peptide 1 receptor	NM_012728	8.09	6	208401_s_at	U01157	1.35	12	1422330_at	NM_021332	7.109	17
**GLP1R**	A	1369699_at	glucagon-like peptide 1 receptor	NM_012728	8.09	6	208390_s_at	U01104	1.16	12	1422330_at	NM_021332	7.109	17
**GLP1R**	A	1369699_at	glucagon-like peptide 1 receptor	NM_012728	8.09	6	211232_x_at	L23503	1.19	12	1458719_at	BM233846	1.828	17
**GLRX2**		1374340_at	Glutaredoxin 2 (thioltransferase)	AA848536	1.38	4	219933_at	NM_016066	1.26	8	1422998_a_at	NM_023505	1.754	15
**GLRX2**		1373675_at	glutaredoxin 2 (thioltransferase) (predicted)	AW525635	1.36	4	219933_at	NM_016066	1.26	8	1422998_a_at	NM_023505	1.754	15
**GMPPA**		1372859_at	GDP-mannose pyrophosphorylase A	AI598958	1.58	6	218070_s_at	NM_013335	1.34	8	1417578_a_at	NM_133708	1.878	17
**GOLGA4**	C	1394347_at	golgi autoantigen golgin subtype a4; tGolgin-1	BF568007	2.98	4	201567_s_at	NM_002078	1.89	10	1448803_at	NM_018748	6.103	17
**GOLGA4**	C	1394347_at	golgi autoantigen golgin subtype a4; tGolgin-1	BF568007	2.98	4	201567_s_at	NM_002078	1.89	10	1460213_at	NM_018748	2.014	15
**GOLGA5**	C	1376650_at	golgi autoantigen, golgin subfamily a, 5 (predicted)	BF399323	1.66	6	218241_at	NM_005113	1.84	12	1418447_at	NM_013747	1.771	17
**GORASP2**	C	1370325_at	golgi reassembly stacking protein 2	AF110267	1.49	6	208843_s_at	BC001408	1.57	12	1424710_a_at	BC016455	1.879	17
**GORASP2**	C	1370325_at	golgi reassembly stacking protein 2	AF110267	1.49	6	207812_s_at	NM_015530	1.36	12	1424710_a_at	BC016455	1.879	17
**GORASP2**	C	1370325_at	golgi reassembly stacking protein 2	AF110267	1.49	6	208842_s_at	W93787	1.34	14	1424710_a_at	BC016455	1.879	17
**GOSR2**		1374900_at	golgi SNAP receptor complex member 2	BM388807	1.8	6	213144_at	AI074611	1.19	12	1419371_s_at	NM_019650	1.636	17
**GOSR2**		1375438_at	golgi SNAP receptor complex member 2	AA964687	1.65	6	213180_s_at	BE895285	1.19	8	1419371_s_at	NM_019650	1.636	17
**GPD2**		1387670_at	glycerol-3-phosphate dehydrogenase 2	U08027	1.4	2	210007_s_at	U36310	1.75	12	1452741_s_at	BQ175968	4.985	17
**GPD2**		1369666_at	Glycerol-3-phosphate dehydrogenase 2	U08027	1.34	0	210007_s_at	U36310	1.75	12	1452741_s_at	BQ175968	4.985	17
**GSPT1**		1371585_at	G1 to S phase transition 1	BE108192	1.41	6	201912_s_at	NM_002094	1.65	10	1455173_at	AW537663	1.648	13
**GSPT1**		1371585_at	G1 to S phase transition 1	BE108192	1.41	6	215438_x_at	BE906054	1.43	8	1455173_at	AW537663	1.648	13
**GSPT1**		1371585_at	G1 to S phase transition 1	BE108192	1.41	6	217595_at	AV701723	1.24	10	1455173_at	AW537663	1.648	13
**HADH**	B	1370237_at	L-3-hydroxyacyl-Coenzyme A dehydrogenase, short chain	AA799574	5.96	6	201036_s_at	NM_005327	4.88	14	1436756_x_at	BB114220	2.089	13
**HADH**	B	1370237_at	L-3-hydroxyacyl-Coenzyme A dehydrogenase, short chain	AA799574	5.96	6	211569_s_at	AF001903	4.71	14	1455972_x_at	AV018774	2.261	13
**HDGFRP3**	B	1370458_at	hepatoma-derived growth factor, related protein 3	AF389347	1.41	2	209524_at	AK001280	3.21	8	1435977_at	BM240385	2.504	13
**HDGFRP3**	B	1374151_at	Hepatoma-derived growth factor, related protein 3	BI289940	1.59	0	209524_at	AK001280	3.21	8	1450924_at	BB291880	1.796	13
**HDLBP**	C	1398859_at	high density lipoprotein binding protein	U90725	1.52	4	221767_x_at	AA515560	1.47	10	1449615_s_at	C77256	9.396	17
**HDLBP**	C	1388918_at	High density lipoprotein binding protein	BG378074	1.73	6	200643_at	NM_005336	1.25	8	1415988_at	BG065877	9.246	17
**HDLBP**	C	1388918_at	High density lipoprotein binding protein	BG378074	1.73	6	200643_at	NM_005336	1.25	8	1419806_at	C77256	7.508	17
**HERPUD1**	C	1367741_at	homocysteine-inducible, endoplasmic reticulum stress-inducible, ubiqu	NM_053523	4.43	6	217168_s_at	AF217990	1.72	14	1435626_a_at	AI835088	2.46	15
**HERPUD1**	C	1367741_at	homocysteine-inducible, endoplasmic reticulum stress-inducible, ubiqu	NM_053523	4.43	6	217168_s_at	AF217990	1.72	14	1435626_a_at	AI835088	2.46	15
**HIC2**		1376400_at	hypermethylated in cancer 2 (predicted)	BG380445	1.08	4	212965_at	BE465318	1.21	12	1454944_at	BE648070	2.167	15
**HLXB9**			homeo box HB9	not on RG230.20 array			214614_at	AI738662	3.6	14	1460299_at	NM_019944	11.54	17
**HMGN3**	C	1371989_at	high mobility group nucleosomal binding domain 3	BM386357	2.76	4	209377_s_at	AF274949	1.25	12	1434875_a_at	AV018952	3.707	15
**HMGN3**	C	1371989_at	high mobility group nucleosomal binding domain 3	BM386357	2.76	4	209377_s_at	AF274949	1.25	12	1431777_a_at	AK002970	2.612	15
**HNRPC**		1371505_at	heterogeneous nuclear ribonucleoprotein C (predicted)	BG381750	1.44	4	200751_s_at	BE898861	1.41	10	1418693_at	NM_016884	4.408	17
**HNRPC**	C	1371505_at	heterogeneous nuclear ribonucleoprotein C (predicted)	BG381750	1.44	4	200014_s_at	NM_004500	1.24	10	1418693_at	NM_016884	4.408	17
**HNRPDL**	C	1372810_at	heterogeneous nuclear ribonucleoprotein D-like (predicted)	BF281256	1.5	4	209067_s_at	D89092	1.4	8	1428224_at	AV101245	1.976	17
**HSP90B1**	C	1388331_at	heat shock protein 90kDa beta (Grp94), member 1 (tumor rejection ant	BG057543	2.67	6	200598_s_at	AI582238	2.78	14	1438040_a_at	BE995678	3.137	15
**HSP90B1**	C	1388331_at	heat shock protein 90kDa beta (Grp94), member 1 (tumor rejection ant	BG057543	2.67	6	216449_x_at	AK025862	2.72	14	1438040_a_at	BE995678	3.137	15
**HSP90B1**	C	1388331_at	heat shock protein 90kDa beta (Grp94), member 1 (tumor rejection ant	BG057543	2.67	6	200599_s_at	NM_003299	1.75	14	1438040_a_at	BE995678	3.137	15
**HSPA4L**		1371783_at	heat shock 70kDa protein 4-like	AI009936	1.59	6	205543_at	NM_014278	1.73	12	1425993_a_at	D67017	2.093	13
**HSPA5 / G**	C	1370283_at	heat shock 70kD protein 5	M14050	2.92	6	211936_at	AF216292	1.95	14	1427464_s_at	AJ002387	2.179	11
**HSPA5 / G**	C	1370283_at	heat shock 70kD protein 5	M14050	2.92	6	211936_at	AF216292	1.95	14	1416064_a_at	NM_022310	1.762	15
**HYOU1**	A	1371442_at	hypoxia up-regulated 1	BI282904	4.53	6	200825_s_at	NM_006389	1.83	14	1451913_a_at	BC019785	6.251	17
**HYOU1**	A	1370665_at	hypoxia up-regulated 1	U41853	3.87	6	200825_s_at	NM_006389	1.83	14	1451913_a_at	BC019785	6.251	17
**IAPP**	B	1387660_at	islet amyloid polypeptide	M25390	106	6	207062_at	NM_000415	59.8	14	1437323_a_at	BB434117	167.2	17
**IAPP**	B	1387660_at	islet amyloid polypeptide	M25390	106	6	207062_at	NM_000415	59.8	14	1423510_at	BM569571	129.3	17
**IAPP**	B	1387660_at	islet amyloid polypeptide	M25390	106	6	207062_at	NM_000415	59.8	14	1423509_a_at	BM569571	102.7	17
**IAPP**	B	1387660_at	islet amyloid polypeptide	M25390	106	6	207062_at	NM_000415	59.8	14	1456004_x_at	BB434117	52.23	17
**ICA1**		1367787_at	islet cell autoantigen 1	NM_030844	9.43	6	210547_x_at	L21181	1.49	14	1417901_a_at	NM_010492	4.004	17
**ICA1**		1367787_at	islet cell autoantigen 1	NM_030844	9.43	6	207949_s_at	NM_004968	1.4	12	1417901_a_at	NM_010492	4.004	17
**ICA1**		1367787_at	islet cell autoantigen 1	NM_030844	9.43	6	214191_at	W67992	1.14	12	1431644_a_at	AK005965	2.774	13

**IDS**	B	1375843_at	Iduronate 2-sulfatase	BM383777	2.13	2	202439_s_at	NM_000202	5.13	14	1434751_at	BB493523	11.21	17
**IDS**	B	1375843_at	Iduronate 2-sulfatase	BM383777	2.13	2	212221_x_at	AV703259	4.47	10	1434751_at	BB493523	11.21	17
**IDS**	B	1375843_at	Iduronate 2-sulfatase	BM383777	2.13	2	202438_x_at	BF346014	4.36	12	1434751_at	BB493523	11.21	17
**IDS**	B	1375843_at	Iduronate 2-sulfatase	BM383777	2.13	2	212223_at	AI926544	4.31	12	1434751_at	BB493523	11.21	17
**IDS**	B	1375843_at	Iduronate 2-sulfatase	BM383777	2.13	2	206342_x_at	NM_006123	3.1	14	1434751_at	BB493523	11.21	17
**IDS**	B	1375843_at	Iduronate 2-sulfatase	BM383777	2.13	2	217432_s_at	AF179281	1.74	14	1434751_at	BB493523	11.21	17
**IDS**	B	1375843_at	Iduronate 2-sulfatase	BM383777	2.13	2	210666_at	AF050145	1.22	12	1434751_at	BB493523	11.21	17
**IDS**	B	1375843_at	Iduronate 2-sulfatase	BM383777	2.13	2	211782_at	BC006170	1.63	14	1455481_at	BM241722	2.978	15
**IGF1R**		1368123_at	insulin-like growth factor 1 receptor	NM_052807	1.97	6	203628_at	H05812	2.13	10	1452108_at	BE980124	6.634	17
**IGF1R**		1390671_at	Insulin-like growth factor 1 receptor	AI044666	1.35	2	203628_at	H05812	2.13	10	1452108_at	BE980124	6.634	17
**IGF2**		1367571_a_at	insulin-like growth factor 2	NM_031511	2.28	4	202410_x_at	NM_000612	1.66	10	1448152_at	NM_010514	4.061	15
**IGF2**		1367571_a_at	insulin-like growth factor 2	NM_031511	2.28	4	210881_s_at	M17863	1.62	10	1424112_at	BG092290	2.121	17
**IKBKAP**	C	1390504_at	Inhibitor of kappa light polypeptide enhancer in B-cells, kinase complex	AW524470	1.35	6	202491_s_at	NM_003640	1.31	8	1424142_at	AF367244	1.961	17
**IL13RA1**		1388711_at	interleukin 13 receptor, alpha 1	BF282650	1.82	4	211612_s_at	U62858	2.12	14	1427164_at	BB730912	3.887	17
**IL13RA1**		1370728_at	interleukin 13 receptor, alpha 1	AY044251	1.62	4	201888_s_at	U81379	2.18	12	1427164_at	BB730912	3.887	17
**IL13RA1**		1370728_at	interleukin 13 receptor, alpha 1	AY044251	1.62	4	210904_s_at	U81380	1.3	10	1427164_at	BB730912	3.887	17
**INS**	B	1370077_at	insulin 2	NM_019130	56.7	6	206598_at	NM_000207	105	14	1422446_x_at	NM_008387	46.45	17
**INS1 / INS2**	B	1387815_at	insulin 1 /// insulin 2	NM_019129	35.2	6	206598_at	NM_000207	105	14	1422447_at	NM_008386	66.14	17
**INSM1**	B		insulinoma-associated 1	not on RG230.20 array			206502_s_at	NM_002196	25.3	14	1455865_at	BB468410	54.21	17
**INSM1**	B		insulinoma-associated 1	not on RG23	0	0	206502_s_at	NM_002196	25.3	14	1421399_at	NM_016889	14.43	17
**IQGAP1**	C	1388764_at	IQ motif containing GTPase activating protein 1 (predicted)	BM387072	1.57	2	200791_s_at	NM_003870	2.26	10	1431395_a_at	BI645591	3.016	15
**IQGAP1**	C	1388764_at	IQ motif containing GTPase activating protein 1 (predicted)	BM387072	1.57	2	210840_s_at	D29640	2.16	10	1431396_at	BI645591	3.019	15
**IRAK1**	C	1373379_at	interleukin-1 receptor-associated kinase 1 (predicted)	BF396350	2.01	6	201587_s_at	NM_001569	1.7	10	1460649_at	BE949425	1.83	13
**IRF6**		1377379_at	interferon regulatory factor 6 (predicted)	BF410603	4.36	6	202597_at	AU144284	1.65	12	1418301_at	NM_016851	1.665	11
**IRF6**		1386568_at	interferon regulatory factor 6 (predicted)	BF557891	3.51	4	202597_at	AU144284	1.65	12	1418301_at	NM_016851	1.665	11
**ITM2C**		1371334_at	integral membrane protein 2C (predicted)	BG668228	1.88	-2	221004_s_at	NM_030926	2.13	10	1415961_at	NM_022417	2.191	15
**JUN**	C	1374404_at	c-Jun / v-jun sarcoma virus 17 oncogene homolog (avian)	BI288619	7.79	4	201465_s_at	BC002646	3.97	12	1448694_at	NM_010591	2.41	15
**JUN**	C	1389528_s_at	c-Jun / v-jun sarcoma virus 17 oncogene homolog (avian)	BI288619	5.27	6	201466_s_at	NM_002228	3.6	10	1417409_at	NM_010591	3.658	15
**JUN**	C	1369788_s_at	c-Jun / v-jun sarcoma virus 17 oncogene homolog (avian)	NM_021835	3.1	4	201464_x_at	BG491844	2.87	14	1448694_at	NM_010591	2.41	15
**KCNH2**		1368343_at	potassium voltage-gated channel, subfamily H (eag-related), member 2	NM_053949	1.61	4	205262_at	NM_000238	4.11	14	1449544_a_at	NM_013569	2.737	15
**KCNH2**		1368343_at	potassium voltage-gated channel, subfamily H (eag-related), member 2	NM_053949	1.61	4	210036_s_at	AB044806	2.44	14	1449544_a_at	NM_013569	2.737	15
**KIF5B**		1372444_at	kinesin family member 5B	AW918352	2.62	6	201992_s_at	NM_004521	1.46	10	1418431_at	BI328541	4.809	17
**KIF5B**		1372444_at	kinesin family member 5B	AW918352	2.62	6	201991_s_at	BF223224	1.28	8	1418429_at	BI328541	2.029	13
**KIF5C**	A	1373977_at	Kinesin family member 5C (predicted)	BE108253	5.73	2	203130_s_at	NM_004522	7.19	8	1457255_x_at	BB099427	8.989	17
**KIF5C**	A	1373977_at	Kinesin family member 5C (predicted)	BE108253	5.73	2	203129_s_at	BF059313	6.16	8	1422945_a_at	AI844677	4.754	17
**KIF5C**	A	1373977_at	Kinesin family member 5C (predicted)	BE108253	5.73	2	203129_s_at	BF059313	6.16	8	1450804_at	AI844677	1.783	17
**LEPR**		1369739_at	leptin receptor	D85558	1.41	6	209894_at	U50748	2.22	10	1425873_a_at	U58861	2.541	17
**LEPR**		1369739_at	leptin receptor	D85558	1.41	6	211354_s_at	U52913	1.42	8	1425873_a_at	U58861	2.541	17
**LEPR**		1369739_at	leptin receptor	D85558	1.41	6	211356_x_at	U66495	1.33	8	1425873_a_at	U58861	2.541	17
**LEPR**		1369739_at	leptin receptor	D85558	1.41	6	211355_x_at	U52914	1.31	8	1425873_a_at	U58861	2.541	17
**LMAN1**	A	1368848_at	lectin, mannose-binding, 1	NM_053886	3.9	6	203293_s_at	NM_005570	1.84	14	1428130_at	BG071597	5.81	17
**LMAN1**	A	1368848_at	lectin, mannose-binding, 1	NM_053886	3.9	6	203293_s_at	NM_005570	1.84	14	1428129_at	BG071597	4.626	17
**LMAN1**	A	1368848_at	lectin, mannose-binding, 1	NM_053886	3.9	6	203293_s_at	NM_005570	1.84	14	1452671_s_at	BG071597	3.926	17
**LMAN2**		1373815_at	lectin, mannose-binding 2 (predicted)	BI304064	1.49	4	200805_at	NM_006816	1.68	10	1423074_at	AK004952	1.527	13
**LNPEP**		1370743_a_at	leucyl/cystinyl aminopeptidase	AY079189	1.7	6	207904_s_at	NM_005575	1.1	12	1438432_at	BE850004	1.948	17
**LONP1**		1375802_at	lon peptidase 1, mitochondrial (Protease, serine, 15)	AI011610	1.69	6	209017_s_at	U02389	1.38	14	1428365_a_at	AK004820	2.054	17
**LRBA**		1374495_at	LPS-responsive beige-like anchor (predicted)	BM388121	2.57	6	212692_s_at	W60686	1.15	10	1436690_at	BB494139	3.505	17
**LRBA**		1374495_at	LPS-responsive beige-like anchor (predicted)	BM388121	2.57	6	212692_s_at	W60686	1.15	10	1425717_at	AF188506	2.313	17
**LRBA**		1374495_at	LPS-responsive beige-like anchor (predicted)	BM388121	2.57	6	214109_at	AI659561	1.18	10	1449099_at	NM_030695	1.941	13
**LRP12**		1374108_at	low density lipoprotein-related protein 12 (predicted)	BM388942	1.43	4	220254_at	NM_013437	9.99	10	1433864_at	AV254798	2.807	17

**LRP12**		1374108_at	low density lipoprotein-related protein 12 (predicted)	BM388942	1.43	4	220253_s_at	NM_013437	2.71	12	1433864_at	AV254798	2.807	17
**LRP12**		1374108_at	low density lipoprotein-related protein 12 (predicted)	BM388942	1.43	4	219631_at	NM_024937	2.19	12	1433864_at	AV254798	2.807	17
**LRRC8D**		1373431_at	leucine rich repeat containing 8 family, member D (alias: leucine rich re	AI101427	1.77	4	218684_at	NM_018103	1.31	12	1447491_at	BE987722	2.488	17
**LRRC8D**		1373431_at	leucine rich repeat containing 8 family, member D (alias: leucine rich re	AI101427	1.77	4	218684_at	NM_018103	1.31	12	1447491_at	BB315861	1.996	15
**MAGED1**		1367744_at	melanoma antigen, family D, 2	NM_080479	2.83	2	208682_s_at	AF126181	2.23	14	1426306_a_at	AF319976	1.534	13
**MAGED1**		1367744_at	melanoma antigen, family D, 2	NM_080479	2.83	2	213627_at	AI924630	1.41	10	1426306_a_at	AF319976	1.534	13
**MAGED2**		1386895_at	melanoma antigen, family D, 1	NM_053409	4.44	6	209014_at	AF217963	1.71	8	1450062_a_at	NM_019791	3.699	17
**MAP1B**	A	1371003_at	microtubule-associated protein 1b	BG378086	1.51	2	212233_at	AL523076	4.6	8	1450397_at	BB731480	11.28	17
**MAP1B**	A	1371003_at	microtubule-associated protein 1b	BG378086	1.51	2	212233_at	AL523076	4.6	8	1421851_at	BB731480	6.253	17
**MAP1B**	A	1371003_at	microtubule-associated protein 1b	BG378086	1.51	2	212233_at	AL523076	4.6	8	1421850_at	BB731480	2.08	13
**MAP1B**	A	1373363_at	microtubule-associated protein 1b	BI281702	2.39	0	214577_at	BG164365	1.93	8	1444595_at	BF148767	2.656	17
**MAP2**		1388152_at	microtubule-associated protein 2	BG374290	20.5	2	210015_s_at	U89330	2.84	10	1443239_at	BB386302	2.065	13
**MAP2**		1368411_a_a	microtubule-associated protein 2	X74211	1.84	4	210015_s_at	U89330	2.84	10	1443239_at	BB386302	2.065	13
**MBNL2**	A	1375140_at	muscleblind-like 2 (predicted)	AA818999	1.52	2	203640_at	BE328496	1.61	10	1436858_at	BB003847	1.557	7
**MED6**	C	1372269_at	mediator of RNA polymerase II transcription, subunit 6 homolog (yeast	AI229534	2.36	6	210104_at	AF074723	1.24	12	1458343_x_at	AV070715	1.543	17
**MFAP3**		1390063_at	microfibrillar-associated protein 3	BG380473	2.5	6	214588_s_at	R38475	1.22	10	1434562_at	AW536327	6.121	17
**MINPP1**		1388126_at	multiple inositol polyphosphate histidine phosphatase 1	BG380493	2.17	4	209585_s_at	AF084943	2.83	14	1455787_x_at	AV339366	3.048	17
**MLX**		1371929_at	MAX-like protein X (transcription factor-like 4) (predicted)	BI278563	1.74	6	217909_s_at	BF056105	1.23	10	1418437_a_at	NM_011550	3.64	17
**MYO1D**	B	1388917_at	myosin ID	BI279786	3.86	6	212338_at	AA621962	7.66	14	1458550_at	BB200258	2.347	17
**MYO1D**	B	1369779_at	myosin ID	NM_012983	1.71	6	212338_at	AA621962	7.66	14	1458550_at	BB200258	2.347	17
**MYO5A**		1368450_at	myosin Va	NM_022178	1.75	4	204527_at	NM_000259	1.31	10	1419754_at	NM_010864	1.5	13
**NAP1L1**	C	1371872_at	Nucleosome assembly protein 1-like 1	BE108905	1.36	0	204528_s_at	NM_004537	1.87	10	1420477_at	BG064031	1.73	13
**NAP1L1**	C	1371872_at	Nucleosome assembly protein 1-like 1	BE108905	1.36	0	213864_s_at	AI985751	1.81	10	1420477_at	BG064031	1.73	13
**NAP1L1**	C	1371872_at	Nucleosome assembly protein 1-like 1	BE108905	1.36	0	212967_x_at	AW148801	1.6	10	1420477_at	BG064031	1.73	13
**NAP1L1**	C	1371872_at	Nucleosome assembly protein 1-like 1	BE108905	1.36	0	208752_x_at	AI888672	1.55	12	1420477_at	BG064031	1.73	13
**NCALD**	A	1372953_at	neurocalcin delta	AI101330	3.49	2	211685_s_at	AF251061	1.9	8	1417568_at	BG071381	1.688	11
**NCBP2**		1383965_at	nuclear cap binding protein subunit 2 (predicted)	BI282103	2.73	2	201517_at	BC001255	1.23	10	1423046_s_at	BE285362	1.859	17
**NCBP2**		1383965_at	nuclear cap binding protein subunit 2 (predicted)	BI282103	2.73	2	201521_s_at	NM_007362	1.13	10	1423046_s_at	BE285362	1.859	17
**NEFM**	A	1367845_at	neurofilament 3, medium	NM_017029	3.27	2	205113_at	NM_005382	10.8	10	1457015_at	BB258427	3.819	15
**NEK1**		1389996_at	NIMA (never in mitosis gene a)-related expressed kinase 1 (predicted)	AI406369	1.48	4	213331_s_at	AV700007	2.02	12	1453612_at	AV254337	5.595	17
**NEK1**		1389996_at	NIMA (never in mitosis gene a)-related expressed kinase 1 (predicted)	AI406369	1.48	4	213331_s_at	AV700007	2.02	12	1434267_at	BG069735	1.783	13
**NEO1**		1376786_a_a	Neogenin	BF397709	2.79	6	204321_at	NM_002499	1.59	12	1447693_s_at	BB350308	2.818	17
**NEUROD1**	A	1394135_at	Neurogenic differentiation 1	AI639109	14.7	6	206282_at	NM_002500	21	12	1426413_at	BM116592	4.207	13
**NEUROD1**	A	1387288_at	neurogenic differentiation 1	NM_019218	7.09	6	206282_at	NM_002500	21	12	1426412_at	BM116592	3.089	13
**NFIC**		1371176_at	nuclear factor I/C	AB012233	1.35	4	206929_s_at	NM_005597	1.19	10	1450661_x_at	NM_008688	2.342	17
**NKX2-2**	B	1385387_at	NK2 transcription factor related, locus 2 (Drosophila) (predicted)	not on RG230A array			206915_at	NM_002509	43.8	14	1421112_at	NM_010919	40.65	17
**NKX6-1**	B	1368998_at	NK6 transcription factor related, locus 1 (Drosophila)	NM_031737	18.8	6	221366_at	NM_006168	2.51	14	1425828_at	AF357883	79.27	17
**NPTX2**	B		neuronal pentraxin II	not on RG230.20 array			213479_at	U26662	32.3	14	1420720_at	NM_016789	2.606	13
**NPY**		1387154_at	neuropeptide Y	NM_012614	2.12	6	206001_at	NM_000905	1.93	10	1419127_at	NM_023456	1.489	9
**NUCB2**	C	1370000_at	nucleobindin 2	NM_021663	6.77	6	203675_at	NM_005013	6.34	14	1418355_at	NM_016773	6.486	17
**NUP98**	C	1368747_at	nucleoporin 98	NM_031074	1.43	4	210793_s_at	U41815	1.67	10	1439154_at	BB473634	1.954	17
**P2RY1**		1370606_at	purinergic receptor P2Y, G-protein coupled 1	U22830	19.9	6	207455_at	NM_002563	1.17	10	1421456_at	NM_008772	7.131	17
**PAFAH1B1**	A	1369641_at	platelet-activating factor acetylhydrolase, isoform 1b, alpha2 subunit	BM392366	1.58	6	200816_s_at	NM_000430	1.68	8	1422793_at	BB539054	2.035	13
**PAFAH1B1**	A	1369642_at	platelet-activating factor acetylhydrolase, isoform 1b, alpha2 subunit	BM392366	1.45	0	200816_s_at	NM_000430	1.68	8	1422793_at	BB539054	2.035	13
**PAM**	B	1370502_at	peptidylglycine alpha-amidating monooxygenase	AW534339	3.73	6	214620_x_at	BF038548	6.34	14	1418908_at	NM_013626	2.265	15
**PAM**	B	1367687_a_a	peptidylglycine alpha-amidating monooxygenase	AW534339	3.77	6	202336_s_at	NM_000919	5.59	14	1418908_at	NM_013626	2.265	15
**PAM**	B	1367687_a_a	peptidylglycine alpha-amidating monooxygenase	AW534339	3.77	6	212958_x_at	AI022882	5.36	14	1418908_at	NM_013626	2.265	15
**PAPSS2**	B	1376047_at	3'-phosphoadenosine 5'-phosphosulfate synthase 2 (predicted)	BI285321	5.84	6	203058_s_at	AW299958	10.3	14	1421987_at	BF786072	7.396	17
**PAPSS2**	B	1376047_at	3'-phosphoadenosine 5'-phosphosulfate synthase 2 (predicted)	BI285321	5.84	6	203060_s_at	AF074331	8.86	14	1434510_at	BF780807	3.808	15
**PAPSS2**	B	1376047_at	3'-phosphoadenosine 5'-phosphosulfate synthase 2 (predicted)	BI285321	5.84	6	203059_s_at	NM_004670	4.08	14	1421989_s_at	BF786072	5.349	17

**PAX6**	B	1369242_at	paired box gene 6	NM_013001	15.4	4	205646_s_at	NM_000280	17.4	14	1452526_a_at	AF443223	9.312	15
**PAX6**	B	1369242_at	paired box gene 6	NM_013001	15.4	4	205646_s_at	NM_000280	17.4	14	1419271_at	BC011272	6.937	15
**PAX6**	B	1369242_at	paired box gene 6	NM_013001	15.4	4	205646_s_at	NM_000280	17.4	14	1425960_s_at	AF457142	4.103	17
**PCLO**	A	1387399_at	piccolo (presynaptic cytomatrix protein)	AF138789	1.43	6	213558_at	AB011131	2.69	8	1452423_at	AW493746	6.142	17
**PCLO**	A	1387399_at	piccolo (presynaptic cytomatrix protein)	AF138789	1.43	6	210650_s_at	BC001304	1.64	8	1419392_at	NM_011995	4.19	15
**PCSK1**	B	1368559_at	proprotein convertase subtilisin/kexin type 1	M83745	22.3	6	205825_at	NM_000439	17.3	14	1421396_at	M69196	28.62	17
**PCSK1**	B	1387247_at	proprotein convertase subtilisin/kexin type 1	M83745	21.6	6	205825_at	NM_000439	17.3	14	1421396_at	M69196	28.62	17
**PCSK2**	B	1387155_at	proprotein convertase subtilisin/kexin type 2	NM_012746	15.5	4	204869_at	AL031664	7.31	14	1447991_at	AI839700	44.29	17
**PCSK2**	B	1387155_at	proprotein convertase subtilisin/kexin type 2	NM_012746	15.5	4	204870_s_at	NM_002594	15.4	14	1448312_at	NM_008792	17.11	17
**PCSK2**	B	1387155_at	proprotein convertase subtilisin/kexin type 2	NM_012746	15.5	4	204869_at	AL031664	7.31	14	1447992_s_at	AI839700	24.52	17
**PCSK2**	B	1387155_at	proprotein convertase subtilisin/kexin type 2	NM_012746	15.5	4	204869_at	AL031664	7.31	14	1428305_at	AK018159	21.61	17
**PCSK2**	B	1387155_at	proprotein convertase subtilisin/kexin type 2	NM_012746	15.5	4	204869_at	AL031664	7.31	14	1444147_at	BB357975	5.453	17
**PDGFRL**		1374616_at	platelet-derived growth factor receptor-like (predicted)	BM384311	1.35	4	205226_at	NM_006207	2.04	8	1428896_at	AK004179	2.982	15
**PDIA3 / GF**	C	1398788_at	protein disulfide isomerase associated 3 (glucose regulated protein, 5	NM_017319	2.67	6	208612_at	D83485	2.16	14	1445330_at	BG916492	2.195	17
**PDIA6**	C	1370859_at	protein disulfide isomerase associated 6 (thioredoxin domain containin	BI284965	2.79	6	216640_s_at	AK026926	2.31	14	1423648_at		3.716	17
**PDIA6**	C	1370859_at	protein disulfide isomerase associated 6 (thioredoxin domain containin	BI284965	2.79	6	207668_x_at	NM_005742	2.31	14	1423648_at	0	3.716	17
**PDIA6**	C	1370859_at	protein disulfide isomerase associated 6 (thioredoxin domain containin	BI284965	2.79	6	208639_x_at	BC001312	2.26	14	1423648_at	0	3.716	17
**PDIA6**	C	1370859_at	protein disulfide isomerase associated 6 (thioredoxin domain containin	BI284965	2.79	6	208638_at	BE910010	1.75	14	1423648_at	0	3.716	17
**PDX1**		1369516_at	pancreatic and duodenal homeobox gene 1	NM_022852	3.42	6	210937_s_at	U35632	1.18	10	1422174_at	AK020261	47.04	17
**PDX1**		1369516_at	pancreatic and duodenal homeobox gene 1	NM_022852	3.42	6	210937_s_at	U35632	1.18	10	1422173_at	AK020261	12.43	17
**PFKFB2**	B	1388044_at	6-phosphofructo-2-kinase/fructose-2,6-biphosphatase 2	L27084	4.33	6	209992_at	AB044805	2.26	14	1422092_at	BC018418	9.607	17
**PFKFB2**	B	1369400_a_a	6-phosphofructo-2-kinase/fructose-2,6-biphosphatase 2	NM_080477	1.73	6	207931_s_at	NM_006212	1.36	12	1422090_a_at	BC018418	5.204	17
**PFKFB2**	B	1369400_a_a	6-phosphofructo-2-kinase/fructose-2,6-biphosphatase 2	NM_080477	1.73	6	207931_s_at	NM_006212	1.36	12	1422091_at	BC018418	3.504	15
**PGCP**		1368399_a_a	plasma glutamate carboxypeptidase	NM_031640	1.82	2	208454_s_at	NM_016134	1.5	14	1416441_at	BB468025	3.014	17
**PGCP**		1368399_a_a	plasma glutamate carboxypeptidase	NM_031640	1.82	2	203501_at	NM_006102	1.3	10	1416441_at	BB468025	3.014	17
**PGRMC2**	C	1388531_at	progesterone receptor membrane component 2 (predicted)	BF283382	1.67	4	201701_s_at	NM_006320	2.21	12	1448233_at	BE630020	2.349	15
**PGRMC2**	C	1388531_at	progesterone receptor membrane component 2 (predicted)	BF283382	1.67	4	213227_at	BE879873	1.77	14	1452882_at	BF322962	2.126	17
**PICALM**		1369452_a_a	phosphatidylinositol binding clathrin assembly protein	NM_053554	1.4	6	215832_x_at	AV722190	1.48	14	1443218_at	BB206107	2.015	17
**PITX3**		1370222_at	paired-like homeodomain transcription factor 3	AI145840	1.53	4	208277_at	NM_005029	1.31	12	1449917_at	NM_008852	2.733	17
**PJA2**		1375421_a_a	praja 2, RING-H2 motif containing	AI600019	1.93	2	201133_s_at	AA142966	1.43	10	1424442_a_at	BC017130	3.494	17
**PJA2**	B	1387903_at	praja 2, RING-H2 motif containing	D32249	1.4	2	201133_s_at	AA142966	1.43	10	1424442_a_at	BC017130	3.494	17
**PLCB4**	B	1386962_at	phospholipase C, beta 4	NM_024353	1.54	-2	203895_at	AL535113	2.63	12	1421987_at	BF786072	7.396	17
**POFUT2**	C	1390590_at	protein O-fucosyltransferase 2 (predicted)	AA965207	1.68	6	209578_s_at	BC000626	1.6	12	1435056_x_at	BB699319	2.464	15
**POFUT2**	C	1388717_at	protein O-fucosyltransferase 2 (predicted)	BM384163	1.7	6	209578_s_at	BC000626	1.6	12	1416573_at	BC018194	1.854	17
**PPP1R1A**	B	1386968_at	protein phosphatase 1, regulatory (inhibitor) subunit 1A	NM_022676	10	6	205478_at	NM_006741	8.52	12	1422605_at	NM_021391	4.05	13
**PPP2R1B**		1373959_at	protein phosphatase 2 (formerly 2A), regulatory subunit A (PR 65), bet	AI411788	1.85	2	202886_s_at	M65254	2.2	14	1428265_at	AK010754	1.682	15
**PPP2R1B**		1373959_at	protein phosphatase 2 (formerly 2A), regulatory subunit A (PR 65), bet	AI411788	1.85	2	202884_s_at	NM_002716	1.46	10	1428265_at	AK010754	1.682	15
**PRKACB**	C	1370746_at	protein kinase, cAMP dependent, catalytic, beta (predicted)	D10770	2.23	2	202741_at	AA130247	2.91	8	1420610_at	AV024339	3.263	15
**PRKACB**	C	1371835_at	protein kinase, cAMP dependent, catalytic, beta (predicted)	AI411907	1.7	0	202742_s_at	NM_002731	3.24	10	1420611_at	AV024339	2.209	15
**PRKAR1A**	C	1386905_at	protein kinase, cAMP dependent regulatory, type I, alpha	NM_013181	1.57	4	200604_s_at	M18468	2.42	10	1447635_at	BB410911	3.689	17
**PRKAR1A**	C	1386905_at	protein kinase, cAMP dependent regulatory, type I, alpha	NM_013181	1.57	4	200605_s_at	NM_002734	1.78	10	1425550_a_at	BC005697	1.875	15
**PRNP**	A	1370156_at	prion protein	BI278802	6.31	6	215707_s_at	AV725328	1.21	8	1416130_at	BE630020	2.294	13
**PRPS1**	C	1368165_at	phosphoribosyl pyrophosphate synthetase 1	M29392	1.75	6	208447_s_at	NM_002764	1.28	10	1416052_at	AK011304	2.404	17
**PRPS1**	C	1387085_at	phosphoribosyl pyrophosphate synthetase 1	M29392	1.82	6	209440_at	BC001605	1.85	10	1448192_s_at	AK011304	1.606	15
**PTK2**	A	1394338_x_a	PTK2 protein tyrosine kinase 2	BF290004	1.35	6	208820_at	AL037339	1.48	10	1430827_a_at	AV301702	2.484	17
**PTK2**	A	1394338_x_a	PTK2 protein tyrosine kinase 2	BF290004	1.35	6	207821_s_at	NM_005607	1.21	10	1430827_a_at	AV301702	2.484	17
**PTPRF**		1368035_a_a	protein tyrosine phosphatase, receptor type, F	X83505	3.23	2	200635_s_at	AU145351	1.36	8	1420842_at	BF235516	2.247	15
**PTPRF**		1368035_a_a	protein tyrosine phosphatase, receptor type, F	X83505	3.23	2	200635_s_at	AU145351	1.36	8	1420841_at	BF235516	1.817	13
**PTPRF**		1368036_at	protein tyrosine phosphatase, receptor type, F	M60103	1.79	4	200637_s_at	AI762627	1.68	12	1420843_at	BF235516	2.462	15
**PTPRN**		1370362_at	protein tyrosine phosphatase, receptor type, N	D45414	4.6	6	204945_at	NM_002846	8.66	14	1416588_at	NM_008985	5.366	15

**PTPRN2**	A	1370182_at	protein tyrosine phosphatase, receptor type, N polypeptide 2	AI574884	12.5	6	203029_s_at	NM_002847	7	14	1425724_at	U57345	1.428	17
**PTPRN2**	A	1370182_at	protein tyrosine phosphatase, receptor type, N polypeptide 2	AI574884	12.5	6	203030_s_at	AF007555	5.32	14	1425724_at	U57345	1.428	17
**PTPRN2**	A	1370182_at	protein tyrosine phosphatase, receptor type, N polypeptide 2	AI574884	12.5	6	211534_x_at	U65065	1.44	12	1425724_at	U57345	1.428	17
**RAB14**	C	1367884_at	RAB14, member RAS oncogene family	NM_053589	1.36	0	211503_s_at	AF112206	1.26	10	1419245_at	AV339290	2.42	17
**RAB1A**	C	1398841_at	RAB1, member RAS oncogene family	AI411122	1.62	6	207791_s_at	NM_004161	1.95	12	1416082_at	AW108405	1.627	15
**RAB1A**	C	1373022_at	RAB1, member RAS oncogene family	AI231860	1.94	4	208724_s_at	BC000905	1.37	14	1416082_at	AW108405	1.627	15
**RAB2A**	B	1370087_at	RAB2, member RAS oncogene family	NM_031718	1.42	6	208731_at	AU158062	1.82	10	1418623_at	NM_021518	1.91	15
**RAB2A**	B	1370087_at	RAB2, member RAS oncogene family	NM_031718	1.42	6	208732_at	AI743756	1.34	8	1418623_at	NM_021518	1.91	15
**RAB2A**	B	1370087_at	RAB2, member RAS oncogene family	NM_031718	1.42	6	208733_at	AW301641	1.31	10	1418623_at	NM_021518	1.91	15
**RAB2A**	B	1370087_at	RAB2, member RAS oncogene family	NM_031718	1.42	6	208734_x_at	M28213	1.62	14	1419946_s_at	C80220	1.566	15
**RAB3B**		1370061_at	RAB3B, member RAS oncogene family	NM_031091	1.94	4	205924_at	BC005035	4.64	14	1422583_at	NM_023537	2.52	13
**RAB3B**		1370061_at	RAB3B, member RAS oncogene family	NM_031091	1.94	4	205925_s_at	NM_002867	2.63	14	1422583_at	NM_023537	2.52	13
**RANBP2**		1375371_at	similar to Ran-binding protein 2 (predicted)	BI296662	1.39	4	201713_s_at	D42063	2.44	14	1440104_at	BB208180	5.203	17
**RANBP2**		1375371_at	similar to Ran-binding protein 2 (predicted)	BI296662	1.39	4	201711_x_at	AI681120	1.62	8	1440104_at	BB208180	5.203	17
**RANBP2**		1375371_at	similar to Ran-binding protein 2 (predicted)	BI296662	1.39	4	201712_s_at	NM_006267	1.36	14	1440104_at	BB208180	5.203	17
**RBBP7**	C	1398768_at	retinoblastoma binding protein 7	NM_031816	1.45	4	201092_at	NM_002893	1.44	10	1439131_at	BB022577	2.616	17
**RBM39**	C	1398993_at	RNA-binding region (RNP1, RRM) containing 2 (predicted)	AI045458	1.63	2	208720_s_at	AI890947	1.24	10	1438398_at	BB203348	3.594	17
**RBM39**	C	1399101_at	RNA-binding region (RNP1, RRM) containing 2 (predicted)	AA849715	1.51	2	208720_s_at	AI890947	1.24	10	1456386_at	BB203348	2.437	15
**RBM39**	C	1381967_at	RNA-binding region (RNP1, RRM) containing 2 (predicted)	BE114972	1.48	2	208720_s_at	AI890947	1.24	10	1438420_at	AV019076	1.886	13
**RBM39**	C	1379737_a_at	RNA-binding region (RNP1, RRM) containing 2 (predicted)	BG372903	1.44	2	208720_s_at	AI890947	1.24	10	1438397_a_at	BB203348	1.785	15
**RGS4**	A	1368505_at	regulator of G-protein signaling 4	U27767	5.5	6	204338_s_at	NM_005613	7.48	12	1416287_at	NM_009062	8.283	15
**RGS4**	A	1368505_at	regulator of G-protein signaling 4	U27767	5.5	6	204339_s_at	BC000737	5.6	12	1416286_at	NM_009062	9.081	15
**RGS4**	A	1368506_at	regulator of G-protein signaling 4	U27767	5.22	6	204337_at	AL514445	9.12	12	1448285_at	NM_009062	3.413	15
**ROBO1**	A	1383767_at	Roundabout homolog 1 (Drosophila)	AW524430	1.4	6	213194_at	BF059159	2.32	12	1427231_at	BG065230	2.391	11
**RPH3AL**	B	1387176_at	rabphilin 3A-like (without C2 domains)	NM_133591	5.17	4	221614_s_at	BC005153	1.69	14	1431695_at	AK018335	2.094	13
**RRAGA**	C	1367736_at	Ras-related GTP binding A	NM_053973	1.71	6	201628_s_at	NM_006570	1.33	8	1428905_at	AI118026	1.591	15
**RRAGD**	B	1373427_at	Ras-related GTP binding D (predicted)	BI288816	1.41	2	221523_s_at	AL138717	5.61	12	1431164_at	AK017818	3.236	17
**RRAGD**	B	1373427_at	Ras-related GTP binding D (predicted)	BI288816	1.41	2	221524_s_at	AF272036	2.98	12	1431164_at	AK017818	3.236	17
**SC4MOL**		1368275_at	sterol-C4-methyl oxidase-like	NM_080886	2.24	4	209146_at	AV704962	2.23	8	1459627_at	BG070057	3.978	17
**SCFD1**	C	1368158_at	sec1 family domain containing 1	NM_019364	2.59	6	215548_s_at	AB020724	1.99	14	1428335_a_at	AK014070	1.852	15
**SCG2**	B	1368044_at	secretogranin 2	NM_022669	67.3	4	204035_at	NM_003469	29	14	1450708_at	NM_009129	22.92	17
**SCG3**	B	1368656_at	secretogranin III	NM_053856	31.6	4	219196_at	NM_013243	53.4	14	1448628_at	NM_009130	14.54	17
**SCG5**	B	1367992_at	secretory granule neuroendocrine protein 1	NM_013175	19.4	4	203889_at	NM_003020	24.8	14	1423150_at	AK019337	6.412	15
**SCN3A**	A	1369691_at	sodium channel, voltage-gated, type III, alpha polypeptide /// hypotheti	NM_013119	2.31	4	210432_s_at	AF225986	2.48	8	1439204_at	BB096886	2.978	13
**SEC22B**		1389138_at	SEC22 vesicle trafficking protein-like 1 (S. cerevisiae)	AA945574	2.51	6	209207_s_at	BC001364	1.83	14	1449063_at	BC009024	2.326	17
**SEC22B**		1389138_at	SEC22 vesicle trafficking protein-like 1 (S. cerevisiae)	AA945574	2.51	6	209206_at	AV701283	1.56	12	1449063_at	BC009024	2.326	17
**SEC24D**	C	1373610_at	SEC24 related gene family, member D (S. cerevisiae) (predicted)	AI232694	6.26	6	202375_at	NM_014822	4.46	14	1426972_at	AK009425	4.549	17
**SEC61B**	C	1382615_at	Sec61 alpha 1 subunit (S. cerevisiae)	BI284366	2.67	6	217716_s_at	NM_013336	1.53	12	1416190_a_at	BC003707	7.606	15
**SEC61B**	C	1382615_at	Sec61 alpha 1 subunit (S. cerevisiae)	BI284366	2.67	6	217716_s_at	NM_013336	1.53	12	1416191_at	BC003707	5.271	15
**SEC61B**	C	1371368_at	Sec61 alpha 1 subunit (S. cerevisiae)	BI279499	3.81	6	217716_s_at	NM_013336	1.53	12	1434986_a_at	AI327023	1.852	15
**SEC61B**	C	1382615_at	Sec61 alpha 1 subunit (S. cerevisiae)	BI284366	2.67	6	217716_s_at	NM_013336	1.53	12	1416189_a_at	BC003707	2.089	15
**SEC61B**	C	1388519_at	Sec61 beta subunit (predicted)	BI281906	2.66	6	203133_at	NM_006808	1.54	14	1417083_at	NM_024171	1.563	15
**SEC63**	C	1390115_at	SEC63-like (S. cerevisiae) (predicted)	BM384667	1.86	6	201916_s_at	NM_007214	1.73	12	1424926_at	C76103	4.32	17
**SEC63**	C	1390115_at	SEC63-like (S. cerevisiae) (predicted)	BM384667	1.86	6	201914_s_at	AK001465	1.59	12	1424925_at	C76103	2.988	17
**SEC63**	C	1390115_at	SEC63-like (S. cerevisiae) (predicted)	BM384667	1.86	6	201914_s_at	AK001465	1.59	12	1424924_at	C76103	1.778	13
**SEPT2**	C	1388389_at	septin 2	BF282620	1.54	4	200015_s_at	NM_004404	1.32	10	1423473_at	AV304911	1.705	17
**SEPT2**	C	1368984_at	septin 2	NM_057148	1.92	6	200778_s_at	AI191427	1.61	14	1423473_at	AV304911	1.705	17
**SEZ6L2**		1374825_at	seizure related 6 homolog (mouse)-like 2 (predicted)	BG380338	2.24	6	218720_x_at	NM_012410	1.24	8	1434641_x_at	AW121511	6.872	15
**SEZ6L2**		1374825_at	seizure related 6 homolog (mouse)-like 2 (predicted)	BG380338	2.24	6	218720_x_at	NM_012410	1.24	8	1423983_at	BC011475	5.546	15
**SFRS10**	C	1370189_at	splicing factor, arginine/serine-rich 10 (transformer 2 homolog, Drosop	BI290979	1.66	4	200892_s_at	BC000451	1.21	8	1446496_at	BB219075	1.782	17

**SFRS10**	C	1370188_at	splicing factor, arginine/serine-rich 10 (transformer 2 homolog, Drosop	BI290979	1.79	4	200892_s_at	BC000451	1.21	8	1441466_at	BG066615	1.508	15
**SLC18A1**	A	1387999_at	solute carrier family 18 (vesicular monoamine), member 1	M97380	1.4	6	207074_s_at	NM_003053	1.17	10	1458777_at	BG068849	7.459	17
**SLC2A2**		1387228_at	solute carrier family 2 (facilitated glucose transporter), member 2	NM_012879	28.6	6	206535_at	NM_000340	5.47	8	1449067_at	NM_031197	4.311	13
**SLC35B1**	B	1377143_at	solute carrier family 35, member B1	BM388792	2.42	6	202433_at	NM_005827	1.45	14	1448769_at	NM_016752	2.512	17
**SLC7A2**		1369460_at	solute carrier family 7 (cationic amino acid transporter, y+ system), me	NM_022619	2.35	4	207626_s_at	NM_003046	1.62	12	1426008_a_at	M62838	3.764	15
**SLC7A2**		1369460_at	solute carrier family 7 (cationic amino acid transporter, y+ system), me	NM_022619	2.35	4	207626_s_at	NM_003046	1.62	12	1422648_at	BF533509	3.524	15
**SLC7A2**		1369460_at	solute carrier family 7 (cationic amino acid transporter, y+ system), me	NM_022619	2.35	4	207626_s_at	NM_003046	1.62	12	1440506_at	BB433596	1.831	15
**SLC7A8**	B	1387057_at	solute carrier family 7 (cationic amino acid transporter, y+ system), me	NM_053442	4.59	4	217248_s_at	AL365343	2.56	12	1417929_at	NM_016972	2.66	17
**SLC7A8**	B	1387057_at	solute carrier family 7 (cationic amino acid transporter, y+ system), me	NM_053442	4.59	4	202752_x_at	NM_012244	1.79	12	1417929_at	NM_016972	2.66	17
**SLC7A8**	B	1387057_at	solute carrier family 7 (cationic amino acid transporter, y+ system), me	NM_053442	4.59	4	216092_s_at	AL365347	1.65	12	1417929_at	NM_016972	2.66	17
**SMARCA2**	C	1376337_at	SWI/SNF related, matrix associated, actin dependent regulator of chro	AI170385	1.59	4	206542_s_at	AV725365	2.29	10	1452333_at	BM230202	1.781	17
**SMARCA2**	C	1376337_at	SWI/SNF related, matrix associated, actin dependent regulator of chro	AI170385	1.59	4	206544_x_at	NM_003070	2.27	12	1452333_at	BM230202	1.781	17
**SMARCA2**	C	1376337_at	SWI/SNF related, matrix associated, actin dependent regulator of chro	AI170385	1.59	4	217707_x_at	AI535683	1.58	10	1452333_at	BM230202	1.781	17
**SND1**	C	1367790_at	staphylococcal nuclease domain containing 1	NM_022694	2.05	6	201622_at	NM_014390	1.72	14	1437036_at	BB240087	5.873	17
**SND1**	C	1367790_at	staphylococcal nuclease domain containing 1	NM_022694	2.05	6	201622_at	NM_014390	1.72	14	1437037_x_at	BB240087	3.261	17
**SND1**	C	1367790_at	staphylococcal nuclease domain containing 1	NM_022694	2.05	6	201622_at	NM_014390	1.72	14	1416038_at	NM_019776	1.729	13
**SNRPN /// S**	A	1368098_a_at	small nuclear ribonucleoprotein N	M29294	1.56	2	206042_x_at	NM_022804	3.04	10	1415896_x_at		3.635	17
**SNRPN /// S**	A	1368098_a_at	small nuclear ribonucleoprotein N	M29294	1.56	2	221974_at	AW770748	2.61	10	1435716_x_at		1.064	9
**SOCS2**		1369577_at	suppressor of cytokine signaling 2	NM_058208	2.42	6	203372_s_at	AB004903	2.13	12	1449109_at	NM_007706	1.914	13
**SOCS2**		1369577_at	suppressor of cytokine signaling 2	NM_058208	2.42	6	203373_at	NM_003877	1.47	12	1449109_at	NM_007706	1.914	13
**SORL1**	C	1393933_at	sortilin-related receptor, L(DLR class) A repeats-containing (predicted)	AW144823	4.02	4	212560_at	AV728268	3.99	10	1445889_at	BE980129	1.997	17
**SORL1**	C	1377457_a_at	sortilin-related receptor, L(DLR class) A repeats-containing (predicted)	AA850618	3.38	4	212560_at	AV728268	3.99	10	1460390_at	BI648081	1.512	15
**SORL1**	C	1386269_x_at	sortilin-related receptor, L(DLR class) A repeats-containing (predicted)	AI177589	3.36	4	212560_at	AV728268	3.99	10	1460390_at	BI648081	1.512	15
**SORL1**	C	1390710_x_at	sortilin-related receptor, L(DLR class) A repeats-containing (predicted)	AA850618	2.02	4	212560_at	AV728268	3.99	10	1460390_at	BI648081	1.512	15
**SORL1**	C	1377458_at	sortilin-related receptor, L(DLR class) A repeats-containing (predicted)	AA850618	1.94	4	212560_at	AV728268	3.99	10	1460390_at	BI648081	1.512	15
**SQLE**		1387017_at	squalene epoxidase	NM_017136	1.81	-2	209218_at	AF098865	1.84	10	1415993_at	NM_009270	2.336	15
**SQLE**		1387017_at	squalene epoxidase	NM_017136	1.81	-2	213562_s_at	BF979497	1.49	8	1415993_at	NM_009270	2.336	15
**SQSTM1**	C	1375374_at	Sequestosome 1	BF400606	3	4	201471_s_at	NM_003900	2.23	14	1444021_at	AW741388	3.21	17
**SQSTM1**	C	1389381_at	sequestosome 1	AI411586	2.56	4	213112_s_at	N30649	1.33	14	1444021_at	AW741388	3.21	17
**SQSTM1**	C	1371353_at	sequestosome 1	BG663093	1.56	4	201471_s_at	NM_003900	2.23	14	1444021_at	AW741388	3.21	17
**SRP72**	C	1375552_at	signal recognition particle,72 kDa subunit	BG372976	3.25	6	208095_s_at	NM_001222	2.01	14	1428876_at	BM942773	2.247	17
**SRP72**	C	1375552_at	signal recognition particle,72 kDa subunit	BG372976	3.25	6	208800_at	AV702627	1.54	12	1428876_at	BM942773	2.247	17
**SRP72**	C	1375552_at	signal recognition particle,72 kDa subunit	BG372976	3.25	6	208801_at	BE856385	1.52	12	1428876_at	BM942773	2.247	17
**SRP72**	C	1375552_at	signal recognition particle,72 kDa subunit	BG372976	3.25	6	208802_at	AI493872	1.19	10	1428877_at	BM942773	1.931	17
**SRP9**	C	1372445_at	Signal recognition particle 9 kDa protein (SRP9)	AI171783	2.25	6	201273_s_at	NM_003133	1.3	10	1448753_at	BI661964	1.878	15
**SRPR**	C	1371343_at	signal recognition particle receptor ('docking protein') (predicted)	BM391232	2.71	6	200918_s_at	NM_003139	2.02	14	1423670_a_at	BC021839	3.12	17
**SRPR**	C	1371343_at	signal recognition particle receptor ('docking protein') (predicted)	BM391232	2.71	6	200917_s_at	BG474541	1.28	10	1423670_a_at	BC021839	3.12	17
**SSR1**	C	1373090_at	signal sequence receptor, alpha (predicted)	AI230697	2.5	6	200889_s_at	AI016620	2.67	14	1441327_a_at	AV325051	3.343	17
**SSR1**	C	1373090_at	signal sequence receptor, alpha (predicted)	AI230697	2.5	6	200890_s_at	AW006345	2.09	14	1441327_a_at	AV325051	3.343	17
**SSR1**	C	1373090_at	signal sequence receptor, alpha (predicted)	AI230697	2.5	6	200891_s_at	NM_003144	2.12	14	1448843_at	BG077348	2.228	13
**SSR2**	C	1373185_at	signal sequence receptor, beta (predicted)	BE329347	1.94	4	200652_at	NM_003145	1.49	8	1449930_a_at	NM_025448	1.555	13
**SSR4**	C	1367690_at	signal sequence receptor 4	NM_017199	2.49	6	201004_at	NM_006280	2.84	14	1427096_s_at	AV051818	2.458	17
**SSTR2**	A	1371174_s_at	somatostatin receptor 2	X98234	1.4	4	217455_s_at	AF184174	1.97	12	1422256_at	NM_009217	1.947	15
**ST18**	B	1370658_a_at	suppression of tumorigenicity 18	AF031942	4.87	6	206135_at	NM_014682	11.7	12	1455123_at	BB178719	22.07	17
**ST18**	B	1370658_a_at	suppression of tumorigenicity 18	AF031942	4.87	6	206135_at	NM_014682	11.7	12	1436793_at	AV347235	2.785	17
**STAU2**	A	1390558_at	staufen, RNA binding protein, homolog 2 (Drosophila)	AI502229	2.63	4	204226_at	NM_014393	1.27	8	1425533_a_at	AJ244015	2.576	17
**STAU2**	A	1390558_at	staufen, RNA binding protein, homolog 2 (Drosophila)	AI502229	2.63	4	204226_at	NM_014393	1.27	8	1450916_at	AI843206	2.557	15
**STCH**	C	1372368_at	Stress 70 protein chaperone, microsome-associated, 60kD human hor	AI230431	3.57	6	202558_s_at	NM_006948	2.72	14	1453172_at	BE533039	17.34	17
**STCH**	C	1368844_at	stress 70 protein chaperone, microsome-associated, 60kD human hor	NM_019271	2.42	6	202557_at	AI718418	2.62	12	1430026_at	AK021006	8.187	17
**STCH**	C	1368844_at	stress 70 protein chaperone, microsome-associated, 60kD human hor	NM_019271	2.42	6	202557_at	AI718418	2.62	12	1433772_at	BE650268	2.426	15

**STCH**	C	1368844_at	stress 70 protein chaperone, microsome-associated, 60kD human hom	NM_019271	2.42	6	202557_at	AI718418	2.62	12	1429502_at	BE533039	2.156	17
**STXBP1**	A	1370518_a_at	syntaxin binding protein 1	U06069	4.21	4	202260_s_at	NM_003165	5.62	8	1420506_a_at	AF326545	9.613	17
**STXBP1**	A	1398330_at	syntaxin binding protein 1	BM388715	2.12	4	202260_s_at	NM_003165	5.62	8	1420506_a_at	AF326545	9.613	17
**STXBP1**	A	1370519_at	syntaxin binding protein 1	U06069	2.74	4	202260_s_at	NM_003165	5.62	8	1420505_a_at	AF326545	2.406	13
**SYP**	A	1368276_at	synaptophysin	NM_012664	1.75	2	213200_at	U93305	2.59	8	1448280_at	NM_009305	1.691	13
**TAF9B**	B	1368429_at	TAF9-like RNA polymerase II, TATA box binding protein (TBP)-associa	NM_133615	1.49	2	221618_s_at	AF220509	1.44	12	1434960_at	AW555571	2.848	17
**TAF9B**	B	1368429_at	TAF9-like RNA polymerase II, TATA box binding protein (TBP)-associa	NM_133615	1.49	2	221618_s_at	AF220509	1.44	12	1447829_x_at	BB431174	2.221	17
**TARS**	C	1372145_at	threonyl-tRNA synthetase	BI289683	1.59	2	201263_at	NM_003191	2.24	12	1460323_at	AK010256	1.944	17
**TCTA**		1372507_at	T-cell leukemia translocation altered gene	AA901093	1.57	6	203054_s_at	NM_022171	1.33	14	1420123_at	C85065	2.286	17
**TERF2IP**		1388780_at	telomeric repeat binding factor 2, interacting protein (predicted)	BF395792	2.17	6	201174_s_at	NM_018975	2.12	8	1419381_at	NM_020584	1.675	13
**TGOLN2**		1371028_at	trans-golgi network protein 2	AW917192	2.32	6	212040_at	BG249599	1.94	14	1423307_s_at	AI314055	1.617	15
**TGOLN2**		1389965_at	trans-golgi network protein 2	AA799818	1.63	4	203833_s_at	BF061845	1.65	14	1423307_s_at	AI314055	1.617	15
**TGOLN2**		1387755_s_at	trans-golgi network protein 2	NM_138840	1.63	6	212043_at	W72053	1.57	12	1423307_s_at	AI314055	1.617	15
**TGOLN2**		1389965_at	trans-golgi network protein 2	AA799818	1.63	4	203834_s_at	NM_006464	1.23	10	1423307_s_at	AI314055	1.617	15
**TLE1**		1374425_at	transducin-like enhancer of split 1, homolog of Drosophila E(spl) (predi	BG380534	2.09	4	203220_s_at	AI951720	1.22	10	1422751_at	NM_011599	1.616	11
**TLOC1**	C	1374188_at	translocation protein 1 (predicted)	AI103194	1.47	6	208942_s_at	BE866511	1.79	12	1455698_at	BQ175470	1.495	7
**TLOC1**	C	1374188_at	translocation protein 1 (predicted)	AI103194	1.47	6	208943_s_at	U93239	1.59	14	1455698_at	BQ175470	1.495	7
**TM4SF4**	B	1368706_at	transmembrane 4 superfamily member 4	NM_053785	11.6	6	209937_at	BC001386	15.8	14	1424962_at	BC010814	9.872	17
**TM9SF4**		1371647_at	transmembrane protein TM9SF3 (predicted)	AA963367	1.58	6	212194_s_at	AI418892	1.53	14	1423204_at	BB367487	7.14	17
**TMOD2**		1369541_at	tropomodulin 2	NM_031613	1.66	4	219701_at	NM_014548	1.85	10	1430153_at	AK018223	11.38	17
**TMOD2**		1390482_at	Tropomodulin 2	BF285511	2.06	6	219701_at	NM_014548	1.85	10	1431326_a_at	AK017072	5.517	15
**TMOD2**		1393418_at	tropomodulin 2	BF567833	1.93	4	219701_at	NM_014548	1.85	10	1435060_at	BB667124	3.117	15
**TPD52**	B	1391880_at	Tumor protein D52 (predicted)	AA893670	1.65	2	201688_s_at	BG389015	5.99	14	1419493_a_at	BC002036	1.967	15
**TPD52**	B	1391880_at	Tumor protein D52 (predicted)	AA893670	1.65	2	201689_s_at	BE974098	3.95	14	1419493_a_at	BC002036	1.967	15
**TPD52**	B	1391880_at	Tumor protein D52 (predicted)	AA893670	1.65	2	201690_s_at	AA524023	3.92	14	1419493_a_at	BC002036	1.967	15
**TPD52**	B	1391880_at	Tumor protein D52 (predicted)	AA893670	1.65	2	201691_s_at	NM_005079	2.04	14	1419493_a_at	BC002036	1.967	15
**TRIB1**	C	1389987_at	tribbles homolog 1 (Drosophila)	AI511059	4.96	6	202241_at	NM_025195	3.48	10	1424881_at	AV237242	3.652	15
**TRIB1**	C	1371019_at	tribbles homolog 1 (Drosophila)	BM387324	2.08	4	202241_at	NM_025195	3.48	10	1424880_at	AV237242	4.079	15
**TSN**		1367728_at	translin	NM_021762	1.88	6	201513_at	AI659180	1.62	12	1448516_at	BB818311	2.941	17
**TSN**		1367728_at	translin	NM_021762	1.88	6	201504_s_at	AI435302	1.44	10	1448516_at	BB818311	2.941	17
**TSN**		1367728_at	translin	NM_021762	1.88	6	201515_s_at	NM_004622	1.35	12	1448516_at	BB818311	2.941	17
**TSPYL4**	A	1372381_at	TSPY-like 4	BM383457	1.84	0	212928_at	AL050331	1.69	8	1424029_at	BC017540	1.556	13
**TSPYL4**	A	1390097_at	TSPY-like 4 (predicted)	BI281738	1.92	0	212928_at	AL050331	1.69	8	1424029_at	BC017540	1.556	13
**TXNDC1**	C	1372067_at	thioredoxin domain containing 1 (predicted)	AI179567	1.91	2	208097_s_at	NM_030755	2.45	14	1437143_a_at	AV310544	2.14	15
**TXNDC1**	C	1372067_at	thioredoxin domain containing 1 (predicted)	AI179567	1.91	2	209476_at	AL080080	1.68	14	1437143_a_at	AV310544	2.14	15
**TXNDC4**	C	1372298_at	thioredoxin domain containing 4 (endoplasmic reticulum) (predicted)	BE098766	2.43	6	208959_s_at	BC005374	1.96	14	1423246_at	BI100077	3.851	17
**TXNL1**	C	1388862_at	Thioredoxin-like (32kD)	AI412143	1.47	4	201588_at	NM_004786	1.3	10	1435235_at	BI662855	2.469	17
**TXNL1**	C	1368936_at	thioredoxin-like (32kD)	NM_080887	1.44	6	201588_at	NM_004786	1.3	10	1435235_at	BI662855	2.469	17
**TXNL1**	C	1380191_s_at	thioredoxin-like (32kD)	BF555110	1.37	6	201588_at	NM_004786	1.3	10	1435235_at	BI662855	2.469	17
**TXNL1**	C	1391789_at	thioredoxin-like (32kD)	BF555110	1.89	6	201588_at	NM_004786	1.3	10	1437905_at	AV106191	1.883	13
**UBL3**	B	1374413_at	ubiquitin-like 3 (predicted)	BM386522	1.8	4	201534_s_at	AF044221	2.11	14	1423461_a_at	AV328436	1.907	15
**UBL3**	B	1374413_at	ubiquitin-like 3 (predicted)	BM386522	1.8	4	201535_at	NM_007106	1.48	8	1423461_a_at	AV328436	1.907	15
**UBQLN2**	C	1372131_at	ubiquilin 2 (predicted)	BF398140	1.57	2	215884_s_at	AK001029	1.86	10	1420844_at	AV171029	2.878	17
**UBXD2**		1388444_at	UBX domain containing 2 (predicted)	BI291633	1.81	6	212006_at	AU149908	2.07	14	1426485_at	AI788596	7.221	17
**UBXD2**		1388444_at	UBX domain containing 2 (predicted)	BI291633	1.81	6	212008_at	N29889	2.29	14	1444057_at	BB253461	1.784	13
**UFC1**	C	1371758_at	Ufm1-conjugating enzyme 1	BM986259	1.69	6	217797_at	NM_016406	1.73	14	1416327_at	NM_025388	2.16	17
**USP47**	C	1399036_at	ubiquitin specific protease 47 (predicted)	AA964374	1.53	4	221518_s_at	BE966019	1.21	8	1426976_at	BG069271	1.501	11
**VAT1**		1374905_at	Vesicle amine transport protein 1 homolog (T californica)	AA925303	1.57	6	208626_s_at	BC001913	1.82	14	1438165_x_at	BB559097	2.079	13
**VLDLR**		1369098_at	very low density lipoprotein receptor	NM_013155	2.13	4	209822_s_at	L22431	4.46	14	1438258_at	BE647363	1.907	11
**VLDLR**		1387455_a_at	very low density lipoprotein receptor	NM_013155	2.02	4	209822_s_at	L22431	4.46	14	1438258_at	BE647363	1.907	11

**WDR6**	C	1373399_at	WD repeat domain 6 (predicted)	BI291997	2.82	4	217734_s_at	NM_018031	1.62	10	1455940_x_at	BB453609	2.105	17
**WFS1**		1387356_at	Wolfram syndrome 1	NM_031823	3.45	6	202908_at	NM_006005	2.27	14	1448411_at	NM_011716	4.078	17
**WFS1**		1368839_at	Wolfram syndrome 1	NM_031823	3	6	202908_at	NM_006005	2.27	14	1448411_at	NM_011716	4.078	17
**WNT4**	B	1368641_at	wingless-related MMTV integration site 4	NM_053402	7.47	4	208606_s_at	NM_030761	15.1	14	1450782_at	NM_009523	3.741	15
**YWHAQ**	C	1370168_at	tyrosine 3-monooxygenase/tryptophan 5-monooxygenase activation pr	BF281342	1.72	-2	200693_at	NM_006826	1.75	10	1437608_x_at	BB414446	3.509	17
**YWHAQ**	C	1387862_at	tyrosine 3-monooxygenase/tryptophan 5-monooxygenase activation pr	BF281342	1.58	-2	212426_s_at	BF033313	1.95	10	1445341_at	BG074147	1.971	15
**ZNF451**	C	1389047_at	Zinc finger protein 451	AI009167	2.17	4	212557_at	AB011148	1.42	10	1456415_at	BI083675	2.433	17
**ZNF451**	C	1389047_at	Zinc finger protein 451	AI009167	2.17	4	212557_at	AB011148	1.42	10	1456350_at	BI714076	1.683	15

Islet-abundant and , in rat tissue data set, islet endocrine non-beta (alpha cell) -enriched

symbol		probe set	gene (Rat)	Accession	av FC	eta sco	probe set	Accession	av FC	eta sco	probe set	Accession	av FC	eta scor
**ANXA4**		1368908_at	annexin A4	NM_024155	1.45	4	201301_s_at	BC000182	3.74	10	1421223_a_at	NM_013471	1.689	9
**ANXA4**		1389305_at	annexin A4	BM385237	1.34	2	201302_at	NM_001153	3.69	8	1421223_a_at	NM_013471	1.689	9
**ALCAM**		1370043_at	activated leukocyte cell adhesion molecule	NM_031753	1.55	-2	201951_at	BF242905	9.6	14	1426301_at	U95030	2.038	15
**ALCAM**		1375263_at	Activated leukocyte cell adhesion molecule	BI296498	1.41	4	201952_at	AA156721	5.39	14	1426301_at	U95030	2.038	15
**ABCC8**		1369632_a_a	ATP-binding cassette, sub-family C (CFTR/MRP), member 8	AB052294	2.69	4	210246_s_at	AF087138	22.3	14	1457066_at	BF466569	9.954	17
**ABCC8**		1369632_a_a	ATP-binding cassette, sub-family C (CFTR/MRP), member 8	AB052294	2.69	4	210246_s_at	AF087138	22.3	14	1455765_a_at	BB515948	3.333	17
**BTG3**		1368072_at	B-cell translocation gene 3	NM_019290	5.1	4	213134_x_at	AI765445	6.48	14	1449007_at	NM_009770	2.914	17
**BTG3**		1368072_at	B-cell translocation gene 3	NM_019290	5.1	4	205548_s_at	NM_006806	5.44	14	1449007_at	NM_009770	2.914	17
**BCLAF1**		1373868_at	BCL2-associated transcription factor 1 (predicted)	AI717547	1.8	4	214499_s_at	AF249273	2.05	8	1438089_a_at	BE853331	3.675	17
**BCLAF1**		1373868_at	BCL2-associated transcription factor 1 (predicted)	AI717547	1.8	4	201083_s_at	AA740754	1.79	12	1438089_a_at	BE853331	3.675	17
**BCLAF1**		1373868_at	BCL2-associated transcription factor 1 (predicted)	AI717547	1.8	4	201084_s_at	NM_014739	1.43	12	1438089_a_at	BE853331	3.675	17
**MTHFD2**		1372808_at	Bifunctional methylenetetrahydrofolate dehydrogenase/cyclohydrolase	AW251324	2.75	4	201761_at	NM_006636	2.44	10	1419254_at	BG076333	2.997	17
**MTHFD2**		1372808_at	Bifunctional methylenetetrahydrofolate dehydrogenase/cyclohydrolase	AW251324	2.75	4	201761_at	NM_006636	2.44	10	1419253_at	BG076333	2.726	17
**MTHFD2**		1372808_at	Bifunctional methylenetetrahydrofolate dehydrogenase/cyclohydrolase	AW251324	2.75	4	201761_at	NM_006636	2.44	10	1436704_x_at	AV215673	1.748	13
**CDH1**		1386947_at	cadherin 1	NM_031334	8.43	4	201131_s_at	NM_004360	9.41	8	1448261_at	NM_009864	1.876	13
**CDH1**		1386947_at	cadherin 1	NM_031334	8.43	4	201130_s_at	L08599	5	12	1448261_at	NM_009864	1.876	13
**CPD**		1369215_a_a	carboxypeptidase D	NM_012836	1.4	4	201942_s_at	D85390	4.1	12	1418018_at	NM_007754	3.633	17
**CPD**		1369215_a_a	carboxypeptidase D	NM_012836	1.4	4	201940_at	AA897514	4.05	14	1418018_at	NM_007754	3.633	17
**CPD**		1369215_a_a	carboxypeptidase D	NM_012836	1.4	4	201941_at	BE349147	3.88	14	1418018_at	NM_007754	3.633	17
**CPD**		1369215_a_a	carboxypeptidase D	NM_012836	1.4	4	201943_s_at	NM_001304	4.61	14	1447392_s_at	BB710395	2.409	15
**CBX6**		1374703_at	Chromobox homolog 6	BI304209	1.48	0	202047_s_at	AI458128	2.33	10	1424407_s_at	BC019942	1.524	7
**CBX6**		1375307_at	chromobox homolog 6 (predicted)	BI275772	1.62	0	202048_s_at	NM_014292	1.74	10	1424407_s_at	BC019942	1.524	7
**CHGB**		1368034_at	chromogranin B	NM_012526	13.9	2	204260_at	NM_001819	31.8	14	1415885_at	NM_007694	10.9	17
**DAP**		1369941_at	death-associated protein	NM_022526	2.15	2	201095_at	NM_004394	2.85	14	1451112_s_at	BC024876	1.964	15
**DPP6**		1376183_at	Dipeptidylpeptidase 6	AI101484	3.21	0	207789_s_at	NM_001936	2.94	10	1439748_at	AV328546	5.194	17
**DPP6**		1369390_a_a	dipeptidylpeptidase 6	NM_022850	2.57	2	207789_s_at	NM_001936	2.94	10	1439748_at	AV328546	5.194	17
**DUSP8**		1372385_at	dual specificity phosphatase 8 (predicted)	AA957292	1.26	4	206374_at	NM_004420	1.42	8	1418714_at	NM_008748	2.079	13
**DYRK3**		1383649_a_a	Dual-specificity tyrosine-(Y)-phosphorylation regulated kinase 3	BI291080	1.92	2	210151_s_at	AF186773	1.42	12	1424229_at	BC006704	2.015	15
**EGR1**		1368321_at	early growth response 1	NM_012551	4.86	4	201694_s_at	NM_001964	4.72	14	1417065_at	NM_007913	4.923	17
**EGR1**		1368321_at	early growth response 1	NM_012551	4.86	4	201693_s_at	AV733950	2.23	14	1417065_at	NM_007913	4.923	17
**EPM2AIP1**		1372568_at	EPM2A (laforin) interacting protein 1 (predicted)	AI411270	1.73	4	202909_at	NM_014805	1.58	10	1434106_at	AV340515	4.85	17
**EPM2AIP1**		1372568_at	EPM2A (laforin) interacting protein 1 (predicted)	AI411270	1.73	4	202909_at	NM_014805	1.58	10	1425624_at	BC018474	4.455	15
**EPM2AIP1**		1389100_at	EPM2A (laforin) interacting protein 1 (predicted)	BF409831	2.76	4	202909_at	NM_014805	1.58	10	1454835_at	AV340515	1.908	15
**EPM2AIP1**		1372568_at	EPM2A (laforin) interacting protein 1 (predicted)	AI411270	1.73	4	202909_at	NM_014805	1.58	10	1434105_at	AV340515	1.766	13
**GCG**		1369888_at	glucagon	NM_012707	39.6	4	206422_at	NM_002054	60	14	1425952_a_at	AF276754	47.58	17
**GNAS**		1387906_a_a	GNAS complex locus	AF107845	8.34	2	217057_s_at	AF107846	26.1	14	1443007_at	AW545941	1.858	13
**GNAS**		1387906_a_a	GNAS complex locus	AF107845	8.34	2	214157_at	AA401492	14.9	14	1427789_s_at	AI561892	4.001	17

**GNAS**	1387906_a_a	GNAS complex locus	AF107845	8.34	2	211858_x_at	AF088184	2.99	14	1427789_s_at	AI561892	4.001	17
**GNAS**	1387906_a_a	GNAS complex locus	AF107845	8.34	2	200981_x_at	NM_016592	2.9	14	1427789_s_at	AI561892	4.001	17
**GNAS**	1387906_a_a	GNAS complex locus	AF107845	8.34	2	212273_x_at	AI591100	2.84	14	1427789_s_at	AI561892	4.001	17
**GNAS**	1387906_a_a	GNAS complex locus	AF107845	8.34	2	200780_x_at	NM_000516	2.83	14	1427789_s_at	AI561892	4.001	17
**GNAS**	1387906_a_a	GNAS complex locus	AF107845	8.34	2	214548_x_at	AF064092	2.75	14	1443375_at	BM239335	1.976	17
**GNAS**	1369897_s_a	GNAS complex locus /// XLas protein	BI277035	1.4	2	217673_x_at	AA650558	2.29	14	1444767_at	AV253089	2.531	17
**GCH1**	1387221_at	GTP cyclohydrolase 1	NM_024356	5.85	4	204224_s_at	NM_000161	18.8	14	1429692_s_at	BB698398	9.306	17
**GCH1**	1373513_at	GTP cyclohydrolase 1	AI012419	6.23	4	204224_s_at	NM_000161	18.8	14	1420499_at	NM_008102	3.857	17
**GCH1**	1380230_at	GTP cyclohydrolase 1	AI639457	4.62	4	204224_s_at	NM_000161	18.8	14	1420499_at	NM_008102	3.857	17
**GCH1**	1368503_at	GTP cyclohydrolase 1	NM_024356	1.89	4	204224_s_at	NM_000161	18.8	14	1420499_at	NM_008102	3.857	17
**GNAI1**	1387505_at	guanine nucleotide binding protein, alpha inhibiting 1	NM_013145	1.41	0	209576_at	AL049933	3.43	12	1427510_at	U38501	2.084	17
**GNAI1**	1387505_at	guanine nucleotide binding protein, alpha inhibiting 1	NM_013145	1.41	0	209576_at	AL049933	3.43	12	1427510_at	U38501	2.084	17
**IER2**	1372389_at	immediate early response 2	BF420059	2.06	4	202081_at	NM_004907	3.84	12	1416442_at	NM_010499	3.838	15
**IGSF1**	1379252_at	Immunoglobulin superfamily, member 4A	AW522833	1.42	-2	209030_s_at	NM_014333	4.28	14	1440412_at	BB312687	1.857	15
**IGSF1**	1379252_at	Immunoglobulin superfamily, member 4A	AW522833	1.42	-2	209031_at	AL519710	3.59	12	1440412_at	BB312687	1.857	15
**IGSF1**	1379252_at	Immunoglobulin superfamily, member 4A	AW522833	1.42	-2	209032_s_at	AF132811	2.4	14	1440412_at	BB312687	1.857	15
**IRS2**	1371091_at	insulin receptor substrate 2	not on RG230A array			209184_s_at	BF700086	2.44	14	1443969_at	BE199054	10.08	17
**IRS2**	1397999_at	Insulin receptor substrate 2	not on RG230A array			209185_s_at	AF073310	1.51	12	1443969_at	BE199054	10.08	17
**ISL1**	1369681_at	ISL1 transcription factor, LIM/homeodomain 1	NM_017339	1.5	2	206104_at	NM_002202	26.9	14	1450723_at	BQ176915	20.03	17
**ISL1**	1369681_at	ISL1 transcription factor, LIM/homeodomain 1	NM_017339	1.5	2	206104_at	NM_002202	26.9	14	1422720_at	BQ176915	8.868	17
**JUNB**	1387788_at	Jun-B oncogene	NM_021836	2	4	201473_at	NM_002229	3.05	12	1415899_at	NM_008416	2.173	15
**NDN**	1371947_at	necdin (predicted)	BG671865	1.7	0	209550_at	U35139	1.82	12	1437853_x_at	BB074430	1.908	13
**NEO1**	1390468_at	Neogenin	BF397709	1.42	2	204321_at	NM_002499	1.59	12	1447693_s_at	BB350308	2.818	17
**NFIC**	1371176_at	nuclear factor I/C	AB012233	1.35	4	213298_at	X12492	1.05	10	1450661_x_at	NM_008688	2.342	17
**NR4A2**	1387410_at	nuclear receptor subfamily 4, group A, member 2	U72345	1.5	4	216248_s_at	S77154	3.27	10	1455034_at	BB703394	2.905	15
**NR4A2**	1387410_at	nuclear receptor subfamily 4, group A, member 2	U72345	1.5	4	204622_x_at	NM_006186	2.93	10	1450749_a_at	NM_013613	2.527	15
**NR4A2**	1387410_at	nuclear receptor subfamily 4, group A, member 2	U72345	1.5	4	204621_s_at	AI935096	2.17	12	1447863_s_at	BB322941	1.991	17
**NOL5A**	1368173_at	nucleolar protein 5	NM_021754	1.78	2	200874_s_at	BE796327	1.57	14	1450986_at	BB729616	2.348	15
**PPY**	1369196_at	pancreatic polypeptide	NM_012626	4.39	4	210670_at	M15788	42.4	14	1420440_at	NM_008918	78.13	17
**PGBD5**	1389597_at	piggyBac transposable element derived 5 (predicted)	BI293445	1.35	0	219225_at	NM_024554	1.96	8	1460570_at	AV151597	1.918	15
**PCSK1N**	1367835_at	proprotein convertase subtilisin/kexin type 1 inhibitor	NM_019279	9.6	2	218952_at	NM_013271	10.8	14	1416965_at	AF181560	5.882	15
**RYBP**	1372674_at	RING1 and YY1 binding protein (predicted)	BE107155	1.42	2	201845_s_at	AB029551	2.02	8	1428219_at	AK010548	2.064	15
**RYBP**	1372674_at	RING1 and YY1 binding protein (predicted)	BE107155	1.42	2	201846_s_at	NM_012234	1.46	8	1428219_at	AK010548	2.064	15
**RYBP**	1372674_at	RING1 and YY1 binding protein (predicted)	BE107155	1.42	2	201844_s_at	W84482	1.29	10	1428219_at	AK010548	2.064	15
**SCN3B**	1370850_at	sodium channel, voltage-gated, type III, beta	AA685184	1.75	0	204722_at	AW007335	1.28	10	1426328_a_at	AY049036	1.972	15
**SLC7A1**	1368391_at	solute carrier family 7 (cationic amino acid transporter, y+ system), me	AB066224	2.29	4	206566_at	NM_003045	1.69	14	1454992_at	BB264620	1.915	15
**SLC7A1**	1368392_at	solute carrier family 7 (cationic amino acid transporter, y+ system), me	AB066224	1.42	4	212295_s_at	AA148507	1.78	12	1454992_at	BB264620	1.915	15
**TIPARP**	1374446_at	TCDD-inducible poly(ADP-ribose) polymerase (predicted)	AI179464	1.49	4	212665_at	AL556438	2.52	12	1452160_at	BB707122	8.334	17
**TIPARP**	1374446_at	TCDD-inducible poly(ADP-ribose) polymerase (predicted)	AI179464	1.49	4	212665_at	AL556438	2.52	12	1452161_at	BB707122	2.728	13
**TIPARP**	1374446_at	TCDD-inducible poly(ADP-ribose) polymerase (predicted)	AI179464	1.49	4	212665_at	AL556438	2.52	12	1426721_s_at	BB707122	2.648	17
**TRIB3**	1386321_s_a	tribbles homolog 3 (Drosophila)	H31287	3.09	4	218145_at	NM_021158	1.62	12	1456225_x_at	BB508622	2.256	15
**TRIB3**	1370695_s_a	tribbles homolog 3 (Drosophila)	AB020967	2.41	4	218145_at	NM_021158	1.62	12	1456225_x_at	BB508622	2.256	15
**TRIB3**	1370694_at	tribbles homolog 3 (Drosophila)	AB020967	1.57	4	218145_at	NM_021158	1.62	12	1456225_x_at	BB508622	2.256	15
**TNFRSF12A**	1371785_at	tumor necrosis factor receptor superfamily, member 12a	BI303379	1.91	2	218368_s_at	NM_016639	1.51	12	1418572_x_at	NM_013749	4.485	17
**TACSTD1**	1388199_at	tumor-associated calcium signal transducer 1	BG376410	10.2	2	201839_s_at	NM_002354	10.7	12	1447899_x_at	AV099587	3.826	17
**WBP11**	1372575_at	WW domain binding protein 11 (predicted)	AA892364	1.67	4	217822_at	NM_016312	1.22	10	1447401_at	AW909375	1.908	15
**ZBTB10**	1376125_at	Zinc finger and BTB domain containing 10	BI289112	1.8	4	219312_s_at	NM_023929	2.16	10	1436067_at	BG068032	2.246	17
**ZBTB10**	1387453_at	zinc finger and BTB domain containing 10	NM_024489	1.88	4	219312_s_at	NM_023929	2.16	10	1436068_at	BG068032	1.62	17
**ZKSCAN1**	1373106_at	Zinc finger protein 36, C3H type-like 2	AI178069	1.47	2	214900_at	AC004522	1.18	12	1452519_a_at	X14678	1.701	15
**ZKSCAN1**	1389851_at	zinc finger protein 36, C3H type-like 2 (predicted)	AA943730	1.37	6	214670_at	AA653300	1.81	12	1452519_a_at	X14678	1.701	15

**ZDHHC14**	1372989_at	Zinc finger, DHHC domain containing 14	BI296586	1.39	2	219247_s_at	NM_024630	1.24	12	1438151_x_at	BB544336	11.38	17
**ZDHHC14**	1372989_at	Zinc finger, DHHC domain containing 14	BI296586	1.39	2	219247_s_at	NM_024630	1.24	12	1437614_x_at	AV223474	1.705	15
**SST**	1367762_at	somatostatin	NM_012659	17.1	4	213921_at	NM_001048	57.4	14	1417954_at	NM_009215	22.47	17

Islet-abundant and , in human tissue data set, pancreatic duct cell-enriched as compared to beta cell fraction

symbol	probe set	gene (Rat)	Accession	av FC	eta sco	probe set	Accession	av FC	eta sco	probe set	Accession	av FC	eta scor
**BACE2**	1377390_at	beta-site APP-cleaving enzyme 2	BG381587	1.74	4	217867_x_at	NM_012105	1.74	8	1435581_at	BE947462	7.756	17
**BACE2**	1377390_at	beta-site APP-cleaving enzyme 2	BG381587	1.74	4	217867_x_at	NM_012105	1.74	8	1437846_x_at	BB348062	7.746	17
**BACE2**	1377390_at	beta-site APP-cleaving enzyme 2	BG381587	1.74	4	217867_x_at	NM_012105	1.74	8	1416673_at	NM_019517	5.473	17
**BACE2**	1377390_at	beta-site APP-cleaving enzyme 2	BG381587	1.74	4	217867_x_at	NM_012105	1.74	8	1438645_x_at	BB558905	3.612	15
**CXCL1**	1387316_at	chemokine (C-X-C motif) ligand 1	NM_030845	6.38	6	204470_at	NM_001511	2.7	8	1457644_s_at	BB554288	14.79	17
**CXCL1**	1387316_at	chemokine (C-X-C motif) ligand 1	NM_030845	6.38	6	204470_at	NM_001511	2.7	8	1441855_x_at	BB554288	11.86	17
**CXCL1**	1387316_at	chemokine (C-X-C motif) ligand 1	NM_030845	6.38	6	204470_at	NM_001511	2.7	8	1419209_at	NM_008176	5.93	17
**DUSP1**	1368147_at	dual specificity phosphatase 1	BE110108	1.84	4	201044_x_at	AA530892	3.09	10	1448830_at	NM_013642	3.849	17
**DUSP1**	1368146_at	dual specificity phosphatase 1	BE110108	1.77	4	201041_s_at	NM_004417	2.16	10	1448830_at	NM_013642	3.849	17
**KLF6**	1387060_at	Kruppel-like factor 6 (core promoter element binding protein(	NM_031642	5.62	6	208961_s_at	AB017493	3.47	10	1418280_at	NM_011803	1.794	15
**KLF6**	1388986_at	Kruppel-like factor 6 (Core promoter element binding protein)	AI598339	3.76	4	208961_s_at	AB017493	3.47	10	1433508_at	AV025472	1.903	13
**RIPK4**	1389648_at	Receptor-interacting serine-threonine kinase 4 (predicted)	AI170382	3.17	4	221215_s_at	NM_020639	1.8	10	1418488_s_at	AF302127	3.269	17
**RIPK4**	1389648_at	Receptor-interacting serine-threonine kinase 4 (predicted)	AI170382	3.17	4	221215_s_at	NM_020639	1.8	10	1418487_at	AF302127	2.111	11
