# Supplementary material for: Clusters of Conserved Beta Cell Marker Genes for Assessment of Beta Cell Phenotype
Source: PLoS One. 2011 Sep 2;6(9):e24134. doi: 10.1371/journal.pone.0024134 (PMC3166300; doi:10.1371/journal.pone.0024134)
Supplement: Table S2 — Statistically overrepresented functional gene ontologies in cultured versus laser capture microdissected human beta cells. 2736 probe sets were ≥1.5 fold (LCB) up-regulated (p<0.05, n = 3) in cultured FACS-enriched versus laser capture microdissected (LCM) human beta cells. Table S2 shows the functional gene ontologies that are statistically (p<0.001) overrepresented in the cultured (blue) versus LCM (green) beta cells. For comparison corresponding mRNA expression signals in freshly isolated islets (red) and statistics versus LCM beta cells are also shown. Cultured FACS-enriched beta cells show a concerted up-regulation of the transcriptional-protein synthetic pathway, starting from mRNA splicing and processing, translational initiation and ribosomal RNA to protein folding in endoplasmic reticulum and subsequent processing through Golgi apparatus to the secretory vesicle. (PDF) [file pone.0024134.s006.pdf]

probe set	gene	Accession	beta	se	LCM	se	LCB	*P value*		LCM	SE	islets	se	LCB	*P value*	
**37 Gene Ontology 'actin cytoskeleton' genes in 2122 annotated genes (genome-wide: 110/11929, p-value: 0.000264) ******																
38964_r_at	WAS: Wiskott-Aldrich syndrome (eczema-thrombocytopenia)	U12707	3093	99	1083	46	**-2.6**	*0.001*	*	1083	46	609	116	**-1.3**	*0.041*	
200634_at	PFN1: profilin 1	NM_005022	1268	59	51	23	**-14.2**	*0.001*	*	51	23	36	10	**-0.3**	*0.601*	
200645_at	GABARAP: GABA(A) receptor-associated protein	NM_007278	3623	166	1304	258	**-2.1**	*0.003*	*	1304	258	1115	157	**-0.8**	*0.572*	
200696_s_at	GSN: gelsolin (amyloidosis, Finnish type)	NM_000177	2252	145	464	90	**-3.6**	*0.001*	*	464	90	599	227	**0.5**	*0.624*	
200727_s_at	ACTR2: ARP2 actin-related protein 2 homolog (yeast)	AA699583	196	16	88	17	**-1.6**	*0.010*	*	88	17	179	40	**1.2**	*0.140*	
200748_s_at	FTH1: ferritin, heavy polypeptide 1	NM_002032	4871	148	129	64	**-20.7**	*0.000*	*	129	64	902	297	**2.6**	*0.115*	
200764_s_at	CTNNA1: catenin (cadherin-associated protein), alpha 1, 102kDa	AI826881	655	48	40	17	**-9.5**	*0.003*	*	40	17	129	34	**1.5**	*0.105*	
200801_x_at	ACTB: actin, beta	NM_001101	7426	199	1165	353	**-4.2**	*0.000*	*	1165	353	1044	289	**-0.5**	*0.804*	
200950_at	ARPC1A: actin related protein 2/3 complex, subunit 1A, 41kDa	NM_006409	648	41	41	10	**-11.0**	*0.003*	*	41	10	63	10	**1.0**	*0.197*	
201021_s_at	DSTN: destrin (actin depolymerizing factor)	BF697964	2290	65	150	69	**-8.7**	*0.000*	*	150	69	298	83	**0.9**	*0.243*	
201237_at	CAPZA2: capping protein (actin filament) muscle Z-line, alpha 2	AV685920	456	25	43	12	**-7.1**	*0.001*	*	43	12	277	121	**1.7**	*0.192*	
201349_at	SLC9A3R1: solute carrier family 9 (sodium/hydrogen exchanger), member 3 regulator 1	NM_004252	286	41	58	17	**-3.1**	*0.019*	*	58	17	83	15	**0.8**	*0.332*	
201668_x_at	MARCKS: myristoylated alanine-rich protein kinase C substrate	AW163148	192	23	46	8	**-3.0**	*0.016*	*	46	8	44	9	**-0.7**	*0.878*	
201745_at	TWF1: twinfilin, actin-binding protein, homolog 1 (Drosophila)	NM_002822	324	52	80	28	**-2.3**	*0.024*	*	80	28	463	23	**3.7**	*0.001*	*
201949_x_at	CAPZB: capping protein (actin filament) muscle Z-line, beta	AL572341	303	38	79	28	**-2.3**	*0.012*	*	79	28	56	19	**-0.6**	*0.536*	
201954_at	ARPC1B /// LOC653888: actin related protein 2/3 complex, subunit 1B, 41kDa /// similar to A	NM_005720	326	48	51	15	**-3.9**	*0.022*	*	51	15	38	16	**-0.6**	*0.573*	
203139_at	DAPK1: death-associated protein kinase 1	NM_004938	350	22	82	26	**-2.8**	*0.002*	*	82	26	310	75	**2.0**	*0.082*	
203242_s_at	PDLIM5: PDZ and LIM domain 5	BG054550	143	15	37	7	**-2.8**	*0.009*	*	37	7	34	4	**-0.7**	*0.774*	
204288_s_at	SORBS2: sorbin and SH3 domain containing 2	NM_021069	331	44	63	19	**-3.3**	*0.014*	*	63	19	48	9	**-0.6**	*0.538*	
205190_at	PLS1: plastin 1 (I isoform)	NM_002670	426	37	66	11	**-4.9**	*0.007*	*	66	11	440	138	**3.1**	*0.113*	
207279_s_at	NEBL: nebulette	NM_016365	143	13	37	10	**-2.6**	*0.004*	*	37	10	88	19	**1.4**	*0.097*	
208374_s_at	CAPZA1: capping protein (actin filament) muscle Z-line, alpha 1	NM_006135	222	12	60	10	**-2.8**	*0.001*	*	60	10	166	29	**1.8**	*0.056*	
208622_s_at	VIL2: villin 2 (ezrin)	AA670344	387	66	78	23	**-3.0**	*0.032*	*	78	23	89	11	**0.7**	*0.692*	
208633_s_at	MACF1: microtubule-actin crosslinking factor 1	W61052	346	21	26	7	**-9.0**	*0.002*	*	26	7	20	5	**-0.6**	*0.564*	
208679_s_at	ARPC2: actin related protein 2/3 complex, subunit 2, 34kDa	AF279893	1173	118	25	13	**-24.6**	*0.010*	*	25	13	100	30	**1.6**	*0.114*	
208736_at	ARPC3: actin related protein 2/3 complex, subunit 3, 21kDa	AF004561	399	21	18	6	**-13.7**	*0.002*	*	18	6	41	8	**1.3**	*0.083*	
211926_s_at	MYH9: myosin, heavy chain 9, non-muscle	AI827941	291	24	79	19	**-2.5**	*0.003*	*	79	19	67	13	**-0.7**	*0.633*	
211963_s_at	ARPC5: actin related protein 2/3 complex, subunit 5, 16kDa	AL516350	1455	150	85	25	**-11.1**	*0.010*	*	85	25	783	71	**6.0**	*0.005*	*
211995_x_at	ACTG1: actin, gamma 1	AL567820	6835	283	448	181	**-9.1**	*0.000*	*	448	181	2086	434	**2.4**	*0.048*	*
212325_at	DKFZP686A01247: hypothetical protein	AK027231	226	28	22	12	**-5.3**	*0.010*	*	22	12	44	18	**0.6**	*0.370*	
212398_at	RDX: radixin	AI057093	179	7	41	7	**-3.4**	*0.000*	*	41	7	28	5	**-1.0**	*0.192*	
213101_s_at	ACTR3: ARP3 actin-related protein 3 homolog (yeast)	Z78330	1949	108	657	129	**-2.2**	*0.002*	*	657	129	2315	374	**2.3**	*0.036*	*
214726_x_at	ADD1: adducin 1 (alpha)	AL556041	311	11	124	45	**-1.6**	*0.045*	*	124	45	88	20	**-0.6**	*0.517*	
219509_at	MYOZ1: myozenin 1	NM_021245	142	10	51	16	**-1.8**	*0.014*	*	51	16	61	13	**0.7**	*0.660*	
219866_at	CLIC5: chloride intracellular channel 5	NM_016929	196	32	68	22	**-1.7**	*0.036*	*	68	22	86	19	**0.7**	*0.579*	
221676_s_at	CORO1C: coronin, actin binding protein, 1C	BC002342	208	19	93	22	**-1.6**	*0.017*	*	93	22	735	266	**3.1**	*0.136*	
221881_s_at	CLIC4: chloride intracellular channel 4	AI638420	354	38	45	22	**-4.2**	*0.005*	*	45	22	43	19	**-0.2**	*0.948*	
**17 Gene Ontology 'ATP synthesis coupled proton transport' genes in 2122 annotated genes (genome-wide: 37/11929, p-value: 0.000482) ******																
200078_s_at	ATP6V0B: ATPase, H+ transporting, lysosomal 21kDa, V0 subunit b	BC005876	1246	101	469	159	**-1.7**	*0.021*	*	469	159	257	84	**-0.7**	*0.325*	
200096_s_at	ATP6V0E1: ATPase, H+ transporting, lysosomal 9kDa, V0 subunit e1	AI862255	2025	84	121	26	**-12.2**	*0.001*	*	121	26	873	93	**5.0**	*0.010*	*
200818_at	ATP5O: ATP synthase, H+ transporting, mitochondrial F1 complex, O subunit (oligomycin se	NM_001697	675	32	38	12	**-11.7**	*0.001*	*	38	12	124	55	**0.8**	*0.255*	
201089_at	ATP6V1B2: ATPase, H+ transporting, lysosomal 56/58kDa, V1 subunit B2	NM_001693	1510	84	46	25	**-17.2**	*0.002*	*	46	25	221	88	**1.4**	*0.177*	
201322_at	ATP5B: ATP synthase, H+ transporting, mitochondrial F1 complex, beta polypeptide	NM_001686	2580	134	101	38	**-15.6**	*0.002*	*	101	38	411	69	**2.3**	*0.027*	*
201527_at	ATP6V1F: ATPase, H+ transporting, lysosomal 14kDa, V1 subunit F	NM_004231	779	36	310	59	**-1.9**	*0.005*	*	310	59	290	35	**-0.7**	*0.788*	
201971_s_at	ATP6V1A: ATPase, H+ transporting, lysosomal 70kDa, V1 subunit A	NM_001690	171	6	16	8	**-6.1**	*0.000*	*	16	8	16	4	**0.5**	*0.936*	
202325_s_at	ATP5J: ATP synthase, H+ transporting, mitochondrial F0 complex, subunit F6	NM_001685	899	126	261	125	**-1.8**	*0.023*	*	261	125	748	262	**1.0**	*0.197*	
202874_s_at	ATP6V1C1: ATPase, H+ transporting, lysosomal 42kDa, V1 subunit C1	NM_001695	406	7	18	4	**-16.5**	*0.000*	*	18	4	72	14	**2.4**	*0.053*	
205711_x_at	ATP5C1: ATP synthase, H+ transporting, mitochondrial F1 complex, gamma polypeptide 1	NM_005174	1921	73	93	23	**-14.6**	*0.001*	*	93	23	734	117	**5.0**	*0.028*	*
207809_s_at	ATP6AP1: ATPase, H+ transporting, lysosomal accessory protein 1	NM_001183	1734	110	454	108	**-2.7**	*0.001*	*	454	108	211	39	**-1.2**	*0.142*	
208899_x_at	ATP6V1D: ATPase, H+ transporting, lysosomal 34kDa, V1 subunit D	AF100741	725	53	20	10	**-19.9**	*0.004*	*	20	10	60	24	**0.9**	*0.231*	
208909_at	UQCRFS1: ubiquinol-cytochrome c reductase, Rieske iron-sulfur polypeptide 1	BC000649	1197	84	226	54	**-3.7**	*0.001*	*	226	54	843	166	**2.2**	*0.054*	
212041_at	ATP6V0D1: ATPase, H+ transporting, lysosomal 38kDa, V0 subunit d1	AL566172	500	38	79	32	**-3.7**	*0.001*	*	79	32	124	32	**0.7**	*0.381*	
213041_s_at	ATP5D: ATP synthase, H+ transporting, mitochondrial F1 complex, delta subunit	BE798517	139	8	60	14	**-1.7**	*0.013*	*	60	14	37	9	**-0.9**	*0.238*	
213738_s_at	ATP5A1: ATP synthase, H+ transporting, mitochondrial F1 complex, alpha subunit 1, cardia	AI587323	2704	211	895	240	**-2.0**	*0.005*	*	895	240	3390	287	**2.6**	*0.003*	*
219173_at	MYO15B: myosin XVB pseudogene	NM_024957	146	12	67	13	**-1.6**	*0.011*	*	67	13	54	9	**-0.8**	*0.478*	
**45 Gene Ontology 'cell motility' genes in 2122 annotated genes (genome-wide: 130/11929, p-value: 0.000032) *******																
200600_at	MSN: moesin	NM_002444	362	62	70	16	**-3.3**	*0.036*	*	70	16	200	67	**1.2**	*0.187*	
200727_s_at	ACTR2: ARP2 actin-related protein 2 homolog (yeast)	AA699583	196	16	88	17	**-1.6**	*0.010*	*	88	17	179	40	**1.2**	*0.140*	
200748_s_at	FTH1: ferritin, heavy polypeptide 1	NM_002032	4871	148	129	64	**-20.7**	*0.000*	*	129	64	902	297	**2.6**	*0.115*	
200801_x_at	ACTB: actin, beta	NM_001101	7426	199	1165	353	**-4.2**	*0.000*	*	1165	353	1044	289	**-0.5**	*0.804*	
200813_s_at	PAFAH1B1: platelet-activating factor acetylhydrolase, isoform Ib, alpha subunit 45kDa	BE256969	199	13	90	16	**-1.7**	*0.007*	*	90	16	104	10	**0.9**	*0.500*	
200973_s_at	TSPAN3: tetraspanin 3	NM_005724	1432	58	55	26	**-14.6**	*0.000*	*	55	26	172	27	**1.6**	*0.035*	*
201168_x_at	ARHGDIA /// LOC728908: Rho GDP dissociation inhibitor (GDI) alpha /// similar to Rho GDI	NM_004309	1128	58	135	36	**-5.7**	*0.000*	*	135	36	49	16	**-1.4**	*0.127*	
201237_at	CAPZA2: capping protein (actin filament) muscle Z-line, alpha 2	AV685920	456	25	43	12	**-7.1**	*0.001*	*	43	12	277	121	**1.7**	*0.192*	
201426_s_at	VIM: vimentin	AI922599	2142	307	62	16	**-22.2**	*0.021*	*	62	16	142	49	**0.9**	*0.240*	
201616_s_at	CALD1: caldesmon 1	AL577531	168	11	52	10	**-2.4**	*0.002*	*	52	10	38	8	**-0.8**	*0.370*	
201668_x_at	MARCKS: myristoylated alanine-rich protein kinase C substrate	AW163148	192	23	46	8	**-3.0**	*0.016*	*	46	8	44	9	**-0.7**	*0.878*	
201841_s_at	HSPB1: heat shock 27kDa protein 1	NM_001540	1481	85	35	18	**-22.3**	*0.002*	*	35	18	46	12	**0.6**	*0.674*	
201949_x_at	CAPZB: capping protein (actin filament) muscle Z-line, beta	AL572341	303	38	79	28	**-2.3**	*0.012*	*	79	28	56	19	**-0.6**	*0.536*	
201954_at	ARPC1B /// LOC653888: actin related protein 2/3 complex, subunit 1B, 41kDa /// similar to A	NM_005720	326	48	51	15	**-3.9**	*0.022*	*	51	15	38	16	**-0.6**	*0.573*	
202109_at	ARFIP2: ADP-ribosylation factor interacting protein 2 (arfaptin 2)	NM_012402	350	6	58	14	**-4.3**	*0.000*	*	58	14	48	8	**-0.7**	*0.539*	
202203_s_at	AMFR: autocrine motility factor receptor	NM_001144	131	8	33	7	**-3.0**	*0.001*	*	33	7	32	5	**-0.7**	*0.922*	
202224_at	CRK: v-crk sarcoma virus CT10 oncogene homolog (avian)	BF304695	119	5	35	7	**-2.5**	*0.001*	*	35	7	59	9	**1.1**	*0.113*	
202859_x_at	IL8: interleukin 8	NM_000584	2144	156	44	12	**-32.8**	*0.005*	*	44	12	2923	856	**31.2**	*0.078*	
202914_s_at	ARHGEF11: Rho guanine nucleotide exchange factor (GEF) 11	NM_014784	90	5	26	7	**-2.4**	*0.003*	*	26	7	29	6	**0.7**	*0.732*	
203311_s_at	ARF6: ADP-ribosylation factor 6	M57763	179	4	71	10	**-2.0**	*0.003*	*	71	10	45	7	**-1.1**	*0.096*	
203476_at	TPBG: trophoblast glycoprotein	NM_006670	164	29	39	11	**-2.5**	*0.038*	*	39	11	101	28	**1.3**	*0.147*	
204035_at	SCG2: secretogranin II (chromogranin C)	NM_003469	3353	229	877	181	**-2.8**	*0.001*	*	877	181	1053	287	**0.6**	*0.635*	
206116_s_at	TPM1: tropomyosin 1 (alpha)	NM_000366	139	20	29	14	**-2.5**	*0.014*	*	29	14	52	14	**0.8**	*0.306*	
208372_s_at	LIMK1: LIM domain kinase 1	AF134379	129	11	49	9	**-1.9**	*0.005*	*	49	9	42	10	**-0.7**	*0.627*	
208374_s_at	CAPZA1: capping protein (actin filament) muscle Z-line, alpha 1	NM_006135	222	12	60	10	**-2.8**	*0.001*	*	60	10	166	29	**1.8**	*0.056*	
208633_s_at	MACF1: microtubule-actin crosslinking factor 1	W61052	346	21	26	7	**-9.0**	*0.002*	*	26	7	20	5	**-0.6**	*0.564*	
208640_at	RAC1: ras-related C3 botulinum toxin substrate 1 (rho family, small GTP binding protein Ra	BG292367	2768	223	1405	156	**-1.6**	*0.010*	*	1405	156	3680	348	**2.1**	*0.012*	*
208679_s_at	ARPC2: actin related protein 2/3 complex, subunit 2, 34kDa	AF279893	1173	118	25	13	**-24.6**	*0.010*	*	25	13	100	30	**1.6**	*0.114*	
208736_at	ARPC3: actin related protein 2/3 complex, subunit 3, 21kDa	AF004561	399	21	18	6	**-13.7**	*0.002*	*	18	6	41	8	**1.3**	*0.083*	
208864_s_at	TXN: thioredoxin	AF313911	1028	87	83	39	**-6.8**	*0.003*	*	83	39	344	89	**1.9**	*0.081*	
208991_at	STAT3: signal transducer and activator of transcription 3 (acute-phase response factor)	AA634272	1624	178	175	72	**-5.3**	*0.007*	*	175	72	267	59	**0.8**	*0.380*	
209026_x_at	TUBB: tubulin, beta	AF141349	1735	135	43	23	**-20.9**	*0.005*	*	43	23	79	23	**0.7**	*0.336*	
209109_s_at	TSPAN6: tetraspanin 6	U84895	93	17	19	6	**-2.9**	*0.038*	*	19	6	54	9	**1.7**	*0.035*	
209114_at	TSPAN1: tetraspanin 1	AF133425	910	67	31	25	**-12.4**	*0.002*	*	31	25	36	22	**0.0**	*0.888*	
210839_s_at	ENPP2: ectonucleotide pyrophosphatase/phosphodiesterase 2 (autotaxin)	D45421	1543	154	49	25	**-16.5**	*0.009*	*	49	25	521	127	**4.8**	*0.060*	
210845_s_at	PLAUR: plasminogen activator, urokinase receptor	U08839	220	30	39	11	**-3.5**	*0.016*	*	39	11	522	203	**4.5**	*0.140*	
211793_s_at	ABI2: abl interactor 2	AF260261	334	6	116	17	**-2.3**	*0.003*	*	116	17	76	16	**-1.0**	*0.161*	
211963_s_at	ARPC5: actin related protein 2/3 complex, subunit 5, 16kDa	AL516350	1455	150	85	25	**-11.1**	*0.010*	*	85	25	783	71	**6.0**	*0.005*	*
211995_x_at	ACTG1: actin, gamma 1	AL567820	6835	283	448	181	**-9.1**	*0.000*	*	448	181	2086	434	**2.4**	*0.048*	*
212180_at	CRKL: v-crk sarcoma virus CT10 oncogene homolog (avian)-like	AK000311	214	20	59	16	**-2.5**	*0.004*	*	59	16	322	58	**3.3**	*0.038*	*
212481_s_at	TPM4: tropomyosin 4	AI214061	173	27	39	13	**-2.6**	*0.021*	*	39	13	614	299	**3.0**	*0.194*	
213101_s_at	ACTR3: ARP3 actin-related protein 3 homolog (yeast)	Z78330	1949	108	657	129	**-2.2**	*0.002*	*	657	129	2315	374	**2.3**	*0.036*	*
214560_at	FPRL2: formyl peptide receptor-like 2	NM_002030	99	11	46	7	**-1.6**	*0.019*	*	46	7	46	7	**0.7**	*0.975*	
218368_s_at	TNFRSF12A: tumor necrosis factor receptor superfamily, member 12A	NM_016639	809	64	103	36	**-4.9**	*0.002*	*	103	36	76	20	**-0.5**	*0.556*	
221664_s_at	F11R: F11 receptor	AF154005	290	21	126	33	**-1.6**	*0.020*	*	126	33	110	16	**-0.6**	*0.706*	
**20 Gene Ontology 'coated pit' genes in 2122 annotated genes (genome-wide: 45/11929, p-value: 0.000247) ******																
38340_at	HIP1R /// LOC728014: huntingtin interacting protein 1 related /// similar to huntingtin interact	AB014555	851	18	248	30	**-2.9**	*0.000*	*	248	30	154	23	**-1.2**	*0.070*	
200613_at	AP2M1: adaptor-related protein complex 2, mu 1 subunit	NM_004068	1136	67	147	61	**-4.6**	*0.000*	*	147	61	74	14	**-0.6**	*0.351*	
200615_s_at	AP2B1: adaptor-related protein complex 2, beta 1 subunit	AL567295	140	11	29	9	**-3.1**	*0.001*	*	29	9	60	12	**1.2**	*0.108*	
201279_s_at	DAB2: disabled homolog 2, mitogen-responsive phosphoprotein (Drosophila)	BC003064	97	13	38	12	**-1.6**	*0.030*	*	38	12	54	9	**0.8**	*0.350*	

201412_at	LRP10: low density lipoprotein receptor-related protein 10	NM_014045	1932	154	371	54	**-4.0**	*0.005*	*	371	54	316	54	**-0.8**	*0.506*	
201613_s_at	AP1G2: adaptor-related protein complex 1, gamma 2 subunit	BC000519	135	15	55	10	**-1.8**	*0.014*	*	55	10	29	4	**-1.3**	*0.109*	
202067_s_at	LDLR: low density lipoprotein receptor (familial hypercholesterolemia)	AI861942	98	12	32	6	**-2.1**	*0.016*	*	32	6	37	4	**0.8**	*0.555*	
202120_x_at	AP2S1: adaptor-related protein complex 2, sigma 1 subunit	NM_004069	377	46	26	12	**-7.8**	*0.012*	*	26	12	42	10	**0.7**	*0.390*	
203142_s_at	AP3B1: adaptor-related protein complex 3, beta 1 subunit	NM_003664	488	33	83	22	**-4.0**	*0.001*	*	83	22	140	21	**1.1**	*0.130*	
203216_s_at	MYO6: myosin VI	NM_004999	96	13	29	11	**-1.9**	*0.018*	*	29	11	252	70	**4.0**	*0.082*	
203300_x_at	AP1S2: adaptor-related protein complex 1, sigma 2 subunit	NM_003916	143	10	46	10	**-2.3**	*0.002*	*	46	10	60	11	**0.8**	*0.403*	
205423_at	AP1B1: adaptor-related protein complex 1, beta 1 subunit	NM_001127	449	23	186	36	**-1.8**	*0.006*	*	186	36	97	24	**-1.2**	*0.120*	
205710_at	LRP2: low density lipoprotein-related protein 2	NM_004525	114	6	56	7	**-1.7**	*0.003*	*	56	7	40	7	**-1.0**	*0.144*	
209559_at	HIP1R: huntingtin interacting protein 1 related	AB013384	155	10	74	12	**-1.6**	*0.007*	*	74	12	62	12	**-0.8**	*0.521*	
209822_s_at	VLDLR: very low density lipoprotein receptor	L22431	510	36	41	13	**-8.0**	*0.003*	*	41	13	299	116	**2.5**	*0.155*	
214953_s_at	APP: amyloid beta (A4) precursor protein (peptidase nexin-II, Alzheimer disease)	X06989	2296	93	521	239	**-2.5**	*0.010*	*	521	239	727	142	**0.7**	*0.508*	
215236_s_at	PICALM: phosphatidylinositol binding clathrin assembly protein	AV721177	107	3	12	3	**-6.2**	*0.000*	*	12	3	14	4	**0.6**	*0.626*	
216295_s_at	CLTA: clathrin, light chain (Lca)	X81636	1271	71	85	26	**-9.9**	*0.001*	*	85	26	224	48	**1.5**	*0.080*	
217749_at	COPG: coatomer protein complex, subunit gamma	NM_016128	319	30	131	26	**-1.8**	*0.009*	*	131	26	53	11	**-1.5**	*0.082*	
218261_at	AP1M2: adaptor-related protein complex 1, mu 2 subunit	NM_005498	127	10	55	12	**-1.7**	*0.011*	*	55	12	46	11	**-0.7**	*0.585*	

27 Gene Ontology 'cytosolic large ribosomal subunit (sensu Eukaryota)' genes in 2122 annotated genes (genome-wide: 60/11929, p-value: 0.000019) ***

200002_at	RPL35: ribosomal protein L35	NM_007209	3469	109	460	89	**-5.7**	*0.000*	*	460	89	515	62	**0.8**	*0.643*	
200003_s_at	RPL28: ribosomal protein L28	NM_000991	3659	88	99	35	**-23.3**	*0.000*	*	99	35	69	16	**-0.6**	*0.502*	
200010_at	RPL11: ribosomal protein L11	NM_000975	3282	146	61	25	**-31.7**	*0.002*	*	61	25	51	22	**-0.4**	*0.770*	
200012_x_at	RPL21: ribosomal protein L21	NM_000982	5354	280	1553	424	**-2.4**	*0.003*	*	1553	424	2019	471	**0.7**	*0.503*	
200013_at	RPL24: ribosomal protein L24	NM_000986	3712	267	867	262	**-2.8**	*0.002*	*	867	262	2270	445	**1.5**	*0.067*	
200022_at	RPL18: ribosomal protein L18	NM_000979	2927	54	168	77	**-9.9**	*0.000*	*	168	77	212	53	**0.6**	*0.665*	
200029_at	RPL19: ribosomal protein L19	NM_000981	4667	146	653	173	**-5.0**	*0.000*	*	653	173	953	347	**0.6**	*0.496*	
200031_s_at	RPS11: ribosomal protein S11	NM_001015	5575	229	1749	405	**-2.3**	*0.003*	*	1749	405	2766	422	**1.0**	*0.157*	
200034_s_at	RPL6: ribosomal protein L6	NM_000970	3165	202	1216	396	**-1.7**	*0.022*	*	1216	396	1979	354	**0.9**	*0.225*	
200062_s_at	RPL30: ribosomal protein L30	L05095	4687	190	2485	328	**-1.5**	*0.008*	*	2485	328	4096	368	**1.3**	*0.031*	
200074_s_at	RPL14: ribosomal protein L14	U16738	1560	137	76	20	**-13.8**	*0.007*	*	76	20	1082	214	**8.4**	*0.041*	*
200763_s_at	RPLP1: ribosomal protein, large, P1	NM_001003	5073	147	1302	233	**-3.0**	*0.000*	*	1302	233	1001	221	**-0.8**	*0.400*	
200823_x_at	RPL29: ribosomal protein L29	NM_000992	1786	348	56	14	**-19.0**	*0.038*	*	56	14	37	5	**-0.9**	*0.309*	
200909_s_at	RPLP2: ribosomal protein, large, P2	NM_001004	2704	91	260	58	**-7.6**	*0.000*	*	260	58	544	136	**1.1**	*0.161*	
200936_at	RPL8: ribosomal protein L8	NM_000973	4011	97	332	134	**-7.2**	*0.000*	*	332	134	282	78	**-0.4**	*0.766*	
200937_s_at	RPL5: ribosomal protein L5	NM_000969	3254	135	199	63	**-10.6**	*0.000*	*	199	63	1339	157	**4.2**	*0.010*	*
201033_x_at	RPLP0: ribosomal protein, large, P0	NM_001002	7178	289	1932	260	**-3.0**	*0.000*	*	1932	260	5637	289	**2.3**	*0.001*	*
201154_x_at	RPL4: ribosomal protein L4	NM_000968	6054	129	441	117	**-9.5**	*0.000*	*	441	117	909	124	**1.3**	*0.052*	
201217_x_at	RPL3: ribosomal protein L3	NM_000967	8268	325	2522	508	**-2.4**	*0.001*	*	2522	508	3549	514	**1.0**	*0.228*	
201596_x_at	KRT18: keratin 18	NM_000224	1896	274	122	30	**-10.0**	*0.022*	*	122	30	232	80	**0.8**	*0.305*	
202029_x_at	RPL38: ribosomal protein L38	NM_000999	4001	148	1136	170	**-2.8**	*0.000*	*	1136	170	2169	356	**1.3**	*0.083*	
203034_s_at	hCG_21078 /// RPL27A: ribosomal protein L27a /// hCG21078	NM_000990	5330	132	315	90	**-11.5**	*0.000*	*	315	90	501	146	**0.8**	*0.352*	
212042_x_at	RPL7: ribosomal protein L7	BG389744	4078	336	1500	362	**-1.9**	*0.007*	*	1500	362	3570	255	**1.7**	*0.012*	*
213687_s_at	RPL35A: ribosomal protein L35a	BE968801	2041	108	377	87	**-3.9**	*0.000*	*	377	87	1172	327	**1.6**	*0.128*	
214167_s_at	RPLP0 /// RPLP0-like: ribosomal protein, large, P0 /// similar to ribosomal protein P0	AA555113	2764	142	75	17	**-26.5**	*0.002*	*	75	17	1042	116	**9.5**	*0.013*	*
216383_at	hCG_2040224 /// LOC285053 /// LOC390354 /// LOC650986 /// LOC729955 /// RPL18A: rib	U52111	90	9	9	4	**-5.8**	*0.004*	*	9	4	11	3	**0.5**	*0.660*	
222050_at	FXR2: Fragile X mental retardation, autosomal homolog 2	AI703341	227	27	71	15	**-2.2**	*0.014*	*	71	15	60	8	**-0.7**	*0.571*	

28 Gene Ontology 'cytosolic small ribosomal subunit (sensu Eukaryota)' genes in 2122 annotated genes (genome-wide: 47/11929, p-value: 0.000000) ******

200024_at	RPS5: ribosomal protein S5	NM_001009	2772	420	46	12	**-37.9**	*0.023*	*	46	12	161	11	**2.4**	*0.002*	*
200031_s_at	RPS11: ribosomal protein S11	NM_001015	5575	229	1749	405	**-2.3**	*0.003*	*	1749	405	2766	422	**1.0**	*0.157*	
200081_s_at	RPS6: Ribosomal protein S6	BE741754	3798	232	582	179	**-4.3**	*0.001*	*	582	179	1924	364	**1.9**	*0.047*	*
200082_s_at	RPS7: Ribosomal protein S7	AI805587	2926	234	529	139	**-3.8**	*0.002*	*	529	139	2519	237	**3.2**	*0.004*	*
200652_at	SSR2: signal sequence receptor, beta (translocon-associated protein beta)	NM_003145	671	65	87	12	**-5.9**	*0.010*	*	87	12	172	44	**1.1**	*0.186*	
200741_s_at	RPS27: ribosomal protein S27 (metallopanstimulin 1)	NM_001030	4524	107	534	189	**-5.3**	*0.000*	*	534	189	586	201	**0.4**	*0.858*	
200819_s_at	RPS15: ribosomal protein S15	NM_001018	4831	121	668	195	**-4.9**	*0.000*	*	668	195	504	134	**-0.6**	*0.529*	
200858_s_at	RPS8: ribosomal protein S8	NM_001012	2256	91	72	28	**-18.9**	*0.001*	*	72	28	179	32	**1.3**	*0.068*	
200949_x_at	RPS20: ribosomal protein S20	NM_001023	4177	227	1632	231	**-2.0**	*0.001*	*	1632	231	2632	311	**1.2**	*0.067*	
201049_s_at	RPS18: ribosomal protein S18	NM_022551	5603	334	1170	288	**-3.4**	*0.001*	*	1170	288	3039	810	**1.3**	*0.136*	
201094_at	RPS29: ribosomal protein S29	NM_001032	4113	136	1770	316	**-1.8**	*0.009*	*	1770	316	2669	117	**1.2**	*0.091*	
201258_at	RPS16: ribosomal protein S16	NM_001020	1755	346	44	10	**-24.2**	*0.038*	*	44	10	128	35	**1.5**	*0.127*	
201665_x_at	RPS17: ribosomal protein S17	NM_001021	4317	442	1890	291	**-1.7**	*0.014*	*	1890	291	2637	386	**1.0**	*0.203*	
202649_x_at	RPS19: ribosomal protein S19	NM_001022	3782	118	202	131	**-9.0**	*0.000*	*	202	131	397	120	**0.7**	*0.334*	
203107_x_at	RPS2: ribosomal protein S2	NM_002952	6590	177	1649	346	**-3.0**	*0.001*	*	1649	346	2526	444	**1.0**	*0.199*	
205321_at	EIF2S3: eukaryotic translation initiation factor 2, subunit 3 gamma, 52kDa	NM_001415	128	19	40	8	**-2.2**	*0.029*	*	40	8	42	4	**0.8**	*0.841*	
208645_s_at	RPS14: ribosomal protein S14	AF116710	5114	89	258	100	**-12.1**	*0.000*	*	258	100	511	179	**0.7**	*0.302*	
208692_at	RPS3: ribosomal protein S3	U14990	4894	243	1240	185	**-3.1**	*0.000*	*	1240	185	2356	429	**1.3**	*0.106*	
212391_x_at	RPS3A: ribosomal protein S3A	AI925635	7339	267	612	410	**-5.7**	*0.000*	*	612	410	4505	400	**3.4**	*0.002*	*
212652_s_at	SNX4: sorting nexin 4	AA524345	153	18	67	16	**-1.5**	*0.025*	*	67	16	300	57	**2.7**	*0.045*	*
212955_s_at	POLR2I /// RPSA: ribosomal protein SA /// polymerase (RNA) II (DNA directed) polypeptide	AL037557	268	11	78	17	**-2.5**	*0.001*	*	78	17	73	9	**-0.7**	*0.796*	
216348_at	LOC402057 /// RPS17: ribosomal protein S17 /// similar to 40S ribosomal protein S17	AL049693	237	19	72	22	**-2.1**	*0.005*	*	72	22	58	15	**-0.6**	*0.625*	
216505_x_at	LOC133569 /// LOC649303 /// LOC654029 /// LOC728791 /// LOC730965 /// LOC732348 ///	AL118502	2234	47	67	14	**-24.7**	*0.000*	*	67	14	204	56	**1.6**	*0.129*	
217466_x_at	LOC400963 /// LOC440589 /// LOC441013 /// LOC645173 /// LOC646294 /// LOC650055 ///	L48784	2109	51	37	6	**-44.9**	*0.001*	*	37	6	34	7	**-0.7**	*0.754*	
217747_s_at	RPS9: ribosomal protein S9	NM_001013	2568	219	62	15	**-28.6**	*0.007*	*	62	15	110	18	**1.1**	*0.112*	
217753_s_at	LOC644166 /// LOC644191 /// LOC728937 /// RPS26: ribosomal protein S26 /// similar to 40	NM_001029	1937	206	47	27	**-20.3**	*0.011*	*	47	27	212	68	**1.6**	*0.124*	
217882_at	TMEM111: transmembrane protein 111	NM_018447	425	31	66	21	**-4.2**	*0.001*	*	66	21	96	13	**0.9**	*0.300*	
222240_s_at	ISYNA1: myo-inositol 1-phosphate synthase A1	AL137749	115	7	32	15	**-2.0**	*0.019*	*	32	15	21	3	**-0.4**	*0.520*	

32 Gene Ontology 'ER to Golgi vesicle-mediated transport' genes in 2122 annotated genes (genome-wide: 73/11929, p-value: 0.000006) ****

200698_at	KDELR2: KDEL (Lys-Asp-Glu-Leu) endoplasmic reticulum protein retention receptor 2	AL542253	668	77	90	42	**-4.0**	*0.007*	*	90	42	449	56	**2.7**	*0.008*	*
200922_at	KDELR1: KDEL (Lys-Asp-Glu-Leu) endoplasmic reticulum protein retention receptor 1	NM_006801	519	34	25	8	**-13.1**	*0.003*	*	25	8	32	6	**0.7**	*0.534*	
201047_x_at	RAB6A: RAB6A, member RAS oncogene family	BC003617	511	20	259	31	**-1.6**	*0.004*	*	259	31	302	75	**0.7**	*0.635*	
201096_s_at	ARF4: ADP-ribosylation factor 4	AL537042	1067	40	107	68	**-4.8**	*0.001*	*	107	68	557	129	**2.2**	*0.053*	
201264_at	COPE: coatomer protein complex, subunit epsilon	NM_007263	301	22	27	8	**-7.2**	*0.003*	*	27	8	17	4	**-0.8**	*0.333*	
201359_at	COPB1: coatomer protein complex, subunit beta 1	NM_016451	766	66	232	63	**-2.2**	*0.004*	*	232	63	675	99	**1.8**	*0.026*	*
201526_at	ARF5: ADP-ribosylation factor 5	NM_001662	799	63	241	101	**-1.9**	*0.014*	*	241	101	103	29	**-0.7**	*0.302*	
201583_s_at	SEC23B: Sec23 homolog B (S. cerevisiae)	NM_006363	327	19	48	8	**-5.3**	*0.002*	*	48	8	181	25	**2.7**	*0.025*	*
201831_s_at	VDP: vesicle docking protein p115	BE875592	159	18	53	11	**-2.1**	*0.012*	*	53	11	45	5	**-0.7**	*0.560*	
202211_at	ARFGAP3: ADP-ribosylation factor GTPase activating protein 3	BC005122	163	24	61	17	**-1.7**	*0.029*	*	61	17	476	27	**5.3**	*0.000*	*
202361_at	SEC24C: SEC24 related gene family, member C (S. cerevisiae)	NM_004922	165	16	36	11	**-2.9**	*0.004*	*	36	11	57	7	**1.0**	*0.200*	
202418_at	YIF1A: Yip1 interacting factor homolog A (S. cerevisiae)	NM_020470	300	29	71	27	**-2.5**	*0.005*	*	71	27	30	8	**-0.8**	*0.272*	
203293_s_at	LMAN1: lectin, mannose-binding, 1	NM_005570	141	12	28	8	**-3.4**	*0.002*	*	28	8	28	9	**0.4**	*0.952*	
203311_s_at	ARF6: ADP-ribosylation factor 6	M57763	179	4	71	10	**-2.0**	*0.003*	*	71	10	45	7	**-1.1**	*0.096*	
204426_at	TMED2: transmembrane emp24 domain trafficking protein 2	NM_006815	655	102	28	10	**-13.2**	*0.025*	*	28	10	22	3	**-0.5**	*0.627*	
204630_s_at	GOSR1: golgi SNAP receptor complex member 1	NM_004871	241	38	60	20	**-2.4**	*0.024*	*	60	20	60	11	**-0.4**	*0.996*	
206491_s_at	NAPA: N-ethylmaleimide-sensitive factor attachment protein, alpha	NM_003827	477	27	229	48	**-1.5**	*0.019*	*	229	48	90	14	**-1.6**	*0.092*	
207265_s_at	KDELR3: KDEL (Lys-Asp-Glu-Leu) endoplasmic reticulum protein retention receptor 3	NM_016657	416	36	116	25	**-2.6**	*0.004*	*	116	25	133	18	**0.8**	*0.602*	
207305_s_at	KIAA1012: KIAA1012	NM_014939	179	22	64	21	**-1.7**	*0.020*	*	64	21	180	31	**1.6**	*0.045*	*
207791_s_at	RAB1A: RAB1A, member RAS oncogene family	NM_004161	340	54	10	4	**-19.3**	*0.025*	*	10	4	36	7	**2.0**	*0.047*	
208097_s_at	TXNDC1: thioredoxin domain containing 1	NM_030755	207	15	37	11	**-3.7**	*0.001*	*	37	11	60	10	**1.0**	*0.187*	
208684_at	COPA: coatomer protein complex, subunit alpha	U24105	651	22	263	40	**-2.0**	*0.003*	*	263	40	127	9	**-1.5**	*0.072*	
208734_x_at	RAB2A: RAB2A, member RAS oncogene family	M28213	547	25	97	45	**-3.2**	*0.003*	*	97	45	172	53	**0.7**	*0.343*	
208750_s_at	ARF1: ADP-ribosylation factor 1	AA580004	1646	28	21	7	**-48.8**	*0.000*	*	21	7	21	6	**-0.4**	*0.949*	
210616_s_at	SEC31A: SEC31 homolog A (S. cerevisiae)	AB020712	523	23	123	38	**-2.8**	*0.002*	*	123	38	190	32	**0.9**	*0.249*	
211622_s_at	ARF3: ADP-ribosylation factor 3	M33384	235	21	66	17	**-2.4**	*0.004*	*	66	17	58	11	**-0.6**	*0.692*	
214257_s_at	SEC22B: SEC22 vesicle trafficking protein homolog B (S. cerevisiae)	AA890010	525	34	106	30	**-3.3**	*0.001*	*	106	30	270	123	**0.6**	*0.311*	
216032_s_at	ERGIC3: ERGIC and golgi 3	AF091085	297	25	87	18	**-2.4**	*0.003*	*	87	18	59	12	**-0.9**	*0.282*	
217726_at	COPZ1: coatomer protein complex, subunit zeta 1	NM_016057	306	21	106	27	**-2.0**	*0.005*	*	106	27	218	17	**1.4**	*0.033*	
217749_at	COPG: coatomer protein complex, subunit gamma	NM_016128	319	30	131	26	**-1.8**	*0.009*	*	131	26	53	11	**-1.5**	*0.082*	
217959_s_at	TRAPPC4: trafficking protein particle complex 4	NM_016146	247	30	26	7	**-6.2**	*0.015*	*	26	7	155	11	**4.1**	*0.002*	*
221423_s_at	YIPF5: Yip1 domain family, member 5	NM_030799	182	7	18	10	**-5.2**	*0.000*	*	18	10	36	5	**1.0**	*0.221*	

62 Gene Ontology 'GTPase activity' genes in 2122 annotated genes (genome-wide: 171/11929, p-value: 0.000000) *****

200059_s_at	RHOA: ras homolog gene family, member A	BC001360	2529	62	286	106	-5.5	0.000	*	286	106	637	93	1.3	0.069	
31846_at	RHOD: ras homolog gene family, member D	AW003733	1173	45	512	60	-1.9	0.001	*	512	60	268	65	-1.3	0.051	
564_at	GNA11: guanine nucleotide binding protein (G protein), alpha 11 (Gq class)	M69013	446	8	58	17	-5.2	0.000	*	58	17	43	9	-0.7	0.479	
200744_s_at	GNB1: guanine nucleotide binding protein (G protein), beta polypeptide 1	AI741124	441	90	34	12	-7.0	0.044	*	34	12	49	16	0.6	0.497	
200750_s_at	RAN: RAN, member RAS oncogene family	AF054183	397	29	43	8	-6.8	0.004	*	43	8	283	40	4.5	0.024	*
200778_s_at	SEPT2: septin 2	AI191427	700	50	119	40	-3.7	0.001	*	119	40	602	108	2.9	0.034	*
200780_x_at	GNAS: GNAS complex locus	NM_000516	11337	255	1392	445	-5.3	0.000	*	1392	445	2486	727	0.8	0.282	
200823_x_at	RPL29: ribosomal protein L29	NM_000992	1786	348	56	14	-19.0	0.038	*	56	14	37	5	-0.9	0.309	
200852_x_at	GNB2: guanine nucleotide binding protein (G protein), beta polypeptide 2	NM_005273	985	9	69	60	-5.9	0.004	*	69	60	104	48	0.3	0.670	
200863_s_at	RAB11A: RAB11A, member RAS oncogene family	AI215102	2322	183	447	173	-3.1	0.002	*	447	173	1088	232	1.3	0.097	
200885_at	RHOC: ras homolog gene family, member C	NM_005167	235	35	82	19	-1.9	0.030	*	82	19	75	12	-0.7	0.768	
200927_s_at	RAB14: RAB14, member RAS oncogene family	AA919115	550	32	44	6	-9.9	0.003	*	44	6	233	42	3.5	0.043	*
201047_x_at	RAB6A: RAB6A, member RAS oncogene family	BC003617	511	20	259	31	-1.6	0.004	*	259	31	302	75	0.7	0.635	
201096_s_at	ARF4: ADP-ribosylation factor 4	AL537042	1067	40	107	68	-4.8	0.001	*	107	68	557	129	2.2	0.053	
201156_s_at	RAB5C: RAB5C, member RAS oncogene family	AF141304	396	67	89	22	-2.8	0.034	*	89	22	53	10	-0.9	0.239	
201526_at	ARF5: ADP-ribosylation factor 5	NM_001662	799	63	241	101	-1.9	0.014	*	241	101	103	29	-0.7	0.302	
201659_s_at	ARL1: ADP-ribosylation factor-like 1	NM_001177	247	27	81	15	-2.2	0.012	*	81	15	378	57	3.2	0.029	*
201912_s_at	GSPT1: G1 to S phase transition 1	NM_002094	167	17	32	9	-3.4	0.006	*	32	9	119	27	2.1	0.069	
202154_x_at	TUBB3: tubulin, beta 3	NM_006086	657	61	63	18	-6.9	0.007	*	63	18	139	30	1.2	0.110	
202615_at	GNAQ: Guanine nucleotide binding protein (G protein), q polypeptide	BF222895	329	17	116	22	-2.1	0.002	*	116	22	200	42	1.1	0.174	
202976_s_at	RHOBTB3: Rho-related BTB domain containing 3	NM_014899	287	61	36	11	-4.4	0.050	*	36	11	73	17	1.1	0.143	
203175_at	RHOG: ras homolog gene family, member G (rho G)	NM_001665	435	54	190	35	-1.6	0.025	*	190	35	91	26	-1.2	0.089	
203311_s_at	ARF6: ADP-ribosylation factor 6	M57763	179	4	71	10	-2.0	0.003	*	71	10	45	7	-1.1	0.096	
203911_at	RAP1GAP: RAP1 GTPase activating protein	NM_002885	503	26	213	23	-2.0	0.001	*	213	23	93	16	-1.7	0.016	*
204102_s_at	EEF2: eukaryotic translation elongation factor 2	NM_001961	4347	131	212	71	-13.2	0.000	*	212	71	196	79	-0.4	0.888	
204540_at	EEF1A2: eukaryotic translation elongation factor 1 alpha 2	NM_001958	1557	147	273	93	-3.5	0.003	*	273	93	25	7	-4.5	0.116	
204892_x_at	EEF1A1: eukaryotic translation elongation factor 1 alpha 1	NM_001402	10917	553	2311	725	-3.1	0.001	*	2311	725	8404	###	2.2	0.019	*
205321_at	EIF2S3: eukaryotic translation initiation factor 2, subunit 3 gamma, 52kDa	NM_001415	128	19	40	8	-2.2	0.029	*	40	8	42	4	0.8	0.841	
205924_at	RAB3B: RAB3B, member RAS oncogene family	BC005035	227	23	41	10	-3.7	0.006	*	41	10	31	9	-0.7	0.521	
206113_s_at	RAB5A: RAB5A, member RAS oncogene family	NM_004162	113	11	27	6	-2.9	0.006	*	27	6	23	4	-0.7	0.599	
206152_at	CENTG1: centaurin, gamma 1	NM_014770	217	17	108	15	-1.6	0.009	*	108	15	85	24	-0.8	0.474	
207124_s_at	GNB5: guanine nucleotide binding protein (G protein), beta 5	NM_006578	114	7	26	6	-3.1	0.001	*	26	6	34	5	0.9	0.350	
207791_s_at	RAB1A: RAB1A, member RAS oncogene family	NM_004161	340	54	10	4	-19.3	0.025	*	10	4	36	7	2.0	0.047	
208112_x_at	EHD1: EH-domain containing 1	NM_006795	446	33	158	36	-2.0	0.004	*	158	36	86	21	-1.0	0.175	
208290_s_at	EIF5: eukaryotic translation initiation factor 5	NM_001969	103	6	23	4	-3.4	0.001	*	23	4	71	13	2.0	0.054	
208640_at	RAC1: ras-related C3 botulinum toxin substrate 1 (rho family, small GTP binding protein Ra	BG292367	2768	223	1405	156	-1.6	0.010	*	1405	156	3680	348	2.1	0.012	*
208727_s_at	CDC42: cell division cycle 42 (GTP binding protein, 25kDa)	BC002711	791	160	144	54	-2.9	0.045	*	144	54	208	48	0.7	0.425	
208734_x_at	RAB2A: RAB2A, member RAS oncogene family	M28213	547	25	97	45	-3.2	0.003	*	97	45	172	53	0.7	0.343	
208750_s_at	ARF1: ADP-ribosylation factor 1	AA580004	1646	28	21	7	-48.8	0.000	*	21	7	21	6	-0.4	0.949	
209026_x_at	TUBB: tubulin, beta	AF141349	1735	135	43	23	-20.9	0.005	*	43	23	79	23	0.7	0.336	
209084_s_at	RAB28: RAB28, member RAS oncogene family	BE504689	70	7	10	5	-3.6	0.003	*	10	5	73	20	3.0	0.078	
209251_x_at	TUBA1C: tubulin, alpha 1c	BC004949	7838	418	420	306	-8.4	0.000	*	420	306	399	45	0.0	0.952	
209315_at	HBS1L: HBS1-like (S. cerevisiae)	AW297143	171	10	41	9	-3.0	0.001	*	41	9	56	15	0.7	0.439	
209515_s_at	RAB27A: RAB27A, member RAS oncogene family	U38654	305	36	58	28	-2.8	0.007	*	58	28	370	85	3.0	0.054	
210527_x_at	TUBA3C: tubulin, alpha 3c	L11645	1663	40	38	25	-20.8	0.000	*	38	25	70	26	0.6	0.429	
210994_x_at	TRIM23: tripartite motif-containing 23	AF230398	145	10	40	11	-2.4	0.002	*	40	11	51	9	0.8	0.494	
211573_x_at	TGM2: transglutaminase 2 (C polypeptide, protein-glutamine-gamma-glutamyltransferase)	M98478	94	10	29	10	-2.0	0.010	*	29	10	32	8	0.6	0.832	
211622_s_at	ARF3: ADP-ribosylation factor 3	M33384	235	21	66	17	-2.4	0.004	*	66	17	58	11	-0.6	0.692	
211960_s_at	RAB7A: RAB7A, member RAS oncogene family	BG261416	652	41	98	32	-4.3	0.001	*	98	32	164	47	0.8	0.316	
212099_at	RHOB: ras homolog gene family, member B	AI263909	1440	150	54	17	-16.9	0.011	*	54	17	97	42	0.5	0.418	
212117_at	RHOQ: ras homolog gene family, member Q	BF978689	463	47	82	26	-3.6	0.005	*	82	26	136	34	0.9	0.280	
212590_at	RRAS2: related RAS viral (r-ras) oncogene homolog 2	AI431643	113	22	34	14	-1.8	0.049	*	34	14	258	40	4.2	0.020	*
212639_x_at	TUBA1B: tubulin, alpha 1b	AL581768	7479	290	345	254	-9.8	0.000	*	345	254	308	63	0.0	0.900	
213404_s_at	RHEB: Ras homolog enriched in brain	BF033683	655	39	110	32	-4.0	0.000	*	110	32	1050	227	5.3	0.051	
213583_x_at	EEF1A1 /// LOC124199 /// LOC387845 /// LOC389179 /// LOC389223 /// LOC390924 /// LO(	BE964125	6558	296	3015	751	-1.5	0.029	*	3015	751	6291	777	1.4	0.039	
214352_s_at	KRAS: v-Ki-ras2 Kirsten rat sarcoma viral oncogene homolog	BF673699	226	14	57	15	-2.7	0.001	*	57	15	78	17	0.8	0.410	
214393_at	RND2: Rho family GTPase 2	AI884814	113	15	47	7	-1.7	0.033	*	47	7	33	8	-0.9	0.284	
216323_x_at	TUBA3D: tubulin, alpha 3d	K03460	1030	29	46	30	-10.7	0.000	*	46	30	78	26	0.6	0.471	
217793_at	RAB11B: RAB11B, member RAS oncogene family	AL575337	616	37	276	28	-1.9	0.002	*	276	28	175	35	-1.1	0.093	
218360_at	RAB22A: RAB22A, member RAS oncogene family	NM_020673	125	12	31	8	-2.8	0.005	*	31	8	209	25	4.5	0.013	*
218700_s_at	RAB7L1: RAB7, member RAS oncogene family-like 1	BC002585	182	25	21	6	-5.4	0.018	*	21	6	13	5	-0.7	0.414	
222005_s_at	GNG3: guanine nucleotide binding protein (G protein), gamma 3	AL538966	262	35	75	21	-2.2	0.016	*	75	21	162	37	1.2	0.128	

11 Gene Ontology 'heterogeneous nuclear ribonucleoprotein complex' genes in 2122 annotated genes (genome-wide: 18/11929, p-value: 0.000493) ****

200016_x_at	HNRPA1: heterogeneous nuclear ribonucleoprotein A1	NM_002136	4871	249	1840	267	-2.1	0.001	*	1840	267	4608	384	1.9	0.006	*
200097_s_at	HNRPK: heterogeneous nuclear ribonucleoprotein K	AI701949	628	35	31	15	-11.3	0.001	*	31	15	108	39	1.2	0.177	
200593_s_at	HNRPU: heterogeneous nuclear ribonucleoprotein U (scaffold attachment factor A)	BC003621	722	116	226	124	-1.6	0.044	*	226	124	997	88	2.3	0.009	*
201132_at	HNRPH2: heterogeneous nuclear ribonucleoprotein H2 (H')	NM_019597	185	13	16	23	-3.4	0.007	*	16	23	98	35	1.4	0.129	
205292_s_at	HNRPA2B1: heterogeneous nuclear ribonucleoprotein A2/B1	NM_002137	1154	88	79	23	-9.7	0.004	*	79	23	604	63	5.0	0.008	*
208765_s_at	HNRPR: heterogeneous nuclear ribonucleoprotein R	NM_005826	361	34	46	19	-4.5	0.003	*	46	19	53	18	0.4	0.811	
212016_s_at	PTBP1: polypyrimidine tract binding protein 1	AA679988	141	12	42	14	-2.1	0.006	*	42	14	33	11	-0.5	0.640	
213356_x_at	hCG_2023776 /// HNRPA1 /// HNRPA1L-2 /// HNRPA1P4 /// HNRPA1P5 /// LOC391670 /// I	AL568186	5170	202	2055	238	-2.1	0.001	*	2055	238	5050	395	2.0	0.006	*
213619_at	HNRPH1: Heterogeneous nuclear ribonucleoprotein H1 (H)	AV753392	632	64	79	27	-5.0	0.006	*	79	27	180	42	1.2	0.122	
213762_x_at	RBMX: RNA binding motif protein, X-linked	AI452524	363	18	97	26	-2.6	0.002	*	97	26	301	60	1.8	0.059	
216559_x_at	hCG_2023776 /// HNRPA1 /// HNRPA1L-2 /// HNRPA1P4 /// LOC120364 /// LOC344741 ///	AL050348	234	23	36	15	-3.7	0.003	*	36	15	231	57	3.1	0.066	

53 Gene Ontology 'intracellular protein transport' genes in 2122 annotated genes (genome-wide: 164/11929, p-value: 0.000041) *****

200063_s_at	NPM1: nucleophosmin (nucleolar phosphoprotein B23, numatrin)	BC002398	3638	242	1781	178	-1.7	0.005	*	1781	178	2655	282	1.2	0.070	
89476_r_at	NPEPL1: aminopeptidase-like 1	AA398062	856	67	309	50	-2.1	0.004	*	309	50	166	21	-1.3	0.087	
200613_at	AP2M1: adaptor-related protein complex 2, mu 1 subunit	NM_004068	1136	67	147	61	-4.6	0.000	*	147	61	74	14	-0.6	0.351	
200615_s_at	AP2B1: adaptor-related protein complex 2, beta 1 subunit	AL567295	140	11	29	9	-3.1	0.001	*	29	9	60	12	1.2	0.108	
200661_at	CTSA: cathepsin A	NM_000308	1722	128	120	41	-9.0	0.003	*	120	41	72	13	-0.7	0.367	
200662_s_at	TOMM20: translocase of outer mitochondrial membrane 20 homolog (yeast)	NM_014765	1072	159	50	16	-12.9	0.022	*	50	16	622	45	8.0	0.003	*
200698_at	KDELR2: KDEL (Lys-Asp-Glu-Leu) endoplasmic reticulum protein retention receptor 2	AL542253	668	77	90	42	-4.0	0.007	*	90	42	449	56	2.7	0.008	*
200750_s_at	RAN: RAN, member RAS oncogene family	AF054183	397	29	43	8	-6.8	0.004	*	43	8	283	40	4.5	0.024	*
200922_at	KDELR1: KDEL (Lys-Asp-Glu-Leu) endoplasmic reticulum protein retention receptor 1	NM_006801	519	34	25	8	-13.1	0.003	*	25	8	32	6	0.7	0.534	
200991_s_at	SNX17: sorting nexin 17	NM_014748	219	7	47	14	-3.1	0.001	*	47	14	46	7	-0.5	0.962	
200993_at	IPO7: importin 7	AA939270	256	26	64	11	-2.9	0.010	*	64	11	256	56	2.4	0.071	
201004_at	SSR4: signal sequence receptor, delta (translocon-associated protein delta)	NM_006280	3338	134	872	248	-2.6	0.003	*	872	248	799	142	-0.6	0.815	
201020_at	YWHAH: tyrosine 3-monooxygenase/tryptophan 5-monooxygenase activation protein, eta p	NM_003405	700	38	24	10	-17.2	0.002	*	24	10	33	9	0.6	0.541	
201112_s_at	CSE1L: CSE1 chromosome segregation 1-like (yeast)	NM_001316	256	16	59	7	-3.5	0.002	*	59	7	249	20	3.4	0.006	*
201202_at	PCNA: proliferating cell nuclear antigen	NM_002592	114	13	42	8	-1.9	0.014	*	42	8	116	20	1.8	0.047	*
201359_at	COPB1: coatomer protein complex, subunit beta 1	NM_016451	766	66	232	63	-2.2	0.004	*	232	63	675	99	1.8	0.026	*
201583_s_at	SEC23B: Sec23 homolog B (S. cerevisiae)	NM_006363	327	19	48	8	-5.3	0.002	*	48	8	181	25	2.7	0.025	*
201613_s_at	AP1G2: adaptor-related protein complex 1, gamma 2 subunit	BC000519	135	15	55	10	-1.8	0.014	*	55	10	29	4	-1.3	0.109	
201716_at	SNX1: sorting nexin 1	NM_003099	215	9	84	14	-2.0	0.003	*	84	14	93	15	0.7	0.706	
201831_s_at	VDP: vesicle docking protein p115	BE875592	159	18	53	11	-2.1	0.012	*	53	11	45	5	-0.7	0.560	
202113_s_at	SNX2: sorting nexin 2	AF043453	165	5	19	7	-5.5	0.000	*	19	7	47	8	1.4	0.055	
202120_x_at	AP2S1: adaptor-related protein complex 2, sigma 1 subunit	NM_004069	377	46	26	12	-7.8	0.012	*	26	12	42	10	0.7	0.390	
202211_at	ARFGAP3: ADP-ribosylation factor GTPase activating protein 3	BC005122	163	24	61	17	-1.7	0.029	*	61	17	476	27	5.3	0.000	*
202361_at	SEC24C: SEC24 related gene family, member C (S. cerevisiae)	NM_004922	165	16	36	11	-2.9	0.004	*	36	11	57	7	1.0	0.200	
202606_s_at	TLK1: tousled-like kinase 1	NM_012290	97	8	12	5	-4.7	0.001	*	12	5	73	11	3.3	0.020	*
203073_at	COG2: component of oligomeric golgi complex 2	NM_007357	231	24	114	17	-1.5	0.020	*	114	17	145	13	1.0	0.225	
203142_s_at	AP3B1: adaptor-related protein complex 3, beta 1 subunit	NM_003664	488	33	83	22	-4.0	0.001	*	83	22	140	21	1.1	0.130	
203216_s_at	MYO6: myosin VI	NM_004999	96	13	29	11	-1.9	0.018	*	29	11	252	70	4.0	0.082	
203300_x_at	AP1S2: adaptor-related protein complex 1, sigma 2 subunit	NM_003916	143	10	46	10	-2.3	0.002	*	46	10	60	11	0.8	0.403	
203484_at	SEC61G: Sec61 gamma subunit	NM_014302	1483	52	135	30	-8.0	0.000	*	135	30	702	77	3.6	0.010	*
203544_s_at	STAM: signal transducing adaptor molecule (SH3 domain and ITAM motif) 1	NM_003473	138	19	39	17	-1.9	0.019	*	39	17	425	70	5.7	0.026	*
204426_at	TMED2: transmembrane emp24 domain trafficking protein 2	NM_006815	655	102	28	10	-13.2	0.025	*	28	10	22	3	-0.5	0.627	
204485_s_at	TOM1L1: target of myb1 (chicken)-like 1	NM_005486	206	19	66	12	-2.3	0.006	*	66	12	299	86	2.3	0.111	
204630_s_at	GOSR1: golgi SNAP receptor complex member 1	NM_004871	241	38	60	20	-2.4	0.024	*	60	20	60	11	-0.4	0.996	

205423_at	AP1B1: adaptor-related protein complex 1, beta 1 subunit	NM_001127	449	23	186	36	**-1.8**	*0.006*	*	186	36	97	24	**-1.2**	*0.120*	
206491_s_at	NAPA: N-ethylmaleimide-sensitive factor attachment protein, alpha	NM_003827	477	27	229	48	**-1.5**	*0.019*	*	229	48	90	14	**-1.6**	*0.092*	
207657_x_at	TNPO1: transportin 1	NM_002270	224	28	94	23	**-1.6**	*0.025*	*	94	23	151	37	**0.9**	*0.268*	
207707_s_at	SEC13: SEC13 homolog (S. cerevisiae)	NM_030673	1413	62	551	55	**-2.2**	*0.001*	*	551	55	263	63	**-1.4**	*0.027*	
208684_at	COPA: coatomer protein complex, subunit alpha	U24105	651	22	263	40	**-2.0**	*0.003*	*	263	40	127	9	**-1.5**	*0.072*	
209150_s_at	TM9SF1: transmembrane 9 superfamily member 1	U94831	209	13	59	12	**-2.6**	*0.001*	*	59	12	102	19	**1.1**	*0.143*	
209176_at	SEC23IP: SEC23 interacting protein	AI332962	165	21	35	14	**-2.7**	*0.010*	*	35	14	41	15	**0.4**	*0.776*	
209452_s_at	VTI1B: vesicle transport through interaction with t-SNAREs homolog 1B (yeast)	AF035824	306	37	39	11	**-5.0**	*0.013*	*	39	11	125	20	**2.0**	*0.029*	*
210428_s_at	HGS: hepatocyte growth factor-regulated tyrosine kinase substrate	AF260566	206	12	100	18	**-1.6**	*0.012*	*	100	18	78	10	**-0.9**	*0.363*	
210658_s_at	GGA2: golgi associated, gamma adaptin ear containing, ARF binding protein 2	BC000284	127	11	57	12	**-1.6**	*0.014*	*	57	12	108	23	**1.1**	*0.145*	
211955_at	RANBP5: RAN binding protein 5	NM_002271	108	3	39	7	**-2.1**	*0.004*	*	39	7	111	26	**1.7**	*0.098*	
214734_at	EXPH5: exophilin 5	AB014524	252	26	55	12	**-3.2**	*0.008*	*	55	12	69	21	**0.6**	*0.610*	
214840_at	TOM1L2: target of myb1-like 2 (chicken)	AF038192	195	17	77	16	**-1.8**	*0.007*	*	77	16	56	8	**-0.9**	*0.324*	
216295_s_at	CLTA: clathrin, light chain (Lca)	X81636	1271	71	85	26	**-9.9**	*0.001*	*	85	26	224	48	**1.5**	*0.080*	
217726_at	COPZ1: coatomer protein complex, subunit zeta 1	NM_016057	306	21	106	27	**-2.0**	*0.005*	*	106	27	218	17	**1.4**	*0.033*	
217749_at	COPG: coatomer protein complex, subunit gamma	NM_016128	319	30	131	26	**-1.8**	*0.009*	*	131	26	53	11	**-1.5**	*0.082*	
218261_at	AP1M2: adaptor-related protein complex 1, mu 2 subunit	NM_005498	127	10	55	12	**-1.7**	*0.011*	*	55	12	46	11	**-0.7**	*0.585*	
221499_s_at	STX16: syntaxin 16	AK026970	156	26	13	5	**-6.8**	*0.028*	*	13	5	36	7	**1.5**	*0.061*	
221614_s_at	RPH3AL: rabphilin 3A-like (without C2 domains)	BC005153	274	18	55	14	**-3.5**	*0.001*	*	55	14	37	9	**-0.8**	*0.364*	

16 Gene Ontology 'mitochondrial respiratory chain complex I' genes in 2122 annotated genes (genome-wide: 34/11929, p-value: 0.000541) ****

201226_at	NDUFB8: NADH dehydrogenase (ubiquinone) 1 beta subcomplex, 8, 19kDa	NM_005004	1299	68	272	59	**-3.5**	*0.000*	*	272	59	426	67	**1.0**	*0.162*	
201304_at	NDUFA5: NADH dehydrogenase (ubiquinone) 1 alpha subcomplex, 5, 13kDa	NM_005000	815	47	68	16	**-8.4**	*0.002*	*	68	16	542	68	**5.3**	*0.016*	*
202785_at	NDUFA7: NADH dehydrogenase (ubiquinone) 1 alpha subcomplex, 7, 14.5kDa	NM_005001	156	11	71	18	**-1.5**	*0.021*	*	71	18	85	18	**0.7**	*0.615*	
202839_s_at	NDUFB7: NADH dehydrogenase (ubiquinone) 1 beta subcomplex, 7, 18kDa	NM_004146	89	4	22	11	**-2.3**	*0.015*	*	22	11	12	7	**-0.3**	*0.483*	
202941_at	NDUFV2: NADH dehydrogenase (ubiquinone) flavoprotein 2, 24kDa	NM_021074	591	32	120	46	**-3.0**	*0.002*	*	120	46	315	108	**1.0**	*0.206*	
203189_s_at	NDUFS8: NADH dehydrogenase (ubiquinone) Fe-S protein 8, 23kDa (NADH-coenzyme Q r	NM_002496	79	5	13	4	**-4.1**	*0.001*	*	13	4	10	4	**-0.6**	*0.648*	
203478_at	NDUFC1: NADH dehydrogenase (ubiquinone) 1, subcomplex unknown, 1, 6kDa	NM_002494	1165	63	188	77	**-3.7**	*0.001*	*	188	77	343	93	**0.8**	*0.268*	
203613_s_at	NDUFB6: NADH dehydrogenase (ubiquinone) 1 beta subcomplex, 6, 17kDa	NM_002493	626	25	146	50	**-2.7**	*0.004*	*	146	50	447	47	**1.9**	*0.012*	*
203621_at	NDUFB5: NADH dehydrogenase (ubiquinone) 1 beta subcomplex, 5, 16kDa	NM_002492	182	16	39	10	**-3.2**	*0.003*	*	39	10	346	28	**6.0**	*0.004*	*
208714_at	NDUFV1: NADH dehydrogenase (ubiquinone) flavoprotein 1, 51kDa	AF092131	382	12	154	46	**-1.7**	*0.031*	*	154	46	41	6	**-1.9**	*0.131*	
209224_s_at	NDUFA2: NADH dehydrogenase (ubiquinone) 1 alpha subcomplex, 2, 8kDa	BC003674	261	16	36	13	**-4.5**	*0.001*	*	36	13	76	26	**0.8**	*0.270*	
209303_at	NDUFS4: NADH dehydrogenase (ubiquinone) Fe-S protein 4, 18kDa (NADH-coenzyme Q r	BC005270	197	16	33	9	**-4.0**	*0.002*	*	33	9	109	19	**2.0**	*0.038*	*
218160_at	NDUFA8: NADH dehydrogenase (ubiquinone) 1 alpha subcomplex, 8, 19kDa	NM_014222	228	10	91	11	**-2.1**	*0.001*	*	91	11	114	40	**0.5**	*0.612*	
218226_s_at	LOC727762 /// NDUFB4: NADH dehydrogenase (ubiquinone) 1 beta subcomplex, 4, 15kDa	NM_004547	2448	130	1095	139	**-1.8**	*0.002*	*	1095	139	1129	326	**0.5**	*0.931*	
218563_at	NDUFA3: NADH dehydrogenase (ubiquinone) 1 alpha subcomplex, 3, 9kDa	NM_004542	534	22	86	36	**-3.7**	*0.001*	*	86	36	53	13	**-0.5**	*0.457*	
220864_s_at	NDUFA13: NADH dehydrogenase (ubiquinone) 1 alpha subcomplex, 13	NM_015965	487	55	56	27	**-4.7**	*0.006*	*	56	27	43	15	**-0.3**	*0.688*	

171 Gene Ontology 'mitochondrion' genes in 2122 annotated genes (genome-wide: 659/11929, p-value: 0.000001) *******

200030_s_at	SLC25A3: solute carrier family 25 (mitochondrial carrier; phosphate carrier), member 3	NM_002635	2907	209	680	140	**-3.1**	*0.002*	*	680	140	2606	205	**2.8**	*0.002*	*
200064_at	HSP90AB1: heat shock protein 90kDa alpha (cytosolic), class B member 1	AF275719	4584	178	55	24	**-48.5**	*0.001*	*	55	24	278	88	**2.1**	*0.118*	
200079_s_at	KARS: lysyl-tRNA synthetase	AF285758	706	67	247	54	**-2.0**	*0.007*	*	247	54	682	80	**1.9**	*0.015*	*
33646_g_at	GM2A: GM2 ganglioside activator	X61094	255	12	127	19	**-1.6**	*0.007*	*	127	19	128	19	**0.7**	*0.984*	
36475_at	GCAT: glycine C-acetyltransferase (2-amino-3-ketobutyrate coenzyme A ligase)	Z97630	452	27	192	23	**-1.9**	*0.002*	*	192	23	108	11	**-1.4**	*0.048*	
40850_at	FKBP8: FK506 binding protein 8, 38kDa	L37033	569	17	314	34	**-1.5**	*0.007*	*	314	34	166	18	**-1.5**	*0.029*	
200638_s_at	YWHAZ: tyrosine 3-monooxygenase/tryptophan 5-monooxygenase activation protein, zeta p	BC003623	1433	79	30	7	**-33.8**	*0.003*	*	30	7	246	143	**0.4**	*0.269*	
200658_s_at	PHB: prohibitin	AL560017	108	5	32	7	**-2.4**	*0.002*	*	32	7	38	9	**0.7**	*0.606*	
200662_s_at	TOMM20: translocase of outer mitochondrial membrane 20 homolog (yeast)	NM_014765	1072	159	50	16	**-12.9**	*0.022*	*	50	16	622	45	**8.0**	*0.003*	*
200691_s_at	HSPA9: heat shock 70kDa protein 9 (mortalin)	BC000478	588	31	182	71	**-2.0**	*0.017*	*	182	71	473	61	**1.5**	*0.037*	
200695_at	PPP2R1A: protein phosphatase 2 (formerly 2A), regulatory subunit A , alpha isoform	NM_014225	1050	24	32	23	**-15.0**	*0.000*	*	32	23	32	15	**0.2**	*0.987*	
200708_at	GOT2: glutamic-oxaloacetic transaminase 2, mitochondrial (aspartate aminotransferase 2)	NM_002080	532	36	78	43	**-3.6**	*0.001*	*	78	43	183	30	**1.1**	*0.122*	
200736_s_at	GPX1: glutathione peroxidase 1	NM_000581	987	42	32	13	**-18.6**	*0.001*	*	32	13	33	8	**0.5**	*0.949*	
200766_at	CTSD: cathepsin D	NM_001909	164	16	27	8	**-3.9**	*0.004*	*	27	8	19	5	**-0.7**	*0.462*	
200789_at	ECH1: enoyl Coenzyme A hydratase 1, peroxisomal	NM_001398	228	8	33	10	**-4.5**	*0.000*	*	33	10	59	9	**1.1**	*0.128*	
200793_s_at	ACO2: aconitase 2, mitochondrial	NM_001098	312	20	117	36	**-1.7**	*0.017*	*	117	36	103	24	**-0.5**	*0.766*	
200798_x_at	MCL1: myeloid cell leukemia sequence 1 (BCL2-related)	NM_021960	1032	50	30	8	**-23.3**	*0.002*	*	30	8	821	93	**17.7**	*0.013*	*
200800_s_at	HSPA1A /// HSPA1B: heat shock 70kDa protein 1A /// heat shock 70kDa protein 1B	NM_005345	305	52	41	14	**-4.3**	*0.030*	*	41	14	440	380	**0.0**	*0.403*	
200806_s_at	HSPD1: heat shock 60kDa protein 1 (chaperonin)	BE256479	912	97	32	15	**-15.6**	*0.011*	*	32	15	138	36	**2.0**	*0.083*	
200818_at	ATP5O: ATP synthase, H+ transporting, mitochondrial F1 complex, O subunit (oligomycin s	NM_001697	675	32	38	12	**-11.7**	*0.001*	*	38	12	124	55	**0.8**	*0.255*	
200838_at	CTSB: cathepsin B	NM_001908	1634	278	94	30	**-10.2**	*0.030*	*	94	30	42	14	**-1.0**	*0.216*	
200883_at	UQCRC2: ubiquinol-cytochrome c reductase core protein II	NM_003366	134	9	23	10	**-3.3**	*0.001*	*	23	10	125	19	**2.9**	*0.017*	*
200925_at	COX6A1: cytochrome c oxidase subunit VIa polypeptide 1	NM_004373	1890	60	109	56	**-9.4**	*0.000*	*	109	56	104	41	**-0.2**	*0.946*	
200946_x_at	GLUD1: glutamate dehydrogenase 1	AI339331	109	6	35	7	**-2.3**	*0.002*	*	35	7	80	14	**1.5**	*0.063*	
201035_s_at	HADH: hydroxyacyl-Coenzyme A dehydrogenase	BC000306	2382	164	994	152	**-1.9**	*0.003*	*	994	152	196	40	**-3.4**	*0.028*	*
201066_at	CYC1: cytochrome c-1	NM_001916	186	7	43	10	**-3.0**	*0.001*	*	43	10	60	9	**0.9**	*0.307*	
201093_x_at	SDHA: succinate dehydrogenase complex, subunit A, flavoprotein (Fp)	NM_004168	203	15	69	20	**-1.9**	*0.007*	*	69	20	86	25	**0.6**	*0.628*	
201106_at	GPX4: glutathione peroxidase 4 (phospholipid hydroperoxidase)	NM_002085	1129	65	154	44	**-4.9**	*0.000*	*	154	44	91	16	**-0.9**	*0.288*	
201119_s_at	COX8A: cytochrome c oxidase subunit 8A (ubiquitous)	NM_004074	2295	100	1146	120	**-1.7**	*0.002*	*	1146	120	850	121	**-1.0**	*0.158*	
201226_at	NDUFB8: NADH dehydrogenase (ubiquinone) 1 beta subcomplex, 8, 19kDa	NM_005004	1299	68	272	59	**-3.5**	*0.000*	*	272	59	426	67	**1.0**	*0.162*	
201251_at	PKM2: pyruvate kinase, muscle	NM_002654	2292	87	147	46	**-10.2**	*0.000*	*	147	46	56	13	**-1.2**	*0.181*	
201304_at	NDUFA5: NADH dehydrogenase (ubiquinone) 1 alpha subcomplex, 5, 13kDa	NM_005000	815	47	68	16	**-8.4**	*0.002*	*	68	16	542	68	**5.3**	*0.016*	*
201322_at	ATP5B: ATP synthase, H+ transporting, mitochondrial F1 complex, beta polypeptide	NM_001686	2580	134	101	38	**-15.6**	*0.002*	*	101	38	411	69	**2.3**	*0.027*	*
201339_s_at	SCP2: sterol carrier protein 2	NM_002979	1490	42	45	14	**-22.0**	*0.000*	*	45	14	460	67	**6.3**	*0.021*	*
201441_at	COX6B1: cytochrome c oxidase subunit Vib polypeptide 1 (ubiquitous)	NM_001863	1514	70	67	28	**-13.3**	*0.001*	*	67	28	191	67	**1.1**	*0.199*	
201599_at	OAT: ornithine aminotransferase (gyrate atrophy)	NM_000274	1461	128	52	20	**-16.6**	*0.007*	*	52	20	687	112	**7.3**	*0.027*	*
201619_at	PRDX3: peroxiredoxin 3	NM_006793	1219	64	201	84	**-3.6**	*0.001*	*	201	84	682	104	**1.9**	*0.025*	*
201754_at	COX6C: cytochrome c oxidase subunit VIc	NM_004374	2168	71	77	32	**-16.7**	*0.000*	*	77	32	484	201	**1.8**	*0.178*	
201903_at	UQCRC1: ubiquinol-cytochrome c reductase core protein I	NM_003365	1179	42	36	15	**-19.5**	*0.000*	*	36	15	54	13	**0.7**	*0.420*	
201917_s_at	SLC25A36: solute carrier family 25, member 36	AI694452	249	22	96	22	**-1.8**	*0.008*	*	96	22	163	42	**0.9**	*0.250*	
201931_at	ETFA: electron-transfer-flavoprotein, alpha polypeptide (glutaric aciduria II)	NM_000126	180	12	27	6	**-4.7**	*0.001*	*	27	6	121	23	**2.7**	*0.045*	*
201971_s_at	ATP6V1A: ATPase, H+ transporting, lysosomal 70kDa, V1 subunit A	NM_001690	171	6	16	8	**-6.1**	*0.000*	*	16	8	16	4	**0.5**	*0.936*	
202003_s_at	ACAA2: acetyl-Coenzyme A acyltransferase 2 (mitochondrial 3-oxoacyl-Coenzyme A thiolas	NM_006111	703	56	148	41	**-3.2**	*0.002*	*	148	41	403	70	**1.6**	*0.047*	*
202026_at	SDHD: succinate dehydrogenase complex, subunit D, integral membrane protein	NM_003002	146	15	18	7	**-4.9**	*0.006*	*	18	7	137	37	**3.6**	*0.080*	
202041_s_at	FIBP: fibroblast growth factor (acidic) intracellular binding protein	NM_004214	189	10	87	22	**-1.5**	*0.029*	*	87	22	66	9	**-0.7**	*0.456*	
202069_s_at	IDH3A: isocitrate dehydrogenase 3 (NAD+) alpha	AI826060	86	6	22	4	**-2.9**	*0.001*	*	22	4	134	16	**4.3**	*0.014*	*
202079_s_at	TRAK1: trafficking protein, kinesin binding 1	AI633774	113	4	53	12	**-1.6**	*0.027*	*	53	12	61	12	**0.7**	*0.645*	
202110_at	COX7B: cytochrome c oxidase subunit VIIb	NM_001866	474	23	73	27	**-4.0**	*0.000*	*	73	27	138	48	**0.7**	*0.320*	
202139_at	AKR7A2: aldo-keto reductase family 7, member A2 (aflatoxin aldehyde reductase)	NM_003689	111	6	40	12	**-1.8**	*0.012*	*	40	12	51	9	**0.8**	*0.515*	
202154_x_at	TUBB3: tubulin, beta 3	NM_006086	657	61	63	18	**-6.9**	*0.007*	*	63	18	139	30	**1.2**	*0.110*	
202325_s_at	ATP5J: ATP synthase, H+ transporting, mitochondrial F0 complex, subunit F6	NM_001685	899	126	261	125	**-1.8**	*0.023*	*	261	125	748	262	**1.0**	*0.197*	
202343_x_at	COX5B: cytochrome c oxidase subunit Vb	NM_001862	559	35	141	46	**-2.5**	*0.003*	*	141	46	104	34	**-0.6**	*0.557*	
202492_at	ATG9A: ATG9 autophagy related 9 homolog A (S. cerevisiae)	NM_024085	305	38	80	22	**-2.4**	*0.011*	*	80	22	74	14	**-0.6**	*0.834*	
202675_at	SDHB: succinate dehydrogenase complex, subunit B, iron sulfur (Ip)	NM_003000	267	7	29	6	**-6.9**	*0.000*	*	29	6	47	12	**0.9**	*0.261*	
202698_x_at	COX4I1: cytochrome c oxidase subunit IV isoform 1	NM_001861	3436	290	1744	257	**-1.5**	*0.012*	*	1744	257	3939	296	**1.8**	*0.005*	*
202783_at	NNT: nicotinamide nucleotide transhydrogenase	U40490	190	16	70	15	**-1.9**	*0.006*	*	70	15	57	11	**-0.7**	*0.540*	
202785_at	NDUFA7: NADH dehydrogenase (ubiquinone) 1 alpha subcomplex, 7, 14.5kDa	NM_005001	156	11	71	18	**-1.5**	*0.021*	*	71	18	85	18	**0.7**	*0.615*	
202839_s_at	NDUFB7: NADH dehydrogenase (ubiquinone) 1 beta subcomplex, 7, 18kDa	NM_004146	89	4	22	11	**-2.3**	*0.015*	*	22	11	12	7	**-0.3**	*0.483*	
202884_s_at	PPP2R1B: protein phosphatase 2 (formerly 2A), regulatory subunit A, beta isoform	NM_002716	129	11	46	13	**-1.9**	*0.008*	*	46	13	29	6	**-0.8**	*0.325*	
202930_s_at	SUCLA2: succinate-CoA ligase, ADP-forming, beta subunit	NM_003850	110	11	43	11	**-1.7**	*0.013*	*	43	11	263	52	**3.6**	*0.047*	*
202941_at	NDUFV2: NADH dehydrogenase (ubiquinone) flavoprotein 2, 24kDa	NM_021074	591	32	120	46	**-3.0**	*0.002*	*	120	46	315	108	**1.0**	*0.206*	
203028_s_at	CYBA: cytochrome b-245, alpha polypeptide	NM_000101	417	39	37	13	**-7.0**	*0.006*	*	37	13	33	8	**-0.5**	*0.806*	
203033_x_at	FH: fumarate hydratase	NM_000143	326	24	117	26	**-2.0**	*0.004*	*	117	26	483	93	**2.5**	*0.050*	*
203152_at	MRPL40: mitochondrial ribosomal protein L40	NM_003776	141	8	40	11	**-2.4**	*0.003*	*	40	11	64	19	**0.7**	*0.363*	
203189_s_at	NDUFS8: NADH dehydrogenase (ubiquinone) Fe-S protein 8, 23kDa (NADH-coenzyme Q r	NM_002496	79	5	13	4	**-4.1**	*0.001*	*	13	4	10	4	**-0.6**	*0.648*	
203261_at	DCTN6: dynactin 6	NM_006571	339	45	106	38	**-1.9**	*0.018*	*	106	38	375	32	**2.2**	*0.006*	*
203460_s_at	PSEN1: presenilin 1 (Alzheimer disease 3)	NM_007318	121	8	46	15	**-1.7**	*0.019*	*	46	15	99	18	**1.2**	*0.088*	
203466_at	MPV17: MpV17 mitochondrial inner membrane protein	NM_002437	229	16	71	11	**-2.5**	*0.002*	*	71	11	71	7	**0.8**	*0.995*	
203478_at	NDUFC1: NADH dehydrogenase (ubiquinone) 1, subcomplex unknown, 1, 6kDa	NM_002494	1165	63	188	77	**-3.7**	*0.001*	*	188	77	343	93	**0.8**	*0.268*	
203517_at	MTX2: metaxin 2	NM_006554	132	16	55	11	**-1.7**	*0.020*	*	55	11	182	46	**1.8**	*0.103*	
203613_s_at	NDUFB6: NADH dehydrogenase (ubiquinone) 1 beta subcomplex, 6, 17kDa	NM_002493	626	25	146	50	**-2.7**	*0.004*	*	146	50	447	47	**1.9**	*0.012*	*
203621_at	NDUFB5: NADH dehydrogenase (ubiquinone) 1 beta subcomplex, 5, 16kDa	NM_002492	182	16	39	10	**-3.2**	*0.003*	*	39	10	346	28	**6.0**	*0.004*	*

203647_s_at	FDX1: ferredoxin 1	M18003	206	19	62	14	**-2.3**	*0.005*	*	62	14	301	24	**3.4**	*0.002*	*
203658_at	SLC25A20: solute carrier family 25 (carnitine/acylcarnitine translocase), member 20	BC001689	100	3	40	6	**-2.0**	*0.002*	*	40	6	42	4	**0.8**	*0.858*	
203816_at	DGUOK: deoxyguanosine kinase	NM_001929	85	6	22	6	**-2.6**	*0.002*	*	22	6	28	4	**0.8**	*0.459*	
203946_s_at	ARG2: arginase, type II	U75667	189	27	18	7	**-6.0**	*0.020*	*	18	7	94	26	**2.4**	*0.090*	
204214_s_at	RAB32: RAB32, member RAS oncogene family	NM_006834	186	18	90	16	**-1.5**	*0.016*	*	90	16	160	38	**1.0**	*0.193*	
204285_s_at	PMAIP1: phorbol-12-myristate-13-acetate-induced protein 1	AI857639	439	73	40	12	**-6.7**	*0.029*	*	40	12	112	35	**1.3**	*0.165*	
204342_at	SLC25A24: solute carrier family 25 (mitochondrial carrier; phosphate carrier), member 24	NM_013386	196	30	67	28	**-1.6**	*0.036*	*	67	28	521	100	**4.0**	*0.037*	*
204355_at	DHX30: DEAH (Asp-Glu-Ala-His) box polypeptide 30	NM_014966	119	5	23	8	**-3.3**	*0.001*	*	23	8	20	6	**-0.5**	*0.750*	
204565_at	THEM2: thioesterase superfamily member 2	NM_018473	167	23	72	14	**-1.6**	*0.033*	*	72	14	201	50	**1.6**	*0.113*	
205052_at	AUH: AU RNA binding protein/enoyl-Coenzyme A hydratase	NM_001698	303	62	39	12	**-4.4**	*0.046*	*	39	12	376	117	**4.3**	*0.100*	
205273_s_at	PITRM1: pitrilysin metallopeptidase 1	NM_014968	537	47	248	16	**-1.8**	*0.018*	*	248	16	303	76	**0.7**	*0.546*	
205412_at	ACAT1: acetyl-Coenzyme A acetyltransferase 1 (acetoacetyl Coenzyme A thiolase)	NM_000019	1114	191	324	143	**-1.8**	*0.033*	*	324	143	1155	80	**2.0**	*0.013*	*
205711_x_at	ATP5C1: ATP synthase, H+ transporting, mitochondrial F1 complex, gamma polypeptide 1	NM_005174	1921	73	93	23	**-14.6**	*0.001*	*	93	23	734	117	**5.0**	*0.028*	*
205770_at	GSR: glutathione reductase	NM_000637	157	9	69	19	**-1.5**	*0.026*	*	69	19	85	15	**0.7**	*0.554*	
205843_x_at	CRAT: carnitine acetyltransferase	NM_000755	571	65	255	55	**-1.5**	*0.022*	*	255	55	167	20	**-1.0**	*0.249*	
207005_s_at	BCL2: B-cell CLL/lymphoma 2	NM_000657	178	17	55	25	**-1.8**	*0.020*	*	55	25	40	18	**-0.3**	*0.662*	
207275_s_at	ACSL1: acyl-CoA synthetase long-chain family member 1	NM_001995	148	10	44	7	**-2.6**	*0.001*	*	44	7	73	7	**1.3**	*0.038*	
208517_x_at	BTF3: basic transcription factor 3	NM_001207	2063	144	104	97	**-7.7**	*0.001*	*	104	97	596	186	**1.7**	*0.101*	
208629_s_at	HADHA: hydroxyacyl-Coenzyme A dehydrogenase/3-ketoacyl-Coenzyme A thiolase/enoyl-(	BG472176	376	47	58	16	**-4.2**	*0.013*	*	58	16	59	12	**0.6**	*0.943*	
208649_s_at	VCP: valosin-containing protein	AF100752	718	47	40	32	**-7.7**	*0.001*	*	40	32	33	19	**0.0**	*0.870*	
208714_at	NDUFV1: NADH dehydrogenase (ubiquinone) flavoprotein 1, 51kDa	AF092131	382	12	154	46	**-1.7**	*0.031*	*	154	46	41	6	**-1.9**	*0.131*	
208717_at	OXA1L: oxidase (cytochrome c) assembly 1-like	BC001669	298	14	113	22	**-2.0**	*0.004*	*	113	22	115	18	**0.7**	*0.953*	
208846_s_at	VDAC3: voltage-dependent anion channel 3	U90943	357	17	64	11	**-4.3**	*0.000*	*	64	11	168	30	**1.7**	*0.059*	
208905_at	CYCS: cytochrome c, somatic	BC005299	1237	58	522	85	**-1.8**	*0.004*	*	522	85	2260	210	**3.2**	*0.007*	*
208909_at	UQCRFS1: ubiquinol-cytochrome c reductase, Rieske iron-sulfur polypeptide 1	BC000649	1197	84	226	54	**-3.7**	*0.001*	*	226	54	843	166	**2.2**	*0.054*	
208910_s_at	C1QBP: complement component 1, q subcomponent binding protein	L04636	139	5	17	5	**-5.4**	*0.000*	*	17	5	45	25	**0.2**	*0.383*	
208929_x_at	RPL13: ribosomal protein L13	BC004954	4632	376	2360	354	**-1.5**	*0.012*	*	2360	354	2126	436	**-0.7**	*0.700*	
208967_s_at	AK2: adenylate kinase 2	U39945	312	23	45	13	**-4.5**	*0.002*	*	45	13	139	43	**1.4**	*0.152*	
209003_at	SLC25A11: solute carrier family 25 (mitochondrial carrier; oxoglutarate carrier), member 11	AF070548	117	4	56	11	**-1.6**	*0.021*	*	56	11	55	7	**-0.7**	*0.966*	
209009_at	ESD: esterase D/formylglutathione hydrolase	BC001169	1324	52	164	34	**-6.0**	*0.000*	*	164	34	223	29	**0.9**	*0.261*	
209018_s_at	PINK1: PTEN induced putative kinase 1	BF432478	365	19	119	30	**-2.1**	*0.004*	*	119	30	80	17	**-0.8**	*0.338*	
209075_s_at	ISCU: iron-sulfur cluster scaffold homolog (E. coli)	AY009128	868	49	198	68	**-2.8**	*0.002*	*	198	68	422	87	**1.2**	*0.116*	
209092_s_at	GLOD4: glyoxalase domain containing 4	AF061730	317	26	22	9	**-8.6**	*0.004*	*	22	9	242	101	**3.2**	*0.160*	
209095_at	DLD: dihydrolipoamide dehydrogenase	J03620	292	21	47	26	**-3.3**	*0.002*	*	47	26	413	31	**4.6**	*0.001*	*
209182_s_at	C10orf10: chromosome 10 open reading frame 10	AI302100	1052	95	139	45	**-4.8**	*0.004*	*	139	45	46	13	**-1.3**	*0.165*	
209224_s_at	NDUFA2: NADH dehydrogenase (ubiquinone) 1 alpha subcomplex, 2, 8kDa	BC003674	261	16	36	13	**-4.5**	*0.001*	*	36	13	76	26	**0.8**	*0.270*	
209228_x_at	TUSC3: tumor suppressor candidate 3	U42349	3058	85	366	95	**-5.8**	*0.000*	*	366	95	1231	249	**2.0**	*0.059*	
209303_at	NDUFS4: NADH dehydrogenase (ubiquinone) Fe-S protein 4, 18kDa (NADH-coenzyme Q r	BC005270	197	16	33	9	**-4.0**	*0.002*	*	33	9	109	19	**2.0**	*0.038*	*
209397_at	ME2: malic enzyme 2, NAD(+)-dependent, mitochondrial	BC000147	280	33	103	26	**-1.8**	*0.015*	*	103	26	342	35	**2.2**	*0.007*	*
209424_s_at	AMACR: alpha-methylacyl-CoA racemase	AI796120	144	16	57	12	**-1.8**	*0.014*	*	57	12	67	18	**0.6**	*0.674*	
209932_s_at	DUT: dUTP pyrophosphatase	U90223	608	64	129	39	**-3.0**	*0.006*	*	129	39	111	23	**-0.6**	*0.714*	
210010_s_at	SLC25A1: solute carrier family 25 (mitochondrial carrier; citrate transporter), member 1	U25147	237	27	74	11	**-2.4**	*0.015*	*	74	11	64	8	**-0.8**	*0.533*	
211662_s_at	VDAC2: voltage-dependent anion channel 2	L08666	1263	106	556	73	**-1.8**	*0.007*	*	556	73	1291	176	**1.7**	*0.038*	*
211971_s_at	LRPPRC: leucine-rich PPR-motif containing	AI653608	692	51	371	40	**-1.5**	*0.009*	*	371	40	567	24	**1.3**	*0.021*	
212038_s_at	VDAC1: voltage-dependent anion channel 1	AL515918	290	14	32	7	**-6.4**	*0.000*	*	32	7	61	32	**0.3**	*0.460*	
212085_at	SLC25A6: solute carrier family 25 (mitochondrial carrier; adenine nucleotide translocator), m	AA916851	1633	164	60	42	**-12.3**	*0.008*	*	60	42	63	28	**0.2**	*0.949*	
212088_at	PMPCA: peptidase (mitochondrial processing) alpha	BF570122	197	12	61	11	**-2.4**	*0.001*	*	61	11	52	11	**-0.7**	*0.620*	
212135_s_at	ATP2B4: ATPase, Ca++ transporting, plasma membrane 4	AW517686	705	28	176	36	**-3.0**	*0.000*	*	176	36	167	30	**-0.7**	*0.864*	
212228_s_at	COQ9: coenzyme Q9 homolog (S. cerevisiae)	AC004382	124	7	41	11	**-2.1**	*0.005*	*	41	11	63	10	**1.0**	*0.212*	
212312_at	BCL2L1: BCL2-like 1	AL117381	360	33	135	27	**-1.9**	*0.007*	*	135	27	91	14	**-0.9**	*0.251*	
212459_x_at	SUCLG2: succinate-CoA ligase, GDP-forming, beta subunit	BF593940	276	8	15	6	**-11.5**	*0.000*	*	15	6	98	11	**3.9**	*0.006*	*
212581_x_at	GAPDH: glyceraldehyde-3-phosphate dehydrogenase	BE561479	6935	945	196	45	**-23.5**	*0.019*	*	196	45	291	112	**0.5**	*0.496*	
212639_x_at	TUBA1B: tubulin, alpha 1b	AL581768	7479	290	345	254	**-9.8**	*0.000*	*	345	254	308	63	**0.0**	*0.900*	
213041_s_at	ATP5D: ATP synthase, H+ transporting, mitochondrial F1 complex, delta subunit	BE798517	139	8	60	14	**-1.7**	*0.013*	*	60	14	37	9	**-0.9**	*0.238*	
213133_s_at	GCSH /// LOC730107: glycine cleavage system protein H (aminomethyl carrier) /// similar to	AW237404	107	12	40	10	**-1.8**	*0.014*	*	40	10	185	71	**1.6**	*0.174*	
213540_at	HSD17B8: hydroxysteroid (17-beta) dehydrogenase 8	AL031228	843	57	354	68	**-1.8**	*0.006*	*	354	68	291	104	**-0.7**	*0.641*	
213738_s_at	ATP5A1: ATP synthase, H+ transporting, mitochondrial F1 complex, alpha subunit 1, cardia	AI587323	2704	211	895	240	**-2.0**	*0.005*	*	895	240	3390	287	**2.6**	*0.003*	*
213846_at	COX7C: cytochrome c oxidase subunit VIIc	AA382702	225	33	37	8	**-4.0**	*0.024*	*	37	8	84	15	**1.4**	*0.072*	
213897_s_at	MRPL23: mitochondrial ribosomal protein L23	AI832239	120	10	28	9	**-2.7**	*0.003*	*	28	9	23	4	**-0.6**	*0.642*	
214203_s_at	PRODH: proline dehydrogenase (oxidase) 1	AA074145	517	64	189	41	**-1.9**	*0.017*	*	189	41	99	10	**-1.2**	*0.152*	
214610_at	CYP11B1: cytochrome P450, family 11, subfamily B, polypeptide 1	AV702430	177	8	66	15	**-1.9**	*0.008*	*	66	15	30	9	**-1.2**	*0.130*	
216383_at	hCG_2040224 /// LOC285053 /// LOC390354 /// LOC650986 /// LOC729955 /// RPL18A: ribo	U52111	90	9	9	4	**-5.8**	*0.004*	*	9	4	11	3	**0.5**	*0.660*	
216591_s_at	hCG_1776980 /// SDHC: succinate dehydrogenase complex, subunit C, integral membrane	AF080579	127	9	35	9	**-2.5**	*0.002*	*	35	9	28	4	**-0.7**	*0.549*	
216841_s_at	SOD2: superoxide dismutase 2, mitochondrial	X15132	91	15	10	5	**-4.4**	*0.023*	*	10	5	498	238	**9.4**	*0.177*	
217791_s_at	ALDH18A1: aldehyde dehydrogenase 18 family, member A1	NM_002860	456	21	137	56	**-2.0**	*0.019*	*	137	56	95	24	**-0.5**	*0.544*	
217883_at	C2orf25: chromosome 2 open reading frame 25	NM_015702	892	93	360	62	**-1.8**	*0.012*	*	360	62	1589	218	**3.1**	*0.023*	*
217900_at	IARS2: isoleucyl-tRNA synthetase 2, mitochondrial	NM_018060	220	15	68	27	**-1.9**	*0.015*	*	68	27	164	39	**1.2**	*0.120*	
217919_s_at	MRPL42: mitochondrial ribosomal protein L42	BE782148	377	37	60	20	**-3.9**	*0.004*	*	60	20	145	22	**1.4**	*0.050*	
217933_s_at	LAP3: leucine aminopeptidase 3	NM_015907	87	4	21	7	**-2.7**	*0.003*	*	21	7	51	5	**1.6**	*0.025*	
217955_at	BCL2L13: BCL2-like 13 (apoptosis facilitator)	NM_015367	195	26	50	21	**-2.2**	*0.014*	*	50	21	426	92	**4.3**	*0.049*	*
217960_s_at	TOMM22: translocase of outer mitochondrial membrane 22 homolog (yeast)	NM_020243	129	6	49	12	**-1.9**	*0.008*	*	49	12	58	10	**0.7**	*0.601*	
218001_at	MRPS2: mitochondrial ribosomal protein S2	NM_016034	101	7	23	7	**-2.9**	*0.002*	*	23	7	22	4	**-0.5**	*0.960*	
218026_at	CCDC56: coiled-coil domain containing 56	NM_014019	1246	50	351	95	**-2.4**	*0.003*	*	351	95	194	45	**-0.9**	*0.235*	
218034_at	FIS1: fission 1 (mitochondrial outer membrane) homolog (S. cerevisiae)	NM_016068	543	33	76	16	**-5.2**	*0.001*	*	76	16	55	13	**-0.8**	*0.373*	
218046_s_at	MRPS16: mitochondrial ribosomal protein S16	NM_016065	156	11	48	10	**-2.4**	*0.002*	*	48	10	109	26	**1.3**	*0.127*	
218083_at	PTGES2: prostaglandin E synthase 2	NM_025072	615	34	115	38	**-3.4**	*0.001*	*	115	38	68	16	**-0.7**	*0.346*	
218105_s_at	MRPL4: mitochondrial ribosomal protein L4	NM_015956	279	17	138	26	**-1.5**	*0.014*	*	138	26	72	10	**-1.3**	*0.108*	
218160_at	NDUFA8: NADH dehydrogenase (ubiquinone) 1 alpha subcomplex, 8, 19kDa	NM_014222	228	10	91	11	**-2.1**	*0.001*	*	91	11	114	40	**0.5**	*0.612*	
218197_s_at	OXR1: oxidation resistance 1	NM_018002	71	7	17	5	**-2.7**	*0.004*	*	17	5	22	6	**0.6**	*0.563*	
218226_s_at	LOC727762 /// NDUFB4: NADH dehydrogenase (ubiquinone) 1 beta subcomplex, 4, 15kDa	NM_004547	2448	130	1095	139	**-1.8**	*0.002*	*	1095	139	1129	326	**0.5**	*0.931*	
218268_at	TBC1D15: TBC1 domain family, member 15	NM_022771	151	5	31	5	**-3.8**	*0.000*	*	31	5	530	222	**5.3**	*0.154*	
218270_at	MRPL24: mitochondrial ribosomal protein L24	NM_024540	290	17	128	17	**-1.8**	*0.002*	*	128	17	123	27	**-0.7**	*0.873*	
218273_s_at	PPM2C: protein phosphatase 2C, magnesium-dependent, catalytic subunit	NM_018444	136	11	59	17	**-1.5**	*0.026*	*	59	17	49	9	**-0.6**	*0.634*	
218307_at	RSAD1: radical S-adenosyl methionine domain containing 1	NM_018346	127	8	55	9	**-1.8**	*0.004*	*	55	9	52	7	**-0.7**	*0.812*	
218320_s_at	NDUFB11: NADH dehydrogenase (ubiquinone) 1 beta subcomplex, 11, 17.3kDa	NM_019056	946	44	180	64	**-3.3**	*0.001*	*	180	64	152	37	**-0.5**	*0.728*	
218398_at	MRPS30: mitochondrial ribosomal protein S30	NM_016640	106	10	39	7	**-2.0**	*0.007*	*	39	7	114	17	**2.0**	*0.033*	*
218450_at	HEBP1: heme binding protein 1	NM_015987	164	16	78	14	**-1.5**	*0.017*	*	78	14	735	79	**6.8**	*0.012*	*
218563_at	NDUFA3: NADH dehydrogenase (ubiquinone) 1 alpha subcomplex, 3, 9kDa	NM_004542	534	22	86	36	**-3.7**	*0.001*	*	86	36	53	13	**-0.5**	*0.457*	
218653_at	SLC25A15: solute carrier family 25 (mitochondrial carrier; ornithine transporter) member 15	NM_014252	137	9	63	13	**-1.6**	*0.014*	*	63	13	62	9	**-0.6**	*0.956*	
218725_at	SLC25A22: solute carrier family 25 (mitochondrial carrier: glutamate), member 22	NM_024698	147	8	66	13	**-1.7**	*0.010*	*	66	13	55	7	**-0.8**	*0.526*	
218760_at	COQ6: coenzyme Q6 homolog, monooxygenase (S. cerevisiae)	NM_015940	110	6	45	11	**-1.7**	*0.013*	*	45	11	49	7	**0.7**	*0.792*	
218773_s_at	MSRB2: methionine sulfoxide reductase B2	NM_012228	387	23	81	30	**-3.0**	*0.002*	*	81	30	129	36	**0.7**	*0.359*	
220864_s_at	NDUFA13: NADH dehydrogenase (ubiquinone) 1 alpha subcomplex, 13	NM_015965	487	55	56	27	**-4.7**	*0.006*	*	56	27	43	15	**-0.3**	*0.688*	
221142_s_at	PECR: peroxisomal trans-2-enoyl-CoA reductase	NM_018441	80	5	26	8	**-2.0**	*0.008*	*	26	8	38	8	**0.8**	*0.368*	
221478_at	BNIP3L: BCL2/adenovirus E1B 19kDa interacting protein 3-like	AL132665	341	53	100	26	**-2.2**	*0.028*	*	100	26	386	85	**2.2**	*0.067*	
221692_s_at	MRPL34: mitochondrial ribosomal protein L34	AB049652	253	19	128	21	**-1.5**	*0.012*	*	128	21	121	32	**-0.7**	*0.873*	
221693_s_at	MRPS18A: mitochondrial ribosomal protein S18A	AB049952	479	70	114	18	**-3.0**	*0.029*	*	114	18	115	22	**0.7**	*0.958*	
221881_s_at	CLIC4: chloride intracellular channel 4	AI638420	354	38	45	22	**-4.2**	*0.005*	*	45	22	43	19	**-0.2**	*0.948*	
222216_s_at	MRPL17: mitochondrial ribosomal protein L17	AK026857	446	28	158	27	**-2.2**	*0.002*	*	158	27	122	37	**-0.8**	*0.471*	

64 Gene Ontology 'mRNA processing' genes in 2122 annotated genes (genome-wide: 209/11929, p-value: 0.000034) ***

200000_s_at	PRPF8: PRP8 pre-mRNA processing factor 8 homolog (S. cerevisiae)	NM_006445	565	24	43	31	**-6.0**	*0.000*	*	43	31	36	22	**0.0**	*0.864*	
200033_at	DDX5: DEAD (Asp-Glu-Ala-Asp) box polypeptide 5	NM_004396	1853	127	764	255	**-1.5**	*0.033*	*	764	255	1432	98	**1.2**	*0.106*	
200041_s_at	BAT1: HLA-B associated transcript 1	NM_004640	390	28	27	9	**-9.1**	*0.003*	*	27	9	34	9	**0.6**	*0.620*	
200057_s_at	NONO: non-POU domain containing, octamer-binding	NM_007363	2242	174	713	168	**-2.2**	*0.003*	*	713	168	1103	183	**1.0**	*0.192*	
200060_s_at	RNPS1: RNA binding protein S1, serine-rich domain	BC001659	778	31	325	23	**-2.1**	*0.000*	*	325	23	639	83	**1.5**	*0.055*	
200097_s_at	HNRPK: heterogeneous nuclear ribonucleoprotein K	AI701949	628	35	31	15	**-11.3**	*0.001*	*	31	15	108	39	**1.2**	*0.177*	
33132_at	CPSF1: cleavage and polyadenylation specific factor 1, 160kDa	U37012	288	36	109	13	**-2.0**	*0.027*	*	109	13	55	11	**-1.4**	*0.038*	
200593_s_at	HNRPU: heterogeneous nuclear ribonucleoprotein U (scaffold attachment factor A)	BC003621	722	116	226	124	**-1.6**	*0.044*	*	226	124	997	88	**2.3**	*0.009*	*
200619_at	SF3B2: splicing factor 3b, subunit 2, 145kDa	NM_006842	244	25	106	21	**-1.6**	*0.015*	*	106	21	132	24	**0.8**	*0.465*	
200826_at	SNRPD2: small nuclear ribonucleoprotein D2 polypeptide 16.5kDa	NM_004597	1295	46	51	23	**-14.6**	*0.000*	*	51	23	55	21	**0.4**	*0.890*	

200892_s_at	SFRS10: splicing factor, arginine/serine-rich 10 (transformer 2 homolog, Drosophila)	BC000451	672	23	40	13	**-10.9**	*0.000*	*	40	13	171	32	**2.4**	*0.042*	*
201070_x_at	SF3B1: splicing factor 3b, subunit 1, 155kDa	AI739389	371	17	96	15	**-3.0**	*0.000*	*	96	15	67	11	**-1.0**	*0.199*	
201077_s_at	NHP2L1: NHP2 non-histone chromosome protein 2-like 1 (S. cerevisiae)	AF155235	1379	73	273	64	**-3.6**	*0.000*	*	273	64	496	131	**1.0**	*0.228*	
201224_s_at	SRRM1: serine/arginine repetitive matrix 1	AU147713	1278	75	506	113	**-1.8**	*0.007*	*	506	113	149	32	**-2.0**	*0.077*	
201303_at	EIF4A3: eukaryotic translation initiation factor 4A, isoform 3	NM_014740	494	28	94	26	**-3.6**	*0.000*	*	94	26	407	88	**2.4**	*0.061*	
201386_s_at	DHX15: DEAH (Asp-Glu-Ala-His) box polypeptide 15	AF279891	292	35	41	8	**-4.9**	*0.015*	*	41	8	676	119	**10.6**	*0.033*	*
201488_x_at	KHDRBS1: KH domain containing, RNA binding, signal transduction associated 1	BC000717	663	25	261	69	**-1.8**	*0.019*	*	261	69	64	19	**-2.0**	*0.096*	
201586_s_at	SFPQ: splicing factor proline/glutamine-rich (polypyrimidine tract binding protein associated)	NM_005066	155	22	13	5	**-6.9**	*0.020*	*	13	5	132	34	**4.9**	*0.070*	
201698_s_at	SFRS9: splicing factor, arginine/serine-rich 9	NM_003769	820	29	19	8	**-24.6**	*0.001*	*	19	8	91	19	**2.4**	*0.048*	*
201742_x_at	SFRS1: splicing factor, arginine/serine-rich 1 (splicing factor 2, alternate splicing factor)	NM_006924	311	35	95	43	**-1.8**	*0.019*	*	95	43	125	22	**0.7**	*0.585*	
202690_s_at	SNRPD1: small nuclear ribonucleoprotein D1 polypeptide 16kDa	BC001721	64	6	11	5	**-3.1**	*0.002*	*	11	5	40	11	**1.5**	*0.108*	
202736_s_at	LSM4: LSM4 homolog, U6 small nuclear RNA associated (S. cerevisiae)	AA112507	129	7	25	6	**-3.6**	*0.000*	*	25	6	26	6	**0.6**	*0.917*	
202899_s_at	SFRS3: splicing factor, arginine/serine-rich 3	NM_003017	745	75	42	16	**-10.7**	*0.009*	*	42	16	80	34	**0.6**	*0.377*	
203316_s_at	SNRPE: small nuclear ribonucleoprotein polypeptide E	NM_003094	270	33	82	11	**-2.5**	*0.022*	*	82	11	671	94	**5.9**	*0.023*	*
203378_at	PCF11: PCF11, cleavage and polyadenylation factor subunit, homolog (S. cerevisiae)	AB020631	593	17	167	14	**-3.1**	*0.000*	*	167	14	86	13	**-1.5**	*0.014*	
204064_at	THOC1: THO complex 1	NM_005131	352	21	126	24	**-2.1**	*0.002*	*	126	24	150	23	**0.8**	*0.497*	
204299_at	FUSIP1 /// LOC727922: FUS interacting protein (serine/arginine-rich) 1 /// similar to FUS-int	NM_021993	102	13	23	15	**-2.1**	*0.017*	*	23	15	233	17	**4.8**	*0.001*	*
204559_s_at	LSM7: LSM7 homolog, U6 small nuclear RNA associated (S. cerevisiae)	NM_016199	200	13	50	17	**-2.5**	*0.003*	*	50	17	29	6	**-0.7**	*0.344*	
204658_at	TRA2A: transformer-2 alpha	NM_013293	119	10	49	16	**-1.5**	*0.027*	*	49	16	57	6	**0.7**	*0.667*	
205292_s_at	HNRPA2B1: heterogeneous nuclear ribonucleoprotein A2/B1	NM_002137	1154	88	79	23	**-9.7**	*0.004*	*	79	23	604	63	**5.0**	*0.008*	*
206051_at	ELAVL4: ELAV (embryonic lethal, abnormal vision, Drosophila)-like 4 (Hu antigen D)	NM_021952	334	50	85	24	**-2.4**	*0.022*	*	85	24	96	20	**0.6**	*0.741*	
206052_s_at	SLBP: stem-loop (histone) binding protein	NM_006527	91	15	17	5	**-3.3**	*0.028*	*	17	5	214	30	**8.0**	*0.021*	*
206989_s_at	SFRS2IP: splicing factor, arginine/serine-rich 2, interacting protein	NM_004719	146	10	55	16	**-1.8**	*0.012*	*	55	16	114	23	**1.2**	*0.107*	
207830_s_at	PPP1R8: protein phosphatase 1, regulatory (inhibitor) subunit 8	NM_002713	115	10	54	11	**-1.5**	*0.016*	*	54	11	121	18	**1.5**	*0.046*	
208174_x_at	ZRSR2: zinc finger (CCCH type), RNA-binding motif and serine/arginine rich 2	NM_005089	500	36	219	61	**-1.5**	*0.025*	*	219	61	142	20	**-0.8**	*0.337*	
208627_s_at	YBX1: Y box binding protein 1	BE966374	1567	326	58	12	**-16.2**	*0.043*	*	58	12	65	7	**0.8**	*0.652*	
208765_s_at	HNRPR: heterogeneous nuclear ribonucleoprotein R	NM_005826	361	34	46	19	**-4.5**	*0.003*	*	46	19	53	18	**0.4**	*0.811*	
208821_at	SNRPB: small nuclear ribonucleoprotein polypeptides B and B1	J04564	249	13	67	14	**-2.8**	*0.001*	*	67	14	60	12	**-0.7**	*0.715*	
208879_x_at	PRPF6: PRP6 pre-mRNA processing factor 6 homolog (S. cerevisiae)	BG469030	140	7	39	19	**-2.0**	*0.021*	*	39	19	25	6	**-0.3**	*0.527*	
209024_s_at	SYNCRIP: synaptotagmin binding, cytoplasmic RNA interacting protein	AI472757	137	10	34	8	**-2.9**	*0.002*	*	34	8	27	6	**-0.7**	*0.523*	
209055_s_at	CDC5L: CDC5 cell division cycle 5-like (S. pombe)	AW268817	99	5	25	7	**-2.7**	*0.002*	*	25	7	25	4	**0.6**	*0.985*	
209162_s_at	PRPF4: PRP4 pre-mRNA processing factor 4 homolog (yeast)	U82756	169	6	53	14	**-2.2**	*0.008*	*	53	14	56	9	**0.7**	*0.882*	
209388_at	PAPOLA: poly(A) polymerase alpha	BC000927	199	13	87	22	**-1.6**	*0.017*	*	87	22	620	96	**4.5**	*0.026*	*
210092_at	MAGOH: mago-nashi homolog, proliferation-associated (Drosophila)	AF067173	87	6	18	6	**-3.1**	*0.001*	*	18	6	59	9	**2.0**	*0.025*	
210183_x_at	g6563229	AF112222	3506	112	281	84	**-8.3**	*0.000*	*	281	84	60	75	**-1.3**	*0.120*	
211090_s_at	PRPF4B: PRP4 pre-mRNA processing factor 4 homolog B (yeast)	Z25435	92	5	21	5	**-3.2**	*0.000*	*	21	5	20	3	**-0.6**	*0.882*	
211747_s_at	LSM5: LSM5 homolog, U6 small nuclear RNA associated (S. cerevisiae)	BC005938	130	8	45	16	**-1.8**	*0.019*	*	45	16	127	25	**1.5**	*0.062*	
211933_s_at	HNRPA3 /// HNRPA3P1: heterogeneous nuclear ribonucleoprotein A3 pseudogene 1 /// hete	AA528233	351	36	134	28	**-1.9**	*0.010*	*	134	28	571	81	**2.9**	*0.023*	*
212016_s_at	PTBP1: polypyrimidine tract binding protein 1	AA679988	141	12	42	14	**-2.1**	*0.006*	*	42	14	33	11	**-0.5**	*0.640*	
212266_s_at	SFRS5: splicing factor, arginine/serine-rich 5	AW084582	1962	305	344	218	**-2.6**	*0.015*	*	344	218	542	180	**0.5**	*0.524*	
212438_at	RY1: putative nucleic acid binding protein RY-1	BG252325	270	17	47	11	**-4.1**	*0.001*	*	47	11	142	30	**1.8**	*0.072*	
212721_at	SFRS12: splicing factor, arginine/serine-rich 12	AI810380	119	12	45	10	**-1.9**	*0.011*	*	45	10	89	14	**1.3**	*0.066*	
213356_x_at	hCG_2023776 /// HNRPA1 /// HNRPA1L-2 /// HNRPA1P4 /// HNRPA1P5 /// LOC391670 /// L	AL568186	5170	202	2055	238	**-2.1**	*0.001*	*	2055	238	5050	395	**2.0**	*0.006*	*
213619_at	HNRPH1: Heterogeneous nuclear ribonucleoprotein H1 (H)	AV753392	632	64	79	27	**-5.0**	*0.006*	*	79	27	180	42	**1.2**	*0.122*	
213687_s_at	RPL35A: ribosomal protein L35a	BE968801	2041	108	377	87	**-3.9**	*0.000*	*	377	87	1172	327	**1.6**	*0.128*	
213729_at	PRPF40A: PRP40 pre-mRNA processing factor 40 homolog A (S. cerevisiae)	Z78308	225	13	61	27	**-2.1**	*0.013*	*	61	27	36	8	**-0.5**	*0.450*	
213762_x_at	RBMX: RNA binding motif protein, X-linked	AI452524	363	18	97	26	**-2.6**	*0.002*	*	97	26	301	60	**1.8**	*0.059*	
214882_s_at	SFRS2: splicing factor, arginine/serine-rich 2	BG254869	69	5	8	5	**-4.2**	*0.001*	*	8	5	39	11	**1.9**	*0.092*	
215157_x_at	PABPC1: poly(A) binding protein, cytoplasmic 1	AI734929	3248	172	108	31	**-20.2**	*0.002*	*	108	31	689	157	**3.5**	*0.061*	
215424_s_at	SNW1: SNW domain containing 1	AV689564	156	7	32	8	**-3.4**	*0.000*	*	32	8	62	13	**1.1**	*0.133*	
216559_x_at	hCG_2023776 /// HNRPA1 /// HNRPA1L-2 /// HNRPA1P4 /// LOC120364 /// LOC344741 ///	AL050348	234	23	36	15	**-3.7**	*0.003*	*	36	15	231	57	**3.1**	*0.066*	
217840_at	DDX41: DEAD (Asp-Glu-Ala-Asp) box polypeptide 41	NM_016222	144	9	65	17	**-1.5**	*0.024*	*	65	17	47	7	**-0.8**	*0.401*	
219085_s_at	GEMIN7: gem (nuclear organelle) associated protein 7	NM_024707	242	29	108	22	**-1.6**	*0.023*	*	108	22	52	11	**-1.2**	*0.111*	
221263_s_at	SF3B5: splicing factor 3b, subunit 5, 10kDa	NM_031287	405	15	95	59	**-2.1**	*0.028*	*	95	59	75	22	**0.0**	*0.780*	

19 Gene Ontology 'nuclear mRNA splicing, via spliceosome' genes in 2122 annotated genes (genome-wide: 42/11929, p-value: 0.000281) ****

200000_s_at	PRPF8: PRP8 pre-mRNA processing factor 8 homolog (S. cerevisiae)	NM_006445	565	24	43	31	**-6.0**	*0.000*	*	43	31	36	22	**0.0**	*0.864*	
200014_s_at	HNRPC: heterogeneous nuclear ribonucleoprotein C (C1/C2)	NM_004500	590	83	194	71	**-1.8**	*0.023*	*	194	71	1171	206	**3.4**	*0.031*	*
200016_x_at	HNRPA1: heterogeneous nuclear ribonucleoprotein A1	NM_002136	4871	249	1840	267	**-2.1**	*0.001*	*	1840	267	4608	384	**1.9**	*0.006*	*
200041_s_at	BAT1: HLA-B associated transcript 1	NM_004640	390	28	27	9	**-9.1**	*0.003*	*	27	9	34	9	**0.6**	*0.620*	
200060_s_at	RNPS1: RNA binding protein S1, serine-rich domain	BC001659	778	31	325	23	**-2.1**	*0.000*	*	325	23	639	83	**1.5**	*0.055*	
200619_at	SF3B2: splicing factor 3b, subunit 2, 145kDa	NM_006842	244	25	106	21	**-1.6**	*0.015*	*	106	21	132	24	**0.8**	*0.465*	
200892_s_at	SFRS10: splicing factor, arginine/serine-rich 10 (transformer 2 homolog, Drosophila)	BC000451	672	23	40	13	**-10.9**	*0.000*	*	40	13	171	32	**2.4**	*0.042*	*
201070_x_at	SF3B1: splicing factor 3b, subunit 1, 155kDa	AI739389	371	17	96	15	**-3.0**	*0.000*	*	96	15	67	11	**-1.0**	*0.199*	
204299_at	FUSIP1 /// LOC727922: FUS interacting protein (serine/arginine-rich) 1 /// similar to FUS-int	NM_021993	102	13	23	15	**-2.1**	*0.017*	*	23	15	233	17	**4.8**	*0.001*	*
204559_s_at	LSM7: LSM7 homolog, U6 small nuclear RNA associated (S. cerevisiae)	NM_016199	200	13	50	17	**-2.5**	*0.003*	*	50	17	29	6	**-0.7**	*0.344*	
204658_at	TRA2A: transformer-2 alpha	NM_013293	119	10	49	16	**-1.5**	*0.027*	*	49	16	57	6	**0.7**	*0.667*	
205292_s_at	HNRPA2B1: heterogeneous nuclear ribonucleoprotein A2/B1	NM_002137	1154	88	79	23	**-9.7**	*0.004*	*	79	23	604	63	**5.0**	*0.008*	*
208821_at	SNRPB: small nuclear ribonucleoprotein polypeptides B and B1	J04564	249	13	67	14	**-2.8**	*0.001*	*	67	14	60	12	**-0.7**	*0.715*	
208879_x_at	PRPF6: PRP6 pre-mRNA processing factor 6 homolog (S. cerevisiae)	BG469030	140	7	39	19	**-2.0**	*0.021*	*	39	19	25	6	**-0.3**	*0.527*	
213356_x_at	hCG_2023776 /// HNRPA1 /// HNRPA1L-2 /// HNRPA1P4 /// HNRPA1P5 /// LOC391670 /// L	AL568186	5170	202	2055	238	**-2.1**	*0.001*	*	2055	238	5050	395	**2.0**	*0.006*	*
213687_s_at	RPL35A: ribosomal protein L35a	BE968801	2041	108	377	87	**-3.9**	*0.000*	*	377	87	1172	327	**1.6**	*0.128*	
215424_s_at	SNW1: SNW domain containing 1	AV689564	156	7	32	8	**-3.4**	*0.000*	*	32	8	62	13	**1.1**	*0.133*	
216559_x_at	hCG_2023776 /// HNRPA1 /// HNRPA1L-2 /// HNRPA1P4 /// LOC120364 /// LOC344741 ///	AL050348	234	23	36	15	**-3.7**	*0.003*	*	36	15	231	57	**3.1**	*0.066*	
219085_s_at	GEMIN7: gem (nuclear organelle) associated protein 7	NM_024707	242	29	108	22	**-1.6**	*0.023*	*	108	22	52	11	**-1.2**	*0.111*	

7 Gene Ontology 'pentose-phosphate shunt' genes in 2122 annotated genes (genome-wide: 7/11929, p-value: 0.000311) ****

200822_x_at	TPI1: triosephosphate isomerase 1	NM_000365	1674	68	653	176	**-1.8**	*0.018*	*	653	176	506	78	**-0.7**	*0.504*	
201118_at	PGD: phosphogluconate dehydrogenase	NM_002631	246	7	48	10	**-3.8**	*0.000*	*	48	10	39	5	**-0.8**	*0.480*	
201463_s_at	TALDO1: transaldolase 1	NM_006755	173	19	36	16	**-2.7**	*0.006*	*	36	16	46	13	**0.6**	*0.645*	
202275_at	G6PD: glucose-6-phosphate dehydrogenase	NM_000402	144	7	46	10	**-2.3**	*0.002*	*	46	10	30	5	**-0.9**	*0.248*	
218388_at	PGLS: 6-phosphogluconolactonase	NM_012088	167	25	55	16	**-1.9**	*0.025*	*	55	16	31	6	**-0.9**	*0.278*	
218506_x_at	N-PAC: cytokine-like nuclear factor n-pac	NM_018149	199	11	71	33	**-1.6**	*0.048*	*	71	33	100	11	**0.8**	*0.478*	
221531_at	WDR61: WD repeat domain 61	AF309553	102	5	23	4	**-3.5**	*0.001*	*	23	4	117	16	**3.6**	*0.024*	*

20 Gene Ontology 'proteasome complex (sensu Eukaryota)' genes in 2122 annotated genes (genome-wide: 41/11929, p-value: 0.000074) *****

200039_s_at	PSMB2: proteasome (prosome, macropain) subunit, beta type, 2	NM_002794	408	21	101	20	**-3.0**	*0.000*	*	101	20	208	29	**1.4**	*0.045*	
200814_at	PSME1: proteasome (prosome, macropain) activator subunit 1 (PA28 alpha)	NM_006263	723	57	329	48	**-1.7**	*0.007*	*	329	48	309	72	**-0.7**	*0.830*	
200820_at	PSMD8: proteasome (prosome, macropain) 26S subunit, non-ATPase, 8	NM_002812	727	25	36	28	**-8.8**	*0.000*	*	36	28	70	25	**0.6**	*0.422*	
200876_s_at	PSMB1: proteasome (prosome, macropain) subunit, beta type, 1	NM_002793	1330	47	123	68	**-5.7**	*0.000*	*	123	68	576	61	**2.4**	*0.008*	*
200987_x_at	PSME3: proteasome (prosome, macropain) activator subunit 3 (PA28 gamma; Ki)	AA758755	167	18	76	9	**-1.7**	*0.022*	*	76	9	101	36	**0.5**	*0.572*	
201068_s_at	PSMC2: proteasome (prosome, macropain) 26S subunit, ATPase, 2	NM_002803	1170	101	526	96	**-1.6**	*0.010*	*	526	96	1566	86	**2.3**	*0.001*	*
201198_s_at	PSMD1: proteasome (prosome, macropain) 26S subunit, non-ATPase, 1	AI860431	635	24	60	14	**-7.6**	*0.000*	*	60	14	88	22	**0.8**	*0.359*	
201252_at	LOC652826 /// PSMC4: proteasome (prosome, macropain) 26S subunit, ATPase, 4 /// simila	NM_006503	230	13	48	18	**-2.9**	*0.002*	*	48	18	68	15	**0.7**	*0.452*	
201267_s_at	PSMC3: proteasome (prosome, macropain) 26S subunit, ATPase, 3	AL545523	241	15	56	10	**-3.3**	*0.001*	*	56	10	49	9	**-0.8**	*0.644*	
201274_at	PSMA5: proteasome (prosome, macropain) subunit, alpha type, 5	NM_002790	359	65	108	25	**-2.1**	*0.047*	*	108	25	614	107	**3.6**	*0.036*	*
201400_at	PSMB3: proteasome (prosome, macropain) subunit, beta type, 3	NM_002795	569	27	63	9	**-7.2**	*0.001*	*	63	9	113	17	**1.3**	*0.078*	
201532_at	PSMA3: proteasome (prosome, macropain) subunit, alpha type, 3	NM_002788	239	35	37	7	**-4.4**	*0.025*	*	37	7	363	26	**7.3**	*0.004*	*
201676_x_at	PSMA1: proteasome (prosome, macropain) subunit, alpha type, 1	NM_002786	1123	48	85	19	**-9.5**	*0.001*	*	85	19	758	74	**6.2**	*0.009*	*
201705_at	PSMD7: proteasome (prosome, macropain) 26S subunit, non-ATPase, 7 (Mov34 homolog)	NM_002811	256	19	90	28	**-1.8**	*0.012*	*	90	28	768	153	**4.8**	*0.044*	*
202243_s_at	PSMB4: proteasome (prosome, macropain) subunit, beta type, 4	NM_002796	577	31	56	14	**-7.3**	*0.001*	*	56	14	400	60	**4.6**	*0.024*	*
208777_s_at	PSMD11: proteasome (prosome, macropain) 26S subunit, non-ATPase, 11	AF001212	311	19	45	14	**-4.6**	*0.001*	*	45	14	47	10	**0.6**	*0.893*	
209040_s_at	PSMB8: proteasome (prosome, macropain) subunit, beta type, 8 (large multifunctional pepti	U17496	159	12	45	8	**-2.6**	*0.002*	*	45	8	54	9	**0.8**	*0.523*	
209503_s_at	PSMC5: proteasome (prosome, macropain) 26S subunit, ATPase, 5	AF035309	193	13	65	20	**-2.0**	*0.008*	*	65	20	115	21	**1.0**	*0.151*	
210460_s_at	PSMD4: proteasome (prosome, macropain) 26S subunit, non-ATPase, 4	AB033605	540	36	51	12	**-7.5**	*0.003*	*	51	12	79	16	**0.9**	*0.251*	
219485_s_at	PSMD10: proteasome (prosome, macropain) 26S subunit, non-ATPase, 10	NM_002814	156	20	65	18	**-1.5**	*0.029*	*	65	18	487	98	**4.3**	*0.046*	*

16 Gene Ontology 'regulation of translational initiation' genes in 2122 annotated genes (genome-wide: 30/11929, p-value: 0.000140) ****

200004_at	EIF4G2: eukaryotic translation initiation factor 4 gamma, 2	NM_001418	3224	145	618	112	**-4.0**	*0.000*	*	618	112	1497	177	**1.7**	*0.019*	*
200005_at	EIF3S7: eukaryotic translation initiation factor 3, subunit 7 zeta, 66/67kDa	NM_003753	507	23	131	45	**-2.5**	*0.005*	*	131	45	436	28	**2.1**	*0.008*	*
200023_s_at	EIF3S5: eukaryotic translation initiation factor 3, subunit 5 epsilon, 47kDa	NM_003754	789	90	238	66	**-2.2**	*0.010*	*	238	66	562	150	**1.2**	*0.151*	

200596_s_at	EIF3S10: eukaryotic translation initiation factor 3, subunit 10 theta, 150/170kDa	BE614908	468	24	72	25	**-4.1**	*0.000*	*	72	25	242	52	**1.8**	*0.063*	
200776_s_at	BZW1 /// LOC151579: basic leucine zipper and W2 domains 1 /// similar to basic leucine zip	AL518328	725	73	80	18	**-6.3**	*0.009*	*	80	18	213	45	**1.6**	*0.081*	
201122_x_at	EIF5A: eukaryotic translation initiation factor 5A	BC000751	799	62	200	29	**-3.1**	*0.004*	*	200	29	116	25	**-1.2**	*0.089*	
201592_at	EIF3S3: eukaryotic translation initiation factor 3, subunit 3 gamma, 40kDa	NM_003756	1454	105	628	113	**-1.7**	*0.006*	*	628	113	1194	195	**1.3**	*0.082*	
201841_s_at	HSPB1: heat shock 27kDa protein 1	NM_001540	1481	85	35	18	**-22.3**	*0.002*	*	35	18	46	12	**0.6**	*0.674*	
202021_x_at	EIF1: eukaryotic translation initiation factor 1	AF083441	4086	494	162	43	**-16.5**	*0.015*	*	162	43	1571	367	**5.3**	*0.060*	
202461_at	EIF2B2: eukaryotic translation initiation factor 2B, subunit 2 beta, 39kDa	NM_014239	163	13	70	10	**-1.8**	*0.006*	*	70	10	163	24	**1.6**	*0.044*	*
208290_s_at	EIF5: eukaryotic translation initiation factor 5	NM_001969	103	6	23	4	**-3.4**	*0.001*	*	23	4	71	13	**2.0**	*0.054*	
208625_s_at	EIF4G1: eukaryotic translation initiation factor 4 gamma, 1	AF104913	178	14	26	13	**-3.7**	*0.001*	*	26	13	18	6	**-0.2**	*0.647*	
208756_at	EIF3S2: eukaryotic translation initiation factor 3, subunit 2 beta, 36kDa	U36764	473	15	62	12	**-5.8**	*0.000*	*	62	12	174	28	**1.9**	*0.041*	*
208887_at	EIF3S4: eukaryotic translation initiation factor 3, subunit 4 delta, 44kDa	BC000733	385	43	85	22	**-3.0**	*0.009*	*	85	22	82	14	**-0.6**	*0.896*	
210949_s_at	EIF3S8 /// LOC728689: eukaryotic translation initiation factor 3, subunit 8, 110kDa /// similar	BC000533	1573	158	66	26	**-14.1**	*0.009*	*	66	26	120	34	**0.8**	*0.282*	
211937_at	EIF4B: eukaryotic translation initiation factor 4B	NM_001417	1849	123	825	124	**-1.8**	*0.004*	*	825	124	560	167	**-0.9**	*0.277*	

65 Gene Ontology 'RNA splicing' genes in 2122 annotated genes (genome-wide: 183/11929, p-value: 0.000000) *****

200000_s_at	PRPF8: PRP8 pre-mRNA processing factor 8 homolog (S. cerevisiae)	NM_006445	565	24	43	31	**-6.0**	*0.000*	*	43	31	36	22	**0.0**	*0.864*	
200014_s_at	HNRPC: heterogeneous nuclear ribonucleoprotein C (C1/C2)	NM_004500	590	83	194	71	**-1.8**	*0.023*	*	194	71	1171	206	**3.4**	*0.031*	*
200016_x_at	HNRPA1: heterogeneous nuclear ribonucleoprotein A1	NM_002136	4871	249	1840	267	**-2.1**	*0.001*	*	1840	267	4608	384	**1.9**	*0.006*	*
200033_at	DDX5: DEAD (Asp-Glu-Ala-Asp) box polypeptide 5	NM_004396	1853	127	764	255	**-1.5**	*0.033*	*	764	255	1432	98	**1.2**	*0.106*	
200041_s_at	BAT1: HLA-B associated transcript 1	NM_004640	390	28	27	9	**-9.1**	*0.003*	*	27	9	34	9	**0.6**	*0.620*	
200057_s_at	NONO: non-POU domain containing, octamer-binding	NM_007363	2242	174	713	168	**-2.2**	*0.003*	*	713	168	1103	183	**1.0**	*0.192*	
200060_s_at	RNPS1: RNA binding protein S1, serine-rich domain	BC001659	778	31	325	23	**-2.1**	*0.000*	*	325	23	639	83	**1.5**	*0.055*	
200097_s_at	HNRPK: heterogeneous nuclear ribonucleoprotein K	AI701949	628	35	31	15	**-11.3**	*0.001*	*	31	15	108	39	**1.2**	*0.177*	
200593_s_at	HNRPU: heterogeneous nuclear ribonucleoprotein U (scaffold attachment factor A)	BC003621	722	116	226	124	**-1.6**	*0.044*	*	226	124	997	88	**2.3**	*0.009*	*
200619_at	SF3B2: splicing factor 3b, subunit 2, 145kDa	NM_006842	244	25	106	21	**-1.6**	*0.015*	*	106	21	132	24	**0.8**	*0.465*	
200695_at	PPP2R1A: protein phosphatase 2 (formerly 2A), regulatory subunit A , alpha isoform	NM_014225	1050	24	32	23	**-15.0**	*0.000*	*	32	23	32	15	**0.2**	*0.987*	
200826_at	SNRPD2: small nuclear ribonucleoprotein D2 polypeptide 16.5kDa	NM_004597	1295	46	51	23	**-14.6**	*0.000*	*	51	23	55	21	**0.4**	*0.890*	
200892_s_at	SFRS10: splicing factor, arginine/serine-rich 10 (transformer 2 homolog, Drosophila)	BC000451	672	23	40	13	**-10.9**	*0.000*	*	40	13	171	32	**2.4**	*0.042*	*
201070_x_at	SF3B1: splicing factor 3b, subunit 1, 155kDa	AI739389	371	17	96	15	**-3.0**	*0.000*	*	96	15	67	11	**-1.0**	*0.199*	
201077_s_at	NHP2L1: NHP2 non-histone chromosome protein 2-like 1 (S. cerevisiae)	AF155235	1379	73	273	64	**-3.6**	*0.000*	*	273	64	496	131	**1.0**	*0.228*	
201224_s_at	SRRM1: serine/arginine repetitive matrix 1	AU147713	1278	75	506	113	**-1.8**	*0.007*	*	506	113	149	32	**-2.0**	*0.077*	
201303_at	EIF4A3: eukaryotic translation initiation factor 4A, isoform 3	NM_014740	494	28	94	26	**-3.6**	*0.000*	*	94	26	407	88	**2.4**	*0.061*	
201342_at	SNRPC: small nuclear ribonucleoprotein polypeptide C	NM_003093	1620	47	533	84	**-2.4**	*0.001*	*	533	84	337	38	**-1.1**	*0.130*	
201386_s_at	DHX15: DEAH (Asp-Glu-Ala-His) box polypeptide 15	AF279891	292	35	41	8	**-4.9**	*0.015*	*	41	8	676	119	**10.6**	*0.033*	*
201517_at	NCBP2: nuclear cap binding protein subunit 2, 20kDa	BC001255	99	13	39	8	**-1.7**	*0.021*	*	39	8	101	22	**1.5**	*0.092*	
201586_s_at	SFPQ: splicing factor proline/glutamine-rich (polypyrimidine tract binding protein associated)	NM_005066	155	22	13	5	**-6.9**	*0.020*	*	13	5	132	34	**4.9**	*0.070*	
201698_s_at	SFRS9: splicing factor, arginine/serine-rich 9	NM_003769	820	29	19	8	**-24.6**	*0.001*	*	19	8	91	19	**2.4**	*0.048*	*
201742_x_at	SFRS1: splicing factor, arginine/serine-rich 1 (splicing factor 2, alternate splicing factor)	NM_006924	311	35	95	43	**-1.8**	*0.019*	*	95	43	125	22	**0.7**	*0.585*	
202690_s_at	SNRPD1: small nuclear ribonucleoprotein D1 polypeptide 16kDa	BC001721	64	6	11	5	**-3.1**	*0.002*	*	11	5	40	11	**1.5**	*0.108*	
202736_s_at	LSM4: LSM4 homolog, U6 small nuclear RNA associated (S. cerevisiae)	AA112507	129	7	25	6	**-3.6**	*0.000*	*	25	6	26	6	**0.6**	*0.917*	
202884_s_at	PPP2R1B: protein phosphatase 2 (formerly 2A), regulatory subunit A, beta isoform	NM_002716	129	11	46	13	**-1.9**	*0.008*	*	46	13	29	6	**-0.8**	*0.325*	
202899_s_at	SFRS3: splicing factor, arginine/serine-rich 3	NM_003017	745	75	42	16	**-10.7**	*0.009*	*	42	16	80	34	**0.6**	*0.377*	
203316_s_at	SNRPE: small nuclear ribonucleoprotein polypeptide E	NM_003094	270	33	82	11	**-2.5**	*0.022*	*	82	11	671	94	**5.9**	*0.023*	*
204064_at	THOC1: THO complex 1	NM_005131	352	21	126	24	**-2.1**	*0.002*	*	126	24	150	23	**0.8**	*0.497*	
204299_at	FUSIP1 /// LOC727922: FUS interacting protein (serine/arginine-rich) 1 /// similar to FUS-int	NM_021993	102	13	23	15	**-2.1**	*0.017*	*	23	15	233	17	**4.8**	*0.001*	*
204559_s_at	LSM7: LSM7 homolog, U6 small nuclear RNA associated (S. cerevisiae)	NM_016199	200	13	50	17	**-2.5**	*0.003*	*	50	17	29	6	**-0.7**	*0.344*	
204658_at	TRA2A: transformer-2 alpha	NM_013293	119	10	49	16	**-1.5**	*0.027*	*	49	16	57	6	**0.7**	*0.667*	
205292_s_at	HNRPA2B1: heterogeneous nuclear ribonucleoprotein A2/B1	NM_002137	1154	88	79	23	**-9.7**	*0.004*	*	79	23	604	63	**5.0**	*0.008*	*
206042_x_at	SNRPN /// SNURF: small nuclear ribonucleoprotein polypeptide N /// SNRPN upstream reac	NM_022804	1682	87	87	63	**-8.8**	*0.000*	*	87	63	237	107	**0.6**	*0.306*	
206989_s_at	SFRS2IP: splicing factor, arginine/serine-rich 2, interacting protein	NM_004719	146	10	55	16	**-1.8**	*0.012*	*	55	16	114	23	**1.2**	*0.107*	
207830_s_at	PPP1R8: protein phosphatase 1, regulatory (inhibitor) subunit 8	NM_002713	115	10	54	11	**-1.5**	*0.016*	*	54	11	121	18	**1.5**	*0.046*	
208174_x_at	ZRSR2: zinc finger (CCCH type), RNA-binding motif and serine/arginine rich 2	NM_005089	500	36	219	61	**-1.5**	*0.025*	*	219	61	142	20	**-0.8**	*0.337*	
208627_s_at	YBX1: Y box binding protein 1	BE966374	1567	326	58	12	**-16.2**	*0.043*	*	58	12	65	7	**0.8**	*0.652*	
208765_s_at	HNRPR: heterogeneous nuclear ribonucleoprotein R	NM_005826	361	34	46	19	**-4.5**	*0.003*	*	46	19	53	18	**0.4**	*0.811*	
208821_at	SNRPB: small nuclear ribonucleoprotein polypeptides B and B1	J04564	249	13	67	14	**-2.8**	*0.001*	*	67	14	60	12	**-0.7**	*0.715*	
208879_x_at	PRPF6: PRP6 pre-mRNA processing factor 6 homolog (S. cerevisiae)	BG469030	140	7	39	19	**-2.0**	*0.021*	*	39	19	25	6	**-0.3**	*0.527*	
209024_s_at	SYNCRIP: synaptotagmin binding, cytoplasmic RNA interacting protein	AI472757	137	10	34	8	**-2.9**	*0.002*	*	34	8	27	6	**-0.7**	*0.523*	
209055_s_at	CDC5L: CDC5 cell division cycle 5-like (S. pombe)	AW268817	99	5	25	7	**-2.7**	*0.002*	*	25	7	25	4	**0.6**	*0.985*	
209162_s_at	PRPF4: PRP4 pre-mRNA processing factor 4 homolog (yeast)	U82756	169	6	53	14	**-2.2**	*0.008*	*	53	14	56	9	**0.7**	*0.882*	
210092_at	MAGOH: mago-nashi homolog, proliferation-associated (Drosophila)	AF067173	87	6	18	6	**-3.1**	*0.001*	*	18	6	59	9	**2.0**	*0.025*	
210183_x_at	g6563229	AF112222	3506	112	281	84	**-8.3**	*0.000*	*	281	84	60	75	**-1.3**	*0.120*	
211090_s_at	PRPF4B: PRP4 pre-mRNA processing factor 4 homolog B (yeast)	Z25435	92	5	21	5	**-3.2**	*0.000*	*	21	5	20	3	**-0.6**	*0.882*	
211747_s_at	LSM5: LSM5 homolog, U6 small nuclear RNA associated (S. cerevisiae)	BC005938	130	8	45	16	**-1.8**	*0.019*	*	45	16	127	25	**1.5**	*0.062*	
211933_s_at	HNRPA3 /// HNRPA3P1: heterogeneous nuclear ribonucleoprotein A3 pseudogene 1 /// hete	AA528233	351	36	134	28	**-1.9**	*0.010*	*	134	28	571	81	**2.9**	*0.023*	*
212016_s_at	PTBP1: polypyrimidine tract binding protein 1	AA679988	141	12	42	14	**-2.1**	*0.006*	*	42	14	33	11	**-0.5**	*0.640*	
212266_s_at	SFRS5: splicing factor, arginine/serine-rich 5	AW084582	1962	305	344	218	**-2.6**	*0.015*	*	344	218	542	180	**0.5**	*0.524*	
212438_at	RY1: putative nucleic acid binding protein RY-1	BG252325	270	17	47	11	**-4.1**	*0.001*	*	47	11	142	30	**1.8**	*0.072*	
212721_at	SFRS12: splicing factor, arginine/serine-rich 12	AI810380	119	12	45	10	**-1.9**	*0.011*	*	45	10	89	14	**1.3**	*0.066*	
213356_x_at	hCG_2023776 /// HNRPA1 /// HNRPA1L-2 /// HNRPA1P4 /// HNRPA1P5 /// LOC391670 /// I	AL568186	5170	202	2055	238	**-2.1**	*0.001*	*	2055	238	5050	395	**2.0**	*0.006*	*
213619_at	HNRPH1: Heterogeneous nuclear ribonucleoprotein H1 (H)	AV753392	632	64	79	27	**-5.0**	*0.006*	*	79	27	180	42	**1.2**	*0.122*	
213687_s_at	RPL35A: ribosomal protein L35a	BE968801	2041	108	377	87	**-3.9**	*0.000*	*	377	87	1172	327	**1.6**	*0.128*	
213729_at	PRPF40A: PRP40 pre-mRNA processing factor 40 homolog A (S. cerevisiae)	Z78308	225	13	61	27	**-2.1**	*0.013*	*	61	27	36	8	**-0.5**	*0.450*	
213762_x_at	RBMX: RNA binding motif protein, X-linked	AI452524	363	18	97	26	**-2.6**	*0.002*	*	97	26	301	60	**1.8**	*0.059*	
214882_s_at	SFRS2: splicing factor, arginine/serine-rich 2	BG254869	69	5	8	5	**-4.2**	*0.001*	*	8	5	39	11	**1.9**	*0.092*	
215157_x_at	PABPC1: poly(A) binding protein, cytoplasmic 1	AI734929	3248	172	108	31	**-20.2**	*0.002*	*	108	31	689	157	**3.5**	*0.061*	
215424_s_at	SNW1: SNW domain containing 1	AV689564	156	7	32	8	**-3.4**	*0.000*	*	32	8	62	13	**1.1**	*0.133*	
216559_x_at	hCG_2023776 /// HNRPA1 /// HNRPA1L-2 /// HNRPA1P4 /// LOC120364 /// LOC344741 ///	AL050348	234	23	36	15	**-3.7**	*0.003*	*	36	15	231	57	**3.1**	*0.066*	
217840_at	DDX41: DEAD (Asp-Glu-Ala-Asp) box polypeptide 41	NM_016222	144	9	65	17	**-1.5**	*0.024*	*	65	17	47	7	**-0.8**	*0.401*	
219085_s_at	GEMIN7: gem (nuclear organelle) associated protein 7	NM_024707	242	29	108	22	**-1.6**	*0.023*	*	108	22	52	11	**-1.2**	*0.111*	
221263_s_at	SF3B5: splicing factor 3b, subunit 5, 10kDa	NM_031287	405	15	95	59	**-2.1**	*0.028*	*	95	59	75	22	**0.0**	*0.780*	

56 Gene Ontology 'small GTPase mediated signal transduction' genes in 2122 annotated genes (genome-wide: 178/11929, p-value: 0.000051) *****

200059_s_at	RHOA: ras homolog gene family, member A	BC001360	2529	62	286	106	**-5.5**	*0.000*	*	286	106	637	93	**1.3**	*0.069*	
31846_at	RHOD: ras homolog gene family, member D	AW003733	1173	45	512	60	**-1.9**	*0.001*	*	512	60	268	65	**-1.3**	*0.051*	
34031_i_at	KRIT1: KRIT1, ankyrin repeat containing	U90269	64	3	11	3	**-4.0**	*0.000*	*	11	3	7	2	**-0.9**	*0.243*	
396_f_at	EPOR: erythropoietin receptor	X97671	1201	67	447	84	**-2.0**	*0.003*	*	447	84	221	41	**-1.3**	*0.097*	
200693_at	YWHAQ: tyrosine 3-monooxygenase/tryptophan 5-monooxygenase activation protein, theta	NM_006826	2579	108	851	119	**-2.4**	*0.000*	*	851	119	1907	259	**1.6**	*0.038*	*
200750_s_at	RAN: RAN, member RAS oncogene family	AF054183	397	29	43	8	**-6.8**	*0.004*	*	43	8	283	40	**4.5**	*0.024*	*
200791_s_at	IQGAP1: IQ motif containing GTPase activating protein 1	NM_003870	180	20	31	9	**-3.7**	*0.008*	*	31	9	199	57	**3.0**	*0.095*	
200823_x_at	RPL29: ribosomal protein L29	NM_000992	1786	348	56	14	**-19.0**	*0.038*	*	56	14	37	5	**-0.9**	*0.309*	
200863_s_at	RAB11A: RAB11A, member RAS oncogene family	AI215102	2322	183	447	173	**-3.1**	*0.002*	*	447	173	1088	232	**1.3**	*0.097*	
200885_at	RHOC: ras homolog gene family, member C	NM_005167	235	35	82	19	**-1.9**	*0.030*	*	82	19	75	12	**-0.7**	*0.768*	
200927_s_at	RAB14: RAB14, member RAS oncogene family	AA919115	550	32	44	6	**-9.9**	*0.003*	*	44	6	233	42	**3.5**	*0.043*	*
201047_x_at	RAB6A: RAB6A, member RAS oncogene family	BC003617	511	20	259	31	**-1.6**	*0.004*	*	259	31	302	75	**0.7**	*0.635*	
201096_s_at	ARF4: ADP-ribosylation factor 4	AL537042	1067	40	107	68	**-4.8**	*0.001*	*	107	68	557	129	**2.2**	*0.053*	
201156_s_at	RAB5C: RAB5C, member RAS oncogene family	AF141304	396	67	89	22	**-2.8**	*0.034*	*	89	22	53	10	**-0.9**	*0.239*	
201526_at	ARF5: ADP-ribosylation factor 5	NM_001662	799	63	241	101	**-1.9**	*0.014*	*	241	101	103	29	**-0.7**	*0.302*	
201659_s_at	ARL1: ADP-ribosylation factor-like 1	NM_001177	247	27	81	15	**-2.2**	*0.012*	*	81	15	378	57	**3.2**	*0.029*	*
202044_at	GRLF1: glucocorticoid receptor DNA binding factor 1	AU159484	169	25	66	12	**-1.7**	*0.038*	*	66	12	46	6	**-1.0**	*0.246*	
202109_at	ARFIP2: ADP-ribosylation factor interacting protein 2 (arfaptin 2)	NM_012402	350	6	58	14	**-4.3**	*0.000*	*	58	14	48	8	**-0.7**	*0.539*	
202564_x_at	ARL2: ADP-ribosylation factor-like 2	NM_001667	135	8	50	11	**-2.0**	*0.005*	*	50	11	38	10	**-0.8**	*0.449*	
202845_s_at	RALBP1: ralA binding protein 1	NM_006788	181	20	68	15	**-1.9**	*0.012*	*	68	15	92	8	**1.0**	*0.247*	
203175_at	RHOG: ras homolog gene family, member G (rho G)	NM_001665	435	54	190	35	**-1.6**	*0.025*	*	190	35	91	26	**-1.2**	*0.089*	
203311_s_at	ARF6: ADP-ribosylation factor 6	M57763	179	4	71	10	**-2.0**	*0.003*	*	71	10	45	7	**-1.1**	*0.096*	
203885_at	RAB21: RAB21, member RAS oncogene family	NM_014999	131	15	54	9	**-1.8**	*0.019*	*	54	9	456	86	**5.4**	*0.042*	*
204214_s_at	RAB32: RAB32, member RAS oncogene family	NM_006834	186	18	90	16	**-1.5**	*0.016*	*	90	16	160	38	**1.0**	*0.193*	
204472_at	GEM: GTP binding protein overexpressed in skeletal muscle	NM_005261	525	110	37	9	**-8.3**	*0.046*	*	37	9	1731	364	**27.4**	*0.043*	*
205924_at	RAB3B: RAB3B, member RAS oncogene family	BC005035	227	23	41	10	**-3.7**	*0.006*	*	41	10	31	9	**-0.7**	*0.521*	
206113_s_at	RAB5A: RAB5A, member RAS oncogene family	NM_004162	113	11	27	6	**-2.9**	*0.006*	*	27	6	23	4	**-0.7**	*0.599*	
206152_at	CENTG1: centaurin, gamma 1	NM_014770	217	17	108	15	**-1.6**	*0.009*	*	108	15	85	24	**-0.8**	*0.474*	
207791_s_at	RAB1A: RAB1A, member RAS oncogene family	NM_004161	340	54	10	4	**-19.3**	*0.025*	*	10	4	36	7	**2.0**	*0.047*	*

208640_at	RAC1: ras-related C3 botulinum toxin substrate 1 (rho family, small GTP binding protein Ra	BG292367	2768	223	1405	156	**-1.6**	*0.010*	*	1405	156	3680	348	**2.1**	*0.012*	*
208727_s_at	CDC42: cell division cycle 42 (GTP binding protein, 25kDa)	BC002711	791	160	144	54	**-2.9**	*0.045*	*	144	54	208	48	**0.7**	*0.425*	
208734_x_at	RAB2A: RAB2A, member RAS oncogene family	M28213	547	25	97	45	**-3.2**	*0.003*	*	97	45	172	53	**0.7**	*0.343*	
208750_s_at	ARF1: ADP-ribosylation factor 1	AA580004	1646	28	21	7	**-48.8**	*0.000*	*	21	7	21	6	**-0.4**	*0.949*	
209051_s_at	RALGDS: ral guanine nucleotide dissociation stimulator	AF295773	404	22	84	16	**-3.6**	*0.000*	*	84	16	51	9	**-1.0**	*0.173*	
209084_s_at	RAB28: RAB28, member RAS oncogene family	BE504689	70	7	10	5	**-3.6**	*0.003*	*	10	5	73	20	**3.0**	*0.078*	
209515_s_at	RAB27A: RAB27A, member RAS oncogene family	U38654	305	36	58	28	**-2.8**	*0.007*	*	58	28	370	85	**3.0**	*0.054*	
209684_at	RIN2: Ras and Rab interactor 2	AL136924	569	83	177	35	**-2.2**	*0.028*	*	177	35	501	41	**2.1**	*0.004*	*
210994_x_at	TRIM23: tripartite motif-containing 23	AF230398	145	20	40	11	**-2.4**	*0.002*	*	40	11	51	9	**0.8**	*0.494*	
211622_s_at	ARF3: ADP-ribosylation factor 3	M33384	235	21	66	17	**-2.4**	*0.004*	*	66	17	58	11	**-0.6**	*0.692*	
211665_s_at	SOS2: son of sevenless homolog 2 (Drosophila)	L20686	80	4	20	6	**-2.7**	*0.001*	*	20	6	33	8	**0.9**	*0.254*	
211960_s_at	RAB7A: RAB7A, member RAS oncogene family	BG261416	652	41	98	32	**-4.3**	*0.001*	*	98	32	164	47	**0.8**	*0.316*	
212099_at	RHOB: ras homolog gene family, member B	AI263909	1440	150	54	17	**-16.9**	*0.011*	*	54	17	97	42	**0.5**	*0.418*	
212117_at	RHOQ: ras homolog gene family, member Q	BF978689	463	47	82	26	**-3.6**	*0.005*	*	82	26	136	34	**0.9**	*0.280*	
212590_at	RRAS2: related RAS viral (r-ras) oncogene homolog 2	AI431643	113	22	34	14	**-1.8**	*0.049*	*	34	14	258	40	**4.2**	*0.020*	*
212895_s_at	ABR: active BCR-related gene	AL527773	612	45	285	58	**-1.6**	*0.013*	*	285	58	100	18	**-1.8**	*0.076*	
213404_s_at	RHEB: Ras homolog enriched in brain	BF033683	655	39	110	32	**-4.0**	*0.000*	*	110	32	1050	227	**5.3**	*0.051*	
214352_s_at	KRAS: v-Ki-ras2 Kirsten rat sarcoma viral oncogene homolog	BF673699	226	14	57	15	**-2.7**	*0.001*	*	57	15	78	17	**0.8**	*0.410*	
214393_at	RND2: Rho family GTPase 2	AI884814	113	15	47	7	**-1.7**	*0.033*	*	47	7	33	8	**-0.9**	*0.284*	
214665_s_at	CHP: calcium binding protein P22	AK000095	148	12	45	16	**-2.0**	*0.008*	*	45	16	45	11	**0.5**	*0.982*	
216689_x_at	ARHGAP1: Rho GTPase activating protein 1	U62794	525	74	84	20	**-4.1**	*0.021*	*	84	20	64	12	**-0.7**	*0.452*	
217777_s_at	LOC732402 /// PTPLAD1: protein tyrosine phosphatase-like A domain containing 1 /// simila	NM_016395	237	7	28	8	**-5.8**	*0.000*	*	28	8	51	11	**1.0**	*0.162*	
217793_at	RAB11B: RAB11B, member RAS oncogene family	AL575337	616	37	276	28	**-1.9**	*0.002*	*	276	28	175	35	**-1.1**	*0.093*	
217976_s_at	DYNC1LI1: dynein, cytoplasmic 1, light intermediate chain 1	NM_016141	154	19	64	10	**-1.7**	*0.023*	*	64	10	187	16	**2.2**	*0.005*	*
218186_at	RAB25: RAB25, member RAS oncogene family	NM_020387	200	9	78	14	**-1.9**	*0.004*	*	78	14	63	9	**-0.8**	*0.416*	
218360_at	RAB22A: RAB22A, member RAS oncogene family	NM_020673	125	12	31	8	**-2.8**	*0.005*	*	31	8	209	25	**4.5**	*0.013*	*
218700_s_at	RAB7L1: RAB7, member RAS oncogene family-like 1	BC002585	182	25	21	6	**-5.4**	*0.018*	*	21	6	13	5	**-0.7**	*0.414*	

7 Gene Ontology 'sphingolipid metabolic process' genes in 2122 annotated genes (genome-wide: 8/11929, p-value: 0.000680) ****

33646_g_at	GM2A: GM2 ganglioside activator	X61094	255	12	127	19	**-1.6**	*0.007*	*	127	19	128	19	**0.7**	*0.984*	
200866_s_at	PSAP: prosaposin (variant Gaucher disease and variant metachromatic leukodystrophy)	M32221	3133	213	47	24	**-35.6**	*0.004*	*	47	24	53	15	**0.5**	*0.843*	
202277_at	SPTLC1: serine palmitoyltransferase, long chain base subunit 1	AL568804	241	27	97	24	**-1.7**	*0.016*	*	97	24	493	38	**3.5**	*0.002*	*
208671_at	SERINC1: serine incorporator 1	AF164794	910	60	45	19	**-11.8**	*0.002*	*	45	19	456	52	**5.7**	*0.009*	*
209093_s_at	GBA /// GBAP: glucosidase, beta; acid (includes glucosylceramidase) /// glucosidase, beta; a	K02920	240	12	102	26	**-1.6**	*0.019*	*	102	26	61	20	**-0.8**	*0.286*	
212989_at	SGMS1: sphingomyelin synthase 1	AI377497	78	6	16	4	**-3.4**	*0.001*	*	16	4	167	49	**5.0**	*0.090*	
221268_s_at	SGPP1: sphingosine-1-phosphate phosphatase 1	NM_030791	71	4	13	3	**-3.7**	*0.000*	*	13	3	18	4	**0.8**	*0.399*	

26 Gene Ontology 'translation initiation factor activity' genes in 2122 annotated genes (genome-wide: 60/11929, p-value: 0.000048) *****

200004_at	EIF4G2: eukaryotic translation initiation factor 4 gamma, 2	NM_001418	3224	145	618	112	**-4.0**	*0.000*	*	618	112	1497	177	**1.7**	*0.019*	*
200005_at	EIF3S7: eukaryotic translation initiation factor 3, subunit 7 zeta, 66/67kDa	NM_003753	507	23	131	45	**-2.5**	*0.005*	*	131	45	436	28	**2.1**	*0.008*	*
200023_s_at	EIF3S5: eukaryotic translation initiation factor 3, subunit 5 epsilon, 47kDa	NM_003754	789	90	238	66	**-2.2**	*0.010*	*	238	66	562	150	**1.2**	*0.151*	
200596_s_at	EIF3S10: eukaryotic translation initiation factor 3, subunit 10 theta, 150/170kDa	BE614908	468	24	72	25	**-4.1**	*0.000*	*	72	25	242	52	**1.8**	*0.063*	
200776_s_at	BZW1 /// LOC151579: basic leucine zipper and W2 domains 1 /// similar to basic leucine zip	AL518328	725	73	80	18	**-6.3**	*0.009*	*	80	18	213	45	**1.6**	*0.081*	
201017_at	EIF1AX: eukaryotic translation initiation factor 1A, X-linked	BG149698	115	12	32	7	**-2.5**	*0.006*	*	32	7	172	27	**3.5**	*0.027*	*
201122_x_at	EIF5A: eukaryotic translation initiation factor 5A	BC000751	799	62	200	29	**-3.1**	*0.004*	*	200	29	116	25	**-1.2**	*0.089*	
201142_at	EIF2S1: eukaryotic translation initiation factor 2, subunit 1 alpha, 35kDa	AA577698	93	10	41	6	**-1.7**	*0.017*	*	41	6	80	13	**1.3**	*0.077*	
201435_s_at	EIF4E: eukaryotic translation initiation factor 4E	AW268640	546	32	88	16	**-4.7**	*0.001*	*	88	16	79	12	**-0.8**	*0.655*	
201530_x_at	EIF4A1: eukaryotic translation initiation factor 4A, isoform 1	NM_001416	2642	82	380	200	**-3.7**	*0.003*	*	380	200	301	109	**-0.2**	*0.753*	
201592_at	EIF3S3: eukaryotic translation initiation factor 3, subunit 3 gamma, 40kDa	NM_003756	1454	105	628	113	**-1.7**	*0.006*	*	628	113	1194	195	**1.3**	*0.082*	
202021_x_at	EIF1: eukaryotic translation initiation factor 1	AF083441	4086	494	162	43	**-16.5**	*0.015*	*	162	43	1571	367	**5.3**	*0.060*	
202461_at	EIF2B2: eukaryotic translation initiation factor 2B, subunit 2 beta, 39kDa	NM_014239	163	13	70	10	**-1.8**	*0.006*	*	70	10	163	24	**1.6**	*0.044*	*
203462_x_at	EIF3S9: eukaryotic translation initiation factor 3, subunit 9 eta, 116kDa	NM_003751	205	12	66	18	**-2.1**	*0.005*	*	66	18	67	14	**0.6**	*0.978*	
205321_at	EIF2S3: eukaryotic translation initiation factor 2, subunit 3 gamma, 52kDa	NM_001415	128	19	40	8	**-2.2**	*0.029*	*	40	8	42	4	**0.8**	*0.841*	
206116_s_at	TPM1: tropomyosin 1 (alpha)	NM_000366	139	20	29	14	**-2.5**	*0.014*	*	29	14	52	14	**0.8**	*0.306*	
208290_s_at	EIF5: eukaryotic translation initiation factor 5	NM_001969	103	6	23	4	**-3.4**	*0.001*	*	23	4	71	13	**2.0**	*0.054*	
208625_s_at	EIF4G1: eukaryotic translation initiation factor 4 gamma, 1	AF104913	178	14	26	13	**-3.7**	*0.001*	*	26	13	18	6	**-0.2**	*0.647*	
208726_s_at	EIF2S2: eukaryotic translation initiation factor 2, subunit 2 beta, 38kDa	BC000461	414	41	36	11	**-7.4**	*0.008*	*	36	11	377	149	**3.4**	*0.149*	
208756_at	EIF3S2: eukaryotic translation initiation factor 3, subunit 2 beta, 36kDa	U36764	473	15	62	12	**-5.8**	*0.000*	*	62	12	174	28	**1.9**	*0.041*	*
208887_at	EIF3S4: eukaryotic translation initiation factor 3, subunit 4 delta, 44kDa	BC000733	385	43	85	22	**-3.0**	*0.009*	*	85	22	82	14	**-0.6**	*0.896*	
209393_s_at	EIF4E2: eukaryotic translation initiation factor 4E family member 2	AF047695	250	16	104	23	**-1.7**	*0.008*	*	104	23	88	18	**-0.7**	*0.612*	
210949_s_at	EIF3S8 /// LOC728689: eukaryotic translation initiation factor 3, subunit 8, 110kDa /// similar	BC000533	1573	158	66	26	**-14.1**	*0.009*	*	66	26	120	34	**0.8**	*0.282*	
211937_at	EIF4B: eukaryotic translation initiation factor 4B	NM_001417	1849	123	825	124	**-1.8**	*0.004*	*	825	124	560	167	**-0.9**	*0.277*	
212581_x_at	GAPDH: glyceraldehyde-3-phosphate dehydrogenase	BE561479	6935	945	196	45	**-23.5**	*0.019*	*	196	45	291	112	**0.5**	*0.496*	
221494_x_at	EIF3S12: eukaryotic translation initiation factor 3, subunit 12	AF085358	778	60	117	38	**-4.2**	*0.002*	*	117	38	217	41	**1.1**	*0.151*	

39 Gene Ontology 'unfolded protein binding' genes in 2122 annotated genes (genome-wide: 102/11929, p-value: 0.000013) *****

200063_s_at	NPM1: nucleophosmin (nucleolar phosphoprotein B23, numatrin)	BC002398	3638	242	1781	178	**-1.7**	*0.005*	*	1781	178	2655	282	**1.2**	*0.070*	
200064_at	HSP90AB1: heat shock protein 90kDa alpha (cytosolic), class B member 1	AF275719	4584	178	55	24	**-48.5**	*0.001*	*	55	24	278	88	**2.1**	*0.118*	
200598_s_at	HSP90B1: heat shock protein 90kDa beta (Grp94), member 1	AI582238	1147	64	57	23	**-12.1**	*0.001*	*	57	23	181	37	**1.7**	*0.059*	
200627_at	PTGES3: prostaglandin E synthase 3 (cytosolic)	BC003005	1895	147	168	74	**-6.4**	*0.002*	*	168	74	1443	184	**4.7**	*0.011*	*
200662_s_at	TOMM20: translocase of outer mitochondrial membrane 20 homolog (yeast)	NM_014765	1072	159	50	16	**-12.9**	*0.022*	*	50	16	622	45	**8.0**	*0.003*	*
200691_s_at	HSPA9: heat shock 70kDa protein 9 (mortalin)	BC000478	588	31	182	71	**-2.0**	*0.017*	*	182	71	473	61	**1.5**	*0.037*	
200800_s_at	HSPA1A /// HSPA1B: heat shock 70kDa protein 1A /// heat shock 70kDa protein 1B	NM_005345	305	52	41	14	**-4.3**	*0.030*	*	41	14	440	380	**0.0**	*0.403*	
200806_s_at	HSPD1: heat shock 60kDa protein 1 (chaperonin)	BE256479	912	97	32	15	**-15.6**	*0.011*	*	32	15	138	36	**2.0**	*0.083*	
200881_s_at	DNAJA1: DnaJ (Hsp40) homolog, subfamily A, member 1	NM_001539	1904	111	580	148	**-2.3**	*0.003*	*	580	148	2396	243	**2.8**	*0.006*	*
200935_at	CALR: calreticulin	NM_004343	173	13	81	17	**-1.5**	*0.016*	*	81	17	36	7	**-1.3**	*0.110*	
200967_at	PPIB: peptidylprolyl isomerase B (cyclophilin B)	NM_000942	914	125	58	28	**-8.3**	*0.017*	*	58	28	91	25	**0.7**	*0.422*	
201293_x_at	PPIA: peptidylprolyl isomerase A (cyclophilin A)	NM_021130	6043	363	1852	541	**-2.2**	*0.005*	*	1852	541	2052	427	**0.6**	*0.787*	
201327_s_at	CCT6A: chaperonin containing TCP1, subunit 6A (zeta 1)	NM_001762	339	29	119	32	**-1.9**	*0.007*	*	119	32	330	31	**1.9**	*0.009*	*
201781_s_at	AIP: aryl hydrocarbon receptor interacting protein	AL558532	158	5	54	8	**-2.3**	*0.001*	*	54	8	69	8	**0.9**	*0.267*	
201946_s_at	CCT2: chaperonin containing TCP1, subunit 2 (beta)	AL545982	273	42	32	16	**-4.4**	*0.019*	*	32	16	77	26	**0.9**	*0.226*	
203045_at	NINJ1: ninjurin 1	NM_004148	92	4	34	8	**-1.9**	*0.010*	*	34	8	30	5	**-0.7**	*0.690*	
203293_s_at	LMAN1: lectin, mannose-binding, 1	NM_005570	141	12	28	8	**-3.4**	*0.002*	*	28	8	28	9	**0.4**	*0.952*	
204299_at	FUSIP1 /// LOC727922: FUS interacting protein (serine/arginine-rich) 1 /// similar to FUS-inte	NM_021993	102	13	23	15	**-2.1**	*0.017*	*	23	15	233	17	**4.8**	*0.001*	*
204517_at	PPIC: peptidylprolyl isomerase C (cyclophilin C)	BE962749	126	22	37	7	**-2.2**	*0.044*	*	37	7	306	131	**2.4**	*0.176*	
205361_s_at	PFDN4: prefoldin subunit 4	AI718295	187	11	16	3	**-9.0**	*0.003*	*	16	3	62	10	**2.6**	*0.038*	
206350_at	APCS: amyloid P component, serum	NM_001639	110	19	39	9	**-1.7**	*0.049*	*	39	9	45	8	**0.7**	*0.672*	
207132_x_at	PFDN5: prefoldin subunit 5	NM_002624	2565	160	67	40	**-19.1**	*0.003*	*	67	40	70	46	**0.0**	*0.952*	
207714_s_at	SERPINH1: serpin peptidase inhibitor, clade H (heat shock protein 47), member 1, (collager	NM_004353	474	98	47	14	**-5.7**	*0.047*	*	47	14	32	11	**-0.7**	*0.432*	
208687_x_at	HSPA8: heat shock 70kDa protein 8	AF352832	3904	173	180	114	**-10.6**	*0.000*	*	180	114	1131	361	**2.2**	*0.108*	
208696_at	CCT5: chaperonin containing TCP1, subunit 5 (epsilon)	AF275798	847	63	318	38	**-2.1**	*0.004*	*	318	38	667	167	**1.2**	*0.167*	
208778_s_at	TCP1: t-complex 1	BC000665	652	64	208	50	**-2.2**	*0.006*	*	208	50	1454	122	**4.9**	*0.004*	*
208829_at	TAPBP: TAP binding protein (tapasin)	AF029750	552	28	141	68	**-2.2**	*0.016*	*	141	68	68	15	**-0.4**	*0.396*	
208852_s_at	CANX: calnexin	AI761759	1721	49	51	15	**-22.5**	*0.000*	*	51	15	315	82	**3.1**	*0.079*	
209157_at	DNAJA2: DnaJ (Hsp40) homolog, subfamily A, member 2	AF011793	184	10	27	9	**-4.4**	*0.000*	*	27	9	188	12	**4.5**	*0.001*	*
210211_s_at	HSP90AA1: heat shock protein 90kDa alpha (cytosolic), class A member 1	AF028832	2583	226	456	200	**-3.2**	*0.002*	*	456	200	3489	285	**4.4**	*0.002*	*
211936_at	HSPA5: heat shock 70kDa protein 5 (glucose-regulated protein, 78kDa)	AF216292	1873	128	70	20	**-18.0**	*0.004*	*	70	20	447	143	**2.8**	*0.116*	
212135_s_at	ATP2B4: ATPase, Ca++ transporting, plasma membrane 4	AW517686	705	28	176	36	**-3.0**	*0.000*	*	176	36	167	30	**-0.7**	*0.864*	
215735_s_at	TSC2: tuberous sclerosis 2	AC005600	174	5	48	6	**-3.0**	*0.000*	*	48	6	35	5	**-1.0**	*0.162*	
218138_at	MKKS: McKusick-Kaufman syndrome	NM_018848	213	23	45	15	**-2.9**	*0.005*	*	45	15	239	65	**2.6**	*0.088*	
218409_s_at	DNAJC1: DnaJ (Hsp40) homolog, subfamily C, member 1	NM_022365	403	17	165	58	**-1.5**	*0.046*	*	165	58	194	45	**0.6**	*0.722*	
218495_at	UXT: ubiquitously-expressed transcript	NM_004182	641	29	249	62	**-1.8**	*0.012*	*	249	62	396	130	**0.7**	*0.386*	
218976_at	DNAJC12: DnaJ (Hsp40) homolog, subfamily C, member 12	NM_021800	2178	296	747	109	**-2.1**	*0.029*	*	747	109	1493	374	**1.1**	*0.177*	
220012_at	ERO1LB: ERO1-like beta (S. cerevisiae)	NM_019891	1709	138	141	54	**-7.2**	*0.003*	*	141	54	531	93	**2.1**	*0.033*	*
221781_s_at	DNAJC10: DnaJ (Hsp40) homolog, subfamily C, member 10	BG168666	162	22	23	5	**-4.9**	*0.020*	*	23	5	54	9	**1.6**	*0.052*	

37 Gene Ontology 'vesicle-mediated transport' genes in 2122 annotated genes (genome-wide: 103/11929, p-value: 0.000075) *****

200613_at	AP2M1: adaptor-related protein complex 2, mu 1 subunit	NM_004068	1136	67	147	61	**-4.6**	*0.000*	*	147	61	74	14	**-0.6**	*0.351*	
200615_s_at	AP2B1: adaptor-related protein complex 2, beta 1 subunit	AL567295	140	11	29	9	**-3.1**	*0.001*	*	29	9	60	12	**1.2**	*0.108*	
200720_s_at	ACTR1A: ARP1 actin-related protein 1 homolog A, centractin alpha (yeast)	AL532341	201	20	78	22	**-1.7**	*0.015*	*	78	22	89	14	**0.7**	*0.694*	
200927_s_at	RAB14: RAB14, member RAS oncogene family	AA919115	550	32	44	6	**-9.9**	*0.003*	*	44	6	233	42	**3.5**	*0.043*	*

201359_at	COPB1: coatomer protein complex, subunit beta 1	NM_016451	766	66	232	63	-2.2	0.004	*	232	63	675	99	1.8	0.026	*
201556_s_at	VAMP2: vesicle-associated membrane protein 2 (synaptobrevin 2)	BC002737	810	29	174	71	-2.8	0.006	*	174	71	114	30	-0.5	0.500	
201567_s_at	GOLGA4: golgi autoantigen, golgin subfamily a, 4	NM_002078	130	10	29	9	-3.0	0.002	*	29	9	311	109	4.2	0.122	
201583_s_at	SEC23B: Sec23 homolog B (S. cerevisiae)	NM_006363	327	19	48	8	-5.3	0.002	*	48	8	181	25	2.7	0.025	*
201613_s_at	AP1G2: adaptor-related protein complex 1, gamma 2 subunit	BC000519	135	15	55	10	-1.8	0.014	*	55	10	29	4	-1.3	0.109	
202118_s_at	CPNE3: copine III	AA541758	278	13	18	6	-10.2	0.001	*	18	6	25	5	0.8	0.421	
202120_x_at	AP2S1: adaptor-related protein complex 2, sigma 1 subunit	NM_004069	377	46	26	12	-7.8	0.012	*	26	12	42	10	0.7	0.390	
202211_at	ARFGAP3: ADP-ribosylation factor GTPase activating protein 3	BC005122	163	24	61	17	-1.7	0.029	*	61	17	476	27	5.3	0.000	*
202546_at	VAMP8: vesicle-associated membrane protein 8 (endobrevin)	NM_003761	199	14	16	6	-7.5	0.002	*	16	6	19	5	0.5	0.725	
202829_s_at	SYBL1: synaptobrevin-like 1	NM_005638	145	11	37	18	-2.1	0.011	*	37	18	336	26	5.0	0.001	*
203142_s_at	AP3B1: adaptor-related protein complex 3, beta 1 subunit	NM_003664	488	33	83	22	-4.0	0.001	*	83	22	140	21	1.1	0.130	
203300_x_at	AP1S2: adaptor-related protein complex 1, sigma 2 subunit	NM_003916	143	10	46	10	-2.3	0.002	*	46	10	60	11	0.8	0.403	
203311_s_at	ARF6: ADP-ribosylation factor 6	M57763	179	4	71	10	-2.0	0.003	*	71	10	45	7	-1.1	0.096	
205423_at	AP1B1: adaptor-related protein complex 1, beta 1 subunit	NM_001127	449	23	186	36	-1.8	0.006	*	186	36	97	24	-1.2	0.120	
207791_s_at	RAB1A: RAB1A, member RAS oncogene family	NM_004161	340	54	10	4	-19.3	0.025	*	10	4	36	7	2.0	0.047	
208684_at	COPA: coatomer protein complex, subunit alpha	U24105	651	22	263	40	-2.0	0.003	*	263	40	127	9	-1.5	0.072	
208750_s_at	ARF1: ADP-ribosylation factor 1	AA580004	1646	28	21	7	-48.8	0.000	*	21	7	21	6	-0.4	0.949	
209452_s_at	VTI1B: vesicle transport through interaction with t-SNAREs homolog 1B (yeast)	AF035824	306	37	39	11	-5.0	0.013	*	39	11	125	20	2.0	0.029	*
209515_s_at	RAB27A: RAB27A, member RAS oncogene family	U38654	305	36	58	28	-2.8	0.007	*	58	28	370	85	3.0	0.054	
209678_s_at	PRKCI: protein kinase C, iota	L18964	185	13	46	12	-2.8	0.001	*	46	12	92	37	0.7	0.335	
210658_s_at	GGA2: golgi associated, gamma adaptin ear containing, ARF binding protein 2	BC000284	127	11	57	12	-1.6	0.014	*	57	12	108	23	1.1	0.145	
211749_s_at	VAMP3: vesicle-associated membrane protein 3 (cellubrevin)	BC005941	549	41	32	8	-11.8	0.005	*	32	8	61	14	1.1	0.161	
212439_at	IHPK1: inositol hexaphosphate kinase 1	BE614199	264	22	84	20	-2.2	0.004	*	84	20	93	14	0.7	0.743	
214257_s_at	SEC22B: SEC22 vesicle trafficking protein homolog B (S. cerevisiae)	AA890010	525	34	106	30	-3.3	0.001	*	106	30	270	123	0.6	0.311	
215236_s_at	PICALM: phosphatidylinositol binding clathrin assembly protein	AV721177	107	3	12	3	-6.2	0.000	*	12	3	14	4	0.6	0.626	
215735_s_at	TSC2: tuberous sclerosis 2	AC005600	174	5	48	6	-3.0	0.000	*	48	6	35	5	-1.0	0.162	
216295_s_at	CLTA: clathrin, light chain (Lca)	X81636	1271	71	85	26	-9.9	0.001	*	85	26	224	48	1.5	0.080	
217726_at	COPZ1: coatomer protein complex, subunit zeta 1	NM_016057	306	21	106	27	-2.0	0.005	*	106	27	218	17	1.4	0.033	
217749_at	COPG: coatomer protein complex, subunit gamma	NM_016128	319	30	131	26	-1.8	0.009	*	131	26	53	11	-1.5	0.082	
217959_s_at	TRAPPC4: trafficking protein particle complex 4	NM_016146	247	30	26	7	-6.2	0.015	*	26	7	155	11	4.1	0.002	*
218193_s_at	GOLT1B: golgi transport 1 homolog B (S. cerevisiae)	NM_016072	135	12	67	11	-1.5	0.015	*	67	11	445	112	3.7	0.076	
218261_at	AP1M2: adaptor-related protein complex 1, mu 2 subunit	NM_005498	127	10	55	12	-1.7	0.011	*	55	12	46	11	-0.7	0.585	
220995_at	STXBP6: syntaxin binding protein 6 (amisyn)	NM_014178	457	13	167	54	-1.8	0.028	*	167	54	79	18	-0.9	0.240	

48 Gene Ontology 'anti-apoptosis' genes in 2122 annotated genes (genome-wide: 141/11929, p-value: 0.000027) ***

200021_at	CFL1: cofilin 1 (non-muscle)	NM_005507	4752	156	2144	122	-2.0	0.000	*	2144	122	870	83	-2.1	0.002	*
200046_at	DAD1: defender against cell death 1	NM_001344	1286	72	184	95	-3.8	0.001	*	184	95	737	145	1.9	0.041	*
200063_s_at	NPM1: nucleophosmin (nucleolar phosphoprotein B23, numatrin)	BC002398	3638	242	1781	178	-1.7	0.005	*	1781	178	2655	282	1.2	0.070	
200598_s_at	HSP90B1: heat shock protein 90kDa beta (Grp94), member 1	AI582238	1147	64	57	23	-12.1	0.001	*	57	23	181	37	1.7	0.059	
200638_s_at	YWHAZ: tyrosine 3-monooxygenase/tryptophan 5-monooxygenase activation protein, zeta p	BC003623	1433	79	30	7	-33.8	0.003	*	30	7	246	143	0.4	0.269	
200679_x_at	HMGB1: high-mobility group box 1	BE311760	716	40	52	12	-10.0	0.002	*	52	12	32	11	-0.9	0.280	
200681_at	GLO1: glyoxalase I	NM_006708	853	51	166	57	-3.2	0.001	*	166	57	744	48	2.8	0.002	*
200691_s_at	HSPA9: heat shock 70kDa protein 9 (mortalin)	BC000478	588	31	182	71	-2.0	0.017	*	182	71	473	61	1.5	0.037	
200782_at	ANXA5: annexin A5	NM_001154	1747	162	158	47	-7.2	0.007	*	158	47	924	231	3.0	0.075	
200787_s_at	PEA15: phosphoprotein enriched in astrocytes 15	BC002426	183	5	52	8	-2.7	0.001	*	52	8	34	5	-1.1	0.143	
200798_x_at	MCL1: myeloid cell leukemia sequence 1 (BCL2-related)	NM_021960	1032	50	30	8	-23.3	0.002	*	30	8	821	93	17.7	0.013	*
200800_s_at	HSPA1A /// HSPA1B: heat shock 70kDa protein 1A /// heat shock 70kDa protein 1B	NM_005345	305	52	41	14	-4.3	0.030	*	41	14	440	380	0.0	0.403	
200824_at	GSTP1: glutathione S-transferase pi	NM_000852	335	69	44	17	-4.0	0.045	*	44	17	91	24	1.0	0.191	
201168_x_at	ARHGDIA /// LOC728908: Rho GDP dissociation inhibitor (GDI) alpha /// similar to Rho GDI	NM_004309	1128	58	135	36	-5.7	0.000	*	135	36	49	16	-1.4	0.127	
201631_s_at	IER3: immediate early response 3	NM_003897	5920	541	1996	943	-1.6	0.033	*	1996	943	4410	867	1.1	0.133	
201687_s_at	API5: apoptosis inhibitor 5	NM_006595	154	13	30	12	-3.0	0.002	*	30	12	121	24	2.1	0.044	*
201841_s_at	HSPB1: heat shock 27kDa protein 1	NM_001540	1481	85	35	18	-22.3	0.002	*	35	18	46	12	0.6	0.674	
202300_at	HBXIP: hepatitis B virus x interacting protein	NM_006402	559	30	76	21	-5.0	0.000	*	76	21	750	38	6.7	0.000	*
203460_s_at	PSEN1: presenilin 1 (Alzheimer disease 3)	NM_007318	121	8	46	15	-1.7	0.019	*	46	15	99	18	1.2	0.088	
203619_s_at	FAIM2: Fas apoptotic inhibitory molecule 2	NM_012306	112	5	20	10	-3.1	0.003	*	20	10	36	5	1.0	0.227	
203627_at	IGF1R: insulin-like growth factor 1 receptor	AI830698	190	5	57	10	-2.6	0.001	*	57	10	43	7	-0.9	0.307	
204540_at	EEF1A2: eukaryotic translation elongation factor 1 alpha 2	NM_001958	1557	147	273	93	-3.5	0.003	*	273	93	25	7	-4.5	0.116	
204614_at	SERPINB2: serpin peptidase inhibitor, clade B (ovalbumin), member 2	NM_002575	440	49	122	26	-2.5	0.010	*	122	26	451	226	0.7	0.282	
206360_s_at	SOCS3: suppressor of cytokine signaling 3	NM_003955	290	10	129	13	-1.9	0.001	*	129	13	76	8	-1.3	0.032	
206724_at	CBX4: chromobox homolog 4 (Pc class homolog, Drosophila)	NM_003655	138	15	34	5	-3.1	0.013	*	34	5	28	7	-0.8	0.547	
207005_s_at	BCL2: B-cell CLL/lymphoma 2	NM_000657	178	17	55	25	-1.8	0.020	*	55	25	40	18	-0.3	0.662	
207163_s_at	AKT1: v-akt murine thymoma viral oncogene homolog 1	NM_005163	172	18	52	11	-2.4	0.009	*	52	11	37	9	-0.8	0.358	
207180_s_at	HTATIP2: HIV-1 Tat interactive protein 2, 30kDa	NM_006410	200	17	89	19	-1.6	0.013	*	89	19	234	56	1.5	0.112	
208097_s_at	TXNDC1: thioredoxin domain containing 1	NM_030755	207	15	37	11	-3.7	0.001	*	37	11	60	10	1.0	0.187	
208296_x_at	TNFAIP8: tumor necrosis factor, alpha-induced protein 8	NM_014350	217	9	71	16	-2.2	0.003	*	71	16	463	164	2.6	0.138	
208485_x_at	CFLAR: CASP8 and FADD-like apoptosis regulator	NM_003879	607	47	219	29	-2.2	0.004	*	219	29	143	30	-1.0	0.141	
208945_s_at	BECN1: beclin 1 (coiled-coil, myosin-like BCL2 interacting protein)	NM_003766	306	27	140	19	-1.7	0.009	*	140	19	151	18	0.8	0.713	
209090_s_at	SH3GLB1: SH3-domain GRB2-like endophilin B1	AL049597	146	17	47	10	-2.1	0.012	*	47	10	162	14	2.5	0.003	*
209782_s_at	DBP: D site of albumin promoter (albumin D-box) binding protein	U79283	187	12	83	9	-1.9	0.003	*	83	9	47	4	-1.4	0.037	
209875_s_at	SPP1: secreted phosphoprotein 1 (osteopontin, bone sialoprotein I, early T-lymphocyte acti	M83248	3806	382	44	20	-48.3	0.010	*	44	20	270	120	1.5	0.199	
210018_x_at	MALT1: mucosa associated lymphoid tissue lymphoma translocation gene 1	AB026118	93	4	30	9	-2.1	0.009	*	30	9	125	28	2.3	0.064	
211475_s_at	BAG1: BCL2-associated athanogene	AF116273	312	24	56	13	-4.0	0.002	*	56	13	113	20	1.3	0.082	
211573_x_at	TGM2: transglutaminase 2 (C polypeptide, protein-glutamine-gamma-glutamyltransferase)	M98478	94	10	29	10	-2.0	0.010	*	29	10	32	8	0.6	0.832	
211658_at	PRDX2: peroxiredoxin 2	L19185	91	4	35	6	-2.0	0.003	*	35	6	27	5	-0.8	0.370	
211936_at	HSPA5: heat shock 70kDa protein 5 (glucose-regulated protein, 78kDa)	AF216292	1873	128	70	20	-18.0	0.004	*	70	20	447	143	2.8	0.116	
212312_at	BCL2L1: BCL2-like 1	AL117381	360	33	135	27	-1.9	0.007	*	135	27	91	14	-0.9	0.251	
212501_at	CEBPB: CCAAT/enhancer binding protein (C/EBP), beta	AL564683	781	113	98	26	-5.1	0.022	*	98	26	459	113	2.5	0.079	
214882_s_at	SFRS2: splicing factor, arginine/serine-rich 2	BG254869	69	5	8	5	-4.2	0.001	*	8	5	39	11	1.9	0.092	
216508_x_at	HMG1L1 /// HMGB1 /// LOC645292 /// LOC731809: high-mobility group box 1 /// high-mobili	AC007277	178	14	46	10	-2.7	0.002	*	46	10	44	14	-0.6	0.887	
216520_s_at	TPT1: tumor protein, translationally-controlled 1	AF072098	6066	216	418	121	-9.8	0.000	*	418	121	3683	347	5.8	0.006	*
216598_s_at	CCL2: chemokine (C-C motif) ligand 2	S69738	512	66	61	24	-4.8	0.013	*	61	24	35	5	-0.6	0.386	
218056_at	BFAR: bifunctional apoptosis regulator	NM_016561	268	17	113	11	-2.0	0.003	*	113	11	93	16	-0.9	0.360	
218229_s_at	POGK: pogo transposable element with KRAB domain	NM_017542	168	11	45	11	-2.6	0.001	*	45	11	59	9	0.8	0.385	
