# Supplementary material for: Clusters of Conserved Beta Cell Marker Genes for Assessment of Beta Cell Phenotype
Source: PLoS One. 2011 Sep 2;6(9):e24134. doi: 10.1371/journal.pone.0024134 (PMC3166300; doi:10.1371/journal.pone.0024134)
Supplement: Table S3 — DiRE analysis of transcriptional regulators of the beta cell phenotype. DiRE was used to retrieve transcription factor binding sites (TFBs) that are statistically overrepresented in beta cell marker genes versus random reference gene sets (±5000 genes) of the human (hg18), mouse (mm9) and rat (rn4) genomes. Score indicate statistical importance (ranging from 0 to 1). For each species, the analysis was run on the whole set of beta cell markers (all), and also separately on the 3 outstanding clusters of neuro-endocrine genes (cluster A, see Fig. 3), beta cell-selective genes (cluster B, see Fig. 2) and markers shared with immune and gut cells (cluster C, see suppl. Fig. 3) genes. Data (human analysis) are graphically presented in Fig. 6. (PDF) [file pone.0024134.s007.pdf]

Table S3:TFB consensus site enrichment using DiRE algorithm on all conserved beta cell marker genes and individual clusters

		Human				Mouse				Rat			
	Mammalian	Importance in gene cluster				Importance in gene cluster				Importance in gene cluster			
TFBs	Transcription Factor	All	A	B	C	All	A	B	C	All	A	B	C
TAL1BETAITF2	Tal1/beta-ITF2	**0.12**	0.03	**0.72**	0.02	0.02	0.01	**0.27**	0.06	**0.12**	**0.14**	**0.13**	0.02
S8	Prx2, Prrx2	**0.48**	**0.19**	**0.69**		0.06				**0.12**	0.00		**0.31**
TCF11	Nfe2l1, Nrf1	**0.24**	0.01		**0.57**	0.03	0.00		0.07	**0.12**	0.09	0.03	**0.73**
P53	P53	**0.51**		0.09	0.08		0.07		0.04		0.04		0.04
MYOGNF1	Myog	0.04	0.04	**0.36**	0.03	0.02	0.01	0.03			0.02	0.05	
STAT4	Stat4	0.10		**0.32**	0.06		0.01		0.00	0.02		**0.16**	
OTX	Otx1/Otx2	0.07			**0.31**				0.08				
CART1	Alx1	**0.23**	**0.17**		**0.23**	**0.15**	0.00		**0.20**				
TST1	Tst1, Oct6	**0.23**	0.08	0.03		**0.32**		**0.16**	**0.14**	0.04		0.02	0.08
SOX9_B1	**Sox9**	**0.23**	0.01	0.03		**0.57**	**0.20**	0.01	**0.43**	**0.11**		0.06	0.07
NKX62	**Nkx6.2**	0.03	0.01	**0.23**		0.02	**0.16**	**0.18**			0.00	**0.17**	
VBP	Vbp	**0.22**		0.07	0.04		0.03	0.00		0.01		0.01	0.01
OCT1	Oct1, POU2AF1	**0.21**		0.05	**0.18**	0.08		0.04	0.05	**0.14**			0.07
OCT4	Oct4, POU5F1	0.08		0.08	**0.21**	0.05		0.01		**0.25**	**0.13**	**0.16**	
RSRFC4	Mef2a	0.02	**0.21**		0.01		0.05		0.07	**0.18**	**0.11**		0.07
RUSH1A	Hltf		0.02	**0.20**	0.05	**0.62**		**0.35**	**0.81**	0.03			0.09
ATF6	**Atf6**				**0.19**		0.01		**0.22**				0.05
RFX1	Rfx1	0.04	**0.18**	0.10		0.01		**0.13**					
NFE2	NF-E2		**0.18**	0.01	0.01	0.00	0.01				0.01		0.02
OLF1	Ebf1/Olf1	0.07	**0.18**				0.03	0.03			0.09	0.00	
NKX25	Nkx2.5	**0.18**	0.09	**0.16**				0.06		**0.16**	0.08	0.08	
RFX	Rfx family	0.01	**0.16**	0.01	0.00	**0.12**	**0.19**	0.10	0.04	0.04			
RORA1	Rora1		**0.16**		0.08	0.01				**0.13**	0.08		0.01
PIT1	Pit-1	0.04		0.07	**0.16**							0.00	
XFD2	HNF3/Fork Head	0.05	**0.16**		**0.14**					**0.26**	**0.32**	**0.11**	0.06
CREB	**Creb**	**0.16**		0.00	0.05	0.02		0.05		**0.14**			**0.13**
PAX3	Pax3	0.01		**0.16**	0.01				**0.18**	0.00			
SF1	Sf1/Nr5a1/Ad4bp		0.03	**0.16**	0.02				0.04	0.00			
OCT	Oct family	0.05	**0.16**	**0.11**		0.01	0.04			0.07	**0.15**		
HFH8	**HNF3**	**0.15**	0.05	0.03	0.06						**0.37**	0.01	
CDXA				**0.15**				0.03					0.00
GRE	GRE	0.04	**0.15**		0.06	**0.22**		0.05	**0.27**		0.02	0.02	
NF1	Ctf/Nf1	0.06		**0.15**	0.10					0.05		0.06	0.00
STAF	Staf				**0.15**	0.00			0.07				
ZID	Zbtb6	**0.15**	**0.13**	0.01			0.00	0.00			0.00		
HFH1	**HNF3**			**0.14**				0.02		0.05			0.01
BACH2	Bach2		0.01		**0.14**				0.06				
LEF1TCF1	Lef1/Tcf1	**0.14**							0.01				
IPF1	**IPF1/PDX1**	**0.13**	0.04	0.09		**0.17**		**0.48**		**0.13**	**0.12**		0.06
SRF	SRF	0.04		0.02	**0.12**	0.05			**0.11**	0.01			0.06
PAX6	**Pax6**		**0.12**		0.01	0.01				0.05	0.09		0.03
ALPHACP1	AlphaCP-1		**0.12**	0.04	0.01				0.00		0.02		0.04
PITX2	Pitx2	**0.12**		**0.11**	0.04	**0.36**	0.00	**0.33**	0.01			0.05	
MRF2	Mrf2				**0.12**								0.02
TITF1	Titf1/Nkx2.1				**0.12**			0.01		0.00		0.06	0.05
TATA	Tata	0.01	**0.11**	0.03	0.00			0.01					
TCF4	**Tcf7l2**	0.05	0.08	**0.11**			0.05	**0.10**	0.01		0.00		0.08
NKX22	**Nkx2.2**	0.04	**0.11**			0.03	0.00	0.00	0.01	**0.25**			**0.16**

Legend

Cluster A	neuron-beta
Cluster B	beta-specific
Cluster C	beta-hemato-gut
