# Supplementary material for: Clusters of Conserved Beta Cell Marker Genes for Assessment of Beta Cell Phenotype
Source: PLoS One. 2011 Sep 2;6(9):e24134. doi: 10.1371/journal.pone.0024134 (PMC3166300; doi:10.1371/journal.pone.0024134)
Supplement: Table S4 — Transcription factor consensus sites identified by 2 independent algorithms. DNA consensus sites (TRANSFAC) with potential for transcription factor binding were identified by DiRE and Matrix Scan; 27 consensus sites were identified by both algorithms (ochre), 27 only by the more stringent Matrix Scan (blue), and 77 only by DiRE (yellow). Marked in bold are transcription factors with established role in beta cell development and physiology Data are graphically presented in Fig. 6. (PDF) [file pone.0024134.s008.pdf]

Table S4: transcription factor consensus sites overrepresented in the conserved beta cell marker genes

Matrix Scan only	Matrix Scan and DiRE		DiRE only		
n=27	*n=27*		*n=77*		
**AML1**	**ATF3**	**ATF6**	AFP1	IRF1	S8
**AP1 / c-Fos c-Jun**	**CREB**	**SREBP1**	ALPHACP1	LEF1TCF1	SEF1
**AP2REP**	**USF**	**IPF1/PDX1**	AP3	MEF3	SF1
**AP4**	**GRE**		BACH2	MIF1	STAF
**AR**	**NFAT**		BRCA	MMEF2	STAT
**AREB6**	TAL1		CART1	NANOG	TAACC
**CLOCK BMAL**	MEIS1BHOXA9		CBF	NF1	TATA
**c-MYC**	HLF		CDC5	NFE2	TBP
**DEC1/DEC2**	**NKX22**		CDXA	**NKX61**	TCF11
**E2A**	**OCT1**		CIZ	OCT4	**TCF4**
**ICSBP**	PR		CMAF	OLF1	TFIII
**LMO2COM**	RFX1		CREL	OTX	TITF1
**MAX**	MRF2		DBP	P53	TST1
**MITF TFE**	STRA13		E2F1DP1RB	**PAX6**	UF1H3BETA
**MYOD1**	MYOGENIN		E2F4DP1	**PBX1**	VBP
**NERF**	YY1		EGR1	**PIT1**	VJUN
**NF-Y**	TBX5		ERR1	PITX2	XFD1
**NNMYC**	TEL2		EVI1	PLZF	ZEC
**NRSE**	**XBP1**		FXR_IR1	POU6F1	ZIC1
**OSF2**	E4F1		GATA	PPARG	ZID
**PAX4**	EBOX		GCM	RFX	HSF
**PU1**	GFI1B		GZF1	RORA1	HTF
**RBPJK**	**ARNT/HIF**		HMX1	RP58	
**SMAD3**	**ETS1**		**HNF6**	RSRFC4	
**SZF11**	ETS2		HOXA3	RUSH1A	
**TGIF**	CEBP		HP1SITEFACTC	SRF	
**ZF5**	**FOX**		SOX9_B1	SRY	
